# Novel asymmetric photodimerization reaction of coumarin derivatives bearing a chiral 2-oxazolidinone auxiliary†

**DOI:** 10.1039/c9ra00822e

**Published:** 2019-04-23

**Authors:** Kennosuke Itoh, Fumiya Odate, Takuma Karikomi, Keishi Obe, Tsutomu Miyamori, Hideaki Kamiya, Kenji Yoza, Kenichiro Nagai, Hideaki Fujii, Hiroyuki Suga, Ken Tokunaga

**Affiliations:** Laboratory of Medicinal Chemistry, School of Pharmacy, Kitasato University 5-9-1 Shirokane, Minato-ku Tokyo 108-8641 Japan itok@pharm.kitasato-u.ac.jp; Medicinal Research Laboratories, School of Pharmacy, Kitasato University 5-9-1 Shirokane, Minato-ku Tokyo 108-8641 Japan; Applied Chemistry and Chemical Engineering Program, Graduate School, Kogakuin University 2665-1 Nakano Hachioji Tokyo 192-0015 Japan; Department of Applied Chemistry, Faculty of Engineering, Kogakuin University 2665-1 Nakano Hachioji Tokyo 192-0015 Japan; Department of Applied Chemistry, School of Advanced Engineering, Kogakuin University 2665-1 Nakano Hachioji Tokyo 192-0015 Japan; Department of Materials Chemistry, Faculty of Engineering, Shinshu University 4-17-1 Wakasato Nagano 380-8553 Japan; Bruker Japan 3-9, Moriya-cho, Kanagawa-ku Yokohama 221-0022 Japan; Division of Liberal Arts, Center for Promotion of Higher Education, Kogakuin University 2665-1 Nakano Hachioji Tokyo 192-0015 Japan

## Abstract

A novel asymmetric photodimerization reaction of coumarin derivatives bearing the (*S*)-4-benzyl-2-oxazolidinone auxiliary provides only the *syn*-head-to-tail (*syn*-HT) dimer with moderate diastereoselectivity (up to 75 : 25). The mechanism of complete *syn*-HT selectivity and moderate diastereoselectivity is proposed based on the result of density functional theory (DFT) calculation. The benzyl group of the (*S*)-4-benzyl-2-oxazolidinone auxiliary in combination with a Lewis acid exerts effective diastereofacial shielding of the reaction site.

## Abstract 2

Photodimerization of chiral coumarin-3-carboxamide affords the *syn*-HT dimer with moderate diastereoselectivity and we propose a mechanism on the basis of DFT calculations.

## Graphical abstract

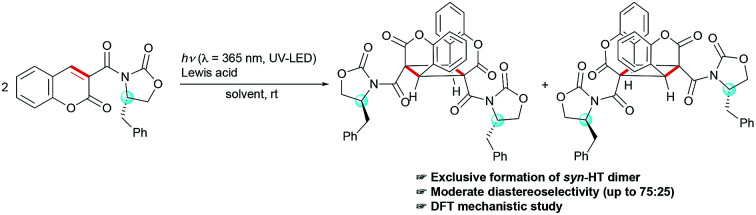

The photodimerization reactions of coumarin derivatives have been recognized as attractive photochemical transformation reactions in terms of their photophysical properties as well as their photochemical properties, from the theoretical point of view.^1^ Recently, photodimerization reactions of coumarins have become increasingly important in several progressive research areas, *e.g.*, drug delivery by the use of coumarin-modified mesoporous silica MCM-41^2*a*^ and the use of coumarin-modified polymeric nanoparticles,^2*b*,2*c*^ chemical biology,^2*d*^ 3D cell culture,^2*e*^ photopatterning of ion gels,^2*f*^ reversible two-photon optical data storage,^2*g*^ nanolithography,^2*h*^ polyoxometalate-containing materials,^2*i*^ single-chain nanoparticles,^2*j*^ gelators,^2*k*^ and optically active polymers.^2*l*^ Although [2 + 2] photodimerization reactions of coumarin derivatives are useful photochemical reactions, difficulties in controlling head-to-head (HH)/head-to-tail (HT), *syn*/*anti*, enantio- and diastereoselectivity of the reactions in solution have prevented further improvement of the usefulness and understanding of the reaction. To overcome these issues, several methodologies for *syn*/*anti* and HH/HT selective [2 + 2] photodimerization reactions of coumarins in solution have been developed, *e.g.*, Lewis acid catalysis,^3^ supramolecular photocatalysis accomplished by the use of cyclodextrins,^4*a*^ hydrazine derivatives,^4*b*^ bisurea macrocycles,^4*c*^ cucurbit[8]uril,^4*d*^ taco-type host–guest complexes^4*e*^ and self-assembled monolayers of coumarin derivatives on gold.^4*f*^ For an enantioselective reaction, Tanaka and Fujiwara reported outstanding results of an asymmetric photodimerization of simple coumarin by the use of TADDOL derivatives which gave an *anti*-HT dimer with excellent enantiomeric excess (up to 96% ee).^5^ However, to the best of our knowledge, the methodology to synthesis a *syn*-HT dimer in a diastereoselective manner has yet to be demonstrated.^6^ Herein, we report a novel asymmetric [2 + 2] photodimerization of chiral coumarin-3-carboxamide which gives only *syn*-HT dimers along with moderate diastereoselectivity.

We initially conducted [2 + 2] photodimerization reactions of (*S*)-4-phenyl-3-(2-oxo-2*H*-chromene-3-carbonyl)-2-oxazolidinone (1A) in acetone, resulting in recovery of 1A without any desired dimers, which could be caused by the incredibly poor solubility of 1A (Table 1). Next, we tried to use (*S*)-4-isopropyl-3-(2-oxo-2*H*-chromene-3-carbonyl)-2-oxazolidinone (1B), which gave 2BE and 2BF as an inseparable mixture with good yield and the ratio of 2BE to 2BF as 55 : 45. X-ray crystallographic analysis provided a structure of 50 : 50 diastereomeric mixture of 2BE and 2BF, which revealed that the reaction had progressed in a *syn*- and HT-selective manner (Fig. 1). We also used (*S*)-4-benzyl-3-(2-oxo-2*H*-chromene-3-carbonyl)-2-oxazolidinone (1C) (entry 3) instead of 1B.

**Table 1:** [2 + 2] Photodimerization reactions of chiral coumarin-3-carboxamides

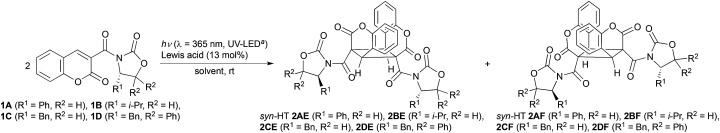						
Entry	Coumarin	Solvent	Additive	Time/h	Yield^b^/%	Diastereomeric ratio^c^
1	1A	Acetone	No	48	0	— (2AE) : — (2AF)
2	1B	Acetone	No	48	83	55 (2BE) : 45 (2BF)
3	1C	Acetone	No	48	99	63 (2CE) : 37 (2CF)
4	1C	Toluene	No	48	90	63 (2CE) : 7 (2CF)
5	1C	CF_3_C_6_H_5_	No	48	56	59 (2CE) : 41 (2CF)
6	1C	CH_2_Cl_2_	No	24	95	61 (2CE) : 39 (2CF)
7	1C	CHCl_3_	No	24	96	58 (2CE) : 42 (2CF)
8	1C	CH_3_CN	No	24	92	53 (2CE) : 47 (2CF)
9	1B	Toluene	Zn(ClO_4_)_2_·6H_2_O	96	24^d^	70 (2BE) : 30 (2BF)
10	1C	Toluene	Zn(ClO_4_)_2_·6H_2_O	48	92	75 (2CE) : 25 (2CF)
11	1C	Acetone	Zn(ClO_4_)_2_·6H_2_O	48	72	75 (2CE) : 25 (2CF)
12	1C	Toluene	BF_3_·OEt_2_	48	90	70 (2CE) : 30 (2CF)
13	1C	Toluene	Mg(ClO_4_)_2_	48	72	69 (2CE) : 31 (2CF)
14	1C	Toluene	Ni(ClO_4_)_2_·6H_2_O	48	75	67 (2CE) : 33 (2CF)
15	1C	Toluene	Co(ClO_4_)_2_·6H_2_O	48	51	70 (2CE) : 30 (2CF)
16	1C	Toluene	Cu(ClO_4_)_2_·6H_2_O	48	42	71 (2CE) : 29 (2CF)
17	1C	Toluene	Fe(ClO_4_)_2_·*x*H_2_O	48	77	72 (2CE) : 28 (2CF)
18	1D	Toluene	Zn(ClO_4_)_2_·6H_2_O	96	0	— (2DE) : — (2CF)

a The external irradiation was directed toward the Pyrex test tube with a working distance of 1 cm. All reactions were degassed by argon bubbling for 15 min prior to irradiation.

b Isolated yield.

c Determined by ^1^H NMR.

d Recovery of 1B in 52%.

**Fig. 1:**
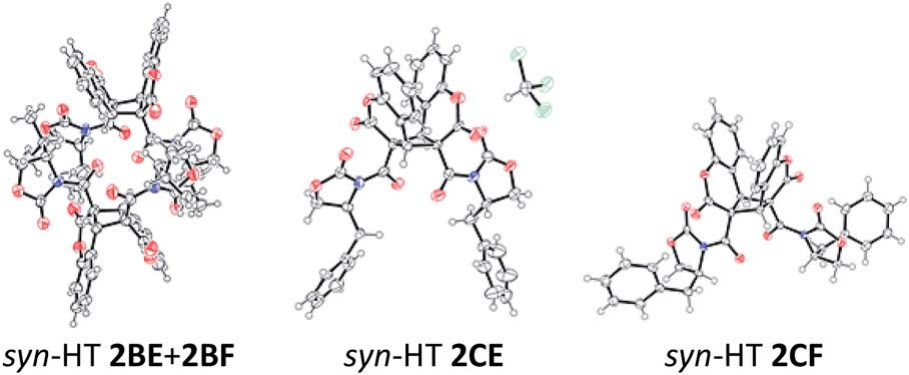
ORTEP drawing of photodimers: the inclusion of chloroform was observed in *syn*-HT 2CE.

As a result, solubility of the chiral coumarin and the diastereomeric ratio of dimers were improved to give 2CE and 2CF with the ratio of 63 : 37 in 99% yield. Photodimer 2CE and 2CF were separable. *syn*-HT structures of 2CE and 2CF were also successfully determined by X-ray crystallographic analyses. When the reaction was carried out in toluene (entry 4), *syn*-HT 2CE and *syn*-HT 2CF were obtained in excellent yield with the same diastereomeric ratio as the reaction in acetone (entry 3 *versus* entry 4). The yield of *syn*-HT 2CE and *syn*-HT 2CF was decreased by the use of α,α,α-trifluorotoluene (CF_3_C_6_H_5_) as a reaction solvent, which was probably due to low solubility of 1C in CF_3_C_6_H_5_ (entry 5). Solubility enhancement of 1C had a good impact on increasing the reaction rate by the use of CH_2_Cl_2_, CHCl_3_ and CH_3_CN as reaction solvents, but decreased the diastereoselectivity as compared to the reaction in toluene (entries 6–8 *versus* entries 3 and 4). As far as we know, there is only one example of a non-photochemical reaction, which is the highly diastereoselective 1,3-dipolar cycloaddition reaction, by the use of a chiral oxazolidinone-functionalized substrate in the absence of Lewis acid as reported by Sibi and co-workers.^7^ We were intrigued by induction of moderate diastereoselectivity in the absence of a chelating agent Lewis acid. Therefore, we tried to add Lewis acid in an effort to improve diastereoselectivity by utilizing a chelation control agent.^8^ Regarding a role for the Lewis acid in [2 + 2] photodimerization reactions, Lewis and co-workers reported the [2 + 2] photodimerization reaction of achiral coumarin in the presence of BF_3_·OEt_2_ to give the *syn*-HT dimer as a single product.^3^ In the search for a good chelating Lewis acid for 3-acyl-2-oxazolidinone derivatives, we tested Zn(ClO_4_)_2_·6H_2_O for the reaction of 1B (entry 9).^9^ A non-coordination solvent toluene was used for the reaction. As a result, the reaction of 1B was inhibited by the addition of Zn(ii) salt to give 2BE and 2BF with recovery of 1B (entry 9).

Interestingly, the reaction of 1C in the presence of Zn(ClO_4_)_2_·6H_2_O in toluene proceeded to achieve an improvement of yields and diastereomeric ratio of *syn*-HT dimers (2CE : 2CF = 75 : 25, entry 10). The use of acetone as a reaction solvent and Zn(ClO_4_)_2_·6H_2_O as a Lewis acid diminished the yield of *syn*-HT 2CE and *syn*-HT 2CF (entry 11). The reaction of 1C in the presence of BF_3_·OEt_2_ in toluene showed a slightly improved diastereomeric ratio in comparison with the reaction in the absence of BF_3_·OEt_2_ (2CE : 2CF = 70 : 30, entry 12). Other metal perchlorates such as Mg, Ni, Co, Cu and Fe slightly improved the 2CE : 2CF ratio (entries 13–17). To improve solubility of the chiral coumarin, we prepared 1D having three hydrophobic phenyl groups and used this compound for the photodimerization reaction. However, undesired photodecomposition of 1D occurred (entry 18). Interestingly, no reaction was induced when the reaction of 1C was performed in the presence of a stoichiometric amount of Zn(ClO_4_)_2_·6H_2_O as well as BF_3_·OEt_2_. This phenomenon might suggest that the ratio of the metal complex of 1C and metal-free 1C in solution is important for the reaction to progress.^3,10^

To gain insight into the mechanism of complete *syn*-HT selectivity and moderate diastereoselectivity, we optimized the structure of 1C in toluene using DFT calculations (Scheme 1).^11^ All calculations were performed using the B3LYP hybrid functional and a basis set (6-311G**) level of theory with Gaussian 16. The solvent effect of toluene was modeled *via* the polarizable continuum model using the integral equation formalism variant (IEFPCM). Three conformers 1CX, 1CY and 1CZ were generated which are energetically closed.^12^ We revealed that the order of the change in energy among three conformers was 1CX (S_0_) < 1CZ (S_0_) < 1CY (S_0_). The crystal structure of 1C was similar to 1CZ, which indicates that the major conformer involved in the [2 + 2] photodimerization would be changed between the solution and the crystalline states.^13^

**Scheme 1:**
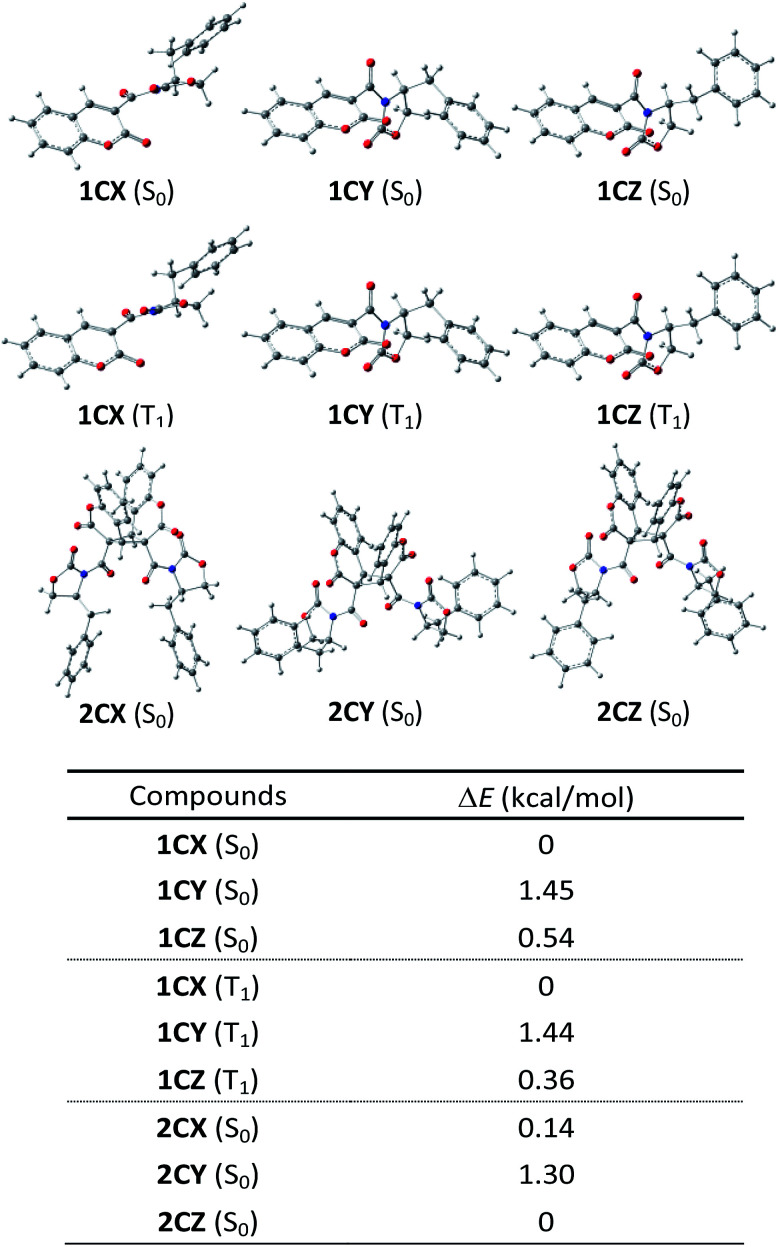
Optimized structures of 1C (S_0_ and T_1_) and 2C (S_0_).

Energies of photodimers *syn*-HH, *anti*-HH and *anti*-HT in toluene were also optimized by DFT calculation (B3LYP/6-311G**) to explain the exclusive formation of *syn*-HT dimer, which would be induced predominantly by accommodating the (*S*)-4-benzyl-2-oxazolidinone auxiliary in a favorable direction with a special allowance in the TS (Fig. 2).^14^ Structures of 2CX and 2CY were found to correspond to the structures of the cycloadducts *syn*-HT 2CE and *syn*-HT 2CF, respectively. It must be noted that 2CZ is the major diastereomer with a different conformation from that of 2CY. Thus, the order of the change in energy for photodimers was 2CZ < 2CX < 2CY. There was not much difference in energy between 2CX and 2CZ. Time-dependent DFT (TD-DFT) calculations showed that HOMO → LUMO transition for each of the three conformers 1CX, 1CY and 1CZ would be best described as a triplet excited state [^3^(π,π)*] (T_1_) for the carbon–carbon double bond of the reaction site with zero oscillator strength (Scheme 2). Therefore, it is suggested that the ground state (S_0_) for each of the three conformers was excited to become a singlet state [^1^(π,π)*] (S_1_) followed by intersystem crossing (ISC) to generate T_1_. Consequently, the order of the change in energy among T_1_ of the three conformers was also calculated as 1CX (T_1_) < 1CZ (T_1_) < 1CY (T_1_). From the results mentioned above, the [2 + 2] photodimerization reaction would start from excitation of the most stable comformer 1CX (S_0_) which preferentially generates 1CX (T_1_) *via* ISC of a singlet excited state 1CX (S_1_). The resulting triplet species 1CX (T_1_) would be subjected to react with 1CX (S_0_) in *syn*-HT fashion (discussed below) to give 2CX. The effect of the Lewis acid is unclear at the present time. However, we postulate that the Lewis acid could stabilize 1CX by a coordination in a bidentate fashion to prevent the conformational change to 1CY and 1CZ, which would be induced by a free rotation of the carbon–carbon single bond between the coumarin ring and the 2-oxazolidinone auxiliary.^3,8,15,16^ The moderate diastereoselectivity could be explained by the following process (also depicted in Scheme 2). Two carbonyl groups in the (*S*)-3-acyl-4-benzyl-2-oxazolidinone auxiliary could be oriented opposite each other to minimize the dipole moment and the electrostatic repulsion. To minimize steric repulsion caused between the benzyl group of the 2-oxazolidinone auxiliary and the carbonyl group of the coumarin ring, the conformational bias leads more favorably toward 1CX rather than 1CY and 1CZ. The carbonyl group including the 2-oxazolidinone ring would serve as a shielding group to prevent approach from the top side, thus affording 2CX, *i.e.*, *syn*-HT 2CE (Scheme 3).

**Fig. 2:**
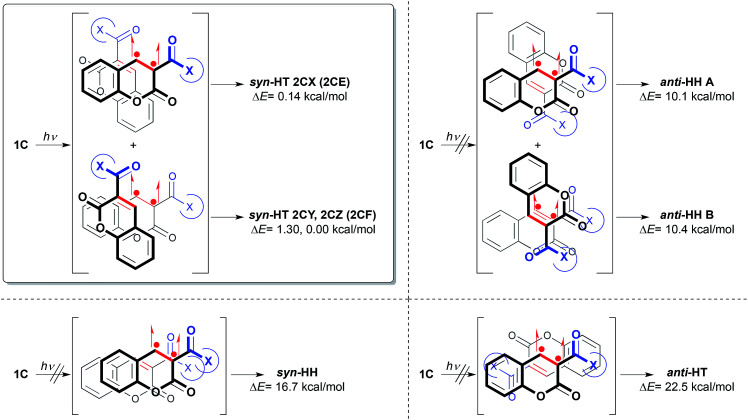
Exclusive formation of *syn*-HT dimers caused by the steric hindrance between two chiral coumarins.

**Scheme 2:**
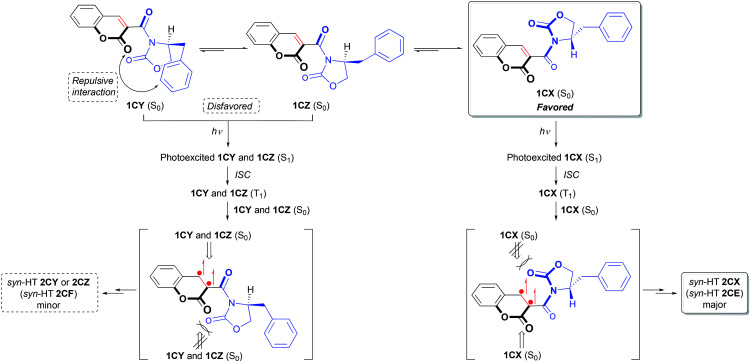
Proposed mechanism for diastereofacial selectivity.

**Scheme 3:**
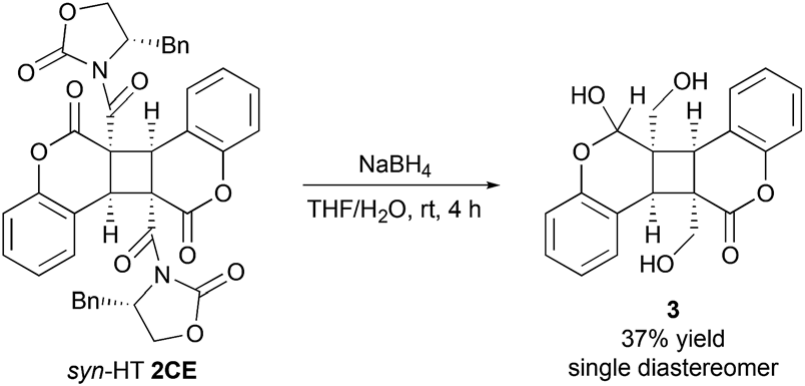
Removal of the chiral auxiliary with the reduction of chroman-2-one moiety.

The reduction of the chiral oxazolidinone moiety was successfully proceeded to give triol 3.^17^ Interestingly, the reduction of chroman-2-one moiety also concomitantly occured with the removal of chiral auxiliary affording 3 as a single diastereomer.^18^

In conclusion, we have developed a novel asymmetric photodimerization reaction of chiral coumarin-3-carboxamide which affords *syn*-HT dimer selectively with a moderate level of diastereoselectivity. Removal of chiral auxiliary was conducted to expand the applicability of the photodimer by the reduction using NaBH_4_ in THF–H_2_O. Further studies to elucidate the reaction mechanism and for application in the area of medicinal chemistry, drug delivery, and chemical biology are ongoing.

## Conflicts of interest

There are no conflicts to declare.

## Supplementary Material

RA-009-C9RA00822E-s001

RA-009-C9RA00822E-s002
