# Supplementary material for: Novel asymmetric photodimerization reaction of coumarin derivatives bearing a chiral 2-oxazolidinone auxiliary
Source: RSC Adv. 2019 Apr 23;9(22):12365–9. doi: 10.1039/c9ra00822e (PMC9063662; doi:10.1039/c9ra00822e)
Supplement: RA-009-C9RA00822E-s001 [file RA-009-C9RA00822E-s001.pdf]

Electronic Supplementary Material (ESI) for RSC Advances.
This journal is © The Royal Society of Chemistry 2019

Supporting Information

Novel asymmetric photodimerization reaction of coumarin derivatives bearing a chiral 2-oxazolidinone auxiliary

Kennosuke Itoh*[,a,b], Fumiya Odate[c], Takuma Karikomi[d], Keishi Obe[e], Tsutomu Miyamori[f], Hideaki Kamiya[f], Kenji Yoza[g], Kenichiro Nagai[b], Hideaki Fujii*[,a,b], Hiroyuki Suga*[,f], and Ken Tokunaga*[,h]

[a.] *Laboratory of Medicinal Chemistry, School of Pharmacy, Kitasato University, 5-9-1 Shirokane, Minato-ku, Tokyo 108-8641, Japan. E-mail: itok@pharm.kitasato-u.ac.jp*
[b.] *Medicinal Research Laboratories, School of Pharmacy, Kitasato University, 5-9-1 Shirokane, Minato-ku, Tokyo 108-8641, Japan.*
[c.] *Applied Chemistry and Chemical Engineering Program, Graduate School, Kogakuin University, 2665-1 Nakano, Hachioji, Tokyo 192-0015, Japan.*
[d.] *Department of Applied Chemistry, Faculty of Engineering, Kogakuin University, 2665-1 Nakano, Hachioji, Tokyo 192-0015, Japan.*
[e.] *Department of Applied Chemistry, School of Advanced Engineering, Kogakuin University, 2665-1 Nakano, Hachioji, Tokyo 192-0015, Japan.*
[f.] *Department of Materials Chemistry, Faculty of Engineering, Shinshu University, 4-17-1 Wakasato, Nagano 380-8553, Japan.*
[g.] *Bruker Japan, 3-9, Moriya-cho, Kanagawa-ku, Yokohama 221-0022, Japan.*
[h.] *Division of Liberal Arts, Center for Promotion of Higher Education, Kogakuin University, 2665-1 Nakano, Hachioji, Tokyo 192-0015, Japan.*

Table of Contents

General informations ········· S3

Photochemical reactor ········· S4

DFT and TD-DFT caluculations of **1C** ········· S5-S26

DFT caluculations of **1C**•$ZnCl_2$ complex ········· S27-S31

Synthesis of chiral coumarin-3-carboxamides ········· S32-S33

Typical procedure for photocycloadducts ········· S33-S34

Removal of chiral auxiliary and stereoselective reduction ········· S35

Limitation of chiral coumarin-3-carboxamides ········· S35-S36

^{1}H NMR and ^{13}C spectra of coumarin derivatives and photodimers ········· S37-S63

^{1}H NMR and ^{13}C spectra of **1C** and **1C**•BF_3•Et_2O ········· S64-S65

^{1}H NMR spectra of **1C** and **1C**•$Zn(ClO_4)_2$•$6H_2O$ ········· S66

Structural characterization of **1C** by the use of 1D and 2D NMR ········· S67-S83

UV-Vis spectra ········· S84-S86

Crystal structure report ········· S87-S115

References ········· S116

General informations

Proton NMR spectra were recorded on Bruker Biospin AV600 (600 MHz), AV500 (500 MHz), AV300 (300 MHz) NMR spectrometer and Agilent 400 MR (400 MHz) spectrometer. Chemical shifts are reported in parts per million downfield (δ) relative to internal tetramethylsilane (TMS, δ 0.00) or with the solvent reference as the internal standard ($CDCl_3$, δ 7.26). Data are reported as follows: chemical shift, multiplicity (s = singlet, d = doublet, t = triplet, q = quartet, quint = quintet, sept = septet, br = broad, m = multiplet), coupling constants (Hz), integration, and assignment. Carbon NMR spectra were recorded on Bruker Biospin AV500 (125.7 MHz), AV300 (75.4 MHz), Agilent 400 MR (100 MHz), Agilent 600 (150 MHz) NMR spectrometer with complete proton decoupling. Chemical shifts are reported in parts per million downfield (δ) relative to internal TMS or/with the respective solvent reference as the internal standard ($CDCl_3$, δ 77.0). Single crystal X-ray diffraction (SCXRD) experiments were performed on a Bruker APEX II CCD detector with graphite-monochromatized Mo Kα radiation and Rigaku R-AXIS RAPID diffractometer with Cu Kα radiation. Structure solution and refinement were performed by direct methods and the program SHELXL-97 respectively. Mass spectra were taken with Bruker Daltonics micrOTOF II (attached to an APCI-Direct Probe or ESI ion sources) and JEOL JMS-T100LP AccuTOF (attached to an ESI ion source). Specific rotations were recorded with JASCO P-1010 polarimeter. Infrared (IR) spectra were recorded on a JASCO FT/IR-5300 IR spectrophotometer, ν_{max} in cm^{-1}. UV-VIS spectra were recorded on a Hitachi double beam spectrophotometer U-2800. Melting points were recorded on Yanako MP-S3 and were not corrected. Flash chromatography was performed using Silica Gel (PSQ-60B) from Fuji Silysia Chemical, LTD. Solvents were purchased from commercial sources and used without purification. Coumarin-3-carboxylic acid was purchased from Sigma-Aldrich Co. LLC. Oxalyl chloride was purchased from Wako Pure Chemical Industries, Ltd. *n*-Butyl lithium in *n*-hexane was purchased from Kanto Chemical Co., Inc. (*S*)-4-Benzyl-1,3-oxazolidine-2-one was purchased from Tokyo Chemical Industry Co., Ltd. (*S*)-5,5-Diphenyl-4-benzyl-1,3-oxazolidine-2-one was prepared by the use of previously reported procedure.[1] 7-Methoxycoumarin-3-carboxylic acid, 5,7-dibromocoumarin-3-carboxylic acid, and 4-methylcoumarin-3-carboxylic acid were prepared by the use of previously reported procedure.[2]

Photochemical reactor

OMRON ZUV-H20MB controlled by OMRON ZUV-C20H was used as the UV-LED light source (Fig. S1). Irradiation power was measured by USHIO UIT-250 equipped with UV sensor (UVD-S365).[3] DOSHISHA, FST-106U WH was used as a cooling fan.

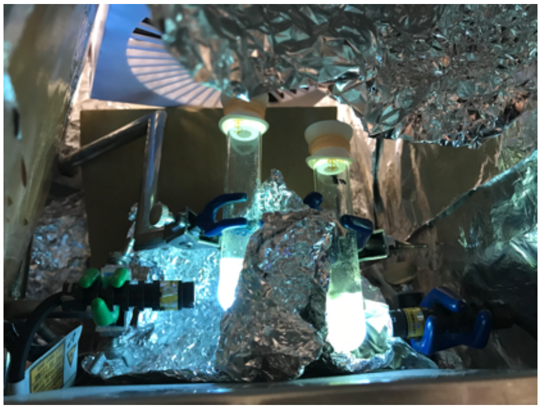

Fig. S1. Photochemical reactor

Computational study

Table S1. Electronic energies (E) of three conformers of monomer reactants (**1C**) in S_0 and T_1 states were optimized by density functional theory (DFT) or time-dependent density functional theory (TD-DFT) calculations in toluene. B3LYP and 6-311G(d,p) were used as the functional and basis set. ΔE represent relative values of E to the most stable conformer. Minimum values are shown with underline.

Compound	Conformation	State	Method	E (a.u.)	ΔE (kcal/mol)
Monomer	**1CX**	S_0	DFT	<u>**-1202.38302821**</u>	<u>**0.00**</u>
	**1CY**	S_0	DFT	-1202.38071775	1.45
	**1CZ**	S_0	DFT	-1202.38217539	0.54
	**1CX**	T_1	TD-DFT	<u>**-1202.29853752**</u>	<u>**0.00**</u>
	**1CY**	T_1	TD-DFT	-1202.29624259	1.44
	**1CZ**	T_1	TD-DFT	-1202.29795688	0.36

- **1CX** is most stable in the ground state (S_0) and the first excited state (T_1) as shown in ΔE value in Table S1.

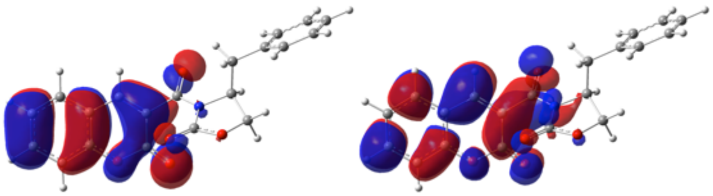

HOMO LUMO

Fig. S2. Frontier molecular orbital of **1CX**

- Time-dependent DFT (TD-DFT) calculations showed that the HOMO→LUMO transition for each of the three conformers would be the best described as a triplet excited state [$^3(\pi,\pi)^*$] (T_1) for the C-C double bond of the reaction site with zero oscillator strength.

Table S2. Electronic energies (*E*) of singlet dimer products (**2C**) in S_0 state were optimized by density functional theory (DFT) calculations in toluene. B3LYP and 6-311G(*d*,*p*) were used as the functional and basis sets. Minimum values are shown with underline.

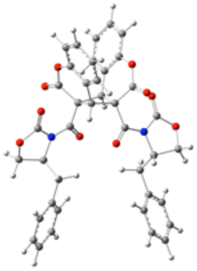

syn-**HT**
2CX (**2CE**)

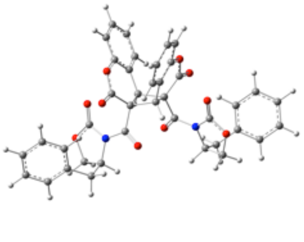

syn-**HT**
2CY (**2CF**)

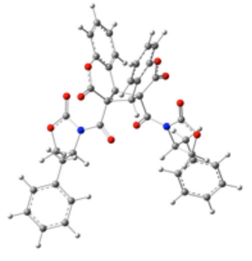

syn-**HT**
2CY (**2CF**)

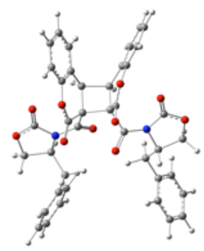

syn-**HH**

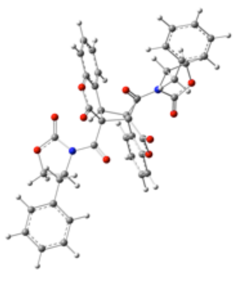

anti-**HH A**

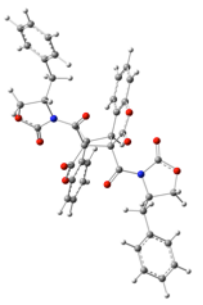

anti-**HH B**

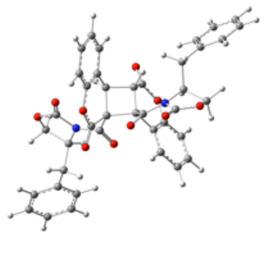

anti-**HT**

Compound	Conformation	*E* (a.u.)	Δ*E* (kcal/mol)
Dimer (**2C**)	*syn*-HT **2CX** (**2CE**)	-2404.74071878	0.14
	syn-HT **2CY** (**2CF**)	-2404.73887831	1.30
	syn-HT **2CZ** (**2CF**)	<u>**-2404.74094220**</u>	<u>**0.00**</u>
	syn-HH	-2404.71435487	16.68
	anti-HH **A**	-2404.72479750	10.13
	anti-HH **B**	-2404.72436810	10.40
	anti-HT	-2404.70503512	22.53

Table S3. Coordinates of the optimized geometry of **1CX** in S_0 state calculated by B3LYP/6-311G(*d*,*p*).

No.	Element	Coordinates (Å)		
		x	y	z
1	C	-3.955605	-1.023708	-0.257057
2	C	-4.475634	0.160616	0.290982
3	C	-2.283675	0.924447	0.983239
4	H	-4.448630	-2.857738	-1.269515
5	C	-4.844855	-1.942289	-0.844803
6	C	-5.839815	0.437427	0.256352
7	C	-6.695612	-0.484855	-0.330741
8	C	-6.202451	-1.676583	-0.880700
9	H	-6.202491	1.361741	0.687345
10	H	-7.758709	-0.277325	-0.361520
11	H	-6.884000	-2.385580	-1.334147
12	O	-3.658831	1.075385	0.889772
13	O	-1.672030	1.749431	1.610536
14	C	-1.725737	-0.267574	0.338168
15	C	-2.536466	-1.211552	-0.196969
16	H	-2.104399	-2.118879	-0.604877
17	O	0.180586	-1.596659	0.806503
18	C	-0.253791	-0.527815	0.416662
19	N	0.614010	0.488698	0.046349
20	C	0.303418	1.638602	-0.701268
21	O	-0.716946	1.888730	-1.276697
22	O	1.395155	2.431994	-0.717322
23	C	2.799135	-0.489429	-0.688372
24	H	2.312059	-1.466013	-0.665676
25	H	2.673601	-0.080928	-1.695742
26	C	4.269323	-0.636214	-0.363570
27	C	4.679256	-1.443207	0.705870
28	C	5.247762	0.032108	-1.106781
29	C	6.027912	-1.572094	1.026336
30	H	3.935643	-1.982572	1.284133
31	C	6.599756	-0.096196	-0.789434
32	H	4.951385	0.648721	-1.949410
33	C	6.993315	-0.896932	0.279760
34	H	6.326354	-2.204619	1.854912
35	H	7.343442	0.426264	-1.380718
36	H	8.043813	-0.999591	0.526865
37	C	2.400111	1.910413	0.188527
38	H	3.376730	2.099224	-0.250178
39	H	2.307056	2.437644	1.139362
40	C	2.058917	0.420024	0.317133
41	H	2.215021	0.056580	1.332388

Table S4. Coordinates of the optimized geometry of **1CY** in S_0 state calculated by B3LYP/6-311G(d,p).

No.	Element	Coordinates (Å)		
		x	y	z
1	C	-2.639333	-1.124919	-0.474999
2	C	-1.443701	-0.491069	-0.500346
3	H	-2.685052	-2.197908	-0.627183
4	C	-1.364070	0.966886	-0.398363
5	O	-2.550637	1.621398	-0.118709
6	O	-0.382317	1.640571	-0.578930
7	C	-3.753371	0.981956	-0.019621
8	C	-3.852667	-0.404897	-0.217290
9	C	-4.874212	1.756435	0.267031
10	H	-4.756136	2.822178	0.415072
11	C	-5.120112	-1.008752	-0.127809
12	H	-5.203465	-2.079035	-0.279444
13	C	-6.113310	1.135717	0.351549
14	H	-6.991097	1.731087	0.573321
15	C	-6.240608	-0.246071	0.152463
16	H	-7.214552	-0.714749	0.220457
17	C	-0.198803	-1.271480	-0.796844
18	O	-0.113042	-1.974173	-1.785951
19	C	2.113807	-1.924029	-0.158213
20	C	2.680970	-2.043674	1.269704
21	H	1.863058	-2.904586	-0.564040
22	H	3.717992	-1.723965	1.343660
23	H	2.571405	-3.050199	1.675608
24	N	0.857662	-1.203098	0.106155
25	O	1.890456	-1.149320	2.085692
26	C	0.781322	-0.750025	1.435383
27	O	-0.105671	-0.129921	1.950181
28	C	3.035116	-1.212846	-1.177332
29	H	3.809463	-1.936057	-1.451289
30	H	2.446130	-1.035641	-2.080569
31	C	3.695041	0.065640	-0.706020
32	C	5.052501	0.071182	-0.361637
33	C	2.976745	1.264229	-0.606940
34	C	5.677701	1.236472	0.078723
35	H	5.630245	-0.843919	-0.450540
36	C	3.600125	2.428821	-0.163354
37	H	1.926567	1.295224	-0.871364
38	C	4.950570	2.420026	0.181499
39	H	6.730939	1.219189	0.336336
40	H	3.025302	3.345176	-0.090176
41	H	5.432983	3.328857	0.523308

Table S5. Coordinates of the optimized geometry of **1CZ** in S_0 state calculated by B3LYP/6-311G(*d*,*p*).

No.	Element	Coordinates (Å)		
		x	y	z
1	C	4.061997	-0.781457	-0.348954
2	C	4.296651	0.083324	0.732924
3	C	1.929099	0.406793	1.121116
4	H	4.990228	-2.008398	-1.852933
5	C	5.166498	-1.340062	-1.017511
6	C	5.589922	0.396374	1.142881
7	C	6.662860	-0.166960	0.465053
8	C	6.455406	-1.037269	-0.614184
9	H	5.730478	1.068598	1.979586
10	H	7.672463	0.071109	0.778463
11	H	7.303178	-1.469187	-1.131461
12	O	3.260588	0.635516	1.430126
13	O	1.094141	0.858191	1.860534
14	C	1.680363	-0.417334	-0.064983
15	C	2.698041	-1.024002	-0.718696
16	H	2.486339	-1.687137	-1.550481
17	O	-0.142343	-1.858880	-0.543201
18	C	0.263670	-0.716468	-0.442242
19	N	-0.592765	0.353817	-0.675466
20	C	-0.224592	1.684073	-0.930069
21	O	0.871554	2.166852	-0.892056
22	O	-1.336089	2.373787	-1.262056
23	C	-2.678341	-0.059251	0.615895
24	H	-2.158842	-0.895630	1.089334
25	H	-2.493617	0.825365	1.231468
26	C	-4.160327	-0.352132	0.537947
27	C	-4.612090	-1.603404	0.098338
28	C	-5.109681	0.613568	0.887842
29	C	-5.973729	-1.877873	0.005022
30	H	-3.890249	-2.371677	-0.160802
31	C	-6.474518	0.341418	0.796552
32	H	-4.779166	1.582329	1.248679
33	C	-6.910223	-0.904401	0.352409
34	H	-6.304484	-2.853880	-0.332255
35	H	-7.194937	1.101459	1.077580
36	H	-7.970617	-1.118810	0.283327
37	C	-2.453373	1.475609	-1.461482
38	H	-3.336149	1.934138	-1.021868
39	H	-2.599126	1.355545	-2.536124
40	C	-2.046924	0.160190	-0.775786
41	H	-2.253209	-0.698678	-1.413455

Table S6. Coordinates of the optimized geometry of **1CX** in T_1 state calculated by B3LYP/6-311G(*d*,*p*).

No.	Element	Coordinates (Å)		
		x	y	z
1	C	-3.954936	-1.066191	-0.236292
2	C	-4.470782	0.174937	0.294837
3	C	-2.273852	0.905248	0.993010
4	H	-4.569555	-2.913480	-1.204869
5	C	-4.925630	-1.969913	-0.808522
6	C	-5.803397	0.495730	0.235057
7	C	-6.714914	-0.404193	-0.351038
8	C	-6.256218	-1.639188	-0.862039
9	H	-6.134375	1.438862	0.651575
10	H	-7.765268	-0.147933	-0.400646
11	H	-6.965030	-2.330408	-1.302863
12	O	-3.633781	1.073598	0.940829
13	O	-1.632122	1.713612	1.623828
14	C	-1.709915	-0.259871	0.320763
15	C	-2.603571	-1.321027	-0.181216
16	H	-2.172594	-2.246264	-0.534865
17	O	0.142716	-1.617998	0.703682
18	C	-0.257558	-0.513566	0.357684
19	N	0.624687	0.502872	0.042094
20	C	0.319761	1.655533	-0.700603
21	O	-0.704600	1.908864	-1.269843
22	O	1.413273	2.442956	-0.716172
23	C	2.806459	-0.490870	-0.686049
24	H	2.317824	-1.466762	-0.662906
25	H	2.682392	-0.084289	-1.694323
26	C	4.276107	-0.639220	-0.359025
27	C	4.683648	-1.443023	0.713636
28	C	5.256089	0.024725	-1.104099
29	C	6.031806	-1.572814	1.035811
30	H	3.939023	-1.979777	1.293048
31	C	6.607559	-0.104645	-0.785060
32	H	4.961552	0.638312	-1.949560
33	C	6.998828	-0.901902	0.287557
34	H	6.328548	-2.202905	1.866809
35	H	7.352609	0.414136	-1.377808
36	H	8.048929	-1.005381	0.535896
37	C	2.417827	1.913080	0.187222
38	H	3.394057	2.095699	-0.254542
39	H	2.330586	2.441011	1.138025
40	C	2.068340	0.423536	0.316722
41	H	2.220704	0.062624	1.333633

Table S7. Coordinates of the optimized geometry of **1CY** in T_1 state calculated by B3LYP/6-311G(*d*,*p*).

No.	Element	Coordinates (Å)		
		x	y	z
1	C	2.738767	-1.127789	0.678294
2	C	1.422790	-0.472629	0.514712
3	H	2.778267	-2.161883	0.988272
4	C	1.351714	0.973972	0.356111
5	O	2.519117	1.640088	0.090619
6	O	0.345876	1.632993	0.485627
7	C	3.740360	0.986714	-0.009218
8	C	3.864314	-0.408472	0.346074
9	C	4.821396	1.740936	-0.389440
10	H	4.670440	2.783224	-0.641195
11	C	5.198641	-0.959961	0.317459
12	H	5.324567	-2.000456	0.592748
13	C	6.103043	1.157713	-0.429985
14	H	6.954085	1.753289	-0.734026
15	C	6.273381	-0.195467	-0.062426
16	H	7.264361	-0.633669	-0.084151
17	C	0.201475	-1.270862	0.734686
18	O	0.147728	-2.018078	1.701698
19	C	-2.113575	-1.907401	0.087589
20	C	-2.689268	-1.976548	-1.343091
21	H	-1.856404	-2.902765	0.453896
22	H	-3.698616	-1.576811	-1.411357
23	H	-2.658514	-2.985260	-1.755245
24	N	-0.860381	-1.174079	-0.153005
25	O	-1.835452	-1.142038	-2.161227
26	C	-0.735756	-0.761736	-1.490868
27	O	0.191403	-0.188609	-1.992450
28	C	-3.033848	-1.243536	1.138764
29	H	-3.794859	-1.986204	1.396996
30	H	-2.438478	-1.088848	2.042175
31	C	-3.717128	0.040172	0.717818
32	C	-5.083316	0.040920	0.409975
33	C	-3.012909	1.247725	0.629621
34	C	-5.730900	1.210998	0.017244
35	H	-5.649430	-0.882259	0.489306
36	C	-3.658498	2.417348	0.233602
37	H	-1.956539	1.281185	0.866018
38	C	-5.017803	2.404007	-0.074267
39	H	-6.790471	1.189900	-0.212379
40	H	-3.094677	3.341139	0.168895
41	H	-5.517727	3.316498	-0.378996

Table S8. Coordinates of the optimized geometry of **1CZ** in T_1 state calculated by B3LYP/6-311G(*d*,*p*).

No.	Element	Coordinates (Å)		
		x	y	z
1	C	4.050336	-0.874773	-0.290201
2	C	4.277330	0.128828	0.724888
3	C	1.908010	0.455889	1.075954
4	H	5.088477	-2.239624	-1.626462
5	C	5.228198	-1.474366	-0.872125
6	C	5.539982	0.518700	1.094510
7	C	6.662812	-0.073117	0.484260
8	C	6.486045	-1.077491	-0.493766
9	H	5.650979	1.273071	1.863369
10	H	7.657840	0.238741	0.774240
11	H	7.355159	-1.536294	-0.950590
12	O	3.215002	0.695557	1.414774
13	O	1.047466	0.940197	1.773103
14	C	1.657260	-0.409053	-0.072921
15	C	2.762163	-1.198525	-0.651393
16	H	2.537583	-1.964544	-1.379417
17	O	-0.086817	-1.846417	-0.642773
18	C	0.275163	-0.685982	-0.497322
19	N	-0.603151	0.367661	-0.697369
20	C	-0.231342	1.684846	-0.996719
21	O	0.875522	2.148040	-1.008994
22	O	-1.342097	2.379868	-1.307260
23	C	-2.666055	-0.088753	0.614043
24	H	-2.142910	-0.940739	1.055308
25	H	-2.466608	0.777235	1.250961
26	C	-4.150645	-0.373079	0.552805
27	C	-4.616623	-1.606951	0.080205
28	C	-5.088264	0.584728	0.952449
29	C	-5.981151	-1.871932	0.003110
30	H	-3.904157	-2.370022	-0.217506
31	C	-6.455871	0.321879	0.877572
32	H	-4.746308	1.539273	1.339500
33	C	-6.905985	-0.906368	0.400162
34	H	-6.323092	-2.834551	-0.360348
35	H	-7.167073	1.075241	1.197283
36	H	-7.968530	-1.113573	0.343717
37	C	-2.482981	1.496347	-1.434030
38	H	-3.327225	1.965930	-0.934367
39	H	-2.701717	1.385953	-2.496686
40	C	-2.056061	0.167579	-0.780329
41	H	-2.266872	-0.678120	-1.435134

Table S9. Coordinates of the optimized geometry of **2CX** in S_0 state calculated by B3LYP/6-311G(*d*,*p*).

No.	Element	Coordinates (Å)		
		x	y	z
1	O	0.605631	2.058731	-3.778577
2	O	-0.597843	3.259697	-2.374308
3	O	2.474298	2.614465	-1.527471
4	O	-0.911184	1.385172	0.619887
5	O	2.744298	3.902460	0.294811
6	O	-2.744298	-3.902460	0.294811
7	O	-2.474298	-2.614465	-1.527471
8	O	-0.605631	-2.058731	-3.778577
9	O	0.911184	-1.385172	0.619887
10	O	0.597843	-3.259697	-2.374308
11	N	1.008381	2.502518	0.286963
12	C	2.614469	2.915218	4.784774
13	H	3.580596	2.858512	4.293373
14	C	2.112144	2.970728	-0.439357
15	N	-1.008381	-2.502518	0.286963
16	C	1.467314	2.500569	4.100384
17	C	0.012105	2.242537	-2.580486
18	C	-0.044932	-1.103060	-1.580930
19	C	-2.737931	0.943565	-3.472533
20	H	-2.982428	1.728428	-2.766190
21	C	-0.012105	1.666175	-0.146896
22	C	-3.364122	0.908742	-4.713013
23	H	-4.098366	1.663293	-4.967846
24	C	-3.044754	-0.099442	-5.622258
25	H	-3.527106	-0.135836	-6.591833
26	C	-1.780188	-0.013223	-3.116249
27	C	-1.104308	0.027395	-1.774951
28	H	-1.842997	0.098467	-0.979973
29	C	-0.012105	-2.242537	-2.580486
30	C	2.535041	3.384200	6.095773
31	H	3.436224	3.696883	6.611215
32	C	1.016554	3.045435	1.658703
33	H	0.007427	3.354190	1.926463
34	C	3.364122	-0.908742	-4.713013
35	H	4.098366	-1.663293	-4.967846
36	C	-1.016554	-3.045435	1.658703
37	H	-0.007427	-3.354190	1.926463
38	C	2.737931	-0.943565	-3.472533
39	H	2.982428	-1.728428	-2.766190
40	C	0.012105	-1.666175	-0.146896
41	C	1.780188	0.013223	-3.116249
42	C	0.044932	1.103060	-1.580930

43	C	1.496286	1.020143	-4.035622
44	C	-2.112144	-2.970728	-0.439357
45	C	1.943390	4.250593	1.454021
46	H	1.393410	5.163169	1.219835
47	H	2.619334	4.426912	2.286910
48	C	1.104308	-0.027395	-1.774951
49	H	1.842997	-0.098467	-0.979973
50	C	-1.467314	-2.500569	4.100384
51	C	1.542814	2.006932	2.672289
52	H	0.949784	1.097600	2.556932
53	H	2.575397	1.753310	2.414579
54	C	3.044754	0.099442	-5.622258
55	H	3.527106	0.135836	-6.591833
56	C	-1.496286	-1.020143	-4.035622
57	C	-2.111548	-1.070766	-5.281979
58	H	-1.853837	-1.876998	-5.956980
59	C	-0.152330	-3.029950	6.072373
60	H	0.809354	-3.064853	6.571813
61	C	0.235104	2.561472	4.764023
62	H	-0.663964	2.228949	4.254878
63	C	2.111548	1.070766	-5.281979
64	H	1.853837	1.876998	-5.956980
65	C	-1.943390	-4.250593	1.454021
66	H	-1.393410	-5.163169	1.219835
67	H	-2.619334	-4.426912	2.286910
68	C	-1.303108	-3.444974	6.742158
69	H	-1.239474	-3.806876	7.761994
70	C	-1.542814	-2.006932	2.672289
71	H	-2.575397	-1.753310	2.414579
72	H	-0.949784	-1.097600	2.556932
73	C	-0.235104	-2.561472	4.764023
74	H	0.663964	-2.228949	4.254878
75	C	1.303108	3.444974	6.742158
76	H	1.239474	3.806876	7.761994
77	C	-2.535041	-3.384200	6.095773
78	H	-3.436224	-3.696883	6.611215
79	C	0.152330	3.029950	6.072373
80	H	-0.809354	3.064853	6.571813
81	C	-2.614469	-2.915218	4.784774
82	H	-3.580596	-2.858512	4.293373

Table S10. Coordinates of the optimized geometry of **2CY** in S_0 state calculated by B3LYP/6-311G(*d*,*p*).

No.	Element	Coordinates (Å)		
		x	y	z
1	C	0.227176	0.714723	1.081006
2	C	1.082470	0.514948	-0.209542
3	C	-0.227176	0.714724	-1.081007
4	H	0.321685	-0.077236	1.820291
5	H	-0.321685	-0.077234	-1.820293
6	C	-2.188711	1.506989	0.506745
7	O	-3.023726	1.303582	1.351090
8	C	-1.354372	2.966140	-1.221060
9	O	-2.160377	2.701389	-0.117213
10	C	-0.427577	2.057181	-1.725950
11	C	0.283310	2.421911	-2.875597
12	H	0.999790	1.723691	-3.292452
13	C	0.093412	3.660540	-3.476997
14	H	0.658878	3.921991	-4.363149
15	C	-0.826749	4.559156	-2.937498
16	H	-0.982802	5.526961	-3.399060
17	C	-1.557406	4.210362	-1.808601
18	H	-2.292187	4.876716	-1.374876
19	C	-1.082470	0.514949	0.209541
20	C	-1.609209	-0.922770	0.400405
21	O	-1.164006	-1.660260	1.257189
22	N	-2.586137	-1.400771	-0.461773
23	C	-3.189739	-0.724211	-1.532019
24	O	-3.897679	-1.604752	-2.254551
25	O	-3.106836	0.445531	-1.790857
26	C	-3.666653	-2.955008	-1.784160
27	H	-4.626625	-3.465274	-1.776350
28	H	-2.988474	-3.441345	-2.486600
29	C	-4.003204	-3.079951	0.796591
30	H	-4.147857	-4.164512	0.824756
31	H	-3.474643	-2.811369	1.713476
32	C	-5.351011	-2.393860	0.736301
33	C	-6.485770	-3.094399	0.308683
34	C	-5.497619	-1.051743	1.109918
35	C	-7.730155	-2.470970	0.238593
36	H	-6.398698	-4.143245	0.041585
37	C	-6.740391	-0.426122	1.036709
38	H	-4.642280	-0.487477	1.462939
39	C	-7.859686	-1.131615	0.598903
40	H	-8.596732	-3.032680	-0.092083
41	H	-6.830443	0.614829	1.325650
42	H	-8.826128	-0.643331	0.545214

43	C	-3.044770	-2.800674	-0.385048
44	H	-2.169105	-3.439861	-0.271735
45	C	2.188712	1.506989	-0.506745
46	O	2.160379	2.701388	0.117214
47	O	3.023726	1.303582	-1.351090
48	C	1.354374	2.966138	1.221062
49	C	0.427578	2.057178	1.725951
50	C	1.557409	4.210359	1.808605
51	H	2.292190	4.876713	1.374880
52	C	-0.283308	2.421908	2.875598
53	H	-0.999789	1.723688	3.292452
54	C	0.826753	4.559152	2.937503
55	H	0.982806	5.526956	3.399066
56	C	-0.093409	3.660536	3.477000
57	H	-0.658875	3.921986	4.363152
58	C	1.609209	-0.922771	-0.400408
59	O	1.164007	-1.660259	-1.257193
60	C	3.044769	-2.800676	0.385044
61	C	3.666649	-2.955012	1.784157
62	H	2.169103	-3.439862	0.271729
63	H	4.626620	-3.465279	1.776348
64	H	2.988469	-3.441349	2.486596
65	N	2.586136	-1.400773	0.461770
66	O	3.897676	-1.604756	2.254549
67	C	3.189737	-0.724214	1.532018
68	O	3.106836	0.445528	1.790857
69	C	4.003204	-3.079952	-0.796594
70	H	4.147857	-4.164513	-0.824760
71	H	3.474644	-2.811369	-1.713479
72	C	5.351011	-2.393861	-0.736302
73	C	6.485769	-3.094399	-0.308681
74	C	5.497619	-1.051744	-1.109919
75	C	7.730153	-2.470970	-0.238588
76	H	6.398696	-4.143244	-0.041582
77	C	6.740391	-0.426123	-1.036708
78	H	4.642281	-0.487479	-1.462942
79	C	7.859685	-1.131615	-0.598898
80	H	8.596730	-3.032679	0.092091
81	H	6.830443	0.614829	-1.325649
82	H	8.826126	-0.643331	-0.545208

Table S11. Coordinates of the optimized geometry of **2CZ** in S_0 state calculated by B3LYP/6-311G(*d*,*p*).

No.	Element	Coordinates (Å)		
		x	y	z
1	C	-0.106418	-1.545332	1.095718
2	C	-1.071183	-1.356540	-0.116416
3	C	0.161912	-1.540824	-1.095111
4	H	-0.153073	-0.759880	1.846353
5	H	0.182311	-0.754308	-1.845830
6	C	2.275827	-2.281014	0.312706
7	O	3.188994	-2.043710	1.060164
8	C	1.313403	-3.774185	-1.317444
9	O	2.214357	-3.483366	-0.297191
10	C	0.327306	-2.886869	-1.742840
11	C	-0.482072	-3.277349	-2.816391
12	H	-1.247339	-2.596320	-3.170473
13	C	-0.327640	-4.519572	-3.420490
14	H	-0.969442	-4.801066	-4.246462
15	C	0.655026	-5.396099	-2.960720
16	H	0.784039	-6.366727	-3.424733
17	C	1.481990	-5.021785	-1.909125
18	H	2.265397	-5.671033	-1.539184
19	C	1.119668	-1.319444	0.117016
20	C	1.636261	0.124624	0.270407
21	O	1.257602	0.852751	1.165759
22	N	2.543796	0.613828	-0.659468
23	C	3.095656	-0.051769	-1.762447
24	O	3.839381	0.822817	-2.461993
25	O	2.956641	-1.205683	-2.064313
26	C	3.638266	2.167350	-1.955063
27	H	4.592133	2.686967	-1.997328
28	H	2.914216	2.664891	-2.602108
29	C	4.197826	2.023861	0.565700
30	H	3.760592	1.640963	1.489882
31	H	5.011978	1.348304	0.287954
32	C	4.726277	3.424509	0.784208
33	C	3.952050	4.373809	1.464018
34	C	5.984675	3.807630	0.308973
35	C	4.420220	5.670720	1.656248
36	H	2.980529	4.089371	1.855975
37	C	6.457120	5.105724	0.501060
38	H	6.607466	3.081095	-0.203141
39	C	5.674907	6.041544	1.172966
40	H	3.809358	6.390461	2.189626
41	H	7.437746	5.381880	0.130028
42	H	6.041603	7.050144	1.325773

43	C	3.109728	1.969631	-0.528214
44	H	2.309119	2.670170	-0.296197
45	C	-2.194118	-2.356785	-0.311938
46	O	-2.092144	-3.556236	0.298236
47	O	-3.114731	-2.150614	-1.059520
48	C	-1.181801	-3.816319	1.318469
49	C	-0.226242	-2.896105	1.743649
50	C	-1.307980	-5.068867	1.910243
51	H	-2.068918	-5.744355	1.540400
52	C	0.596001	-3.258840	2.817147
53	H	1.337763	-2.552232	3.171110
54	C	-0.468684	-5.414865	2.961777
55	H	-0.564668	-6.389273	3.425875
56	C	0.483792	-4.505526	3.421350
57	H	1.134843	-4.765043	4.247278
58	C	-1.636556	0.069213	-0.269821
59	O	-1.281857	0.810205	-1.164407
60	C	-3.175589	1.861371	0.525939
61	C	-3.714592	2.040331	1.951398
62	H	-2.399946	2.590106	0.295962
63	H	-4.687008	2.524551	1.990960
64	H	-3.010948	2.564574	2.599872
65	N	-2.561860	0.526675	0.658816
66	O	-3.867339	0.689711	2.459029
67	C	-3.091078	-0.157655	1.761439
68	O	-2.910589	-1.305518	2.064406
69	C	-4.261815	1.877488	-0.571042
70	H	-3.807666	1.514285	-1.495034
71	H	-5.050950	1.170999	-0.298153
72	C	-4.841876	3.258008	-0.786487
73	C	-4.099199	4.240214	-1.454635
74	C	-6.117785	3.589764	-0.319428
75	C	-4.615253	5.519296	-1.643651
76	H	-3.114292	3.995451	-1.839907
77	C	-6.638208	4.869837	-0.508427
78	H	-6.716023	2.836911	0.183920
79	C	-5.887156	5.838833	-1.168781
80	H	-4.028190	6.265059	-2.167937
81	H	-7.631635	5.106098	-0.143977
82	H	-6.291071	6.833498	-1.319071

Table S12. Coordinates of the optimized geometry of *syn*-HH in S_0 state calculated by B3LYP/6-311G(*d*,*p*).

No.	Element	Coordinates (Å)		
		x	y	z
1	C	-6.121497	-2.638139	0.149339
2	C	-5.090180	-2.635631	1.079304
3	C	-3.854154	-2.099187	0.730500
4	C	-3.622327	-1.548760	-0.528497
5	C	-4.671911	-1.577315	-1.454235
6	C	-5.911640	-2.112158	-1.125675
7	H	-7.084100	-3.057224	0.417619
8	H	-5.214562	-3.049328	2.071932
9	C	-2.280454	-0.981404	-0.891957
10	H	-4.507764	-1.168798	-2.444757
11	H	-6.709017	-2.119552	-1.858987
12	C	-1.256649	-1.018745	0.292801
13	C	-1.579619	-1.853219	1.524536
14	O	-2.886126	-2.166881	1.726262
15	O	-0.769227	-2.159698	2.350909
16	C	0.120969	-1.320642	-0.320105
17	C	2.013475	-2.923544	-0.646193
18	C	2.016294	-4.451514	-0.458170
19	C	-0.166788	-3.780350	-0.270399
20	N	0.620368	-2.620469	-0.255772
21	O	-1.363031	-3.844547	-0.175842
22	O	0.624709	-4.844621	-0.470019
23	C	3.061204	-2.203064	0.217912
24	C	4.472499	-2.622238	-0.139314
25	C	5.067087	-2.178145	-1.327488
26	C	5.209015	-3.464426	0.700044
27	C	6.357596	-2.572496	-1.670495
28	H	4.519958	-1.507632	-1.982718
29	C	6.502370	-3.859659	0.360289
30	H	4.772596	-3.803291	1.633961
31	C	7.079164	-3.417162	-0.827526
32	H	6.803411	-2.214396	-2.591718
33	H	7.059104	-4.508633	1.026923
34	H	8.085500	-3.721340	-1.091811
35	H	2.851877	-2.417807	1.268902
36	H	2.945225	-1.128294	0.075744
37	H	2.153294	-2.657907	-1.697633
38	H	2.522739	-4.985906	-1.257810
39	H	2.437851	-4.741906	0.505278
40	O	0.750924	-0.470466	-0.917007
41	C	-5.801455	2.748710	-1.350931
42	C	-5.376326	2.290432	-0.106616

43	C	-4.163440	1.624184	-0.017817
44	C	-3.361753	1.394714	-1.128982
45	C	-3.794169	1.876936	-2.364290
46	C	-5.008703	2.549828	-2.480341
47	H	-6.746528	3.272430	-1.433034
48	H	-5.958683	2.451238	0.791611
49	C	-2.117950	0.593910	-0.951765
50	H	-3.173170	1.719596	-3.240196
51	H	-5.333302	2.919029	-3.445939
52	C	-1.482468	0.559908	0.476305
53	C	-2.505451	0.876404	1.594960
54	O	-3.775606	1.211372	1.252317
55	O	-2.221717	0.797007	2.756209
56	C	-0.171723	1.282046	0.872080
57	C	1.438642	3.162121	0.531174
58	C	1.163078	4.581505	0.011034
59	C	-0.874135	3.587858	0.338463
60	N	0.089169	2.571877	0.380909
61	O	-2.060753	3.464716	0.469104
62	O	-0.260678	4.772837	0.171574
63	C	2.530358	2.420416	-0.252649
64	C	3.884469	3.082418	-0.098598
65	C	4.594565	2.972351	1.104047
66	C	4.451675	3.820972	-1.142117
67	C	5.832112	3.590742	1.260544
68	H	4.179730	2.388382	1.919472
69	C	5.691844	4.440063	-0.989074
70	H	3.924939	3.903459	-2.087499
71	C	6.384265	4.328865	0.214028
72	H	6.368904	3.490790	2.197232
73	H	6.116899	5.004859	-1.811284
74	H	7.349319	4.807678	0.334449
75	H	2.242949	2.381901	-1.306695
76	H	2.572561	1.393366	0.110040
77	H	1.696610	3.179984	1.593725
78	H	1.672960	5.357998	0.575435
79	H	1.400013	4.679900	-1.050441
80	O	0.639222	0.732048	1.575631
81	H	-1.918890	-1.469149	-1.797733
82	H	-1.366266	0.847065	-1.697869

Table S13. Coordinates of the optimized geometry of *anti*-HH A in S_0 state calculated by B3LYP/6-311G(*d*,*p*).

No.	Element	Coordinates (Å)		
		x	y	z
1	C	-2.142161	-3.197946	3.922756
2	C	-1.646215	-1.901474	3.887409
3	C	-0.837965	-1.502535	2.824969
4	C	-0.516798	-2.371171	1.784592
5	C	-1.029191	-3.672680	1.840877
6	C	-1.832299	-4.090641	2.895596
7	H	-2.767033	-3.511242	4.750761
8	H	-1.863477	-1.185588	4.670089
9	C	0.360853	-1.931380	0.643041
10	H	-0.786239	-4.369364	1.045331
11	H	-2.211100	-5.105406	2.919113
12	C	0.543534	-0.381199	0.587636
13	C	0.276471	0.411984	1.882961
14	O	-0.368031	-0.203901	2.899298
15	O	0.705688	1.520821	2.045678
16	C	1.920248	0.115499	0.102081
17	C	4.382069	0.298183	0.394173
18	C	5.296824	-0.662266	1.162227
19	C	3.185177	-0.950451	2.008210
20	N	3.067401	-0.235035	0.808362
21	O	2.299734	-1.364140	2.704984
22	O	4.492546	-1.110095	2.283732
23	C	4.546775	1.781171	0.791644
24	C	5.840002	2.370759	0.273142
25	C	5.975367	2.689380	-1.084461
26	C	6.925982	2.601374	1.123799
27	C	7.165853	3.216157	-1.577683
28	H	5.136223	2.535586	-1.755523
29	C	8.119529	3.130271	0.632816
30	H	6.833021	2.380543	2.182455
31	C	8.243498	3.436353	-0.719993
32	H	7.250057	3.462587	-2.630225
33	H	8.948241	3.307728	1.309145
34	H	9.169197	3.850568	-1.103024
35	H	4.500637	1.863690	1.881356
36	H	3.696048	2.332870	0.388230
37	H	4.491008	0.184496	-0.683031
38	H	5.570622	-1.538977	0.572650
39	H	6.190681	-0.187854	1.559053
40	O	2.035222	0.814832	-0.880273
41	C	-1.923641	0.113292	-0.084828
42	C	-4.384425	0.315081	-0.376709

43	C	-5.296360	-0.598012	-1.203779
44	C	-3.180846	-0.839512	-2.054709
45	N	-3.068160	-0.194830	-0.814864
46	O	-2.292472	-1.213515	-2.770019
47	O	-4.486916	-0.981839	-2.345064
48	C	-4.545082	1.819090	-0.687982
49	C	-5.844147	2.378277	-0.150722
50	C	-5.994414	2.620177	1.221038
51	C	-6.920949	2.656586	-0.998857
52	C	-7.190367	3.118824	1.730073
53	H	-5.162569	2.428803	1.891516
54	C	-8.119910	3.157465	-0.492003
55	H	-6.816370	2.495674	-2.067196
56	C	-8.258630	3.387295	0.874422
57	H	-7.286087	3.305843	2.793803
58	H	-8.941216	3.372894	-1.166331
59	H	-9.188521	3.779759	1.269992
60	H	-4.486583	1.965796	-1.770300
61	H	-3.699085	2.345799	-0.243299
62	H	-4.499103	0.138969	0.691481
63	H	-5.574401	-1.506728	-0.667032
64	H	-6.187484	-0.100053	-1.577192
65	H	1.288551	-2.502016	0.643908
66	O	-2.042522	0.750172	0.938621
67	C	-0.544628	-0.349088	-0.597479
68	C	-0.279883	0.518062	-1.844907
69	C	-0.355921	-1.892758	-0.742022
70	C	0.523969	-2.262715	-1.906274
71	H	-1.281294	-2.465915	-0.777039
72	C	0.843565	-1.334328	-2.894141
73	C	1.040631	-3.557156	-2.036764
74	C	1.654631	-1.668664	-3.976555
75	C	1.846642	-3.910924	-3.112526
76	H	0.798532	-4.299145	-1.283014
77	C	2.154988	-2.959323	-4.085872
78	H	1.870622	-0.908106	-4.716257
79	H	2.228839	-4.921417	-3.194029
80	H	2.782149	-3.222248	-4.929522
81	O	0.369303	-0.035181	-2.893923
82	O	-0.714454	1.632242	-1.944477

Table S14. Coordinates of the optimized geometry of *anti*-HH B in S_0 state calculated by B3LYP/6-311G(d,p).

No.	Element	Coordinates (Å)		
		x	y	z
1	C	1.875136	-0.442255	-0.708148
2	C	4.236126	-1.175586	-0.927145
3	C	4.747309	-2.622321	-0.942512
4	C	2.727974	-2.660715	0.144176
5	N	2.856208	-1.395688	-0.448003
6	O	1.774723	-3.103474	0.723559
7	O	3.882063	-3.330379	-0.018691
8	C	5.018634	-0.231545	0.009715
9	C	6.397602	0.094028	-0.521498
10	C	6.548093	0.983197	-1.593633
11	C	7.544062	-0.487397	0.029280
12	C	7.809626	1.276791	-2.103806
13	H	5.670759	1.455407	-2.024201
14	C	8.809367	-0.194240	-0.478942
15	H	7.449016	-1.165661	0.871309
16	C	8.945068	0.686991	-1.548542
17	H	7.907177	1.970398	-2.931407
18	H	9.686891	-0.651530	-0.035704
19	H	9.927706	0.917661	-1.943941
20	H	5.092811	-0.692959	0.999126
21	H	4.438049	0.686843	0.119186
22	H	4.198066	-0.754739	-1.930348
23	H	4.643663	-3.089107	-1.922853
24	H	5.770130	-2.725539	-0.588721
25	O	2.189495	0.557495	-1.316211
26	C	0.426198	-0.682892	-0.235373
27	C	-0.075087	-1.885131	-1.059648
28	C	0.235835	-0.743464	1.314111
29	C	-0.818789	-1.731392	1.734690
30	C	-1.313626	-2.681641	0.845359
31	C	-1.326429	-1.731968	3.038913
32	C	-2.286068	-3.603462	1.226332
33	C	-2.293287	-2.647054	3.439395
34	H	-0.949503	-1.004719	3.750769
35	C	-2.775120	-3.586030	2.526230
36	H	-2.634916	-4.321743	0.495102
37	H	-2.666554	-2.630940	4.456352
38	H	-3.528966	-4.304515	2.825571
39	O	0.305307	-2.083291	-2.181012
40	O	-0.867039	-2.801132	-0.459112
41	H	1.153501	-0.918241	1.874053
42	C	-0.426198	0.682894	-0.235374

43	C	0.075086	1.885132	-1.059651
44	C	-0.235835	0.743468	1.314110
45	C	0.818788	1.731397	1.734687
46	C	1.313625	2.681645	0.845355
47	C	1.326429	1.731975	3.038910
48	C	2.286067	3.603467	1.226327
49	C	2.293287	2.647063	3.439391
50	H	0.949503	1.004727	3.750768
51	C	2.775119	3.586038	2.526224
52	H	2.634914	4.321748	0.495096
53	H	2.666554	2.630949	4.456348
54	H	3.528965	4.304523	2.825564
55	O	-0.305307	2.083290	-2.181015
56	O	0.867038	2.801134	-0.459115
57	H	-1.153502	0.918247	1.874052
58	C	-1.875135	0.442257	-0.708149
59	O	-2.189494	-0.557493	-1.316212
60	N	-2.856209	1.395689	-0.448004
61	C	-4.236127	1.175585	-0.927147
62	C	-2.727976	2.660716	0.144174
63	C	-4.747312	2.622319	-0.942513
64	O	-1.774725	3.103476	0.723555
65	O	-3.882066	3.330378	-0.018692
66	H	-5.770132	2.725535	-0.588720
67	H	-4.643668	3.089106	-1.922853
68	C	-5.018633	0.231542	0.009712
69	H	-4.438047	-0.686846	0.119182
70	H	-5.092811	0.692954	0.999123
71	H	-4.198066	0.754739	-1.930350
72	C	-6.397601	-0.094034	-0.521501
73	C	-7.544062	0.487386	0.029279
74	C	-6.548089	-0.983199	-1.593640
75	C	-8.809366	0.194227	-0.478943
76	H	-7.449017	1.165646	0.871311
77	C	-7.809621	-1.276794	-2.103814
78	H	-5.670754	-1.455404	-2.024210
79	C	-8.945065	-0.687001	-1.548546
80	H	-9.686891	0.651512	-0.035702
81	H	-7.907170	-1.970399	-2.931417
82	H	-9.927703	-0.917672	-1.943946

Table S15. Coordinates of the optimized geometry of *anti*-HT in S_0 state calculated by B3LYP/6-311G(*d*,*p*).

No.	Element	Coordinates (Å)		
		x	y	z
1	C	-0.673137	0.860048	-0.285718
2	C	-1.067349	1.871139	-1.372503
3	C	0.071285	1.409237	0.974380
4	C	0.721963	2.744041	0.731849
5	C	0.441691	3.484267	-0.414205
6	C	1.627202	3.277797	1.656411
7	C	1.053414	4.712989	-0.652916
8	C	2.240120	4.505631	1.437084
9	H	1.840684	2.719462	2.562328
10	C	1.952785	5.222370	0.273948
11	H	0.806156	5.246785	-1.561672
12	H	2.933451	4.903434	2.168288
13	O	-1.932169	1.635075	-2.171821
14	O	-0.469132	3.082852	-1.375918
15	H	-0.515842	1.433563	1.891206
16	C	-1.964668	0.077767	0.048840
17	O	-2.106495	-1.099350	-0.193298
18	N	-3.017500	0.751727	0.673799
19	C	-4.294382	0.049048	0.918641
20	C	-3.126027	2.102619	1.030448
21	C	-4.922782	1.005973	1.937841
22	C	-5.111461	-0.114096	-0.381503
23	H	-4.088869	-0.926199	1.356326
24	O	-2.336297	2.981864	0.820387
25	O	-4.295044	2.285550	1.670744
26	H	-4.694431	0.727362	2.968049
27	H	-5.996256	1.126965	1.816405
28	H	-4.472685	-0.601363	-1.120101
29	H	-5.359552	0.877358	-0.771007
30	C	-6.369752	-0.926689	-0.168758
31	C	-7.628768	-0.318854	-0.130834
32	C	-6.292990	-2.314818	0.005248
33	C	-8.781936	-1.074307	0.080734
34	H	-7.710321	0.752651	-0.283124
35	C	-7.442015	-3.071402	0.218157
36	H	-5.326108	-2.806199	-0.039221
37	C	-8.691294	-2.452444	0.258521
38	H	-9.749488	-0.585388	0.100930
39	H	-7.363713	-4.145360	0.345469
40	H	-9.586648	-3.041754	0.420448
41	C	0.117836	-3.434771	-2.377649
42	C	0.229144	-2.070576	-2.136435

43	C	0.431139	-1.580062	-0.841921
44	C	0.513346	-2.504816	0.198096
45	C	0.414311	-3.872513	-0.025393
46	C	0.216587	-4.338595	-1.320059
47	H	-0.048542	-3.790220	-3.387167
48	H	0.138311	-1.368941	-2.957156
49	C	0.607054	-0.103425	-0.550999
50	H	0.480539	-4.545388	0.820282
51	H	0.134416	-5.403878	-1.499574
52	C	0.815177	-0.851475	1.945307
53	C	1.061951	0.215350	0.900804
54	O	0.652897	-2.135757	1.536978
55	O	0.810291	-0.610562	3.122467
56	H	2.100822	0.513956	1.023554
57	C	1.520745	0.456600	-1.674719
58	O	1.040962	1.106978	-2.571579
59	N	2.867549	0.069071	-1.699460
60	C	3.642560	-0.784520	-0.767039
61	C	3.642652	0.237277	-2.889809
62	C	4.414528	-1.621712	-1.787151
63	C	4.588890	0.064905	0.120491
64	H	2.987924	-1.412676	-0.169826
65	O	3.509714	1.037940	-3.760490
66	O	4.625858	-0.698960	-2.877332
67	H	3.829618	-2.469271	-2.150175
68	H	5.385345	-1.958475	-1.434122
69	H	4.004553	0.813193	0.660368
70	H	5.259301	0.624772	-0.537210
71	C	5.401465	-0.761635	1.095472
72	C	6.730307	-1.097883	0.812009
73	C	4.845155	-1.209229	2.300324
74	C	7.478795	-1.870613	1.698401
75	H	7.189506	-0.739792	-0.103853
76	C	5.589810	-1.982191	3.187862
77	H	3.828586	-0.938342	2.565505
78	C	6.908741	-2.318610	2.887597
79	H	8.508034	-2.115462	1.461696
80	H	5.140965	-2.314185	4.117060
81	H	7.489673	-2.917254	3.579712
82	H	2.427011	6.179071	0.089963

Fig. S3 shows energy diagram of coordination of $ZnCl_2$ to coumarin (**1CX**) calculated by B3LYP/6-311G(*d,p*). Coordination of $ZnCl_2$ to coumarin is an exothermic reaction. Energies and geometries of three coumarin-$ZnCl_2$ complexes (**A**, **B**, and **C**) are shown in Table 16 and Fig. S4, respectively. It is found that **complex B** is most stable among three complexes so that $ZnCl_2$ is not coordinating the carbonyl group of the oxazolidinone between N and O. Tables S17-S19 show geometries of complexes listed in Table S16.

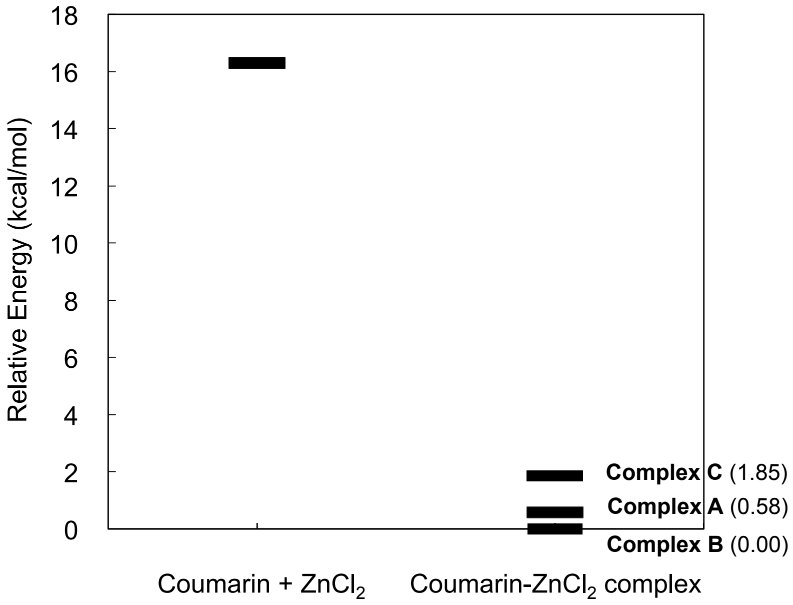

Fig. S3. Energy diagram of coordination of $ZnCl_2$ to coumarin (**1CX**) in S_0 state calculated by B3LYP/6-311G(*d,p*). Values in parentheses are energies relative to **complex B**.

Table S16. Electronic energies (*E*) of three coumarin-$ZnCl_2$ complexes in S_0 state optimized by B3LYP/6-311G(*d,p*) in toluene. Minimum values are shown with underline.

Complex	State	*E* (a.u.)	D*E* (kcal/mol)
**Complex A**	S_0	-3902.28193077	0.58
**Complex B**	S_0	**-3902.28285810**	**0.00**
**Complex C**	S_0	-3902.27991121	1.85

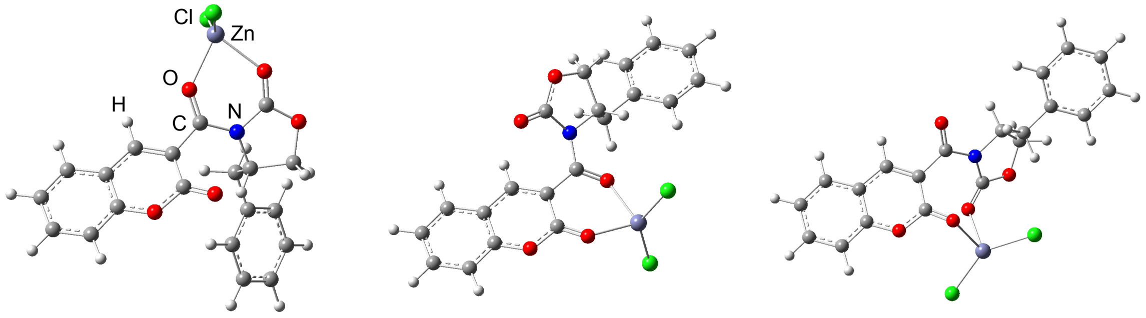

Complex A **Complex B** **Complex C**

Fig. S4. Optimized structures of three coumarin-$ZnCl_2$ complexes in S_0 state calculated by B3LYP/6-311G(*d*,*p*).

Table S17. Coordinates of the optimized geometry of **Complex A** in S_0 state calculated by B3LYP/6-311G(*d*,*p*).

No.	Element	Coordinates (Å)		
		x	y	z
1	C	-1.273656	-1.466438	-1.503377
2	C	-2.269616	-3.426109	-0.480980
3	C	-1.397895	-3.368556	0.620982
4	C	-0.441312	-2.313602	0.645397
5	C	-0.395427	-1.365668	-0.335824
6	H	-3.882967	-4.439545	-1.459003
7	C	-3.231986	-4.424578	-0.594620
8	C	-1.509342	-4.351281	1.626173
9	H	0.268837	-2.259699	1.463203
10	C	-2.461974	-5.346114	1.520225
11	C	-3.320641	-5.378413	0.409856
12	H	-0.836850	-4.314281	2.475122
13	H	-2.547079	-6.102817	2.289693
14	H	-4.064626	-6.162052	0.330449
15	O	-2.190138	-2.503859	-1.481781
16	O	-1.269021	-0.754512	-2.476402
17	C	0.708785	-0.368537	-0.271673
18	O	1.801812	-0.730987	0.156436
19	C	-0.823404	1.645964	-0.743469
20	C	-0.405098	2.877675	-1.554363
21	H	-1.534930	1.057546	-1.311952
22	H	-0.874068	3.798529	-1.218853
23	H	-0.555473	2.742015	-2.625010
24	N	0.481833	0.936678	-0.676642
25	O	1.025791	3.000696	-1.325218
26	C	1.524789	1.846604	-0.907577
27	O	2.713918	1.657167	-0.774407

28	Zn	3.757328	0.149758	0.337984
29	Cl	3.753237	1.036707	2.377750
30	Cl	4.947878	-1.000487	-1.138405
31	C	-1.357070	1.963966	0.668479
32	H	-1.437845	1.024400	1.223476
33	H	-0.630563	2.584923	1.199777
34	C	-2.705226	2.651672	0.628700
35	C	-2.837745	3.996611	0.987259
36	C	-3.847990	1.950212	0.223956
37	C	-4.080409	4.627843	0.940695
38	H	-1.966113	4.552163	1.317740
39	C	-5.089099	2.578351	0.174378
40	H	-3.769132	0.901941	-0.047479
41	C	-5.208344	3.921061	0.532029
42	H	-4.165229	5.669812	1.227341
43	H	-5.963626	2.019602	-0.139013
44	H	-6.174872	4.410230	0.496120

Table S18. Coordinates of the optimized geometry of **Complex B** in S_0 state calculated by B3LYP/6-311G(*d*,*p*).

No.	Element		Coordinates (Å)	
		x	y	z
1	C	2.481164	0.776734	0.170619
2	C	4.441140	-0.524937	-0.306197
3	C	3.681618	-1.680598	-0.552719
4	C	2.273949	-1.592491	-0.373127
5	C	1.672733	-0.424955	0.005515
6	H	6.375238	0.391577	-0.224096
7	C	5.826012	-0.519373	-0.422220
8	C	4.351911	-2.861907	-0.933124
9	H	1.679505	-2.476894	-0.555519
10	C	5.727525	-2.867419	-1.052246
11	C	6.459697	-1.696590	-0.795488
12	H	3.772828	-3.758592	-1.119768
13	H	6.245119	-3.773338	-1.340923
14	H	7.538785	-1.708532	-0.890172
15	O	3.828309	0.643717	0.046914
16	O	2.081684	1.903772	0.423971
17	C	0.206782	-0.294177	0.165065
18	O	-0.379820	0.758673	-0.091324
19	C	-2.035026	-1.250144	0.537000
20	C	-2.454304	-2.487061	1.351007
21	H	-2.302563	-0.329509	1.057350
22	H	-2.852170	-3.282505	0.720390
23	H	-3.163630	-2.258613	2.142524
24	N	-0.556593	-1.381377	0.558281

25	O	-1.245208	-2.982993	1.968828
26	C	-0.151462	-2.388240	1.482477
27	O	0.963694	-2.679248	1.805577
28	Zn	0.202906	2.802204	0.164138
29	Cl	-0.634864	3.173800	2.198910
30	Cl	0.211888	3.742172	-1.848286
31	C	-2.613046	-1.228133	-0.888709
32	H	-2.171833	-0.389384	-1.429521
33	H	-2.317952	-2.147862	-1.402275
34	C	-4.122483	-1.093719	-0.882086
35	C	-4.947274	-2.183825	-1.176870
36	C	-4.720150	0.133905	-0.569642
37	C	-6.335665	-2.055199	-1.154952
38	H	-4.502460	-3.138349	-1.439607
39	C	-6.105855	0.264433	-0.545588
40	H	-4.097210	0.996152	-0.355079
41	C	-6.918233	-0.831120	-0.836740
42	H	-6.959430	-2.909859	-1.391482
43	H	-6.550956	1.223460	-0.306382
44	H	-7.997186	-0.728241	-0.821734

Table S19. Coordinates of the optimized geometry of **Complex C** in S_0 state calculated by B3LYP/6-311G(*d*,*p*).

No.	Element		Coordinates (Å)	
		x	y	z
1	C	-1.902551	-0.230709	-0.74666
2	C	-4.118862	-1.120508	-0.52022
3	C	-3.643087	-2.295879	0.084263
4	C	-2.234949	-2.414090	0.268628
5	C	-1.384953	-1.407094	-0.08304
6	H	-5.797708	0.023354	-1.19833
7	C	-5.474633	-0.900095	-0.73629
8	C	-4.577073	-3.277829	0.473264
9	H	-1.832966	-3.320497	0.707425
10	C	-5.926138	-3.071087	0.261046
11	C	-6.370938	-1.882347	-0.34098
12	H	-4.220082	-4.188681	0.939313
13	H	-6.644865	-3.823510	0.560333
14	H	-7.431058	-1.726015	-0.49964
15	O	-3.244673	-0.151704	-0.92693
16	O	-1.229087	0.693409	-1.19047
17	C	0.087302	-1.653018	0.067549
18	O	0.564941	-2.707882	-0.29797
19	C	2.375215	-0.950335	0.767225
20	C	2.730671	-0.058811	1.966361
21	H	2.547750	-2.002013	0.987751

22	H	3.630776	0.531313	1.816404
23	H	2.798494	-0.613650	2.902311
24	N	0.915569	-0.711459	0.686514
25	O	1.614659	0.865032	2.088411
26	C	0.570823	0.433840	1.393524
27	O	-0.504914	1.002663	1.404435
28	Zn	-0.931013	2.525897	-0.17621
29	Cl	-2.912476	3.322603	0.379161
30	Cl	1.143559	3.061622	-0.77553
31	C	3.071181	-0.562450	-0.5536
32	H	2.609298	-1.143918	-1.35528
33	H	2.873744	0.492564	-0.7615
34	C	4.560364	-0.828419	-0.51607
35	C	5.475984	0.223431	-0.40852
36	C	5.050858	-2.138970	-0.58254
37	C	6.847350	-0.026171	-0.36235
38	H	5.114188	1.245880	-0.37721
39	C	6.419325	-2.390816	-0.53575
40	H	4.356240	-2.966892	-0.68622
41	C	7.322358	-1.333819	-0.42335
42	H	7.542249	0.802449	-0.28525
43	H	6.781852	-3.410996	-0.59491
44	H	8.388203	-1.528990	-0.39116

Synthesis of chiral coumarin-3-carboxamides.

To a suspension of coumarin-3-carboxylic acid (5.00 g, 26.3 mmol, 1.00 equiv.) in anhydrous CH_2Cl_2 (130 mL) at 0 ^oC under an argon atmosphere, oxalyl chloride (2.67 mL, 31.6 mmol, 1.20 equiv.) and anhydrous DMF (2 drops) were added. The resulting suspension was warmed to room temperature and stirred for 24 h. The reaction solvent and volatile compounds were evaporated and dried under high vacuum for an hour to give coumarin-3-carboxylic acid chloride which was used for the preparation of **1C** without purification. In a dried round bottom flask under argon atmosphere, (*S*)-4-benzyl-1,3-oxazolidine-2-one (4.66 g, 26.3 mmol, 1.00 equiv.) was dissolved in anhydrous THF (130 mL) and cooled to -78 ^oC, and then *n*-butyllithium (1.57 M in *n*-hexane solution) (17.6 mL, 27.6 mmol, 1.05 equiv.) was added dropwise and stirred for 30 min. The resulting suspension was warmed to 0 ^oC and stirred for 30 min. A solid coumarin-3-carboxylic acid chloride was added to the reaction mixture in one portion under an argon stream. The resulting suspension was warmed to 0 ^oC and stirred for 30 min. The reaction mixture was allowed to warm to room temperature and stirred for 24 h. The resulting suspension was poured into saturated aqueous NH_4Cl and extracted with AcOEt. The combined organic layer was washed with brine, dried over anhydrous Na_2SO_4, filtered and evaporated. The resulting pale yellow powder was recrystallized from AcOEt to give colorless precipitate which was collected by filteration and washed with AcOEt to give analytically pure **1C** (6.89 g) in 75% yield.

In a synthesis of **1A**, the reaction mixture was poured into saturated aqueous NH_4Cl to give colorless precipitate, which was washed with water and AcOEt. The resulting colorless powder was suspended in AcOEt and refluxed for 1 hr. The resulting a hot suspension was filtered and washed with pre-warmed AcOEt to give analytically pure **1A**.

1A: Colorless powder; mp 274-275 ^oC; $[\alpha]_D^{23}$ +173.7 (c 0.13, DMSO); IR (KBr) ν 3043, 2983, 1789, 1728, 1698, 1608, 1335, 1124, 757, 700 cm^{-1}; ^1H NMR (400 MHz, DMSO-d_6) δ 4.24 (1H, dd, *J* = 4.9, 8.8 Hz), 4.87 (1H, dd, *J* = 8.8, 8.8 Hz), 5.67 (1H, dd, *J* = 4.9, 8.8 Hz), 7.34-7.51 (7H, m), 7.70-7.74 (1H, m), 7.86 (1H, dd, *J* = 1.5, 7.8 Hz), 8.30 (1H, s); ^{13}C NMR (100 MHz, DMSO-d_6) δ 56.9, 70.8, 116.4, 117.9, 123.8, 125.1, 125.9, 128.1, 128.8, 129.5, 133.5, 138.7, 143.0, 153.3, 153.5, 157.4, 163.1; HRMS (ESI-TOF) Calcd for $C_{19}H_{13}NO_5Na$ $[M+Na]^+$: 358.0691; Found: 358.0690.

1B: Colorless powder; mp 188-189 ^oC; $[\alpha]_D^{23}$ +157.5 (c 0.13, $CHCl_3$); IR (KBr) ν 3047, 2966, 1794, 1731, 1685, 1609, 1372, 1118, 754, 718 cm^{-1}; ^1H NMR (400 MHz, $CDCl_3$) δ 1.00 (3H, d, *J* = 7.0 Hz), 1.04 (3H, d, *J* = 7.0 Hz), 2.53-2.58 (1H, m), 4.30 (1H, dd, *J* = 3.7, 9.1 Hz), 4.42 (1H, dd, *J* = 8.8, 8.8 Hz), 4.55-4.59 (1H, m), 7.29-7.31 (2H, m), 7.54-7.61 (2H, m), 7.89 (1H, s); ^{13}C NMR (100 MHz, $CDCl_3$) δ 14.9, 18.0, 28.6, 58.9, 64.2, 117.2, 118.2, 124.8, 124.9, 128.9, 133.3, 142.8, 153.7, 154.5, 157.9, 164.2; HRMS (ESI-TOF) Calcd for $C_{16}H_{15}NO_5Na$ $[M+Na]^+$: 324.0848; Found: 324.0864.

1C: Colorless powder; mp 166-167 ^oC; $[\alpha]_D^{23}$ +68.5 (c 0.14, $CHCl_3$); IR (KBr) ν 3078, 3020, 1780, 1740, 1687, 1606, 1385, 1114, 762, 704 cm^{-1}; ^1H NMR (400 MHz, $CDCl_3$) δ 2.92 (1H, dd, *J* = 9.8, 13.5 Hz), 3.52 (1H, dd, *J* = 3.6, 13.5 Hz), 4.24 (1H, dd, *J* = 3.5, 9.0 Hz), 4.34 (1H, dd, *J* = 8.0, 9.0 Hz), 4.77-4.84 (1H, m), 7.26-7.37 (7H, m), 7.55-7.62 (2H,

m), 7.95 (1H, s); ^{13}C NMR (100 MHz, $CDCl_3$) δ 37.8, 55.6, 67.1, 117.1, 118.1, 124.5, 125.0, 127.5, 129.05, 129.14, 129.6, 133.5, 135.1, 143.2, 153.1, 154.5, 157.9, 164.2; HRMS (ESI-TOF) Calcd for $C_{20}H_{15}NO_5Na$ $[M+Na]^+$: 372.0842; Found: 372.0843.

X-Ray diffraction analysis data of **1A**: $P2_12_12_1$, a = 6.1686 (11) Å, b = 15.664 (3) Å, c = 16.396 (3) Å, α 90°, β 90°, γ 90°, V = 1584.3 (5) Å^3, Z = 4, ρ = 1.465 g cm^{-3}, μ = 0.106 mm^{-1}. The structure was solved by the direct method. And the refinement was performed using the full matrix least-squares, where the final R and wR were 0.0327 and 0.0692 for 5186 reflections. CCDC-1552472.

1D: Colorless powder; mp 225-227 °C; $[\alpha]_D^{23}$ -144.2 (c 0.11, CH_2Cl_2); IR (KBr) ν 3058, 1781, 1708, 1362, 1220, 1146, 987, 760 cm^{-1}; ^{1}H NMR (400 MHz, $CDCl_3$) δ 2.85 (1H, dd, J = 7.6, 14.3 Hz), 3.07 (1H, dd, J = 5.4, 14.3 Hz), 5.73 (1H, dd, J = 5.4, 7.6 Hz), 6.88-6.90 (2H, m), 7.13-7.15 (3H, m), 7.25-7.39 (9H, m), 7.46-7.49 (3H, m), 7.55-7.59 (1H, m), 7.70 (1H, s); ^{13}C NMR (100 MHz, $CDCl_3$) δ 37.1, 63.1, 89.6, 117.2, 118.1, 124.4, 124.9, 126.3, 126.7, 126.8, 128.4, 128.5, 128.96, 128.98, 129.1, 129.3, 133.3, 136.3, 137.58, 137.59, 141.2, 143.0, 151.7, 154.6, 157.6, 163.5; HRMS (ESI-TOF) Calcd for $C_{32}H_{23}NO_5Na$ $[M+Na]^+$: 524.1474; Found: 524.1455.

Typical procedure for photocycloadducts.

In a Pyrex test tube, a solution of **1C** (50.0 mg, 0.143 mmol, 1.00 equiv.) and $Zn(ClO_4)_2$•$6H_2O$ (6.78 mg, 1.82 x 10^{-2} mmol, 0.13 equiv.) were dissolved in anhydrous toluene (1.40 mL) and degassed by argon bubbling for 15 min with stirring. The Pyrex test tube was wrapped roughly with aluminum foil and the external irradiation was directed toward the tube with a working distance of 1 cm. The UV-LED light sources (OMRON ZUV-H20MB) were controlled by OMRON ZUV-C20H [irradiation power was set at maximum value at the controller; mean value and error range of irradiation power of UV-LED light sources was measured by UV meter (USHIO UIT-250) equipped with a UV sensor (UVD-S365) to be revealed as 422 mW/cm^2 and ± 3% respectively][1] for 48 h. Toluene was removed by evaporation. The resulting mixture was purified by flash silica gel column chromatography (FC) eluted with *n*-hexane/EtOAc = 4/1-1/1 to provide analytically pure cycloadducts **2CE**, **2CF** and a mixture of **2CE** and **2CF** (net 46.0 mg) in 92% yield. Analytically pure cycloadducts **2CE** and **2CF** were subjected to recrystallization from appropriate solvents.

syn-HT **2CE**: Colorless prisms from *n*-hexane/$CHCl_3$; mp 124-126 °C; $[\alpha]_D^{23}$ +12.9 (c 0.16, $CHCl_3$); IR (KBr) ν 3063, 3028, 2977, 2922, 1777, 1697, 1455, 1393, 1249, 1116, 761, 703 cm^{-1}; ^{1}H NMR (500 MHz, $CDCl_3$) δ 2.91 (2H, dd, J = 10.2, 13.6 Hz), 3.50 (2H, dd, J = 3.1, 13.6 Hz), 4.29 (2H, dd, J = 2.9, 9.2 Hz), 4.33 (2H, dd, J = 7.7, 9.2 Hz), 4.74-4.79 (2H, m), 5.18 (2H, s), 6.62-6.64 (2H, m), 7.14-7.37 (16H, m); ^{13}C NMR (125 MHz, $CDCl_3$) δ 37.9, 44.9, 55.7, 55.9, 67.7, 115.8, 117.2, 125.5, 127.6, 128.8, 129.2, 129.4, 130.0, 134.8, 150.8, 153.9, 161.4, 166.8; HRMS (ESI-TOF) Calcd for $C_{40}H_{30}N_2O_{10}Na$ $[M+Na]^+$: 721.1793; Found: 721.1796.

X-Ray diffraction analysis data of *syn*-HT **2CE**: orthorhombic space group $P2_12_12_1$, a = 10.620 (5) Å, b = 15.369 (6) Å, c = 21.972 (9) Å, α 90°, β 90°, γ 90°, V = 3586 (3) Å^3, Z = 4, ρ = 1.489 g cm^{-3}, μ = 0.321 mm^{-1}. The structure was solved by the direct method. And the refinement was performed using the full matrix least-squares, where the final R and wR were 0.0956 and 0.2672 for 6314 reflections. CCDC-1484443.

syn-HT **2CF**: Colorless prisms from *n*-hexane/EtOAc; mp 328-330 °C; $[\alpha]_D^{23}$ +133.0 (c 0.13, CH_3CN); IR (KBr) ν 3067, 3033, 2974, 2919, 1775, 1747, 1698, 1493, 1356, 1245, 1116, 754, 702 cm^{-1}; ^{1}H NMR (500 MHz, $CDCl_3$) δ 2.92 (2H, dd, J = 9.6, 13.6 Hz), 3.47 (2H, dd, J = 3.3, 13.6 Hz), 4.28 (2H, dd, J = 2.6, 8.8 Hz), 4.42 (2H, dd, J = 8.5, 8.8 Hz), 4.78-4.83 (2H, m), 5.08 (2H, s), 6.64 (2H, d, J = 8.0 Hz), 7.13-7.38 (16H, m); ^{13}C NMR (125 MHz, $CDCl_3$) δ 37.1, 44.6, 55.6, 55.8, 67.3, 115.8, 117.2, 125.4, 127.6, 128.8, 129.2, 129.6, 130.0, 134.8, 150.8, 153.8, 161.3, 167.1; HRMS (ESI-TOF) Calcd for $C_{40}H_{30}N_2O_{10}Na$ $[M+Na]^+$: 721.1793; Found: 721.1794.

X-Ray diffraction analysis data of *syn*-HT **2CF**: orthorhombic space group $P2_12_12_1$, a = 8.2036 (10) Å, b = 18.123 (2) Å, c = 21.423 (3) Å, α 90°, β 90°, γ 90°, V = 3185.0 (7) Å^3, Z = 4, ρ = 1.457 g cm^{-3}, μ = 0.106 mm^{-1}. The structure was solved by the direct method. And the refinement was performed using the full matrix least-squares, where the final R and wR were 0.0552 and 0.1422 for 8883 reflections. CCDC-1552473.

A 50:50 diastereomeric mixture of *syn*-HT **2BE** and *syn*-HT **2BF**: : Colorless prisms from CH_3CN; mp 380-382 °C; $[\alpha]_D^{23}$ +59.6 (c 0.21, CH_3CN); IR (KBr) ν 2973, 1774, 1695, 1588, 1490, 1367, 1217, 1186, 761 cm^{-1}; ^{1}H NMR (400 MHz, DMSO-d_6) δ 0.83 (6H, d, J = 6.9 Hz), 0.91 (6H, d, J = 6.9 Hz), 0.93 (6H, d, J = 6.8 Hz), 0.97 (6H, d, J = 6.8 Hz), 2.22-2.34 (2H, m), 2.34-2.44 (2H, m), 4.41-4.64 (12H, m), 4.98 (2H, s), 5.02 (2H, s), 6.63-6.71 (4H, m), 6.95-6.99 (4H, m), 7.12-7.39 (4H, m), 7.22-7.28 (4H, m); ^{13}C NMR (100 MHz, DMSO-d_6) δ 14.1, 14.3, 17.2, 17.4, 27.57, 27.58, 43.5, 44.3, 54.7, 55.4, 58.4, 58.6, 64.6, 64.8, 115.9, 116.3, 116.5, 116.6, 124.9, 125.1, 128.7, 128.8, 129.7, 129.9, 150.3, 150.5, 154.3, 154.4, 160.6, 160.9, 165.80, 165.81; HRMS (ESI-TOF) Calcd for $C_{32}H_{30}N_2O_{10}Na$ $[M+Na]^+$: 625.1798; Found: 625.1771.

X-Ray diffraction analysis data of a 50:50 diastereomeric mixture of *syn*-HT **2BE** and **2BF**: orthorhombic space group $P2_12_12_1$, a = 20.0542 (4) Å, b = 11.4435 (2) Å, c = 12.2782 (2) Å, α 90°, β 90°, γ 90°, V = 2817.72 (9) Å^3, Z = 4, ρ = 1.420 g cm^{-3}, μ = 0.89 mm^{-1}. The structure was solved by the direct method. And the refinement was performed using the full matrix least-squares, where the final R and wR were 0.089 and 0.302 for 5177 reflections. CCDC-1885142.

Removel of the chiral auxiliary and the stereoselective reduction.

In a round-bottom flask, $NaBH_4$ (18.0 mg, 0.476 mmol, 25.6 equiv.) was added to a solution of *syn*-HT **2CE** (13.0 mg, 0.0186 mmol, 1.00 equiv.) in THF (2.00 mL) at room temperature and stirred for 4 h. The resulting reaction mixture was poured into saturated aqueous NH_4Cl and stirred for 1 h. The resulting suspension was diluted with water and extracted with EtOAc. The combined organic layer was washed with brine, dried over anhydrous $MgSO_4$, filtered and evaporated. The crude mixture was purified by flash silica gel column chromatography (FC) eluted with *n*-hexane/EtOAc = 4/1-1/1 to provide analytically pure chromane-2-ol derivative **3** (2.50 mg) in 37% yield.

3: Colorless powder; mp 130-132 ^{o}C; $[\alpha]_D^{23}$ +4.38 (c 0.16, MeOH); IR (KBr) ν 3403, 2928, 1743, 1586, 1487, 1455, 1406, 1235, 1189, 1122, 1106, 1057, 974, 757 cm^{-1}; ^1H NMR (600 MHz, CD_3OD) δ 3.61 (1H, s), 3.73 (1H, d, *J* = 12.4 Hz), 3.96 (1H, d, *J* = 12.4 Hz), 4.05 (1H, d, *J* = 12.4 Hz), 4.06 (1H, d, *J* = 12.4 Hz), 4.16 (1H, s), 6.78 (1H, app t, *J* = 7.5 Hz), 6.82 (1H, dd, *J* = 0.6, 8.0 Hz), 6.90 (1H, d, *J* = 8.0 Hz), 7.00 (1H, app t, *J* = 7.4 Hz), 7.10 (1H, app t, *J* = 7.7 Hz), 7.12 (1H, app t, *J* = 7.6 Hz), 7.20-7.26 (2H, m); ^{13}C NMR (150 MHz, CD_3OD) δ 36.2, 43.5, 46.8, 59.1, 60.4, 62.5, 98.9, 116.6, 118.5, 120.7, 120.8, 123.6, 123.8, 128.9, 129.5, 130.6, 130.9, 151.5, 156.6, 175.7; HRMS (ESI-TOF) Calcd for $C_{20}H_{18}N_2O_6Na$ $[M+Na]^+$: 377.1001; Found: 377.1000.

Limitation of chiral coumarin-3-carboxamides.

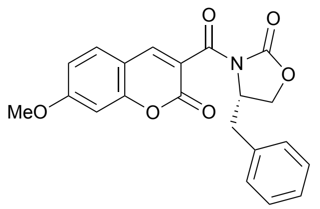

S1
no raction

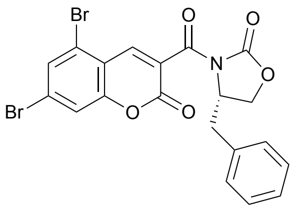

S2
complex mixture

Fig. S4. Chiral coumarin-3-carboxamide **S1** did not dimerize under photoirradiation. In the case of **S2**, a complex mixture of unknown products were observed in ^1H NMR spectrum.

S1: Pale yellow powder; mp 211-213 ^{o}C; $[\alpha]_D^{23}$ +77.1 (c 0.49, CH_2Cl_2); IR (KBr) ν 3029, 1787, 1719, 1605, 1387, 1356, 1213, 1117, 932, 759, 703 cm^{-1}; ^1H NMR (400 MHz, $CDCl_3$) δ 3.06 (1H, dd, *J* = 7.7, 13.6 Hz), 3.18 (1H, dd, *J* = 3.5, 13.6 Hz), 3.89 (3H, s), 4.27 (1H, dd, *J* = 3.4, 9.0 Hz), 4.43 (1H, dd, *J* = 8.6, 9.0 Hz), 4.48-4.88 (1H, m), 7.03 (1H, dd, *J* = 2.4, 8.7 Hz), 7.09 (1H, d, *J* = 2.4 Hz), 7.22-7.30 (1H, m), 7.33 (4H, d, *J* = 4.4 Hz), 7.77 (1H, d, *J* = 8.7 Hz), 8.27 (1H, s); ^{13}C NMR (100 MHz, $CDCl_3$) δ 36.6, 54.5, 56.2, 66.8, 100.8, 111.4, 113.2, 120.3, 127.0, 128.6, 129.6, 130.7, 135.4, 143.2, 152.9, 155.7, 157.6, 163.7, 163.9; HRMS (ESI-TOF) Calcd for $C_{21}H_{17}NO_6Na$ $[M+Na]^+$: 402.0953; Found: 402.0938.

S2: Pale orange powder; mp 220-222 ^{o}C; $[\alpha]_D^{23}$ +81.8 (c 0.80, CH_2Cl_2); IR (KBr) ν 3058, 1784, 1736, 1685, 1388, 1366, 1004, 962, 745, 703 cm^{-1}; ^{1}H NMR (400 MHz, $CDCl_3$) δ 3.08 (1H, dd, *J* = 7.3, 13.6 Hz), 3.17 (1H, dd, *J* = 3.5, 13.6 Hz), 4.29 (1H, dd, *J* = 3.3, 8.9 Hz), 4.46 (1H, dd, *J* = 8.6, 8.9 Hz), 4.79-4.94 (1H, m), 7.25-7.30 (1H, m), 7.34 (4H, d, *J* = 4.4 Hz), 8.16 (1H, d, *J* = 2.3 Hz), 8.25 (1H, d, *J* = 2.3 Hz); ^{13}C NMR (100 MHz, $CDCl_3$) δ 36.4, 54.3, 67.0, 111.4, 110.4, 116.8, 120.8, 125.7, 127.0, 128.6, 129.7, 131.1, 135.2, 137.7, 140.8, 149.3, 153.0, 156.3, 162.7; HRMS (ESI-TOF) Calcd for $C_{20}H_{13}{}^{79}Br^{81}BrNO_5Na$ [M+Na]$^+$: 529.9038; Found: 529.9019.

^{1}H NMR and ^{13}C NMR spectra of coumarins and photodimers

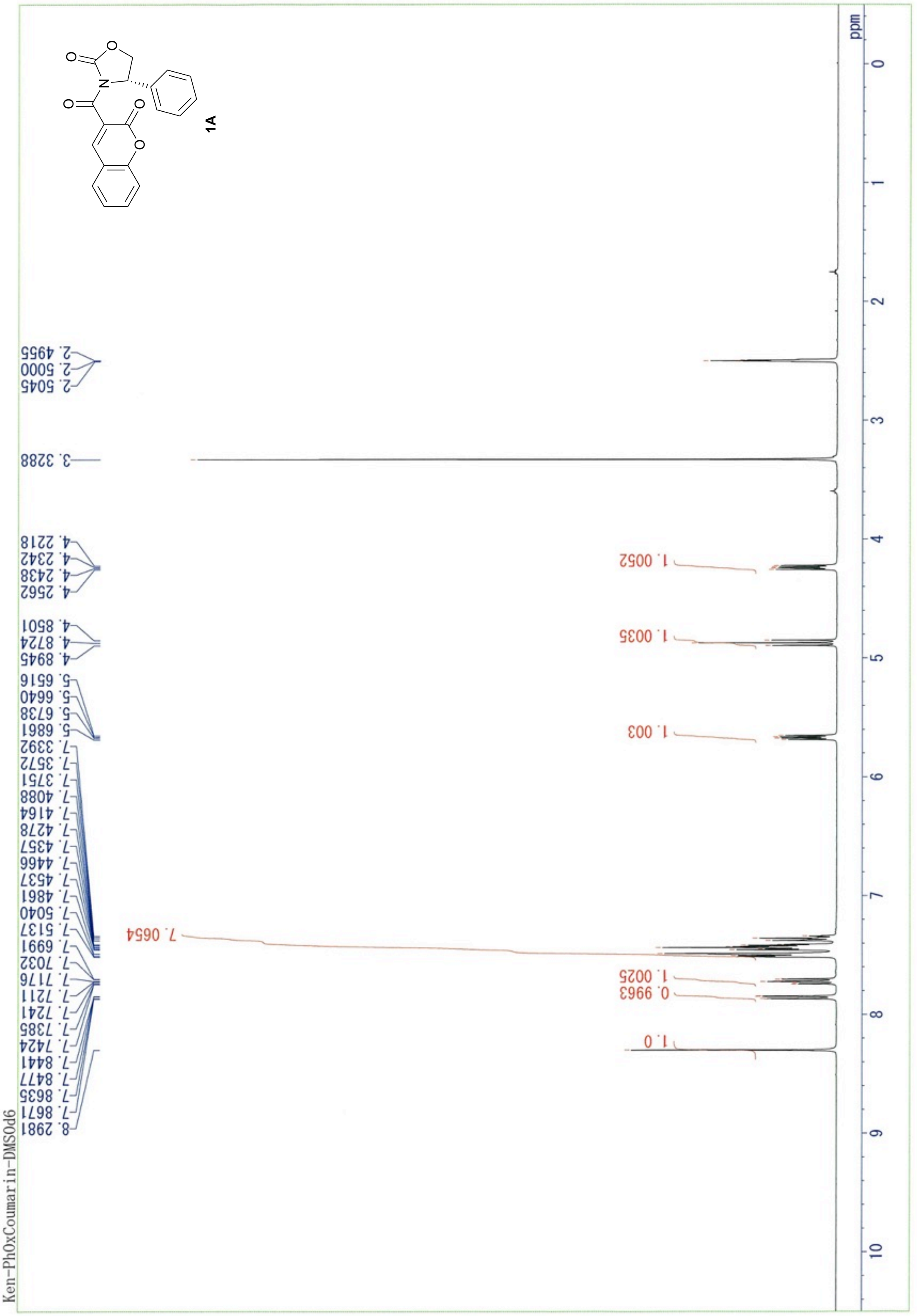

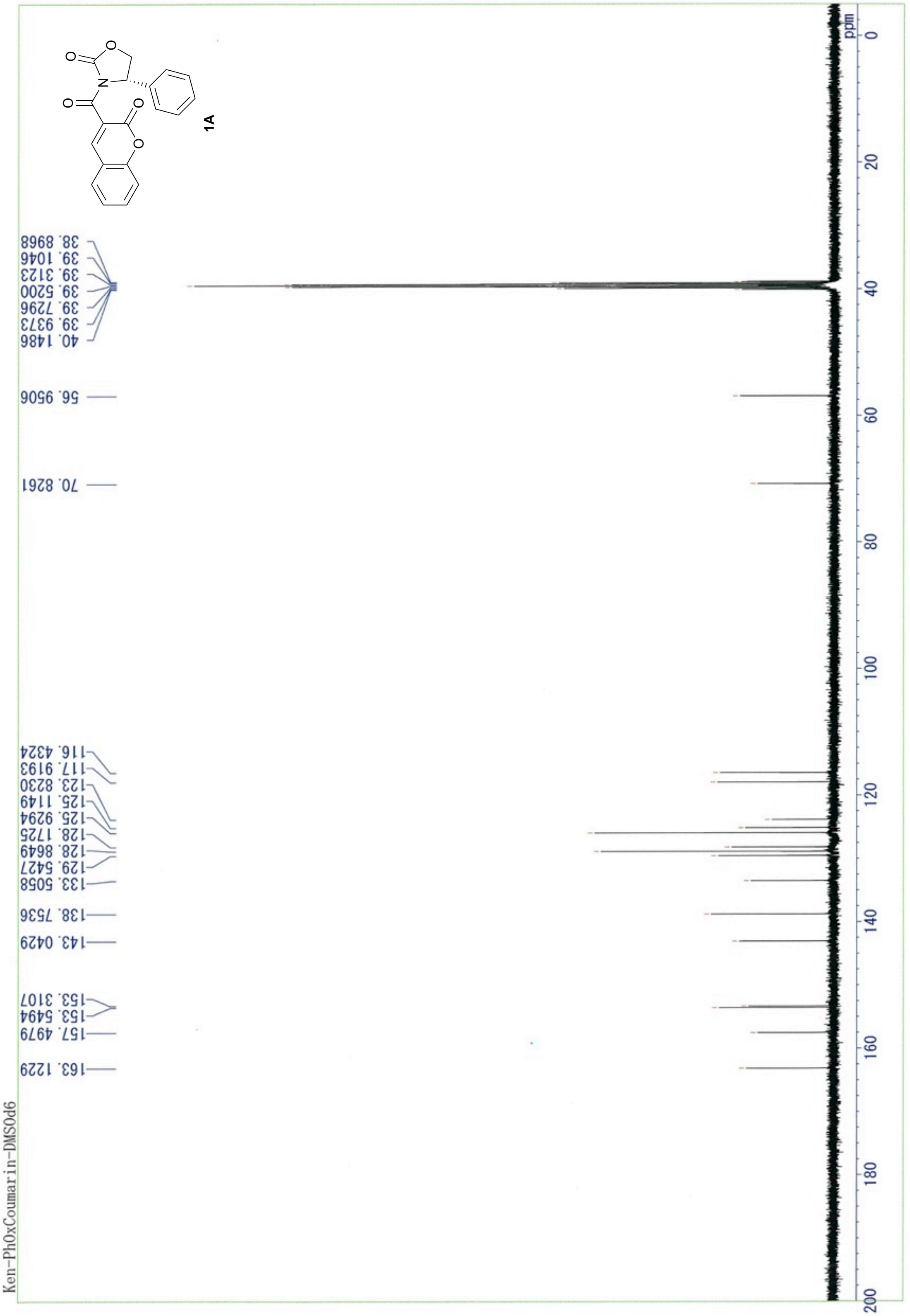
1A
Ken-PhOxCoumarin-DMSOd6
38.8968
39.1046
39.3123
39.5200
39.7296
39.9373
40.1486
56.9506
70.8261
116.4324
117.9193
123.8230
125.1149
125.9294
128.1725
128.8649
129.5427
133.5058
138.7536
143.0429
153.3107
153.5494
157.4979
163.1229
ppm
0
20
40
60
80
100
120
140
160
180
200

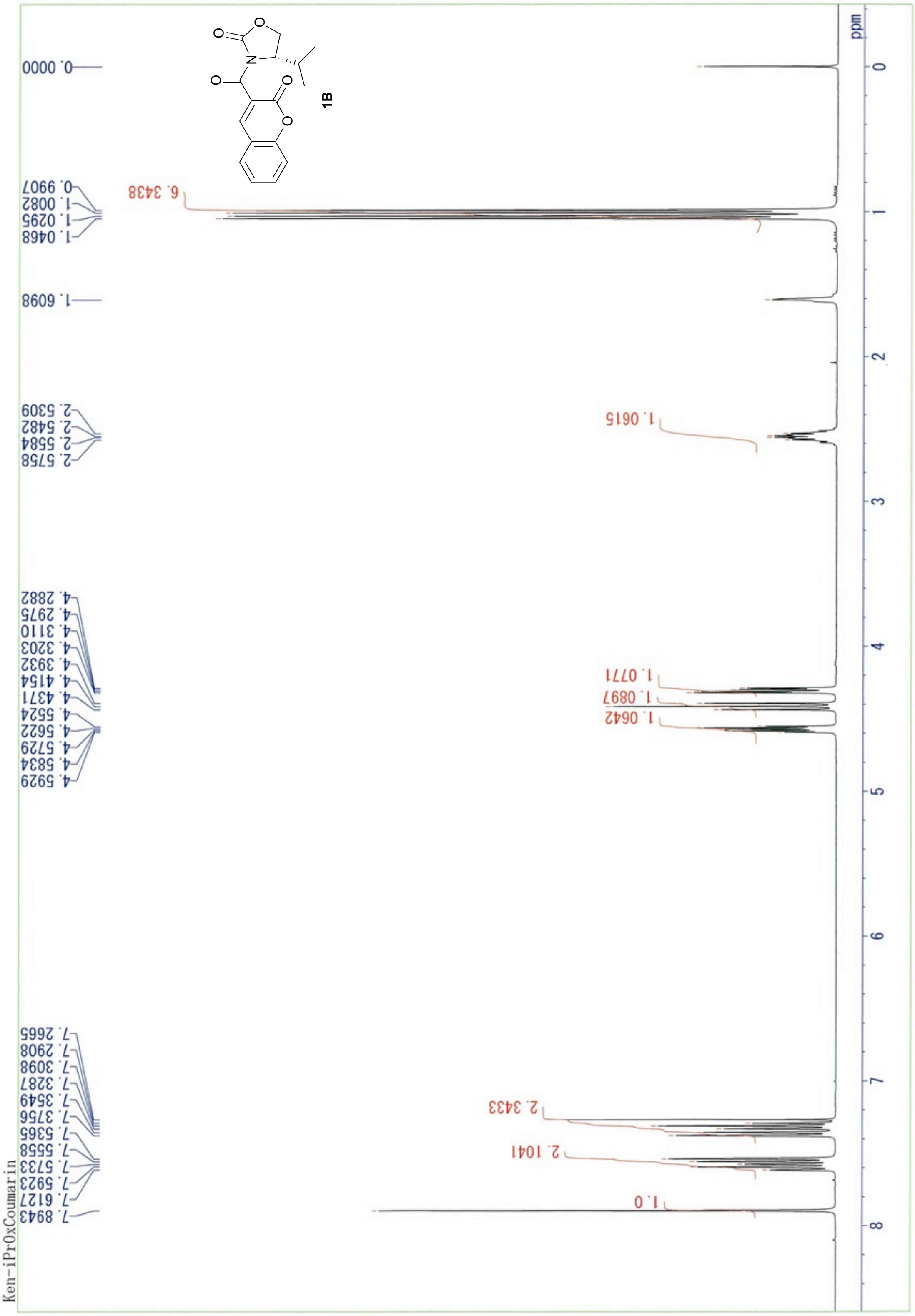
Ken-iPrOxCoumarin
1B
0.0000
0.9907
1.0082
1.0295
1.0468
1.6098
2.5309
2.5482
2.5584
2.5758
4.2882
4.2975
4.3110
4.3203
4.3932
4.4154
4.4371
4.5524
4.5622
4.5729
4.5834
4.5929
7.2665
7.2908
7.3098
7.3287
7.3549
7.3756
7.5365
7.5558
7.5733
7.5923
7.6127
7.8943
6.3438
1.0615
1.0771
1.0897
1.0642
2.3433
2.1041
1.0
ppm
0
1
2
3
4
5
6
7
8

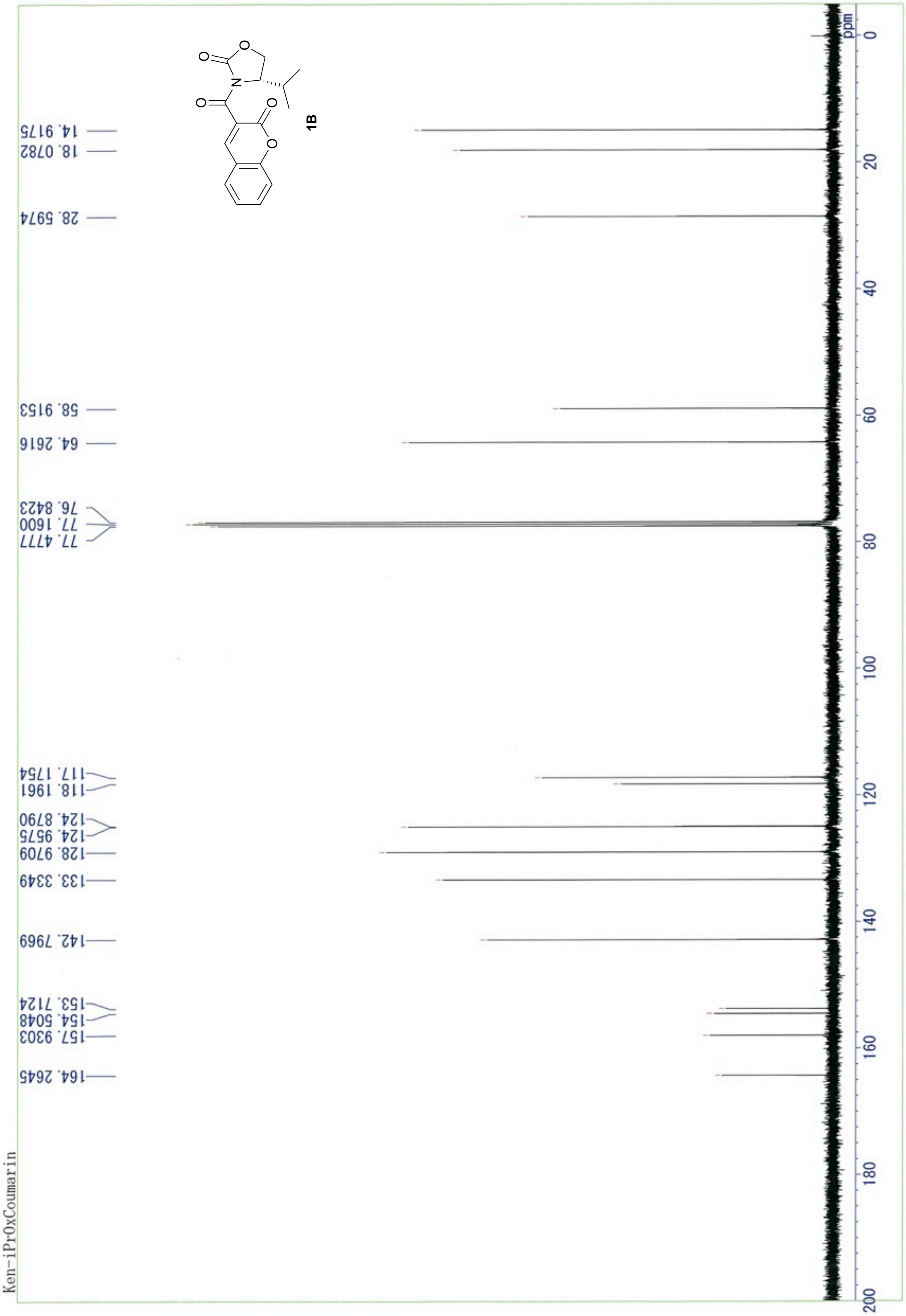
Ken-iPrOxCoumarin
1B
14.9175
18.0782
28.5974
58.9153
64.2616
76.8423
77.1600
77.4777
117.1754
118.1961
124.8790
124.9575
128.9709
133.3349
142.7969
153.7124
154.5048
157.9303
164.2645
ppm

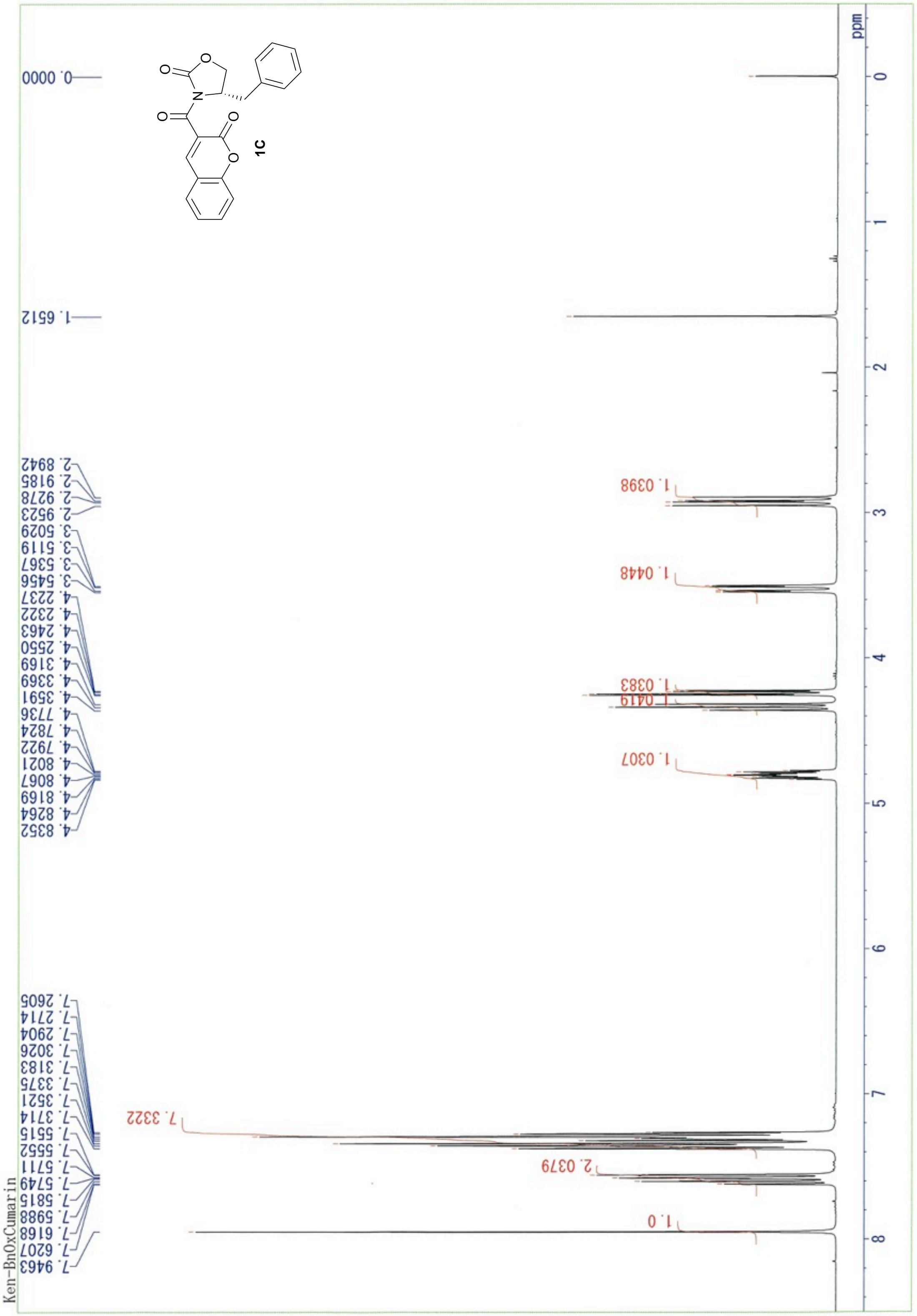

Ken-BnOxCumarin
1c
0.0000
1.6512
2.8942
2.9185
2.9278
2.9523
3.5029
3.5119
3.5367
3.5456
4.2237
4.2322
4.2463
4.2550
4.3169
4.3369
4.3591
4.7736
4.7824
4.7922
4.8021
4.8067
4.8169
4.8264
4.8352
7.2605
7.2714
7.2904
7.3026
7.3183
7.3375
7.3521
7.3714
7.5515
7.5552
7.5711
7.5749
7.5815
7.5988
7.6168
7.6207
7.9463
1.0398
1.0448
1.0383
1.0419
1.0307
7.3322
2.0379
1.0
ppm
0
1
2
3
4
5
6
7
8

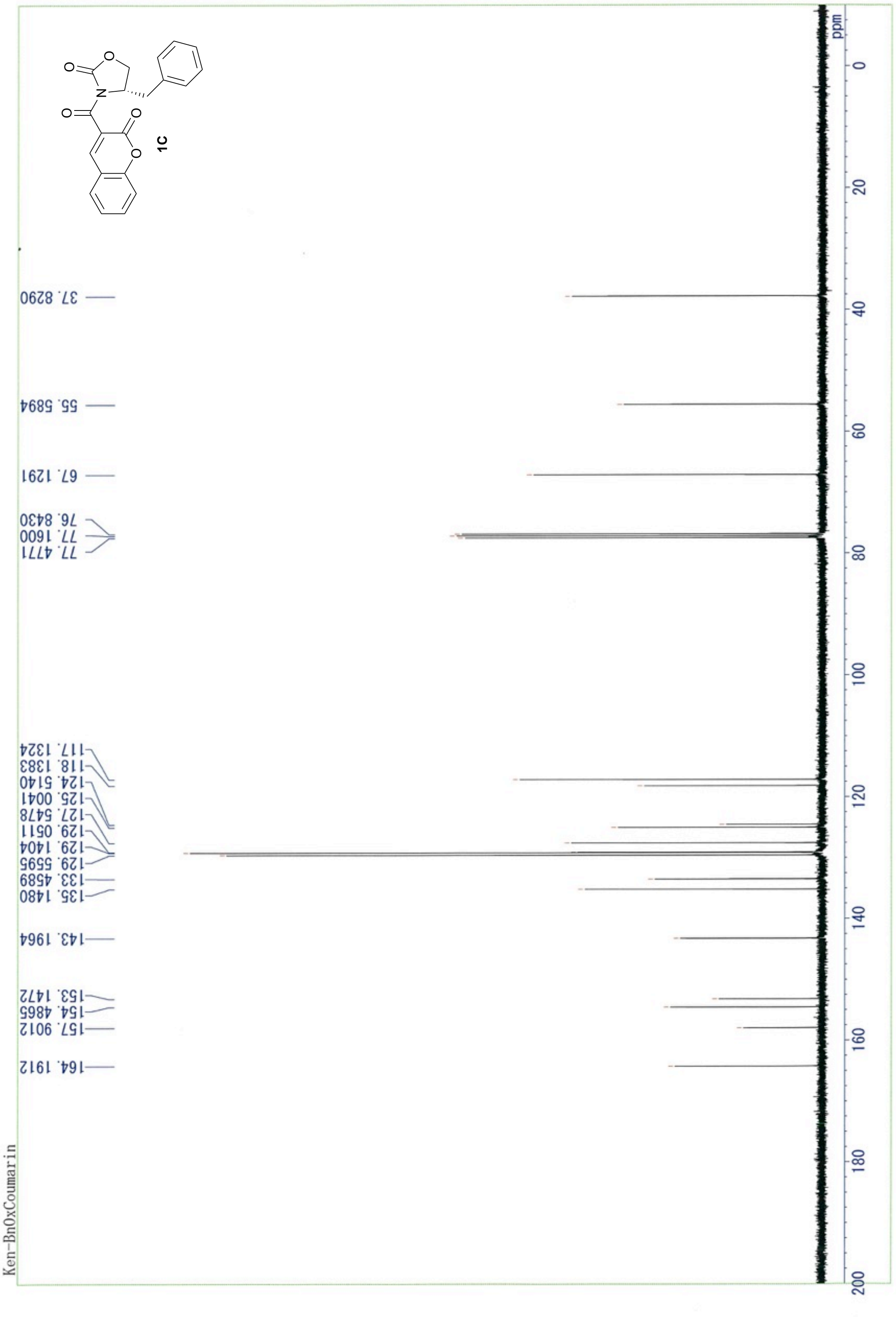
1c
37.8290
55.5894
67.1291
76.8430
77.1600
77.4771
117.1324
118.1383
124.5140
125.0041
127.5478
129.0511
129.1404
129.5595
133.4589
135.1480
143.1964
153.1472
154.4865
157.9012
164.1912
Ken-BnOxCoumarin
ppm
0
20
40
60
80
100
120
140
160
180
200

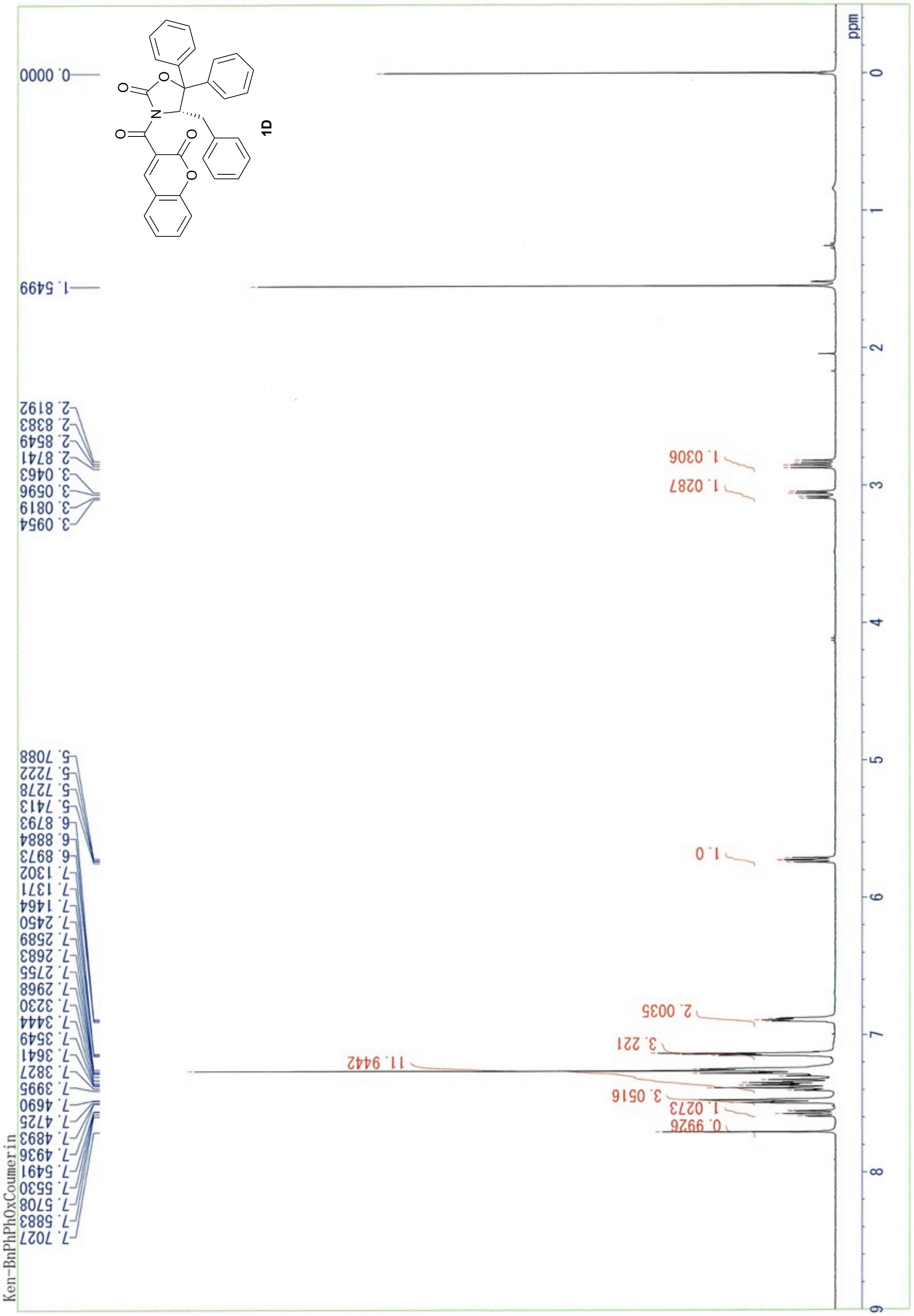

Ken-BnPhPhOxCoumerin
1D
0.0000
1.5499
2.8192
2.8383
2.8549
2.8741
3.0463
3.0596
3.0819
3.0954
5.7088
5.7222
5.7278
5.7413
6.8793
6.8884
6.8973
7.1302
7.1371
7.1464
7.2450
7.2589
7.2683
7.2755
7.2968
7.3230
7.3444
7.3549
7.3641
7.3827
7.3995
7.4690
7.4725
7.4893
7.4936
7.5491
7.5530
7.5708
7.5883
7.7027
1.0306
1.0287
1.0
2.0035
3.221
11.9442
3.0516
1.0273
0.9926
ppm
0
1
2
3
4
5
6
7
8
9

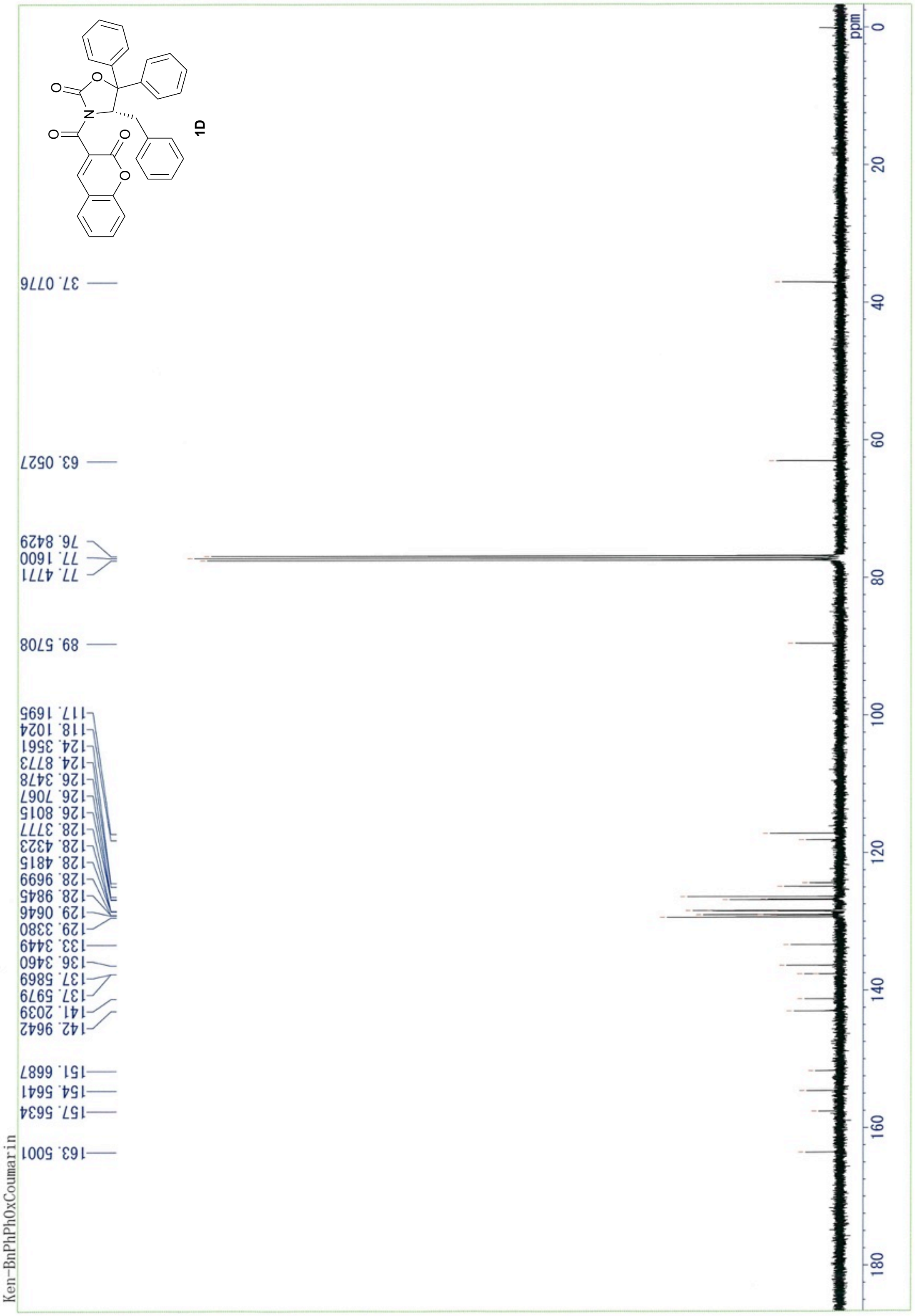
Ken-BnPhPhOxCoumarin
1D
37.0776
63.0527
76.8429
77.1600
77.4771
89.5708
117.1695
118.1024
124.3561
124.8773
126.3478
126.7067
126.8015
128.3777
128.4323
128.4815
128.9699
128.9845
129.0646
129.3380
133.3449
136.3460
137.5869
137.5979
141.2039
142.9642
151.6687
154.5641
157.5634
163.5001
ppm
0
20
40
60
80
100
120
140
160
180

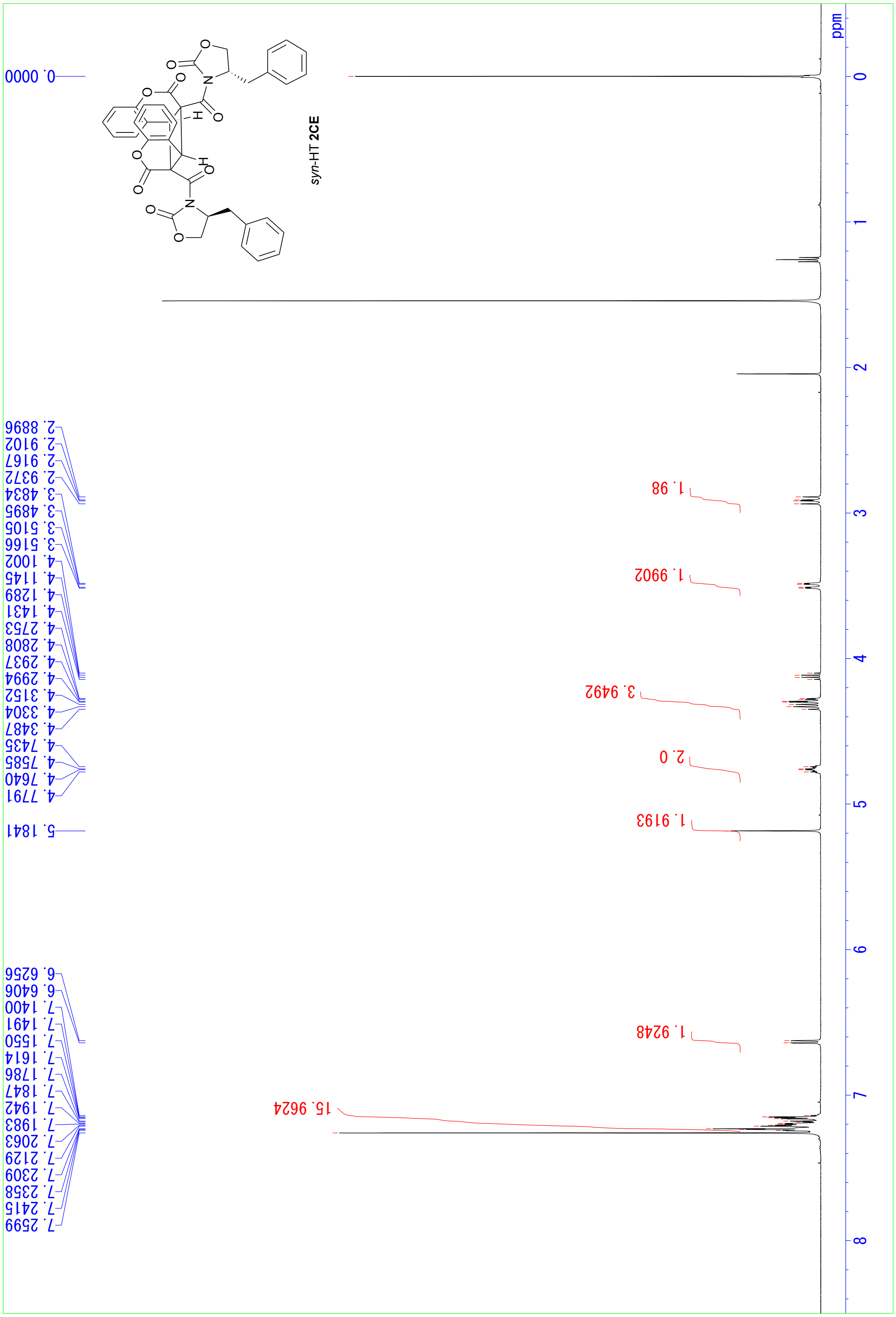
syn-HT 2CE
ppm
0.0000
2.8896
2.9102
2.9167
2.9372
3.4834
3.4895
3.5105
3.5166
4.1002
4.1145
4.1289
4.1431
4.2753
4.2808
4.2937
4.2994
4.3152
4.3304
4.3487
4.7435
4.7585
4.7640
4.7791
5.1841
6.6256
6.6406
7.1400
7.1491
7.1550
7.1614
7.1786
7.1847
7.1942
7.1983
7.2063
7.2129
7.2309
7.2358
7.2415
7.2599
1.98
1.9902
3.9492
2.0
1.9193
1.9248
15.9624

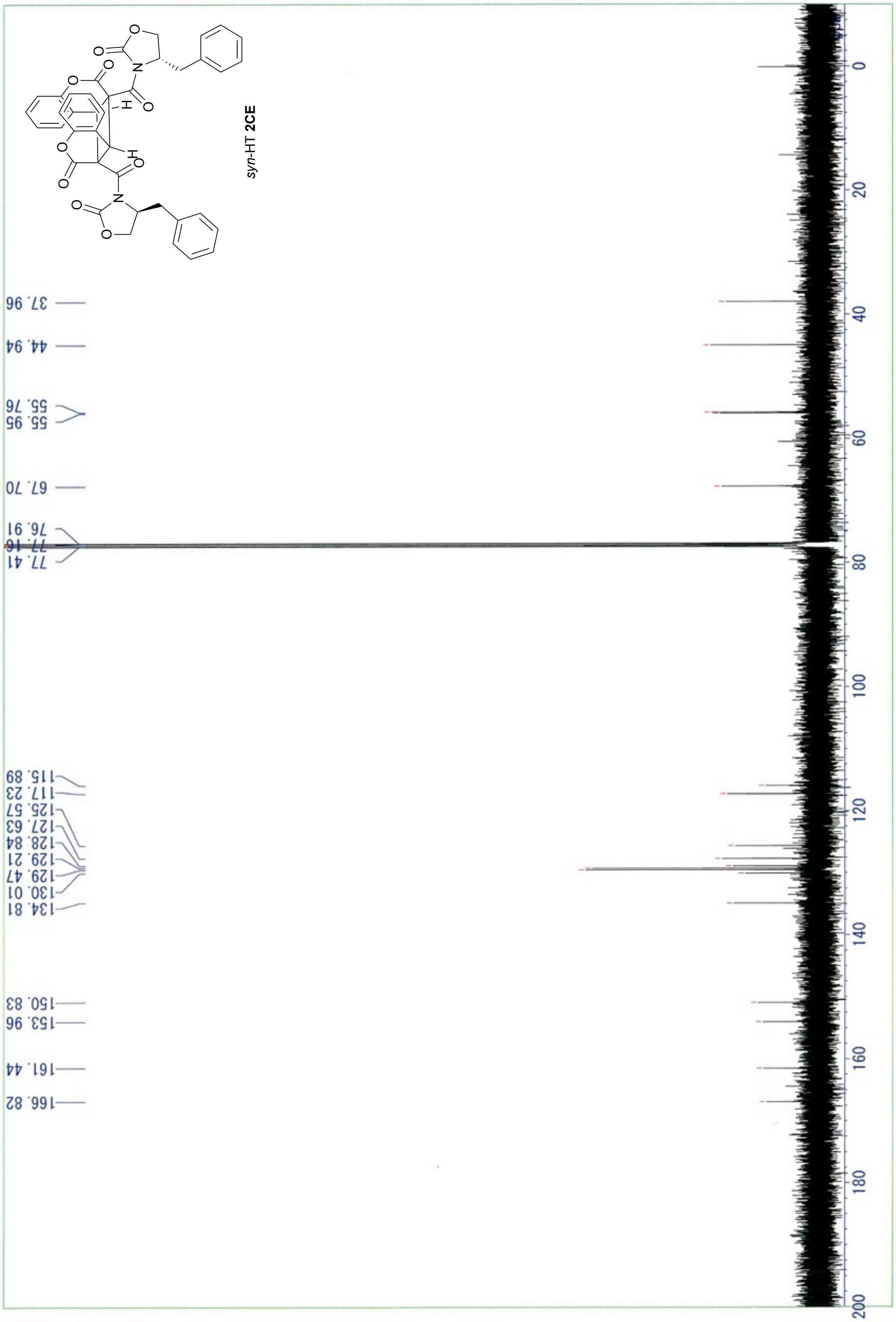

syn-HT 2CE
37.96
44.94
55.76
55.95
67.70
76.91
77.16
77.41
115.89
117.23
125.57
127.63
128.84
129.21
129.47
130.01
134.81
150.83
153.96
161.44
166.82
0
20
40
60
80
100
120
140
160
180
200

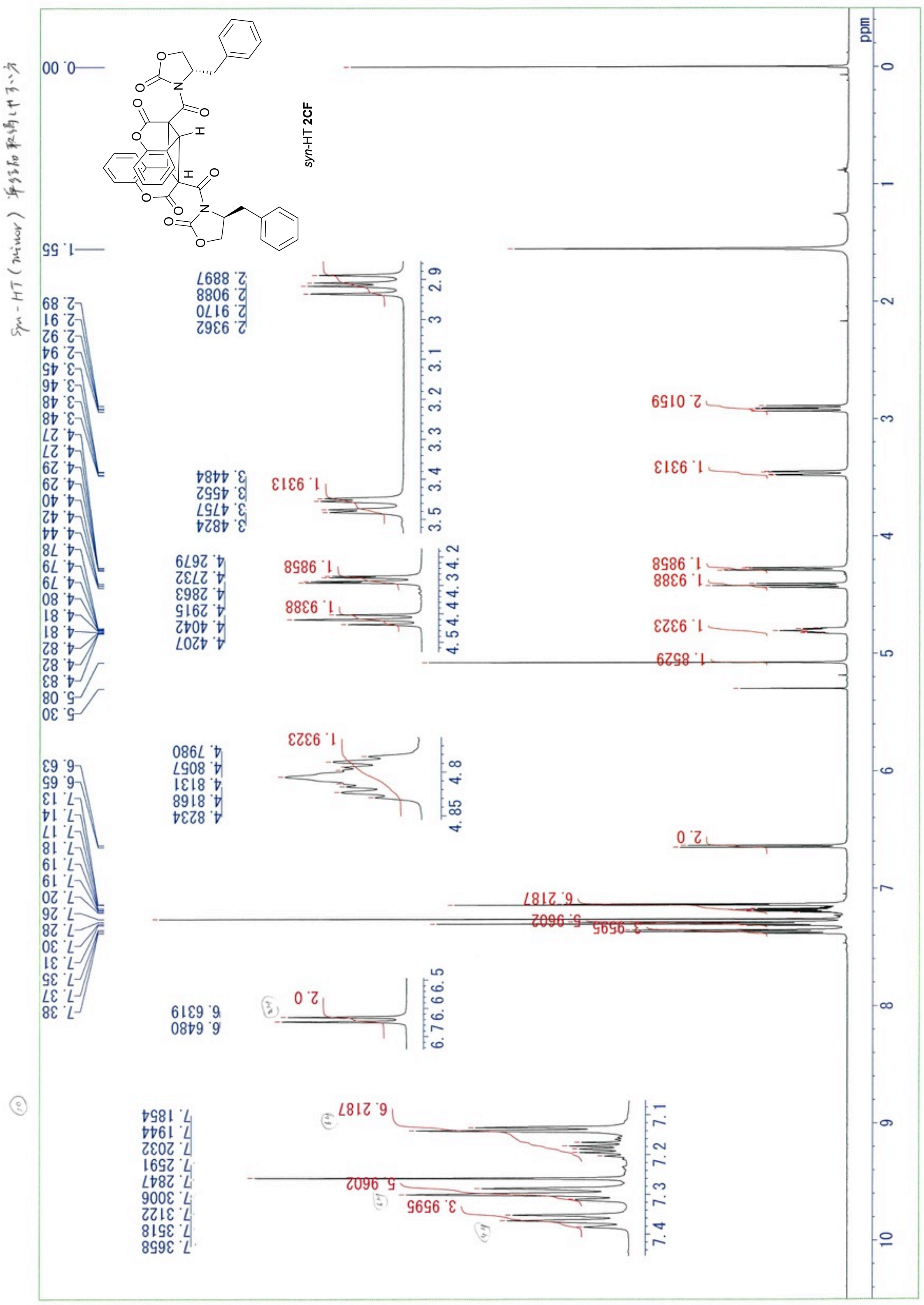
syn-HT 2CF

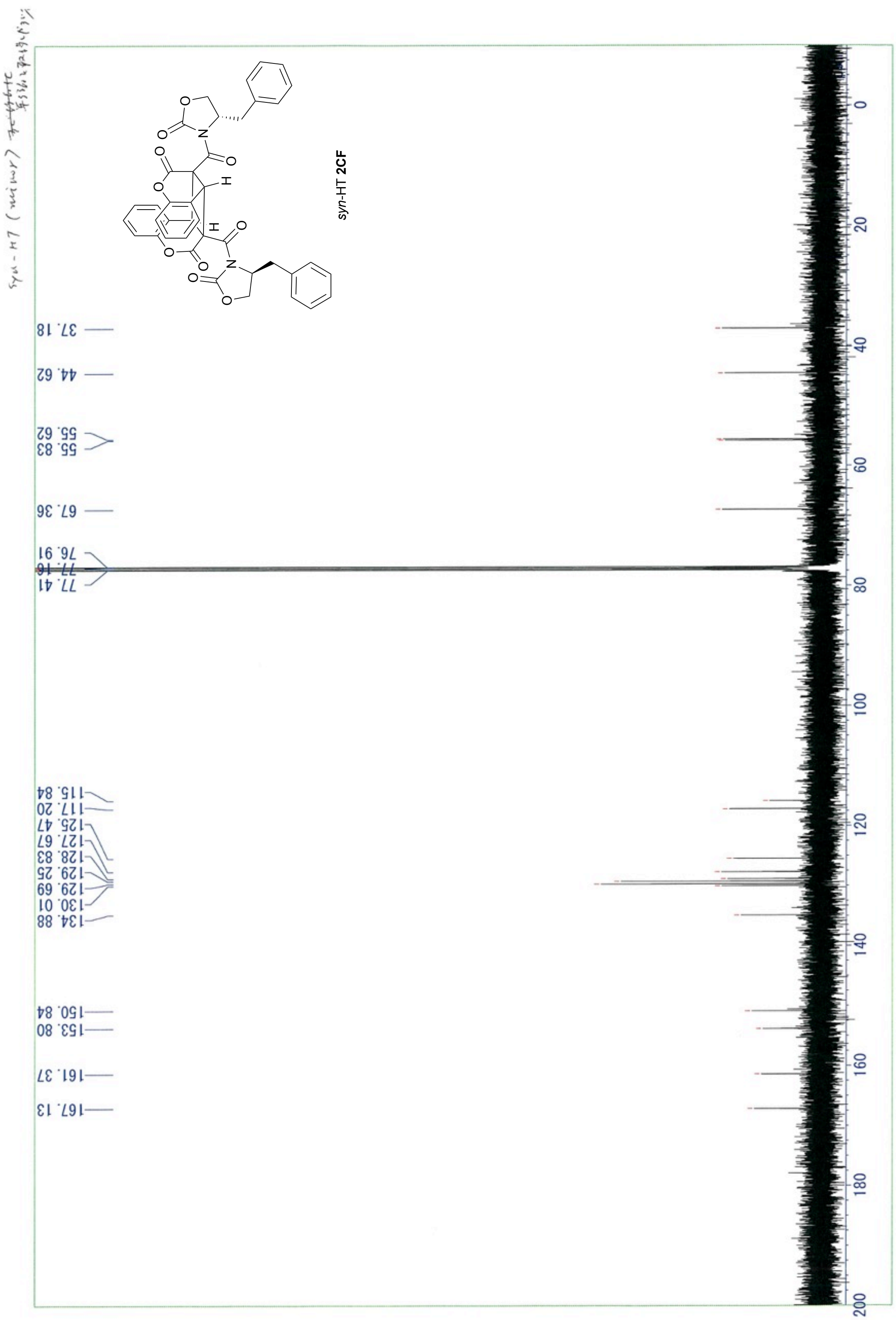

syn-HT 2CF
37.18
44.62
55.62
55.83
67.36
76.91
77.16
77.41
115.84
117.20
125.47
127.67
128.83
129.25
129.69
130.01
134.88
150.84
153.80
161.37
167.13
0
20
40
60
80
100
120
140
160
180
200

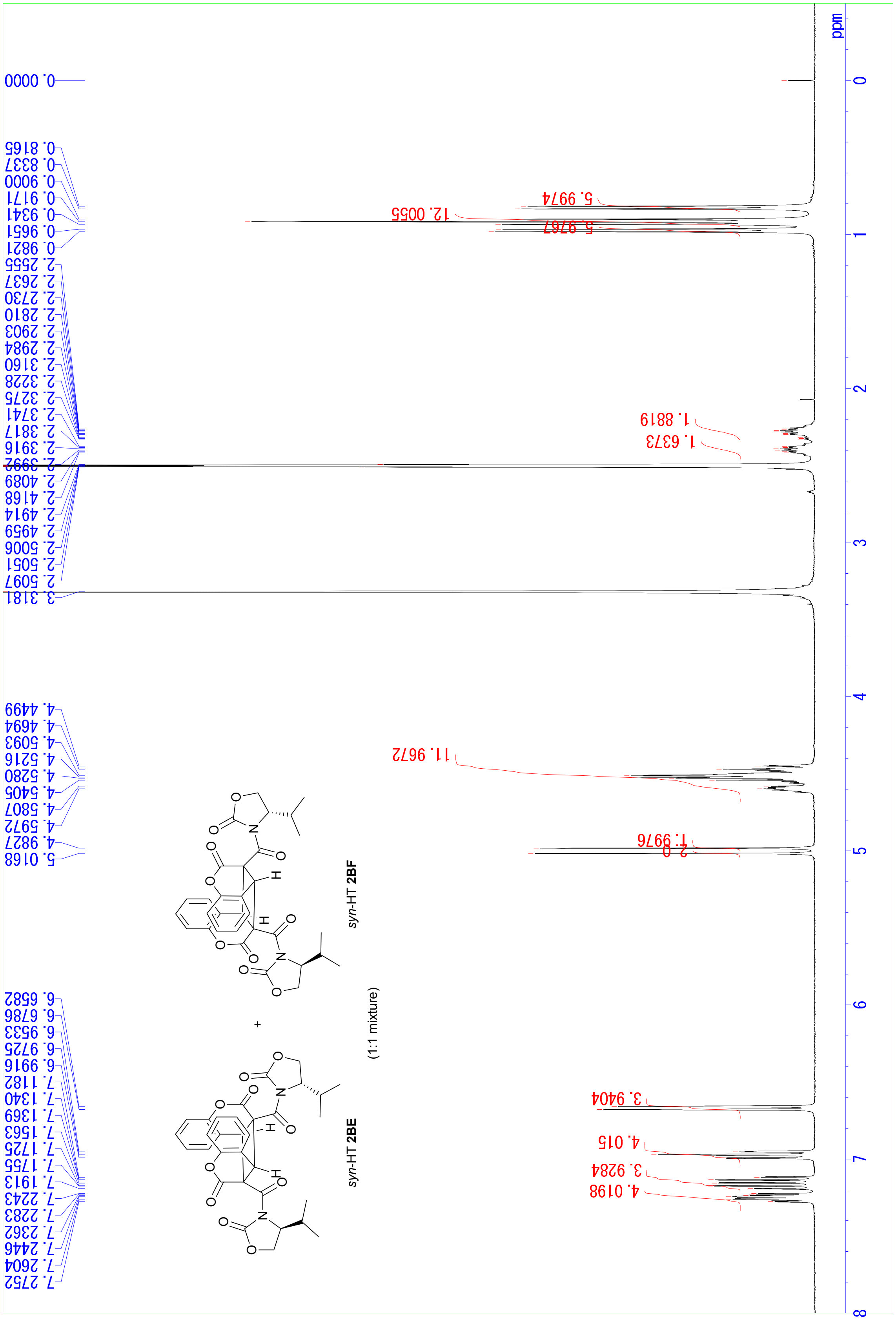
syn-HT 2BE
syn-HT 2BF
(1:1 mixture)
ppm

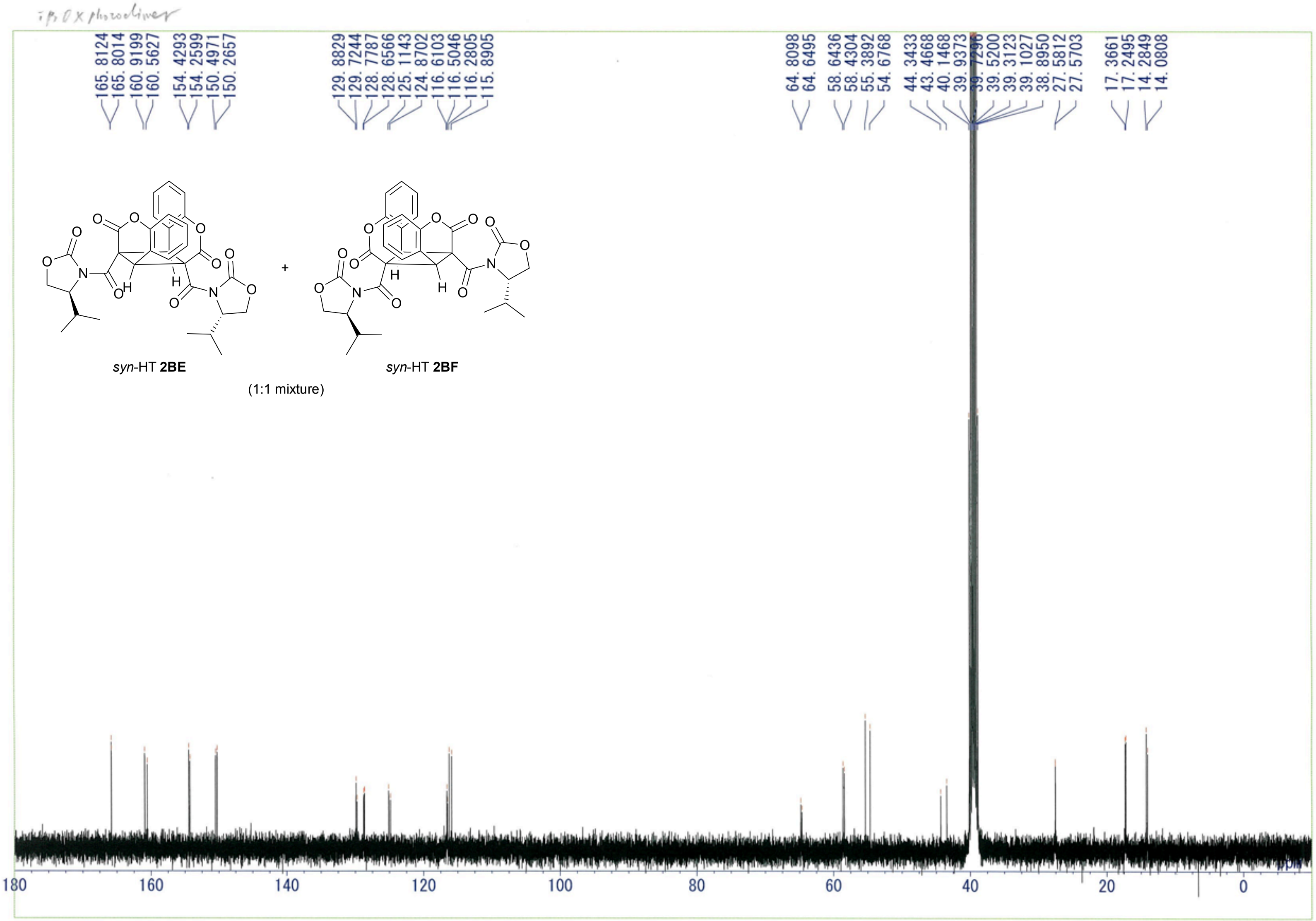
iPrOX photo driver
165.8124
165.8014
160.9199
160.5627
154.4293
154.2599
150.4971
150.2657
129.8829
129.7244
128.7787
128.6566
125.1143
124.8702
116.6103
116.5046
116.2805
115.8905
64.8098
64.6495
58.6436
58.4304
55.3892
54.6768
44.3433
43.4668
40.1468
39.9373
39.7296
39.5200
39.3123
39.1027
38.8950
27.5812
27.5703
17.3661
17.2495
14.2849
14.0808
syn-HT 2BE
syn-HT 2BF
(1:1 mixture)
180
160
140
120
100
80
60
40
20
0
ppm

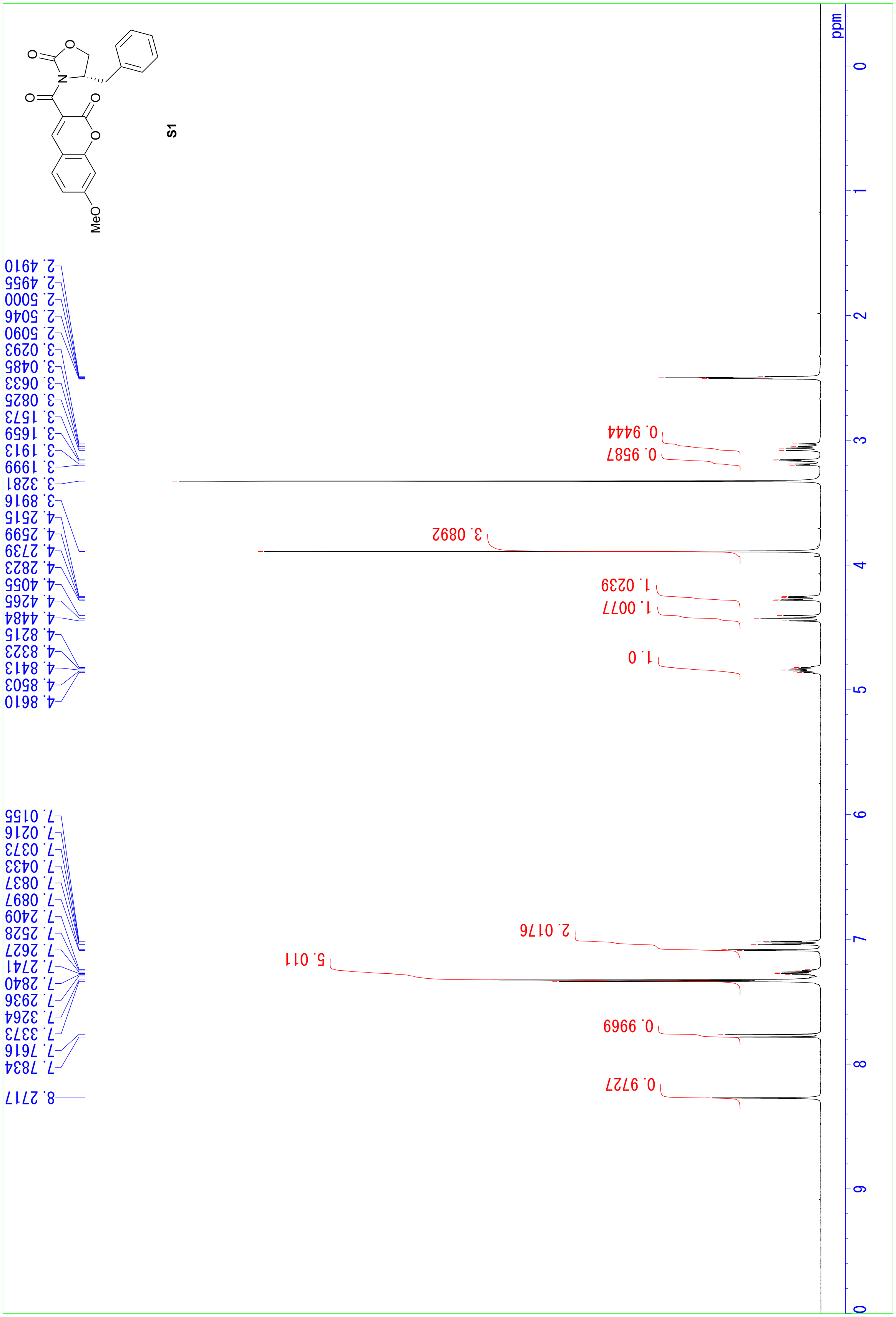
S1
MeO
ppm
2.4910
2.4955
2.5000
2.5046
2.5090
3.0293
3.0485
3.0633
3.0825
3.1573
3.1659
3.1913
3.1999
3.3281
3.8916
4.2515
4.2599
4.2739
4.2823
4.4055
4.4265
4.4484
4.8215
4.8323
4.8413
4.8503
4.8610
7.0155
7.0216
7.0373
7.0433
7.0837
7.0897
7.2409
7.2528
7.2627
7.2741
7.2840
7.2936
7.3264
7.3373
7.7616
7.7834
8.2717
0.9444
0.9587
3.0892
1.0239
1.0077
1.0
2.0176
5.011
0.9969
0.9727

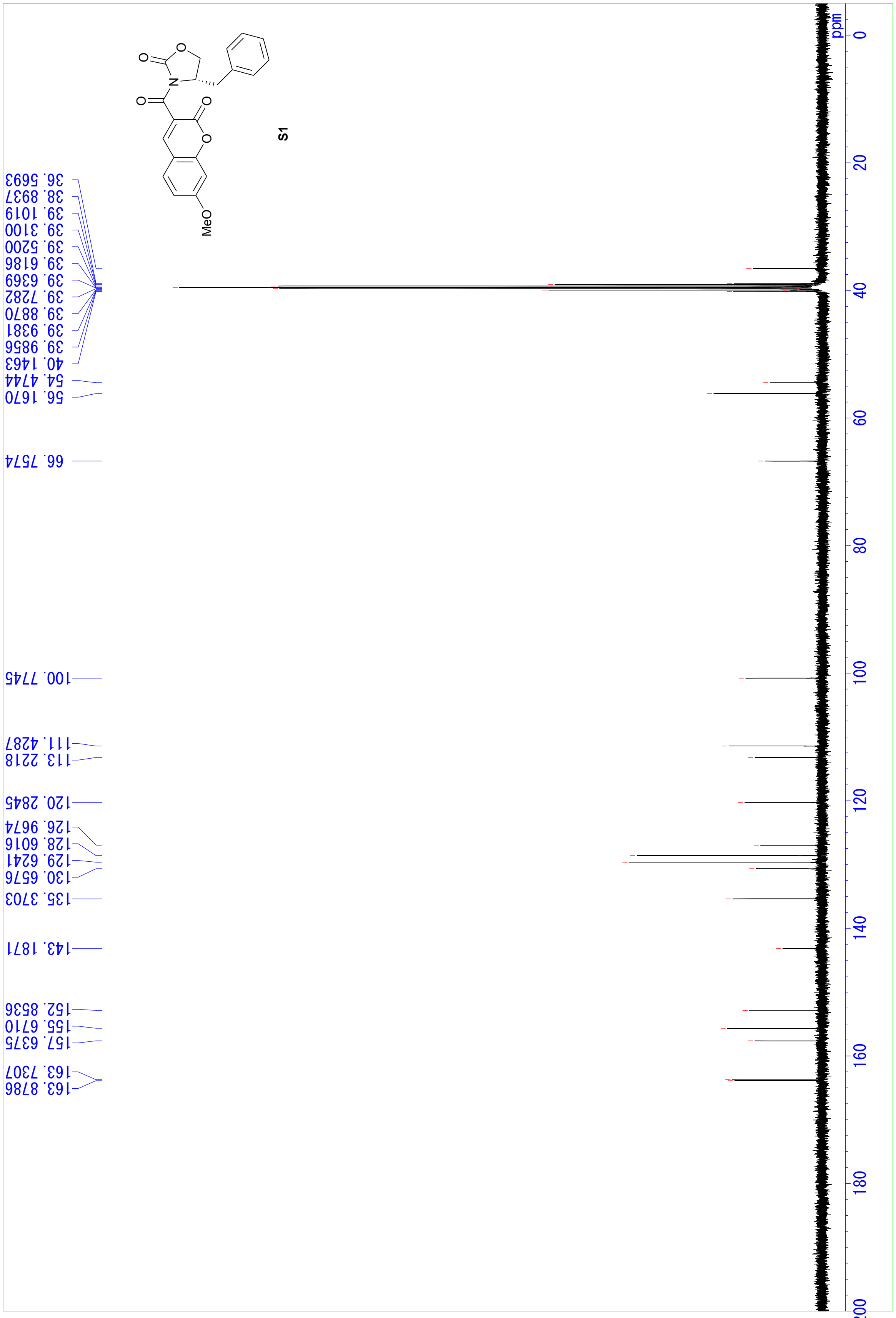
36.5693
38.8937
39.1019
39.3100
39.5200
39.6186
39.6369
39.7282
39.8870
39.9381
39.9856
40.1463
54.4744
56.1670
66.7574
100.7745
111.4287
113.2218
120.2845
126.9674
128.6016
129.6241
130.6576
135.3703
143.1871
152.8536
155.6710
157.6375
163.7307
163.8786
MeO
S1
ppm
0
20
40
60
80
100
120
140
160
180
200

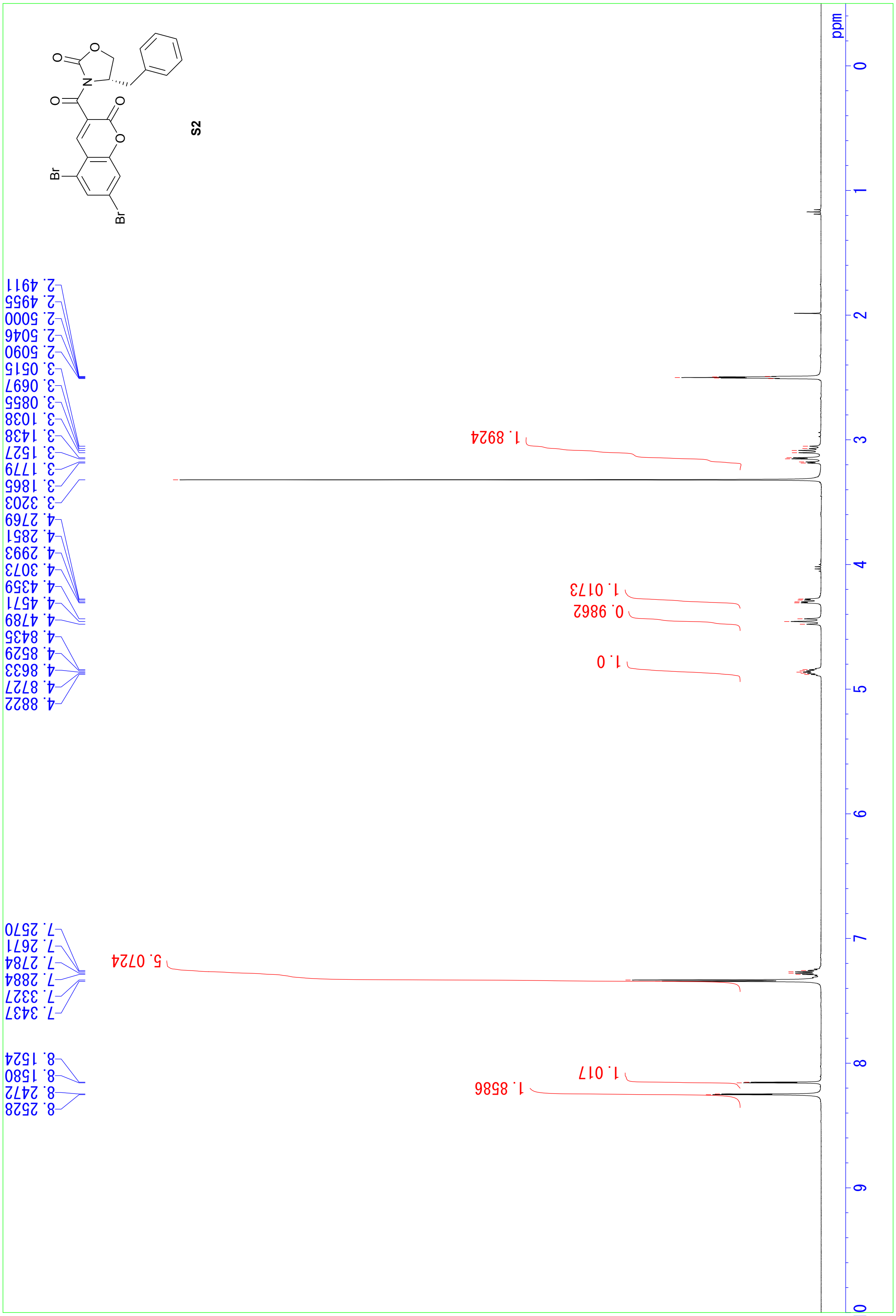
S2
Br
Br
O
O
O
N
O
O
2.4911
2.4955
2.5000
2.5046
2.5090
3.0515
3.0697
3.0855
3.1038
3.1438
3.1527
3.1779
3.1865
3.3203
4.2769
4.2851
4.2993
4.3073
4.4359
4.4571
4.4789
4.8435
4.8529
4.8633
4.8727
4.8822
7.2570
7.2671
7.2784
7.2884
7.3327
7.3437
8.1524
8.1580
8.2472
8.2528
1.8924
1.0173
0.9862
1.0
5.0724
1.017
1.8586
ppm
0
1
2
3
4
5
6
7
8
9
10

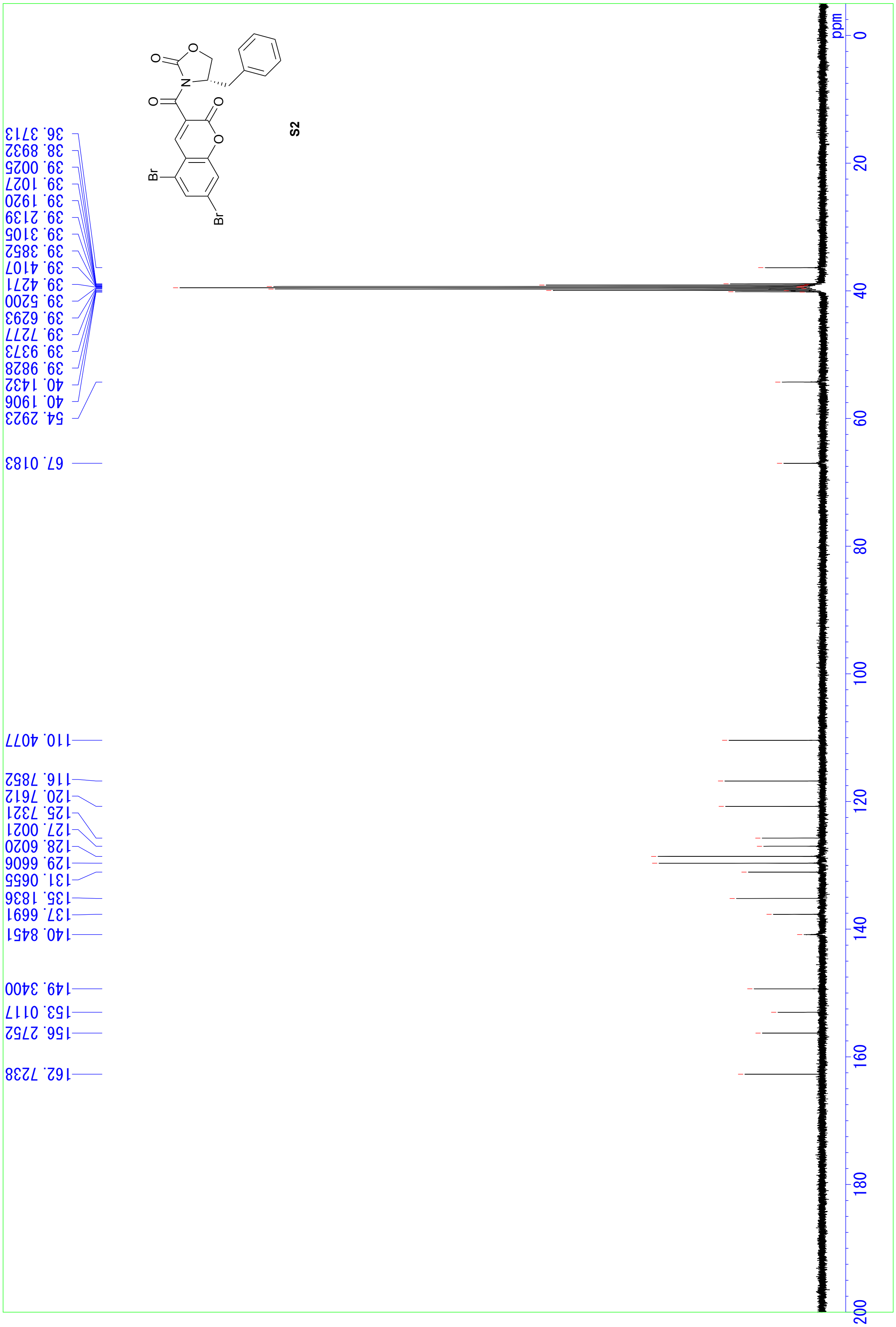
36.3713
38.8932
39.0025
39.1027
39.1920
39.2139
39.3105
39.3852
39.4107
39.4271
39.5200
39.6293
39.7277
39.9373
39.9828
40.1432
40.1906
54.2923
67.0183
110.4077
116.7852
120.7612
125.7321
127.0021
128.6020
129.6606
131.0655
135.1836
137.6691
140.8451
149.3400
153.0117
156.2752
162.7238
S2
ppm

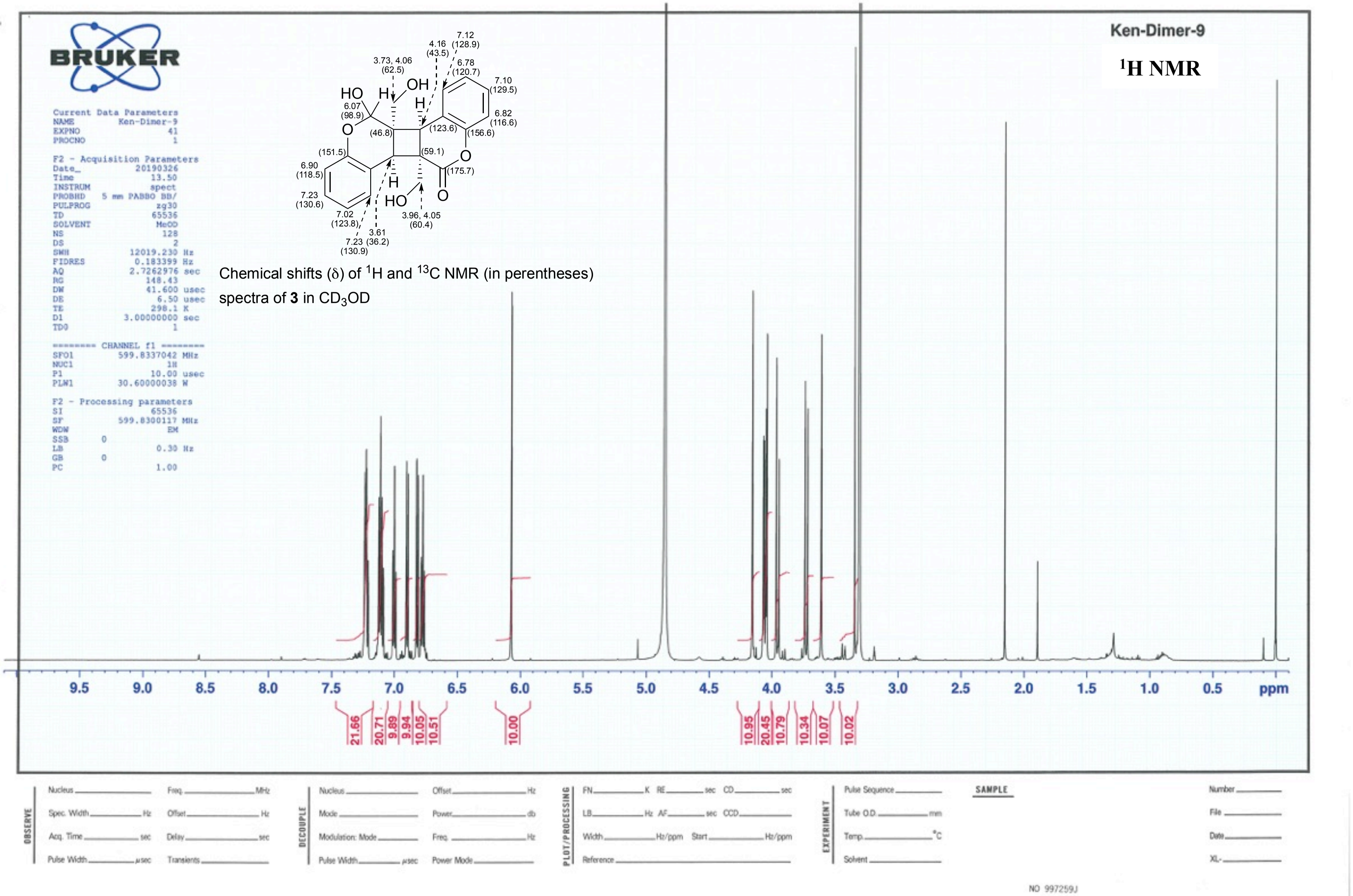
Ken-Dimer-9
^{1}H NMR
BRUKER
Current Data Parameters
NAME Ken-Dimer-9
EXPNO 41
PROCNO 1
F2 - Acquisition Parameters
Date_ 20190326
Time 13.50
INSTRUM spect
PROBHD 5 mm PABBO BB/
PULPROG zg30
TD 65536
SOLVENT MeOD
NS 128
DS 2
SWH 12019.230 Hz
FIDRES 0.183399 Hz
AQ 2.7262976 sec
RG 148.43
DW 41.600 usec
DE 6.50 usec
TE 298.1 K
D1 3.00000000 sec
TD0 1
======== CHANNEL f1 ========
SFO1 599.8337042 MHz
NUC1 1H
P1 10.00 usec
PLW1 30.60000038 W
F2 - Processing parameters
SI 65536
SF 599.8300117 MHz
WDW EM
SSB 0
LB 0.30 Hz
GB 0
PC 1.00
Chemical shifts (δ) of ^{1}H and ^{13}C NMR (in perentheses) spectra of **3** in CD_3OD
ppm
21.66
20.71
9.89
9.94
10.05
10.51
10.00
10.95
20.45
10.79
10.34
10.07
10.02
NO 997259J

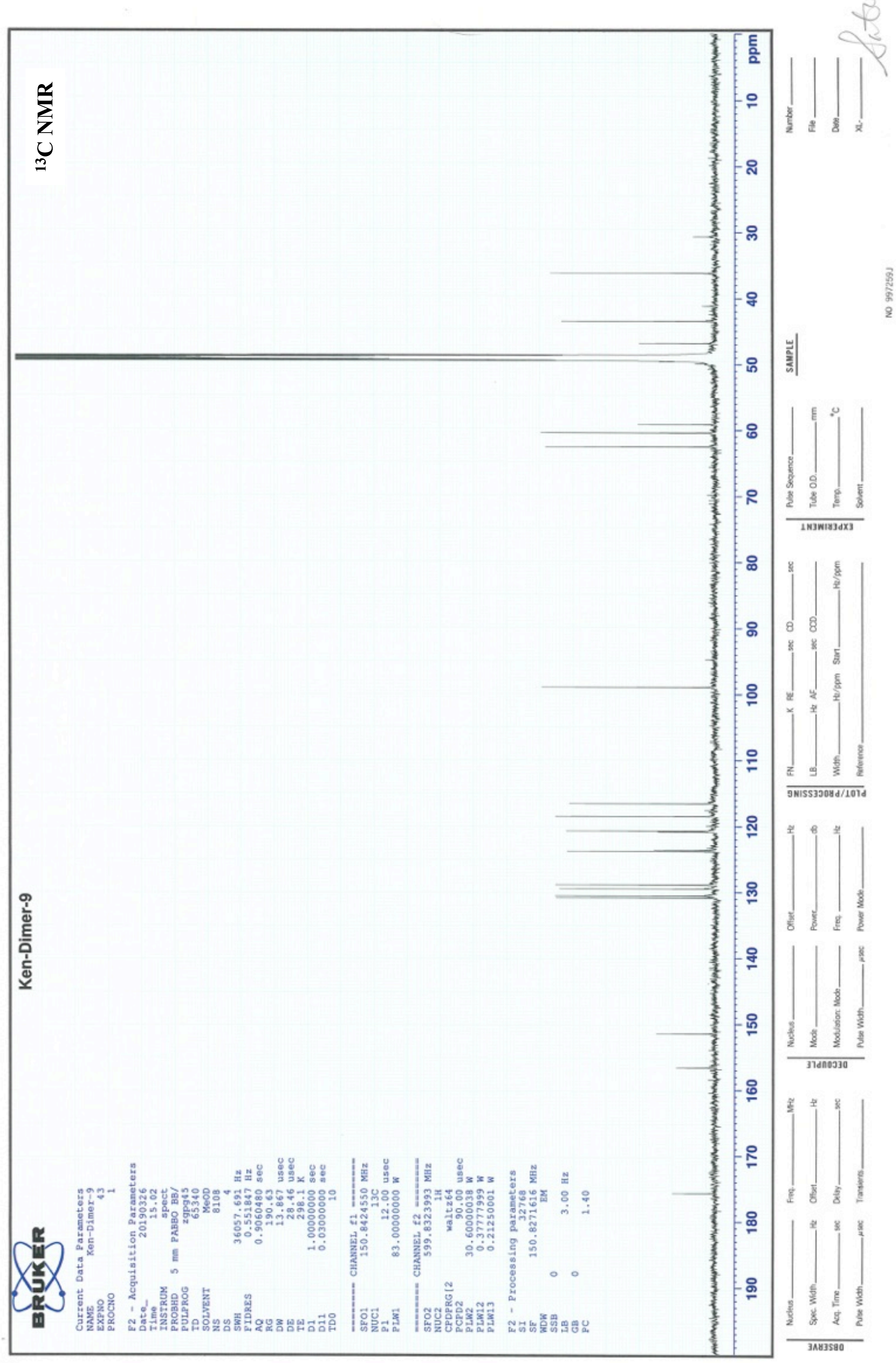
Ken-Dimer-9
13C NMR
Current Data Parameters
NAME Ken-Dimer-9
EXPNO 43
PROCNO 1
F2 - Acquisition Parameters
Date_ 20190326
Time 15.02
INSTRUM spect
PROBHD 5 mm PABBO BB/
PULPROG zgpg45
TD 65340
SOLVENT MeOD
NS 8108
DS 4
SWH 36057.691 Hz
FIDRES 0.551847 Hz
AQ 0.9060480 sec
RG 190.63
DW 13.867 usec
DE 28.46 usec
TE 298.1 K
D1 1.00000000 sec
D11 0.03000000 sec
TD0 10
CHANNEL f1
SFO1 150.8424550 MHz
NUC1 13C
P1 12.00 usec
PLW1 83.00000000 W
CHANNEL f2
SFO2 599.8323993 MHz
NUC2 1H
CPDPRG[2 waltz64
PCPD2 90.00 usec
PLW2 30.60000038 W
PLW12 0.37777999 W
PLW13 0.21250001 W
F2 - Processing parameters
SI 32768
SF 150.8271616 MHz
WDW EM
SSB 0
LB 3.00 Hz
GB 0
PC 1.40
190 180 170 160 150 140 130 120 110 100 90 80 70 60 50 40 30 20 10 ppm

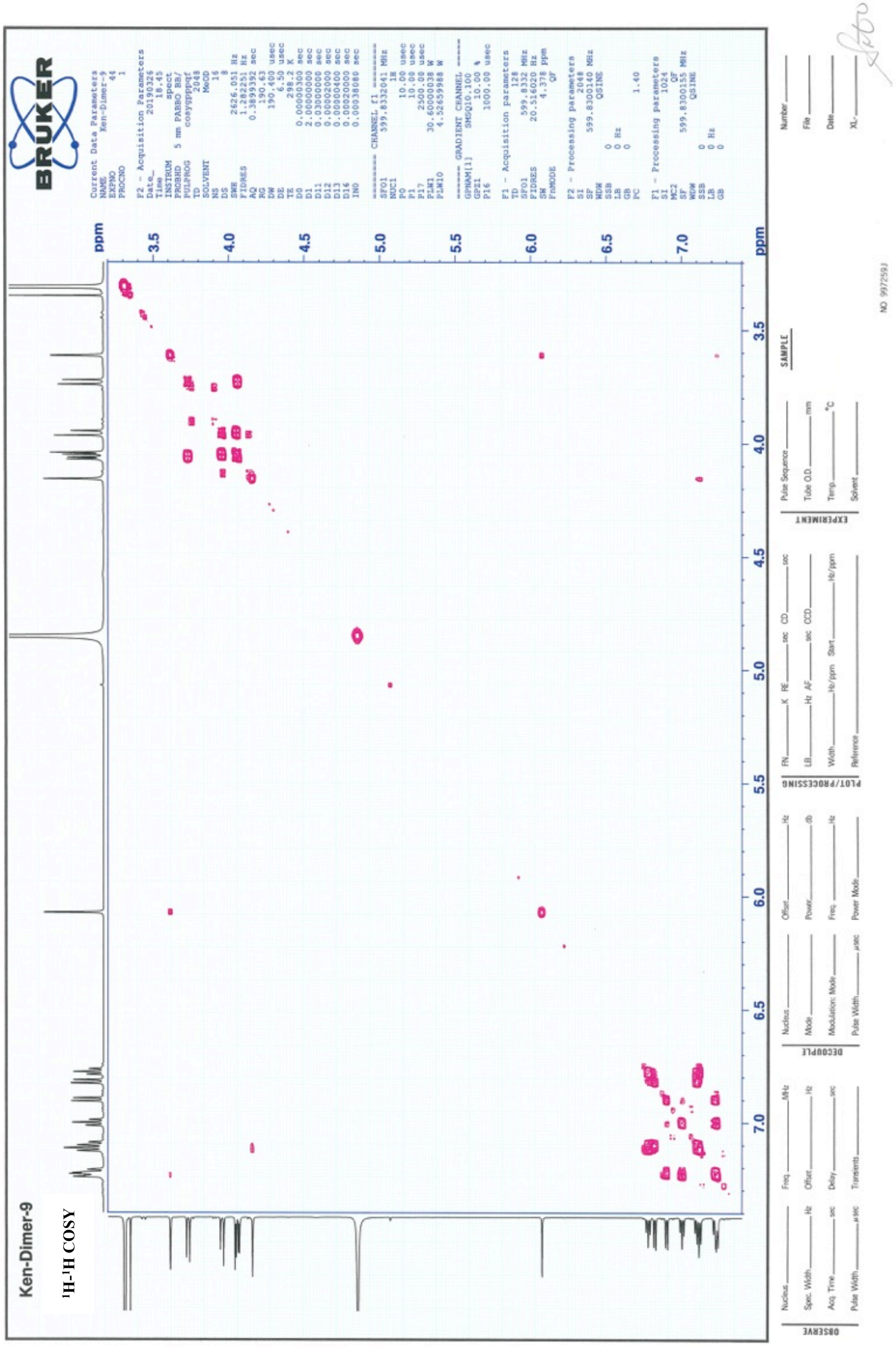
Ken-Dimer-9
1H-1H COSY
BRUKER

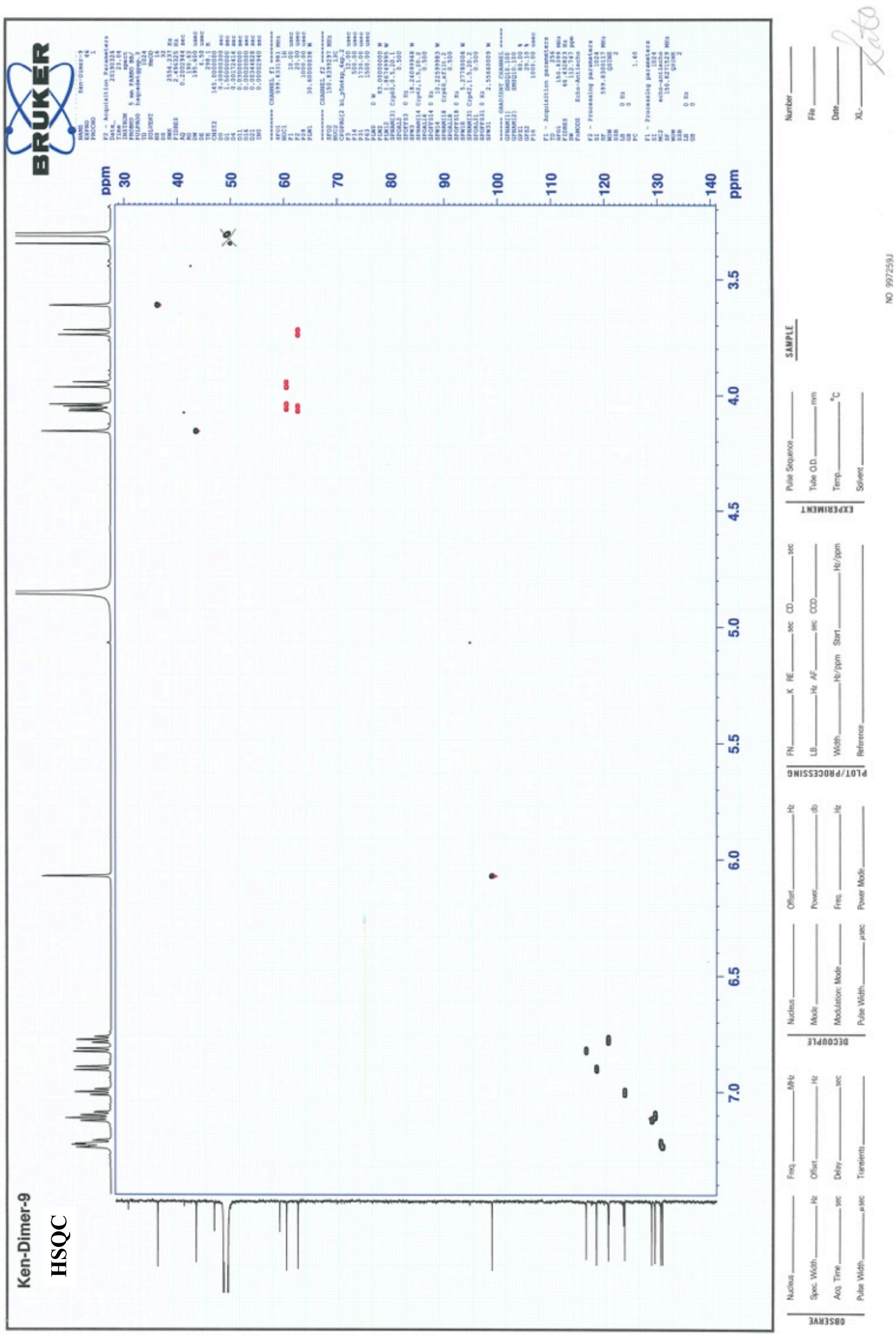
Ken-Dimer-9
HSQC
BRUKER
ppm
SAMPLE
EXPERIMENT
PLOT/PROCESSING
DECOUPLE
OBSERVE

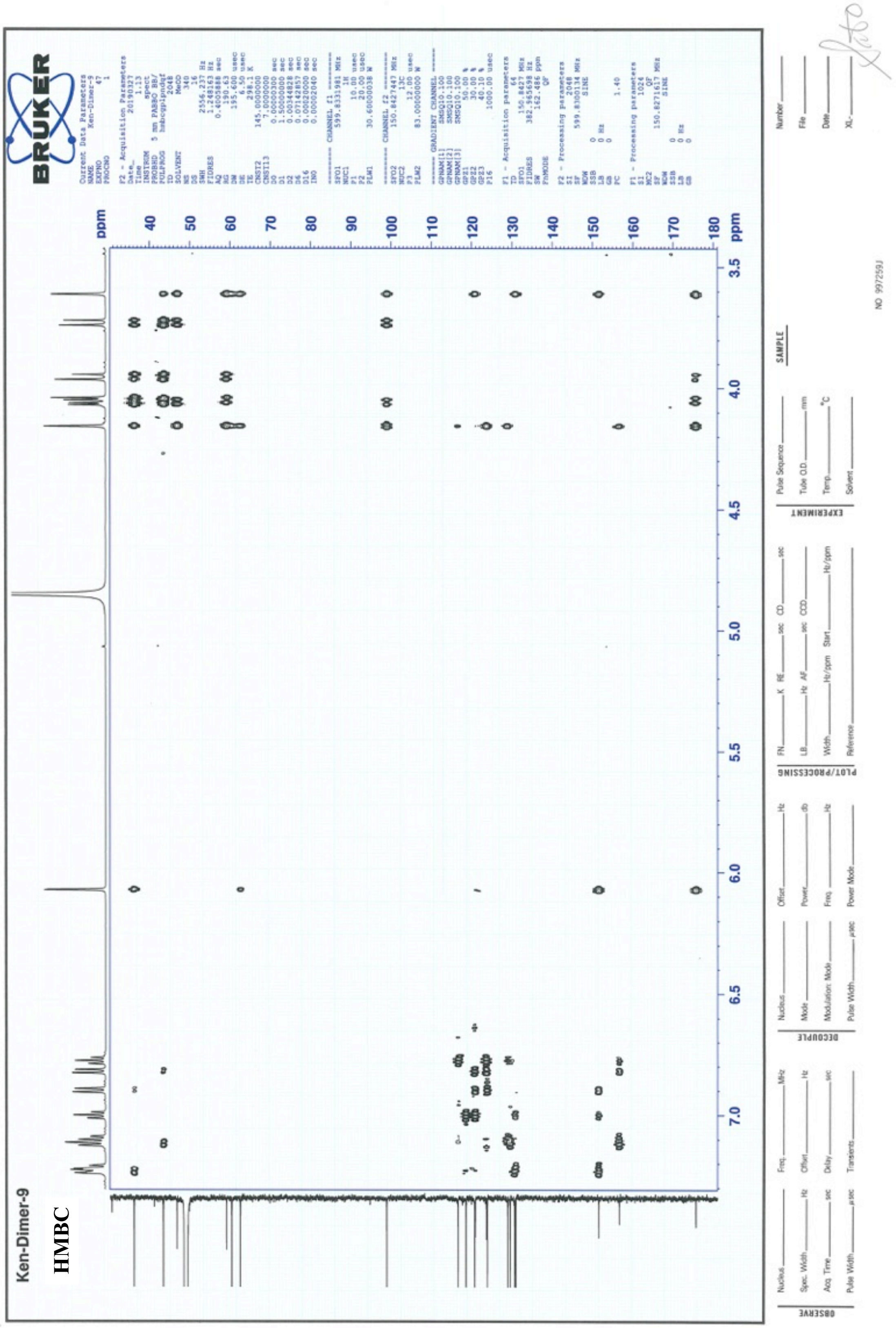
Ken-Dimer-9
HMBC
BRUKER

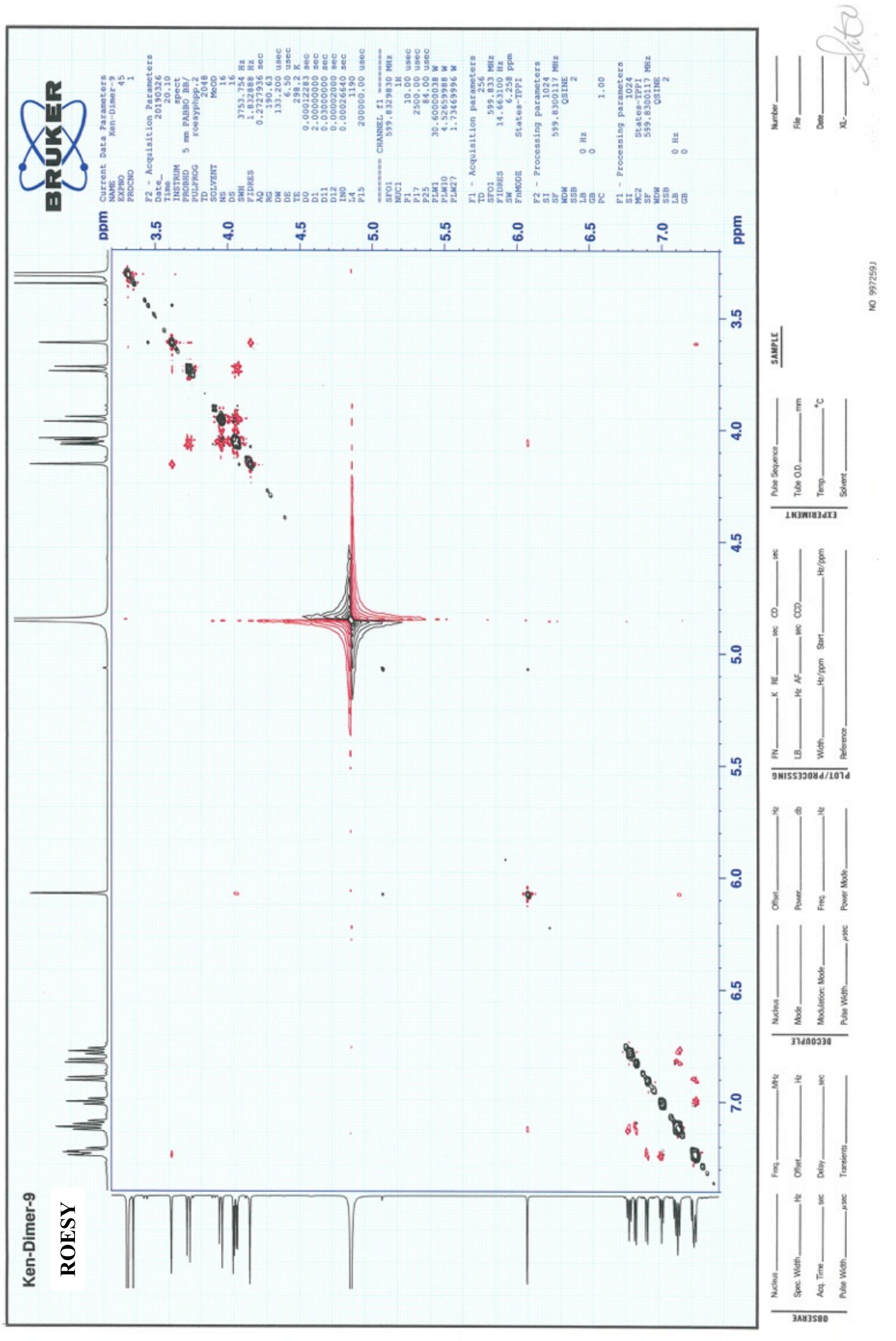
Ken-Dimer-9
ROESY
BRUKER

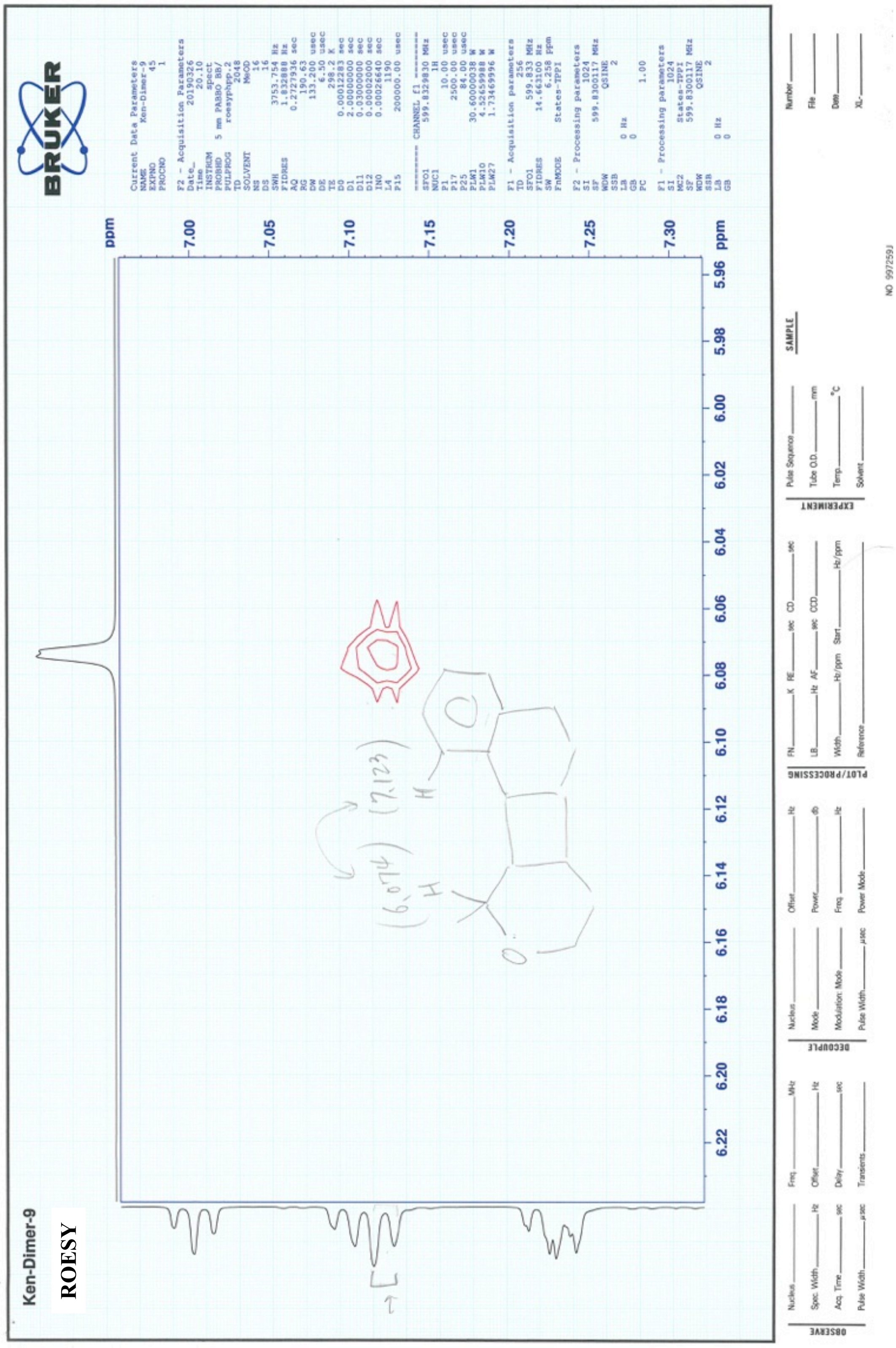

Ken-Dimer-9
ROESY
BRUKER
ppm

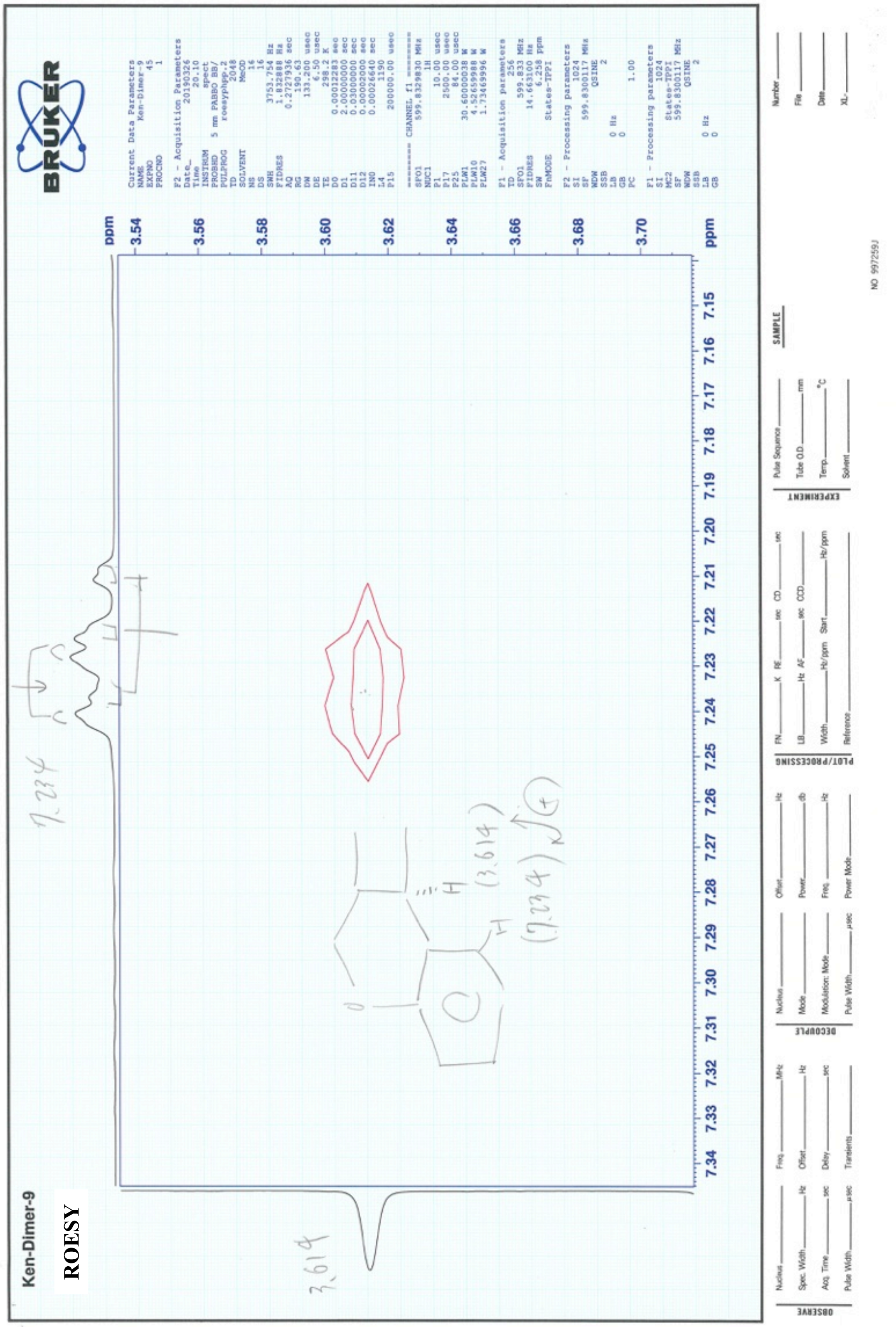
Ken-Dimer-9
ROESY
BRUKER

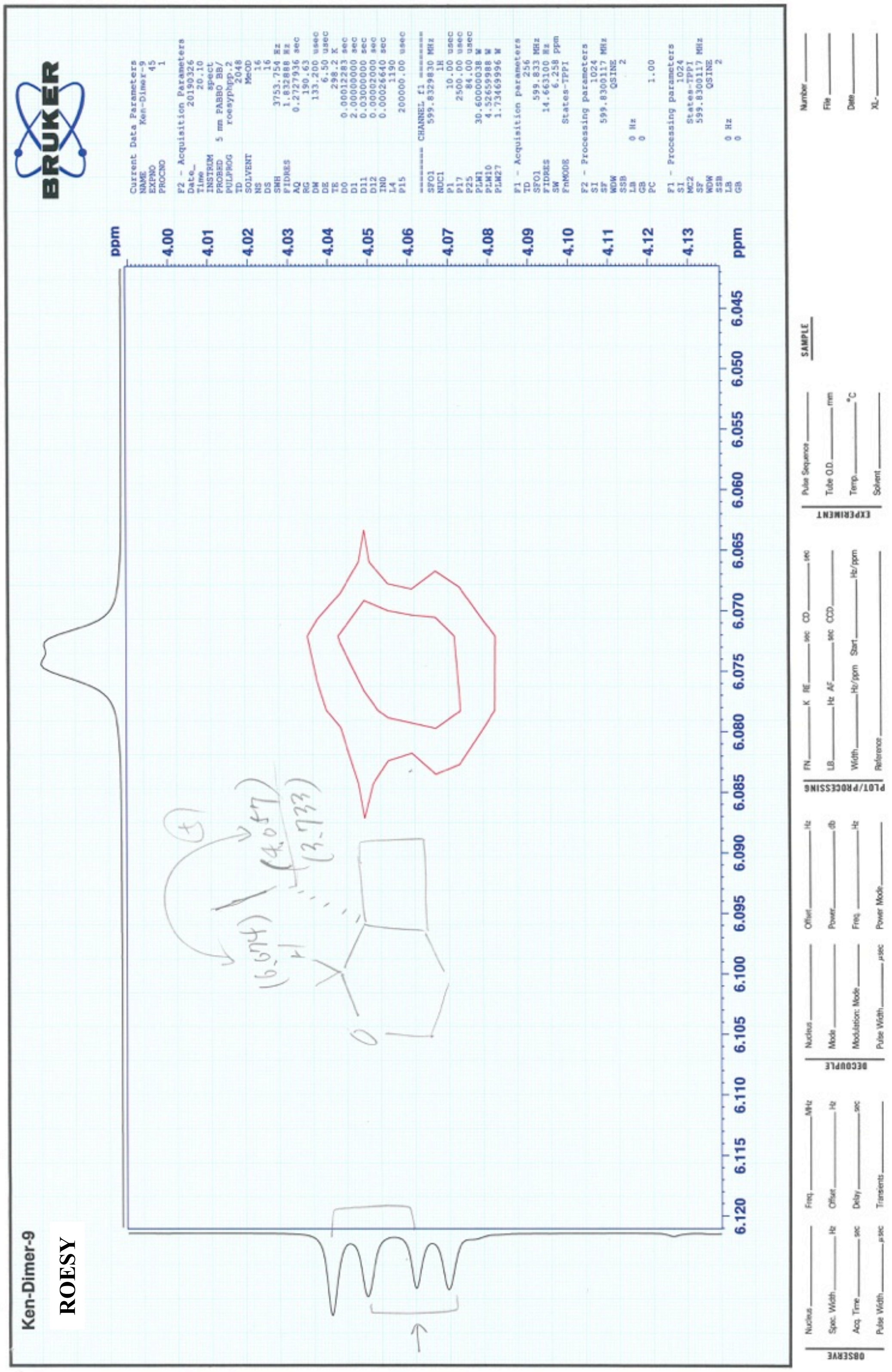
Ken-Dimer-9
ROESY

^{1}H NMR spectra of 1C and 1C-BF_3•OEt_2 in C_6D_6

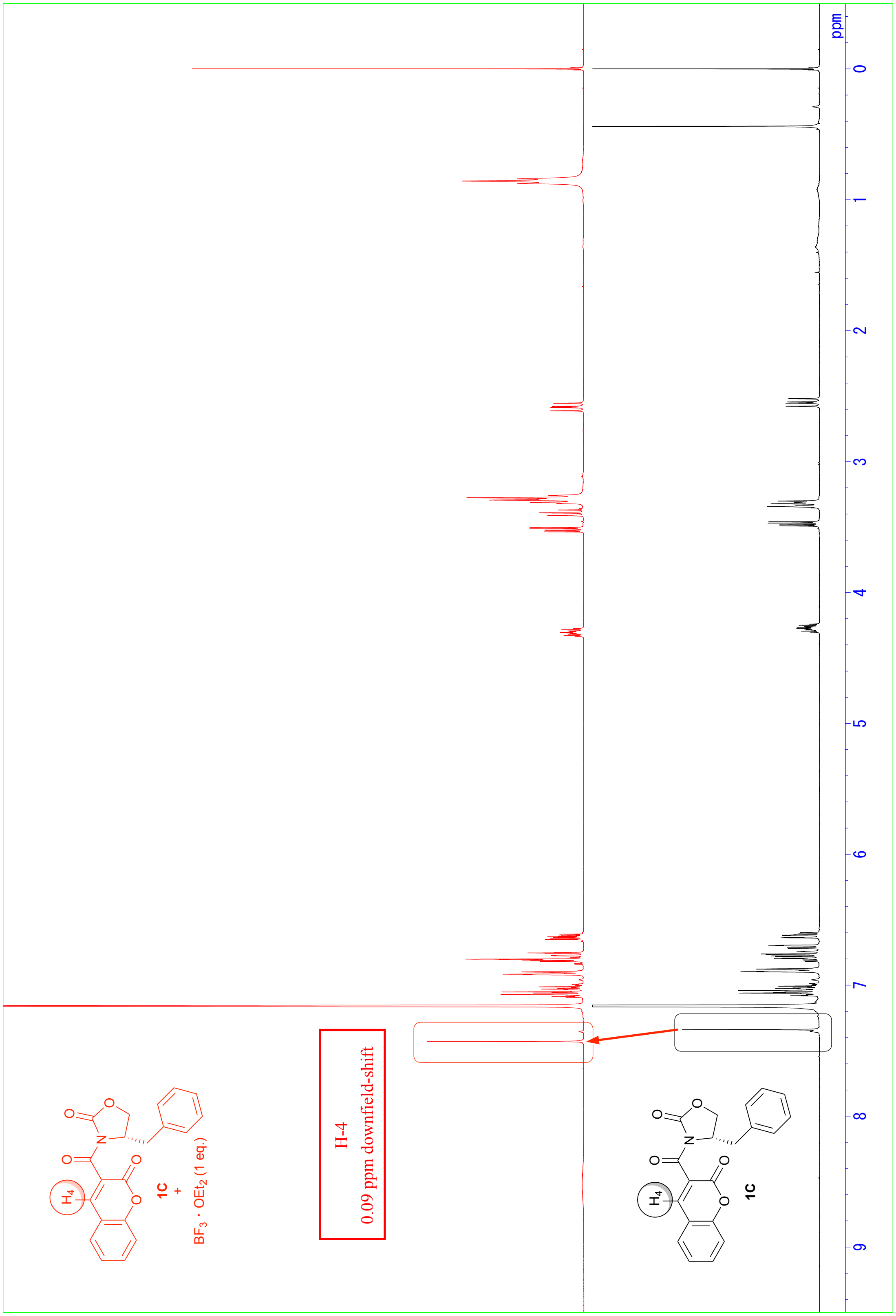

^{13}C NMR spectra of 1C and 1C-BF_3•OEt_2 in C_6D_6

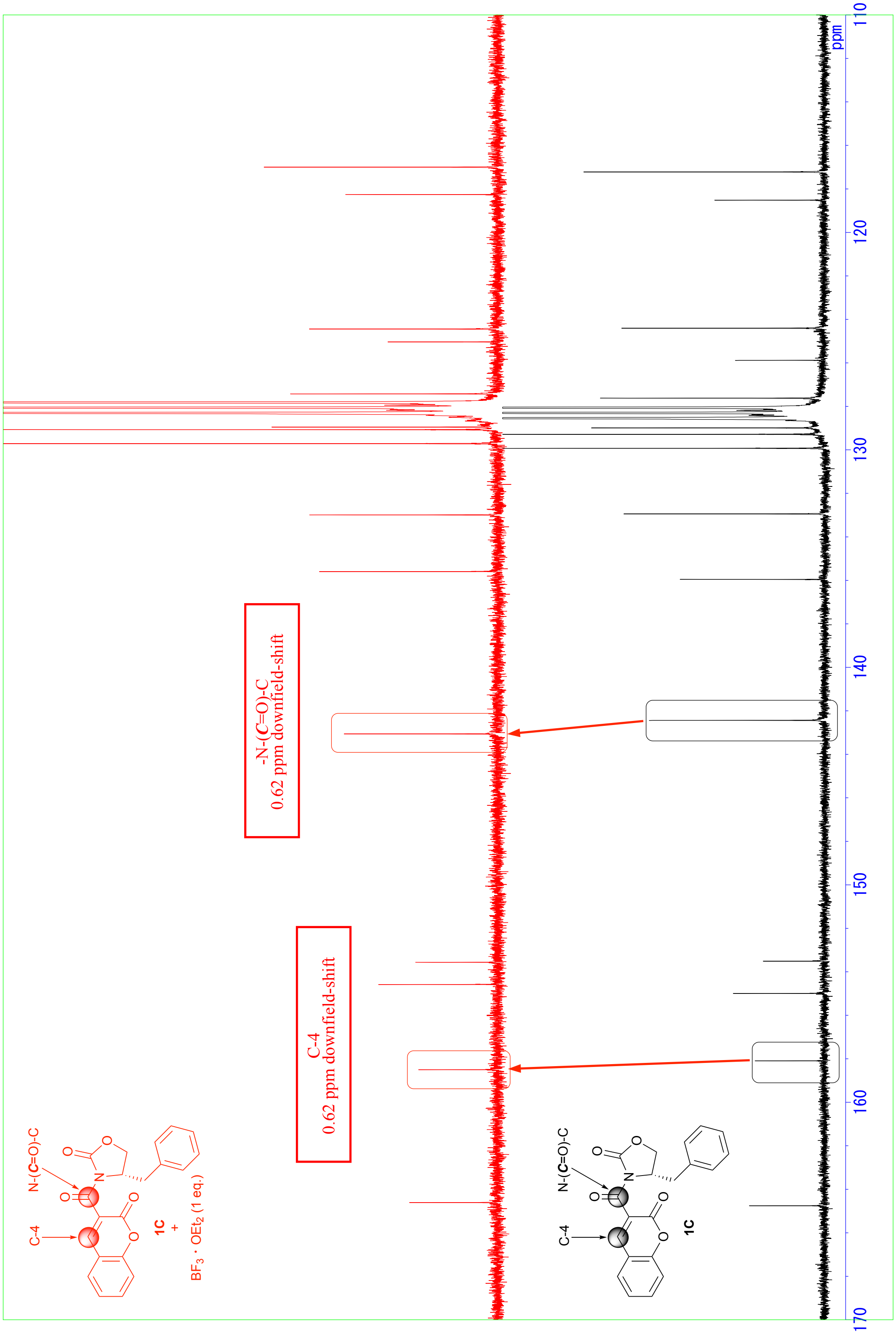

^{1}H NMR spectra of 1C and 1C-$Zn(ClO_4)_2{\bullet}6H_2O$ in $(CD_3)_2CO$

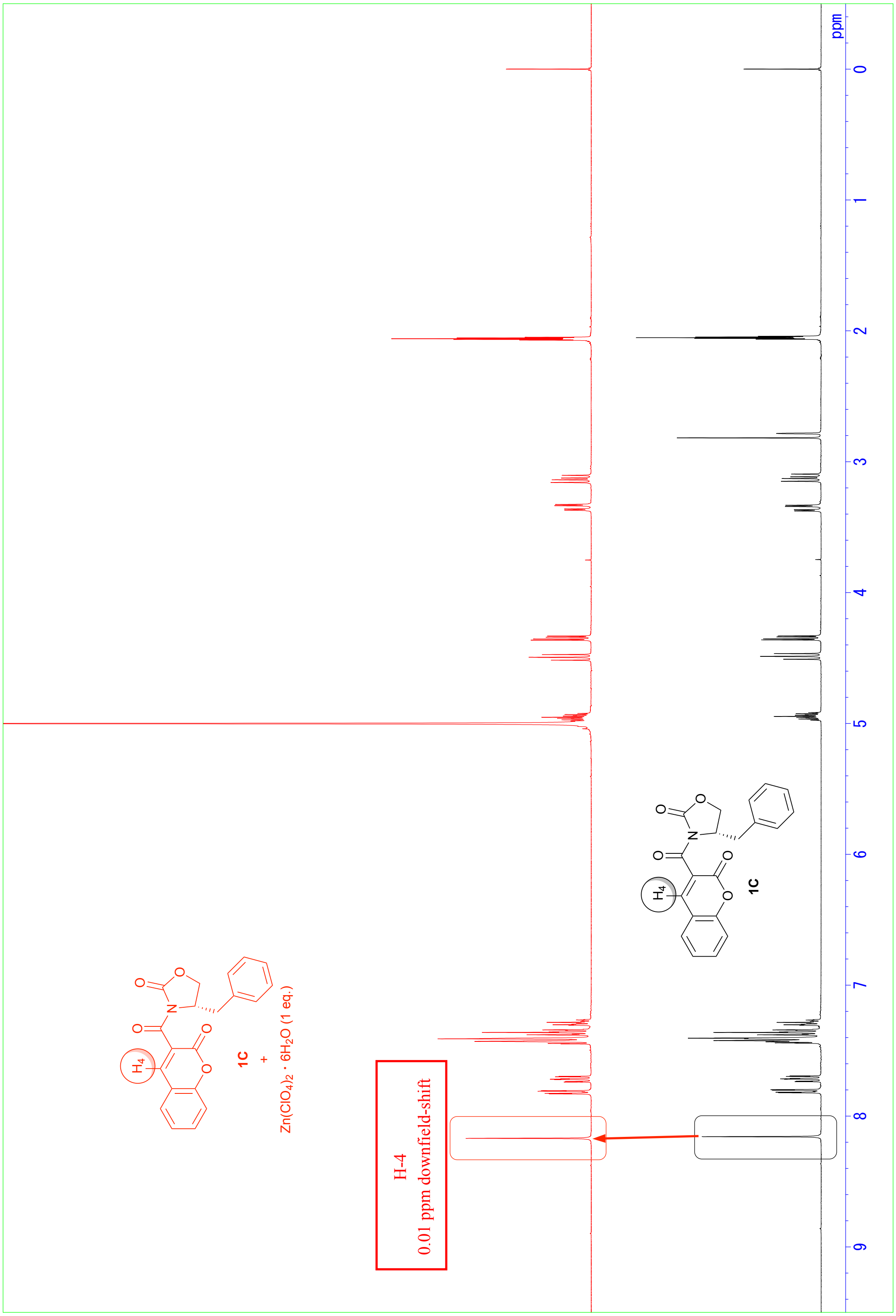

NMR spectra of 1C in C_6D_6 (^{1}H, ^{13}C, DEPT, 1D NOE, ROESY, COSY, HSQC, HMBC)

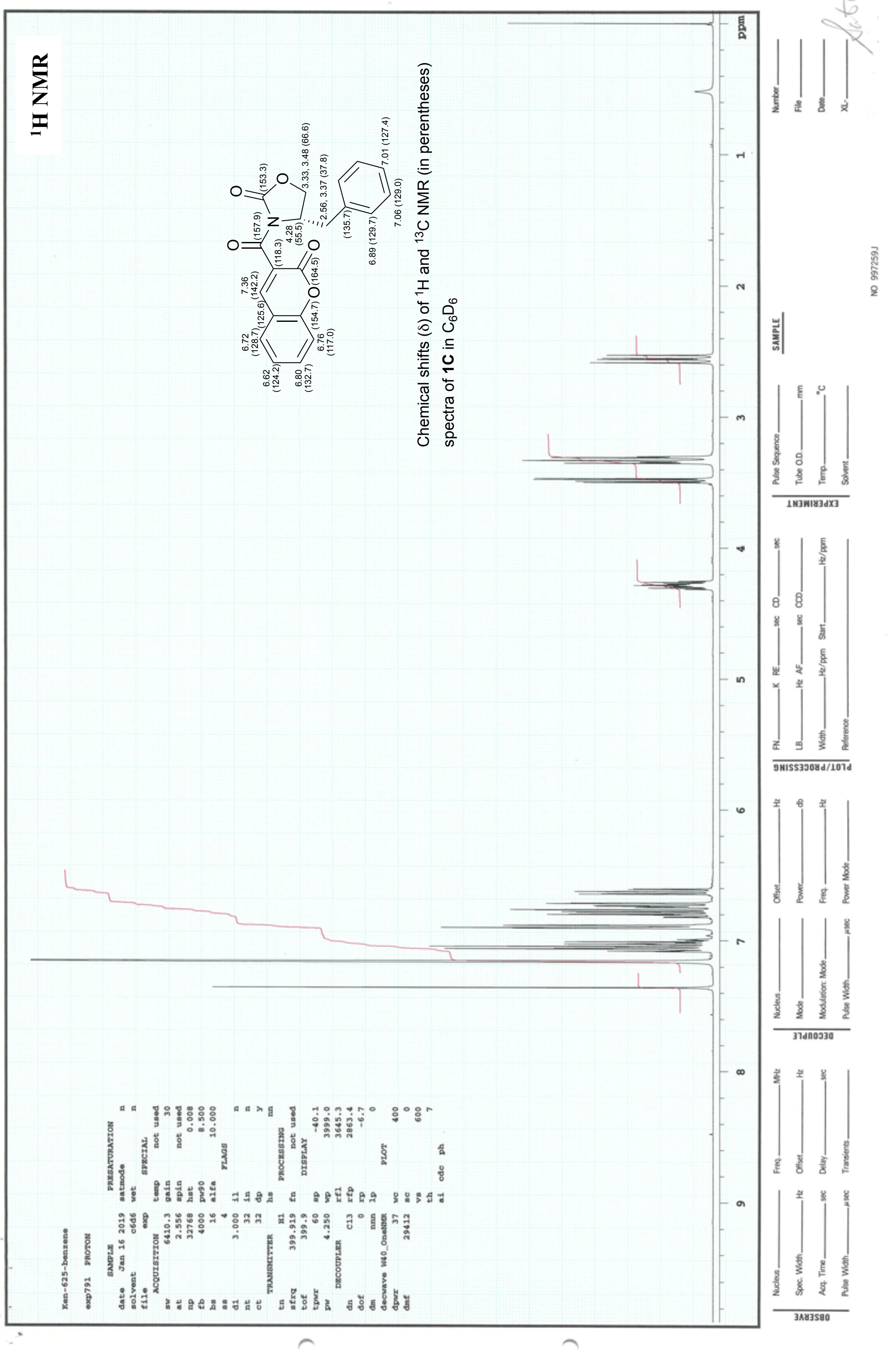

^{1}H NMR

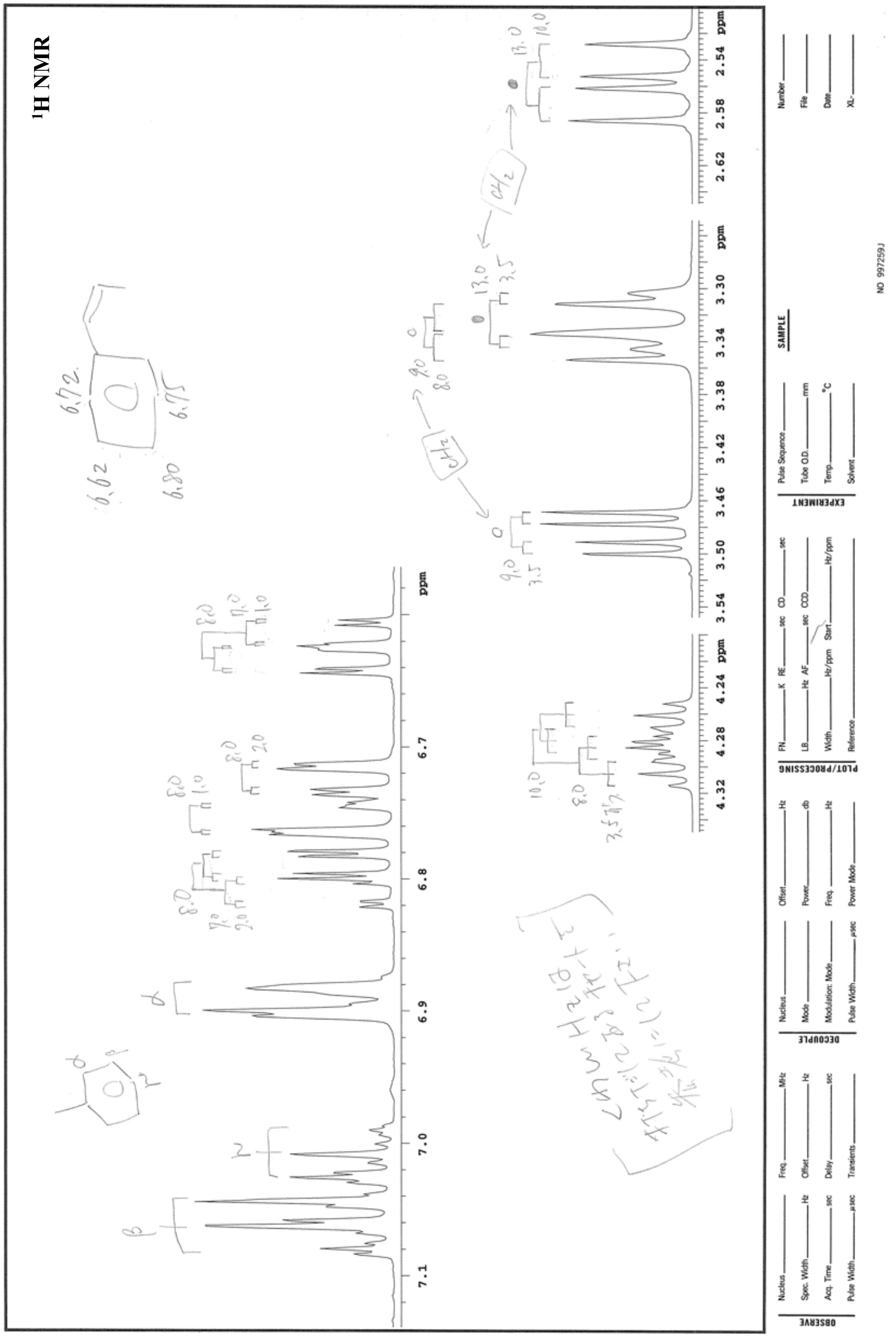

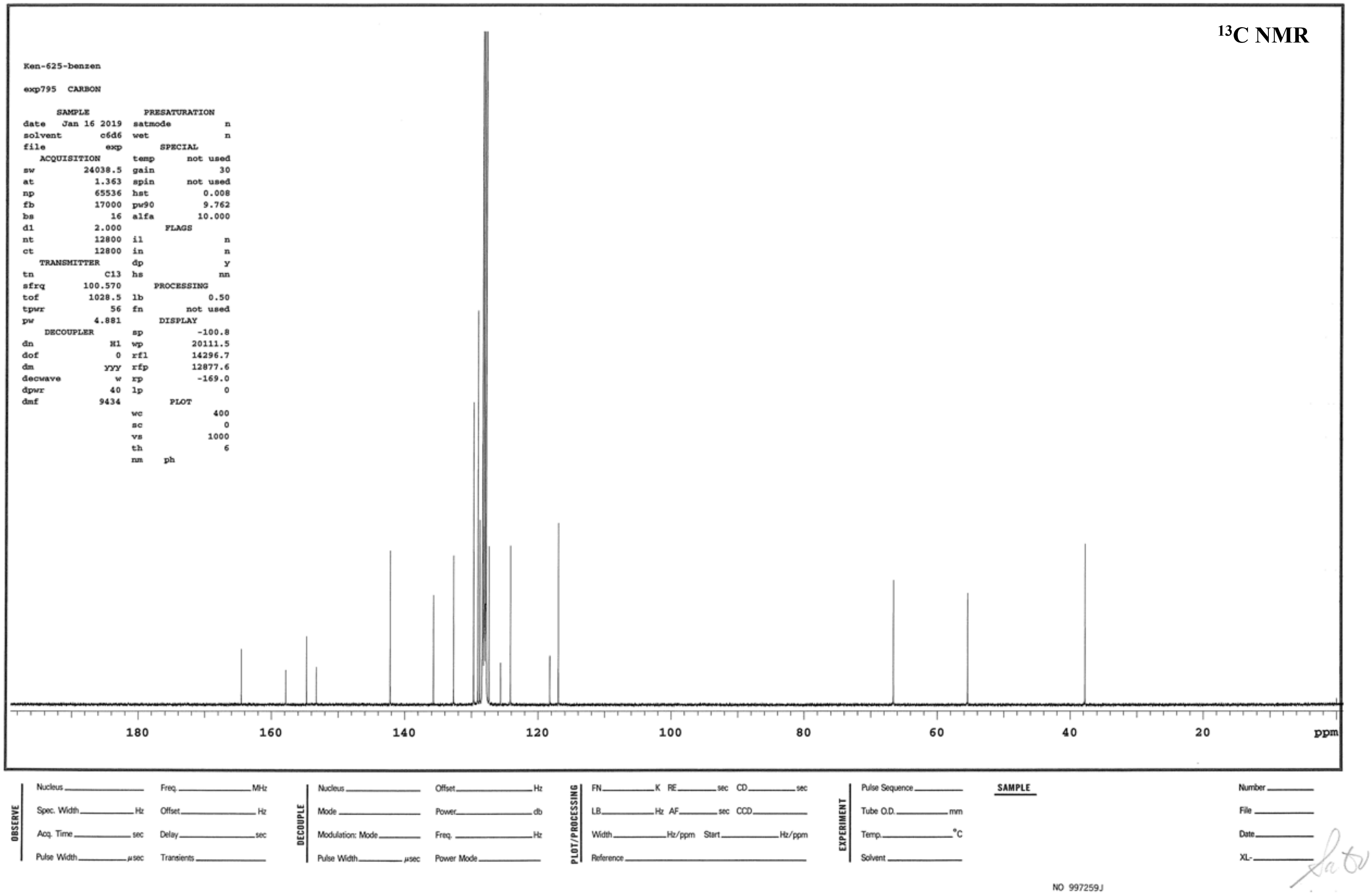
13C NMR
Ken-625-benzen
exp795 CARBON
SAMPLE
date Jan 16 2019
solvent c6d6
file exp
ACQUISITION
sw 24038.5
at 1.363
np 65536
fb 17000
bs 16
d1 2.000
nt 12800
ct 12800
TRANSMITTER
tn C13
sfrq 100.570
tof 1028.5
tpwr 56
pw 4.881
DECOUPLER
dn H1
dof 0
dm yyy
decwave w
dpwr 40
dmf 9434
PRESATURATION
satmode n
wet n
SPECIAL
temp not used
gain 30
spin not used
hst 0.008
pw90 9.762
alfa 10.000
FLAGS
il n
in n
dp y
hs nn
PROCESSING
lb 0.50
fn not used
DISPLAY
sp -100.8
wp 20111.5
rfl 14296.7
rfp 12877.6
rp -169.0
lp 0
PLOT
wc 400
sc 0
vs 1000
th 6
nm ph
180
160
140
120
100
80
60
40
20
ppm
OBSERVE
Nucleus
Freq. MHz
Spec. Width Hz
Offset Hz
Acq. Time sec
Delay sec
Pulse Width μsec
Transients
DECOUPLE
Nucleus
Offset Hz
Mode
Power db
Modulation: Mode
Freq. Hz
Pulse Width μsec
Power Mode
PLOT/PROCESSING
FN K
RE sec
CD sec
LB Hz
AF sec
CCD
Width Hz/ppm
Start Hz/ppm
Reference
EXPERIMENT
Pulse Sequence
Tube O.D. mm
Temp. °C
Solvent
SAMPLE
Number
File
Date
XL-
NO 997259J

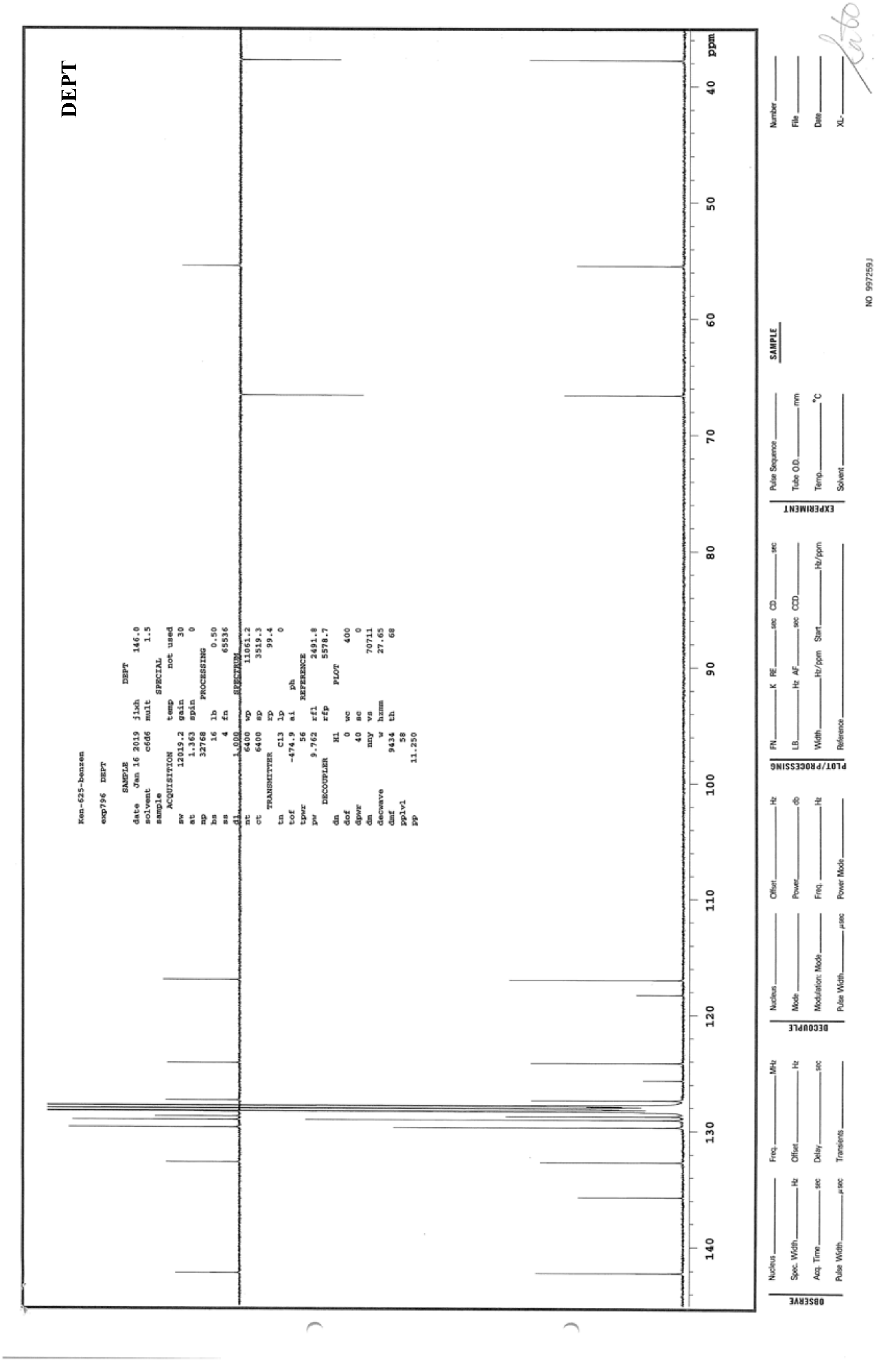
DEPT
Ken-625-benzen
exp796 DEPT
ppm

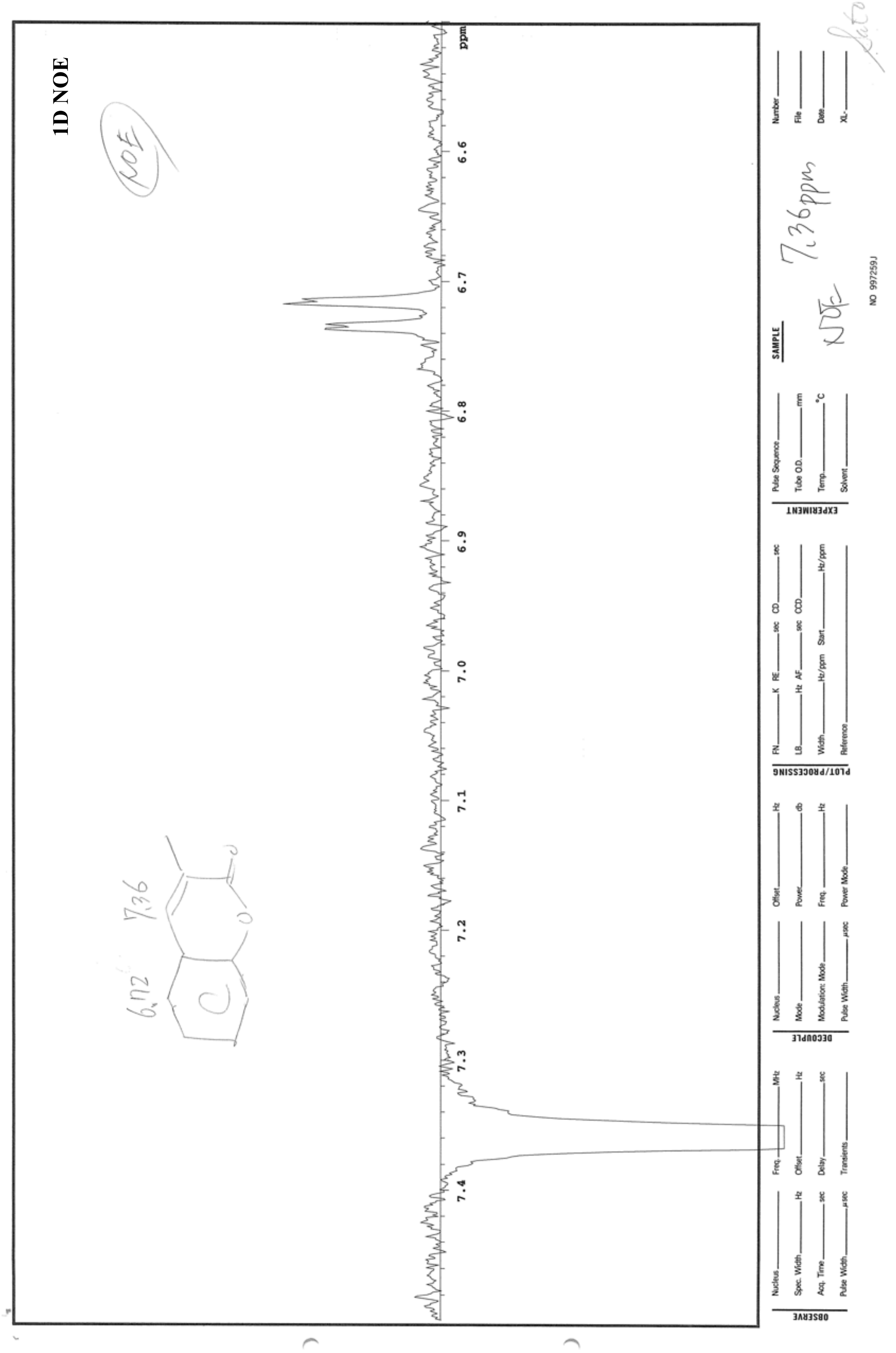
1D NOE
7.4
7.3
7.2
7.1
7.0
6.9
6.8
6.7
6.6
ppm
OBSERVE
DECOUPLE
PLOT/PROCESSING
EXPERIMENT
SAMPLE
NO 997259J

^{1}H-^{1}H COSY

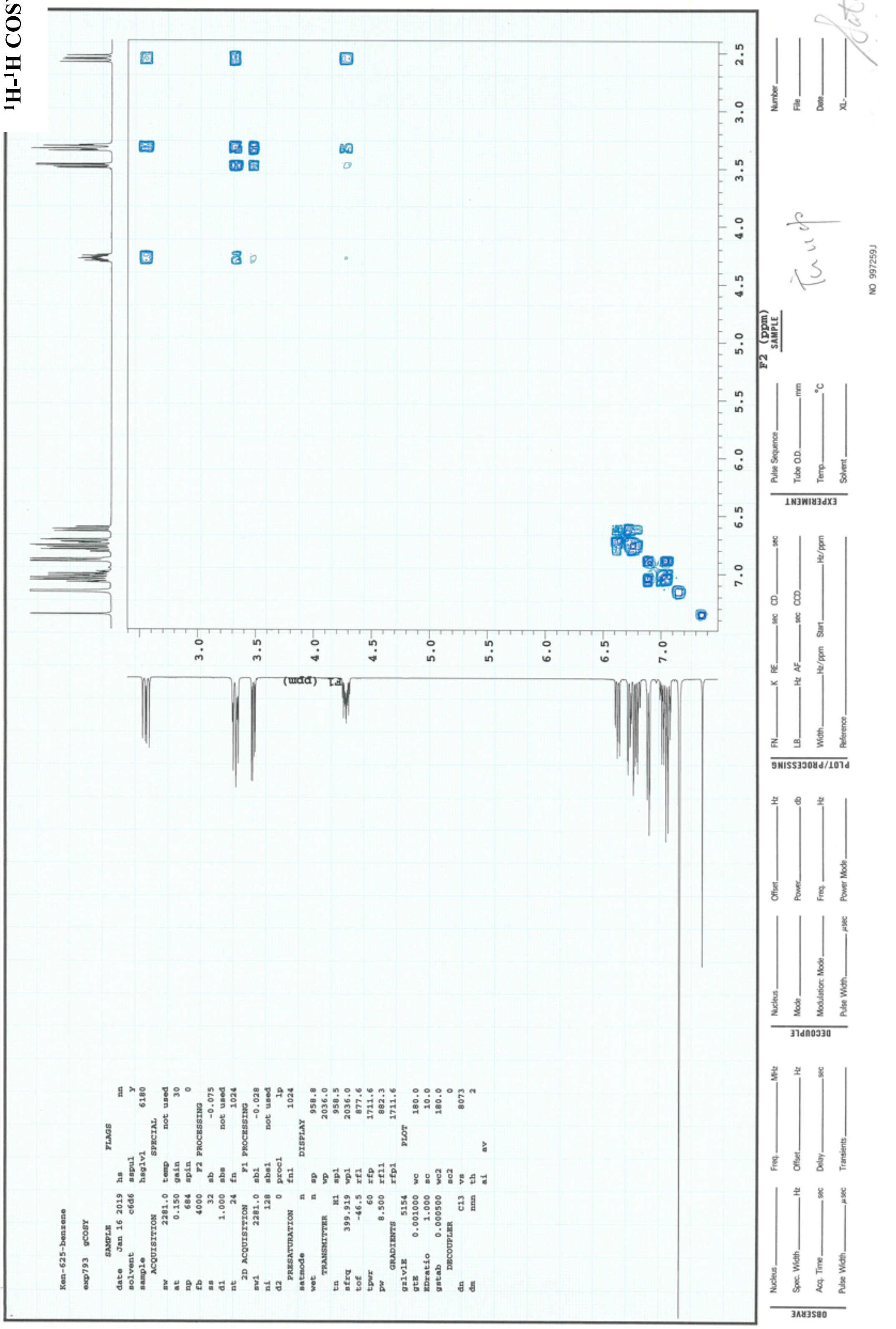

HSQC

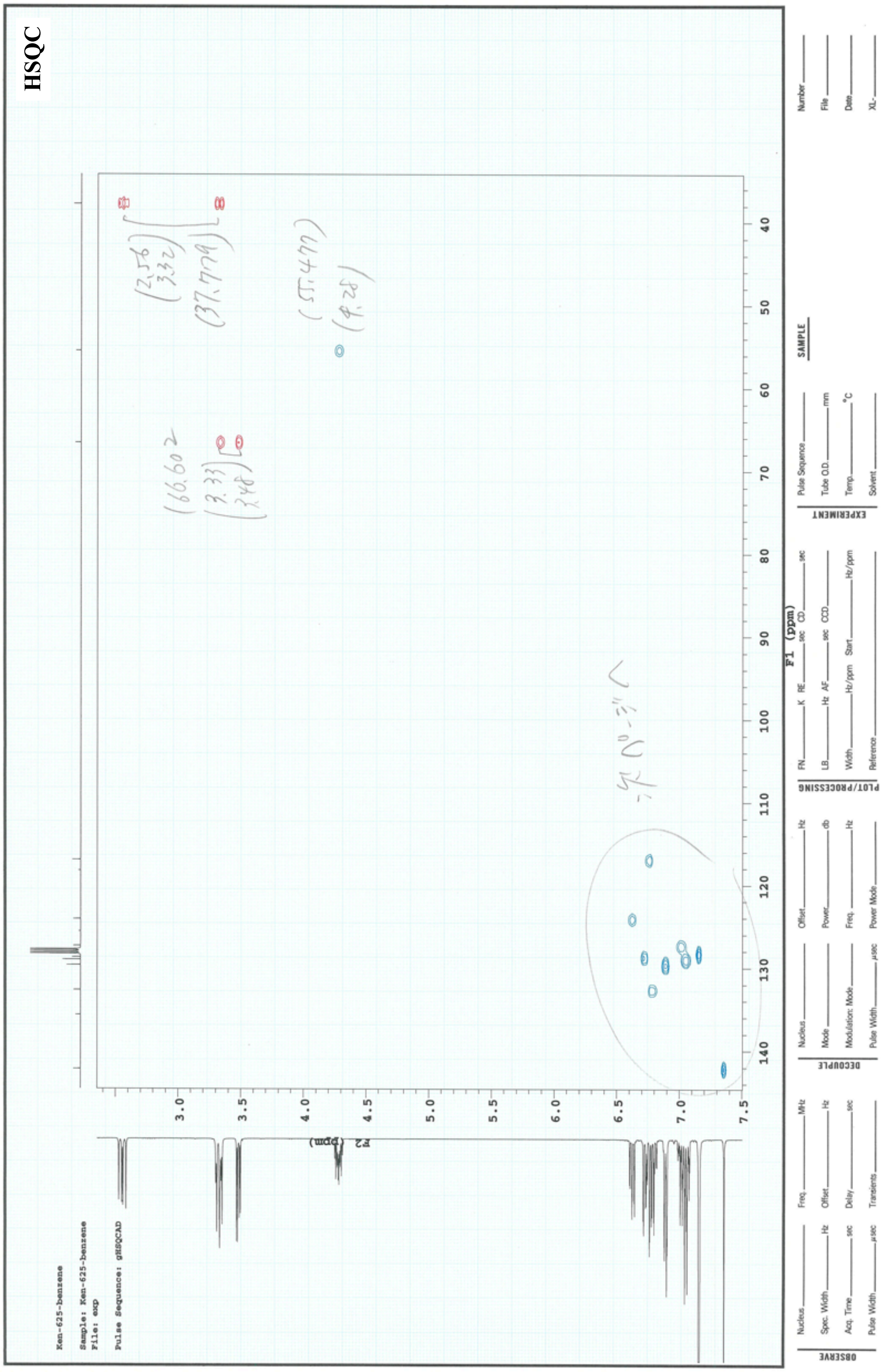
Ken-625-benzene
Sample: Ken-625-benzene
File: exp
Pulse Sequence: gHSQCAD
F1 (ppm)
F2 (ppm)

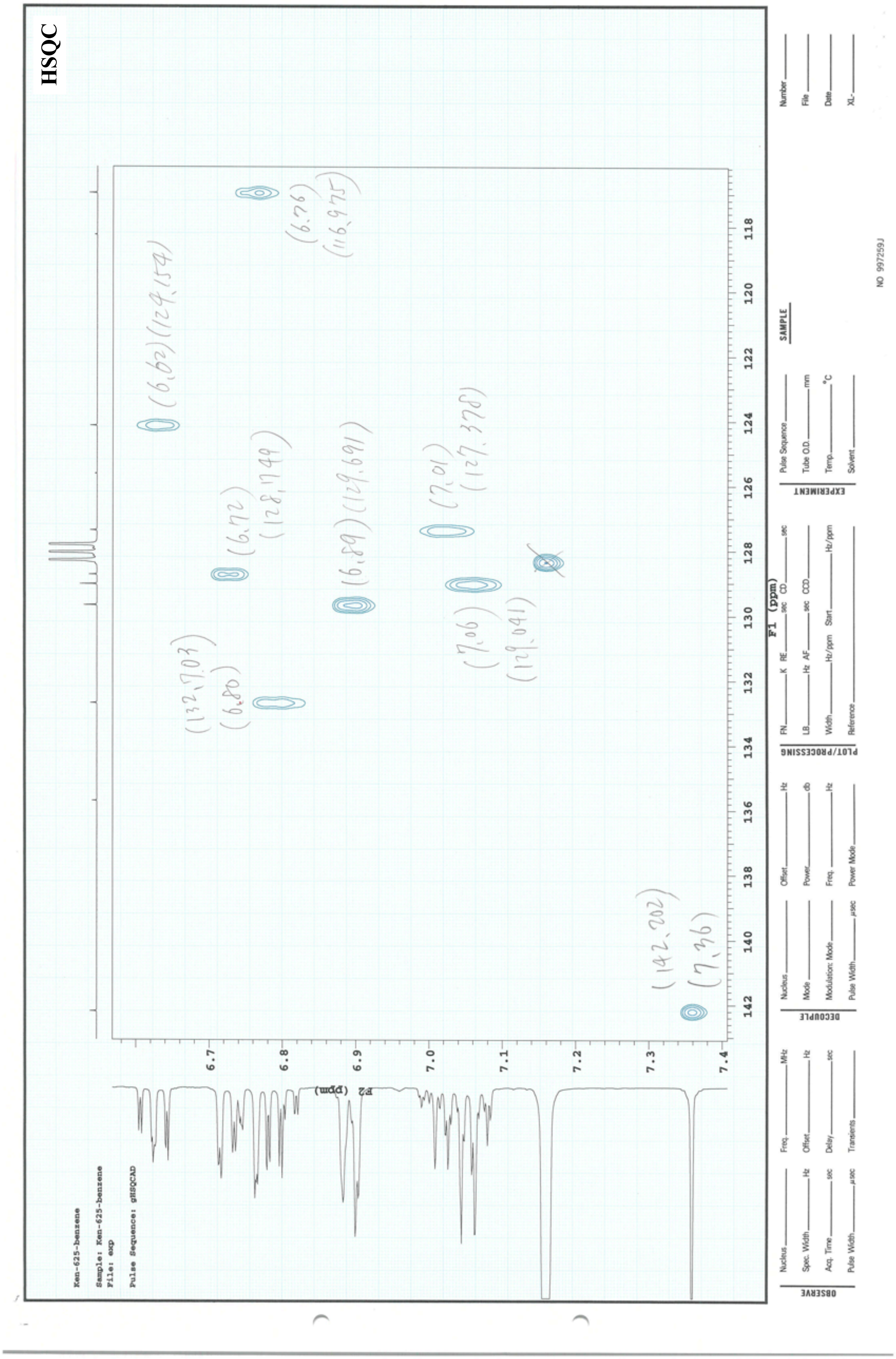
HSQC
Ken-625-benzene
Sample: Ken-625-benzene
File: exp
Pulse Sequence: gHSQCAD
F1 (ppm)
F2 (ppm)

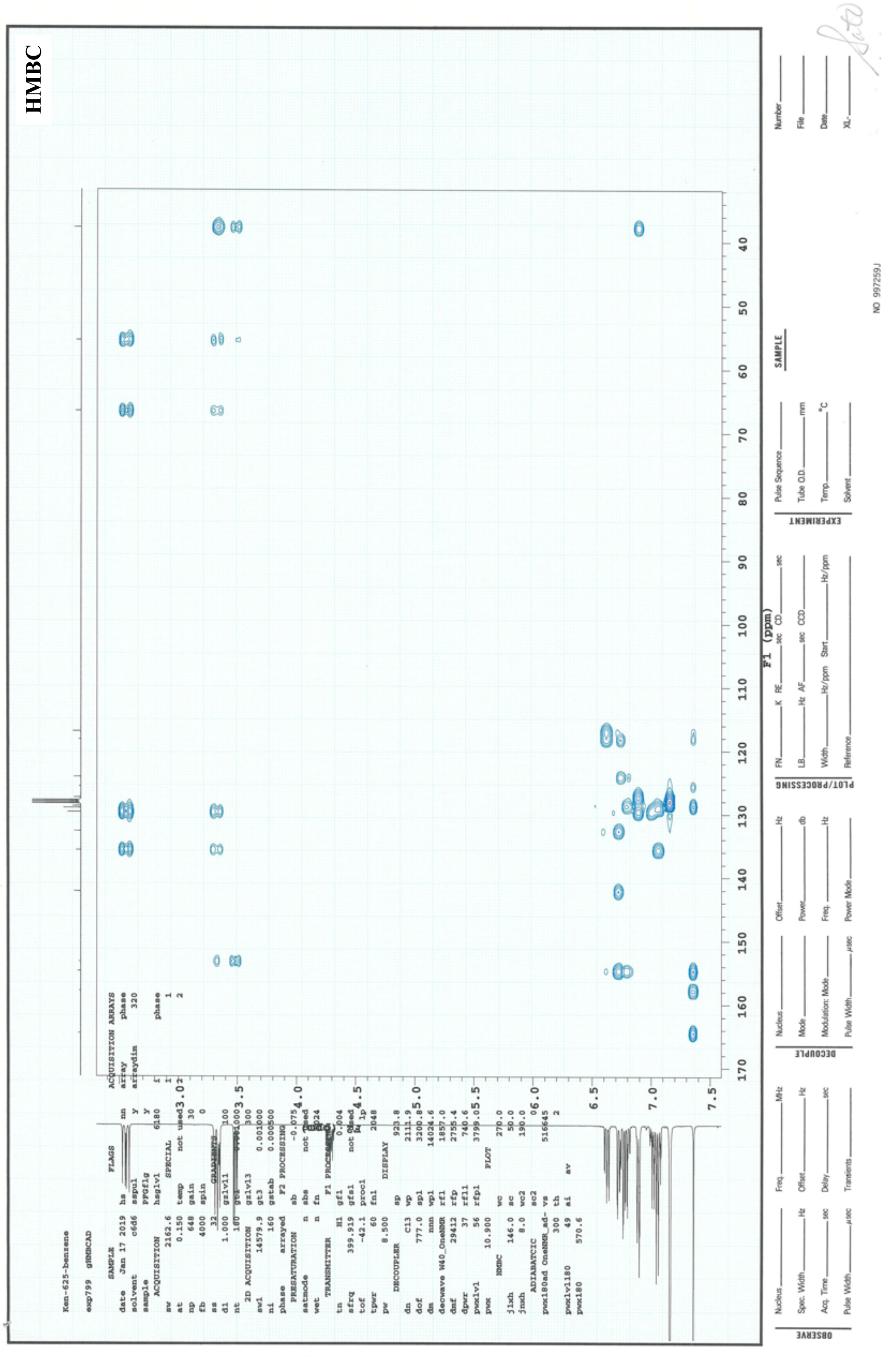
HMBC
F1 (ppm)

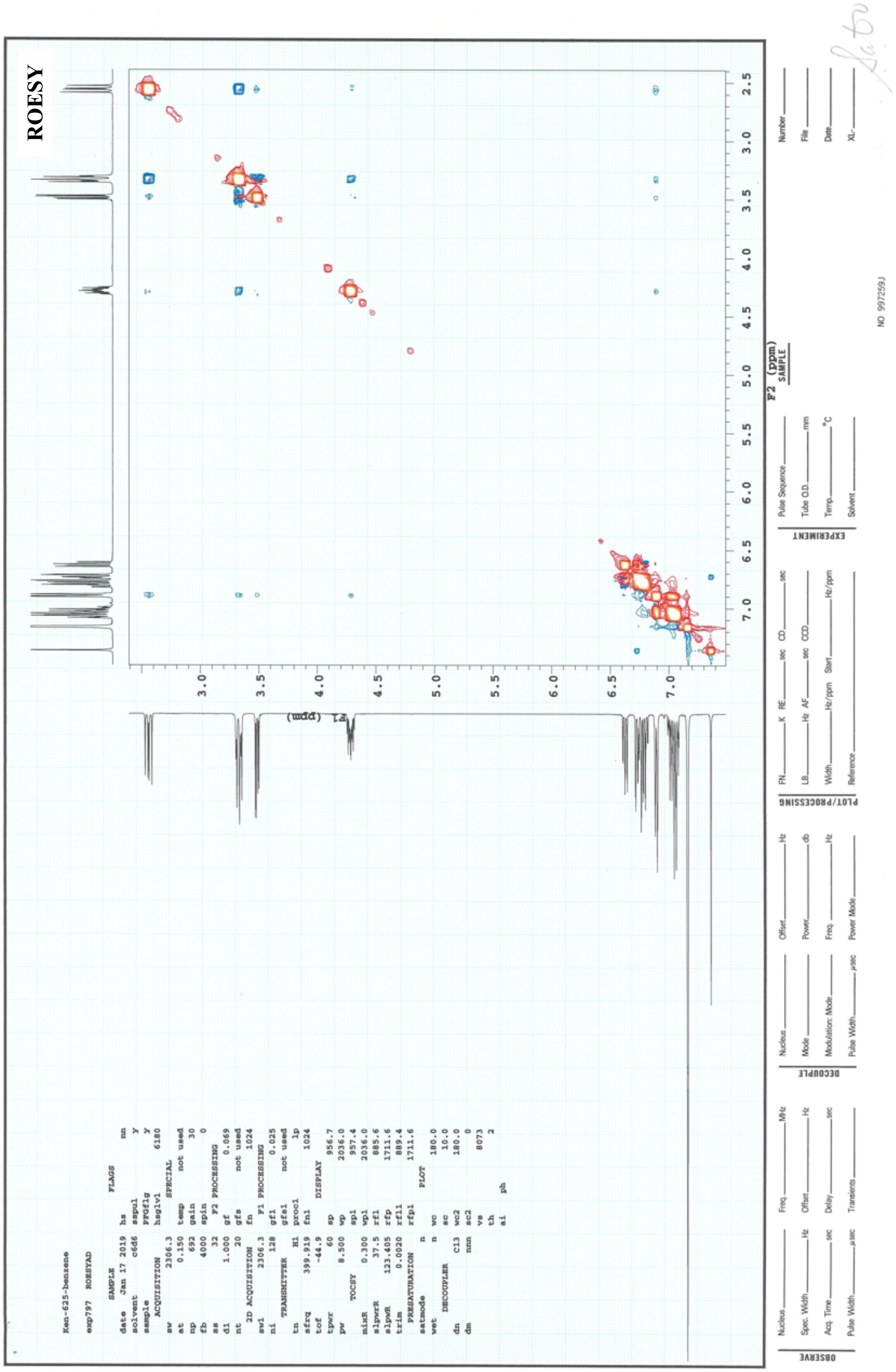
ROESY
Ken-625-benzene
exp797 ROESYAD
F2 (ppm)
F1 (ppm)

NMR spectra of 1C in $(CD_3)_2CO$ (^1H, ^{13}C, ROESY, COSY, HSQC, HMBC)

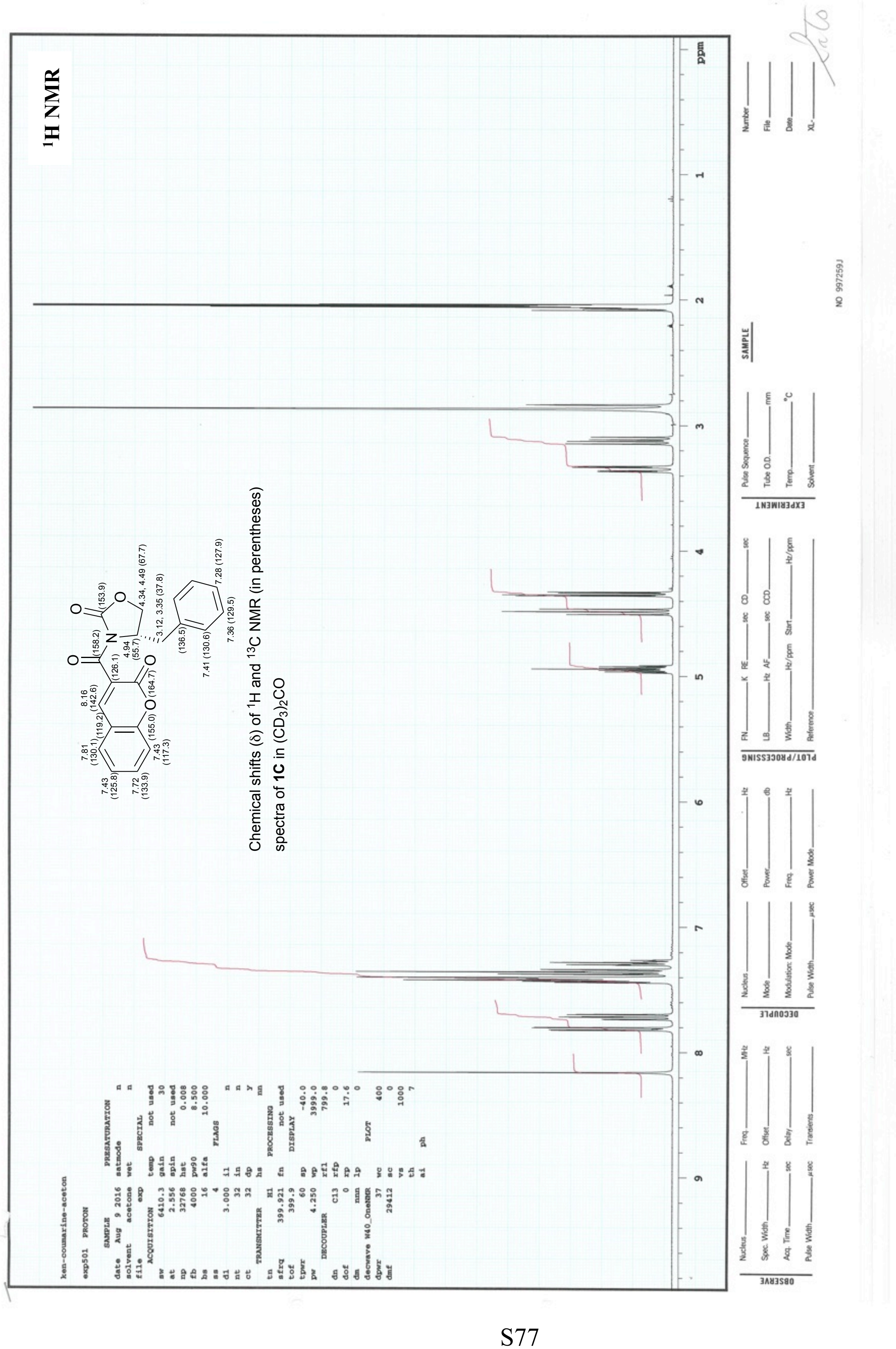

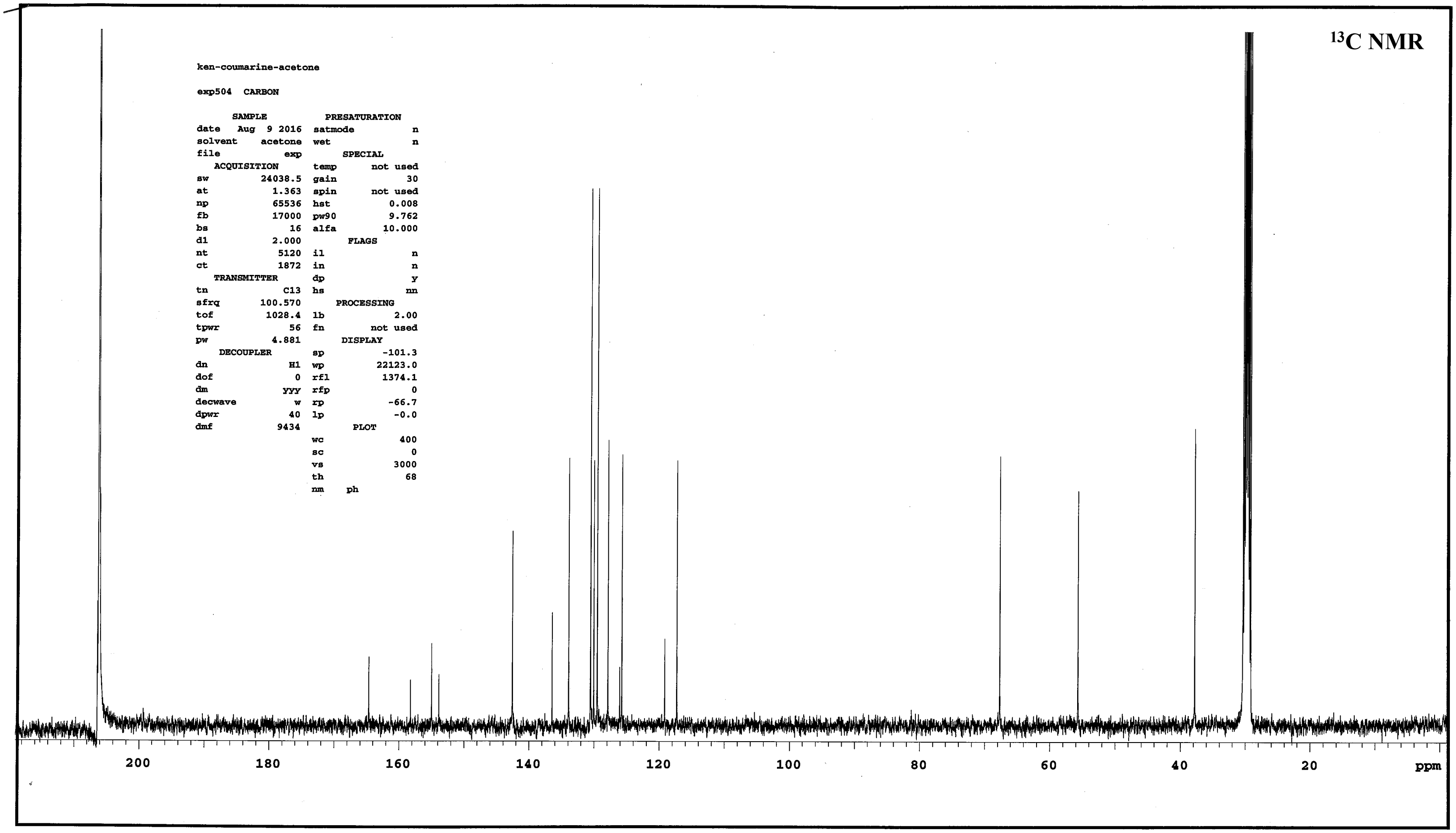

OBSERVE: Nucleus ______ Freq. ______ MHz; Spec. Width ______ Hz Offset ______ Hz; Acq. Time ______ sec Delay ______ sec; Pulse Width ______ μsec Transients ______

DECOUPLE: Nucleus ______ Offset ______ Hz; Mode ______ Power ______ db; Modulation: Mode ______ Freq. ______ Hz; Pulse Width ______ μsec Power Mode ______

PLOT/PROCESSING: FN ______ K RE ______ sec CD ______ sec; LB ______ Hz AF ______ sec CCD ______; Width ______ Hz/ppm Start ______ Hz/ppm; Reference ______

EXPERIMENT: Pulse Sequence ______; Tube O.D. ______ mm; Temp. ______ °C; Solvent ______

SAMPLE

Number ______
File ______
Date ______
XL- ______

NO 997259J

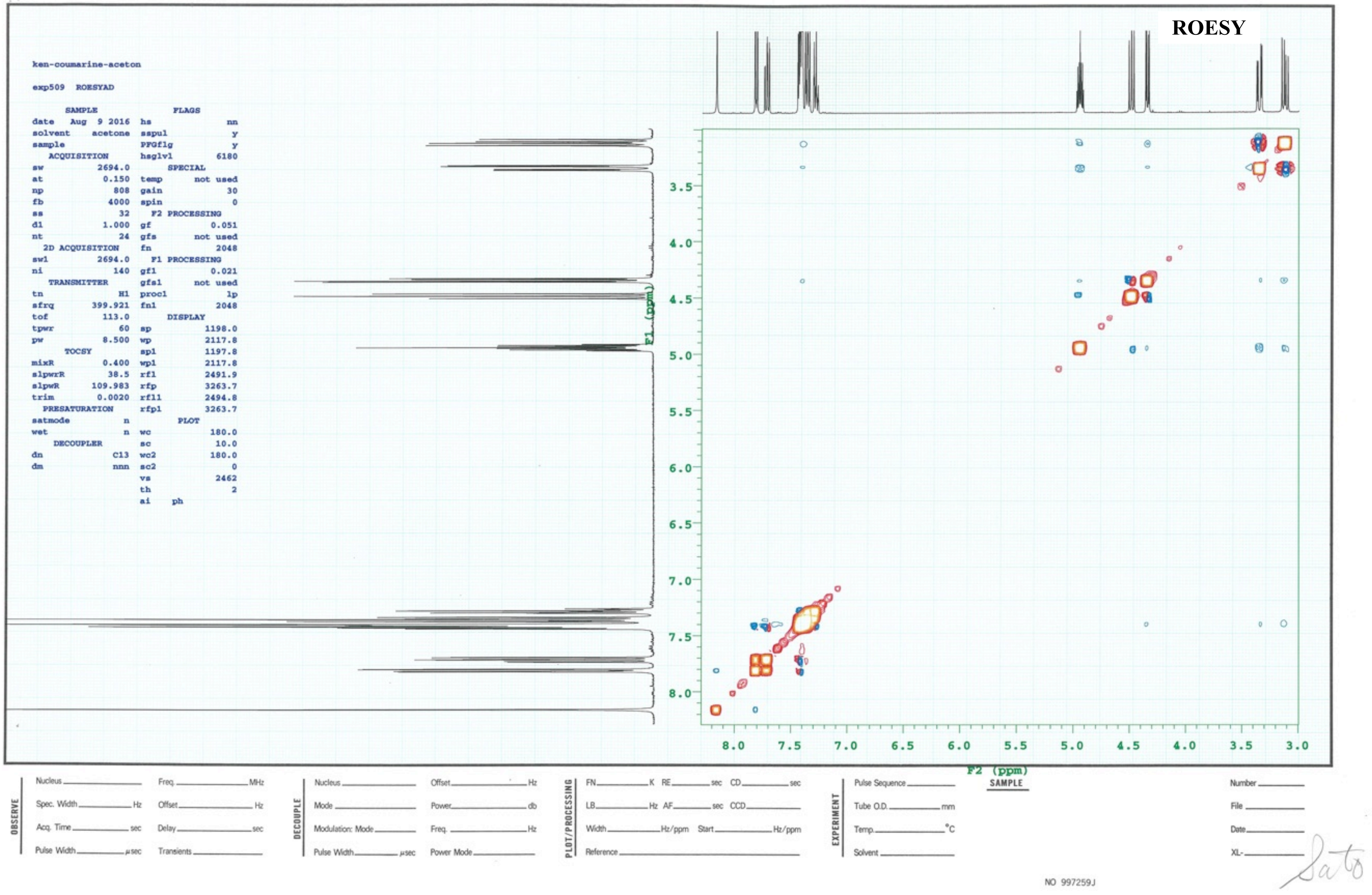
ROESY
ken-coumarine-aceton
exp509 ROESYAD
SAMPLE
date Aug 9 2016
solvent acetone
sample
ACQUISITION
sw 2694.0
at 0.150
np 808
fb 4000
ss 32
d1 1.000
nt 24
2D ACQUISITION
sw1 2694.0
ni 140
TRANSMITTER
tn H1
sfrq 399.921
tof 113.0
tpwr 60
pw 8.500
TOCSY
mixR 0.400
slpwrR 38.5
slpwR 109.983
trim 0.0020
PRESATURATION
satmode n
wet n
DECOUPLER
dn C13
dm nnn
FLAGS
hs nn
sspul y
PFGflg y
hsglvl 6180
SPECIAL
temp not used
gain 30
spin 0
F2 PROCESSING
gf 0.051
gfs not used
fn 2048
F1 PROCESSING
gf1 0.021
gfs1 not used
proc1 lp
fn1 2048
DISPLAY
sp 1198.0
wp 2117.8
sp1 1197.8
wp1 2117.8
rfl 2491.9
rfp 3263.7
rfl1 2494.8
rfp1 3263.7
PLOT
wc 180.0
sc 10.0
wc2 180.0
sc2 0
vs 2462
th 2
ai ph
F1 (ppm)
F2 (ppm)
SAMPLE
OBSERVE
DECOUPLE
PLOT/PROCESSING
EXPERIMENT
Nucleus
Spec. Width
Acq. Time
Pulse Width
Freq.
Offset
Delay
Transients
Mode
Modulation: Mode
Power
Power Mode
FN
LB
Width
Reference
Pulse Sequence
Tube O.D.
Temp.
Solvent
Number
File
Date
XL-
NO 997259J

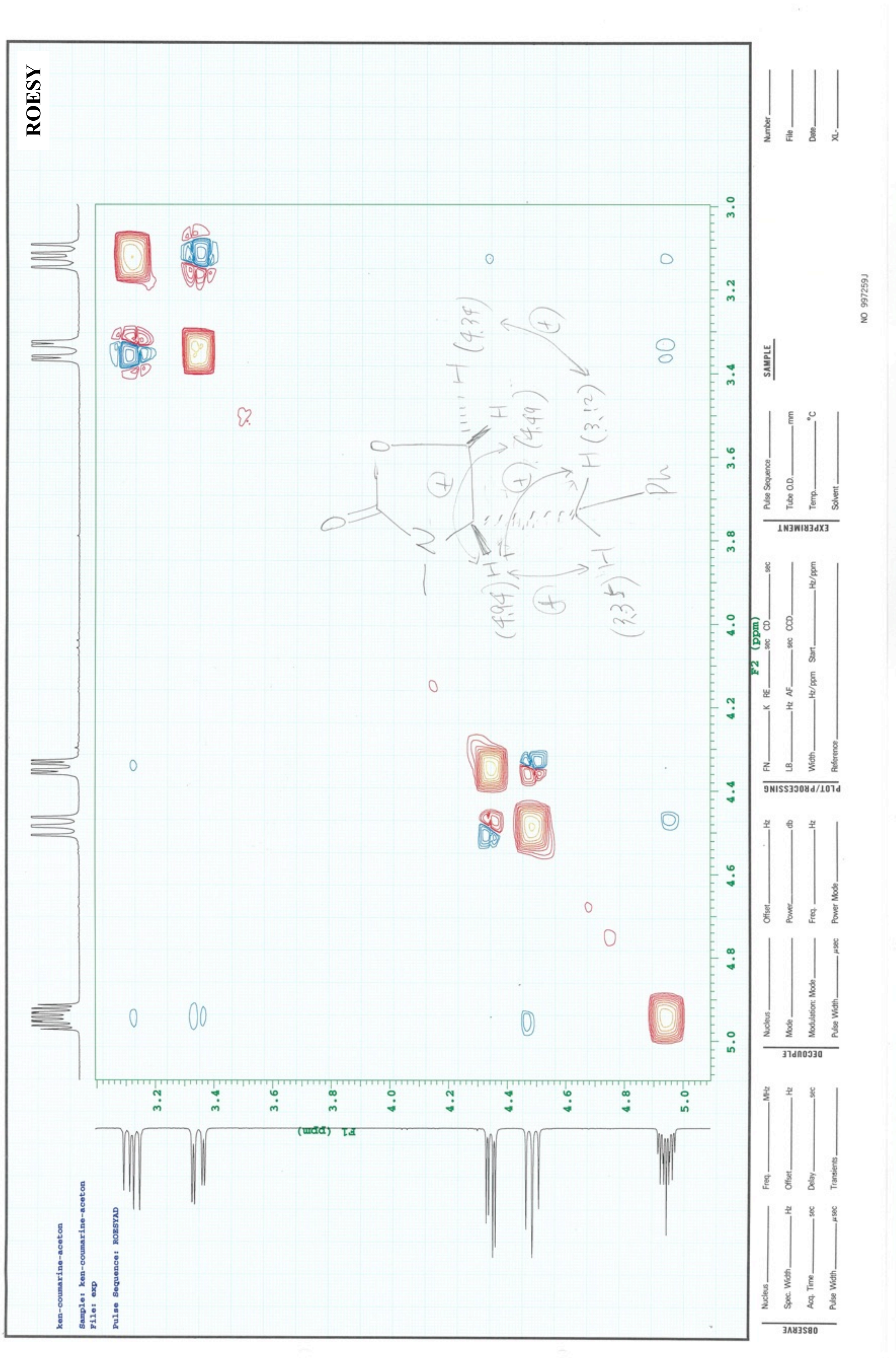
ROESY
ken-coumarine-aceton
Sample: ken-coumarine-aceton
File: exp
Pulse Sequence: ROESYAD
F2 (ppm)
F1 (ppm)

^1H-^1H COSY

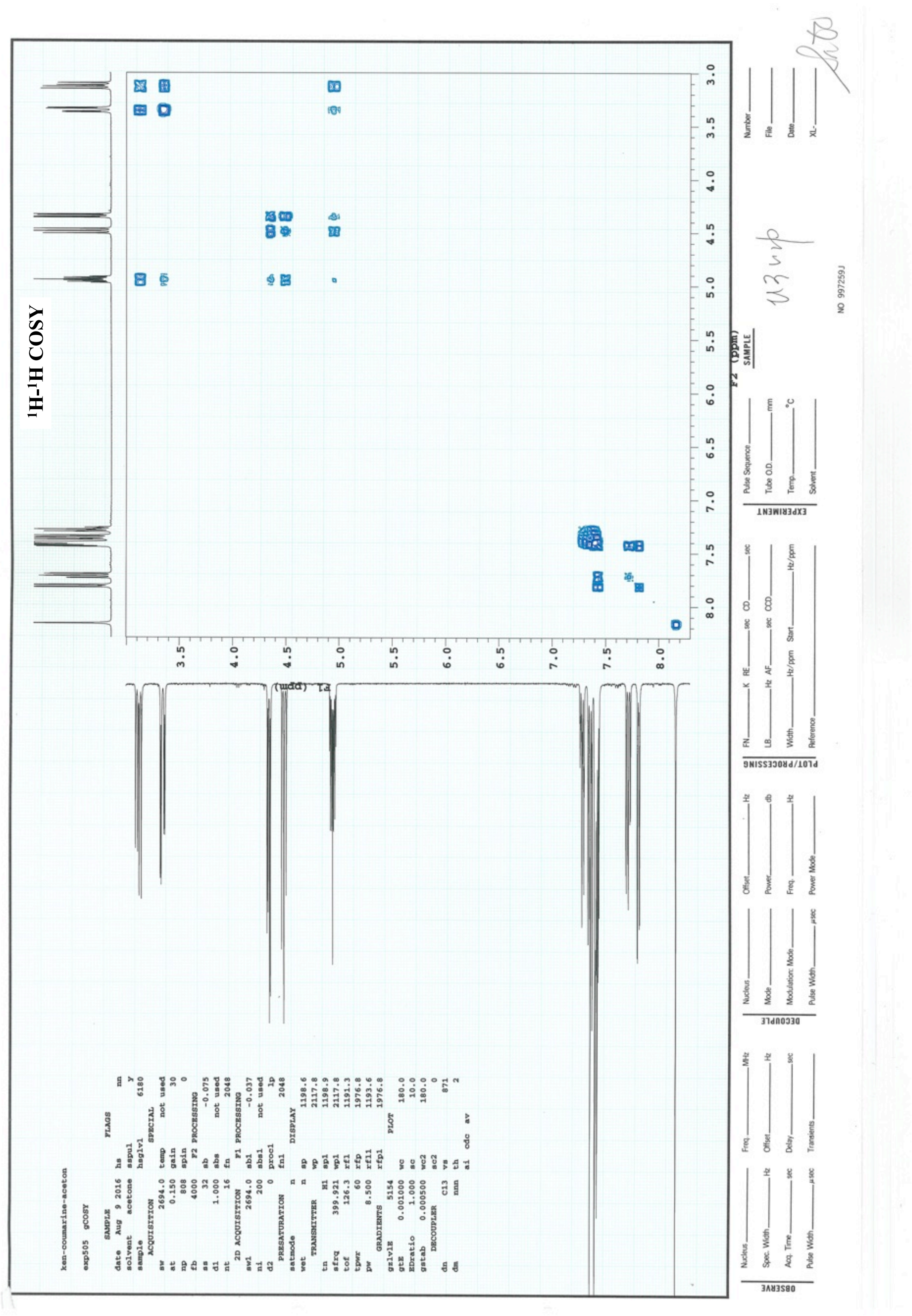

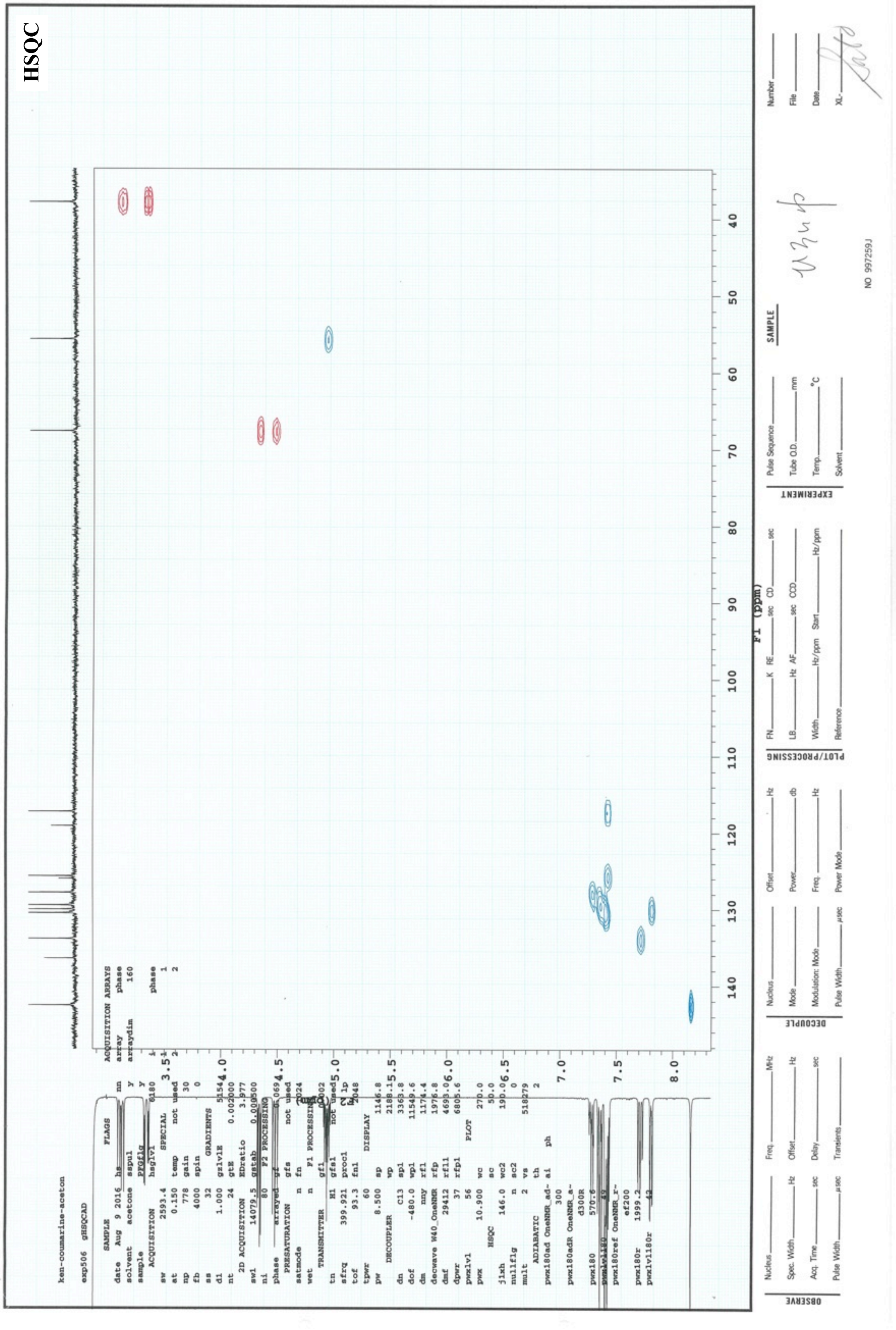
HSQC

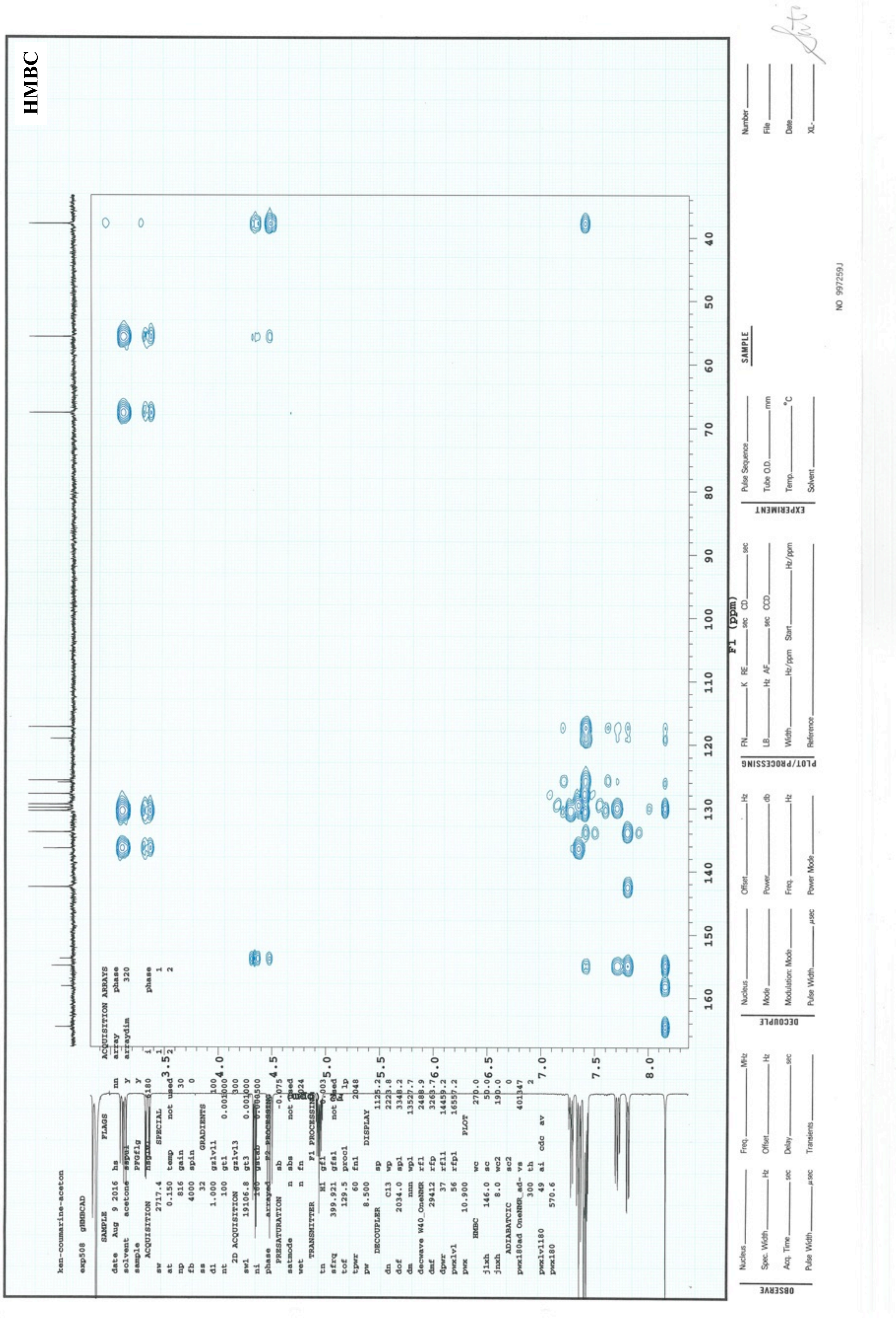
HMBC

UV-vis absorption spectra

UV-vis absorption spectra of **1B**-**1C**, **1B**-BF_3•OEt_2 and **1C**-$Zn(ClO_4)_2$•$6H_2O$ were shown below. UV-vis absorption spectrum of **1A** was difficult to obtain, because of its low solubility in CH_3CN.

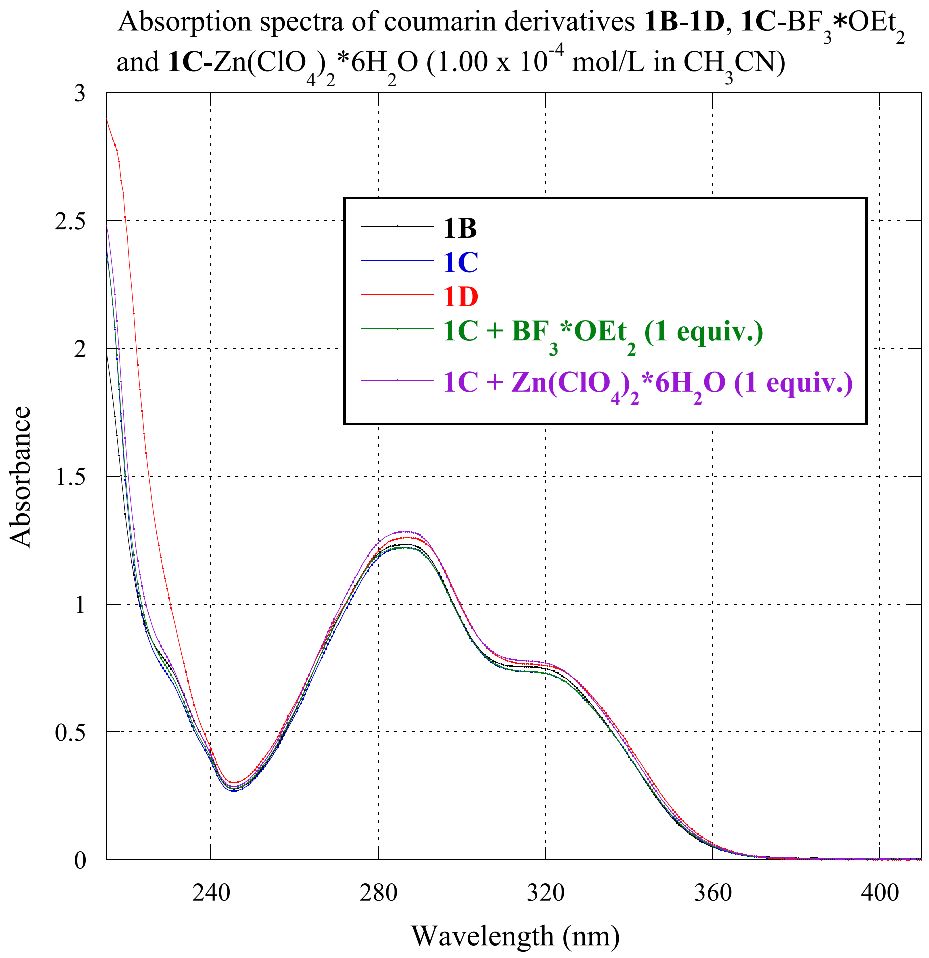

UV-vis absorption spectra of **1C**-$Zn(ClO_4)_2$•$6H_2O$ complexes. Enhanced absorption of **1C**-$Zn(ClO_4)_2$•$6H_2O$ at 287 nm was observed with an increasing amount of $Zn(ClO_4)_2$•$6H_2O$.

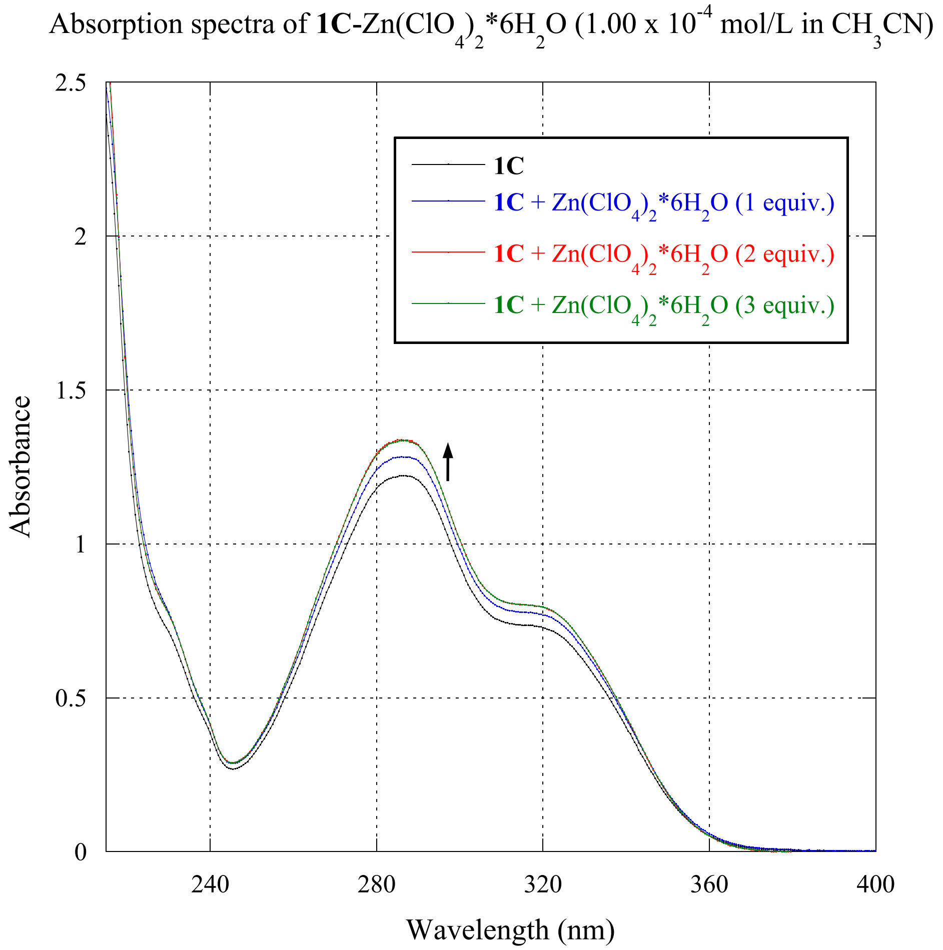

UV-vis absorption spectra of *syn*-HT dimers were shown as below.

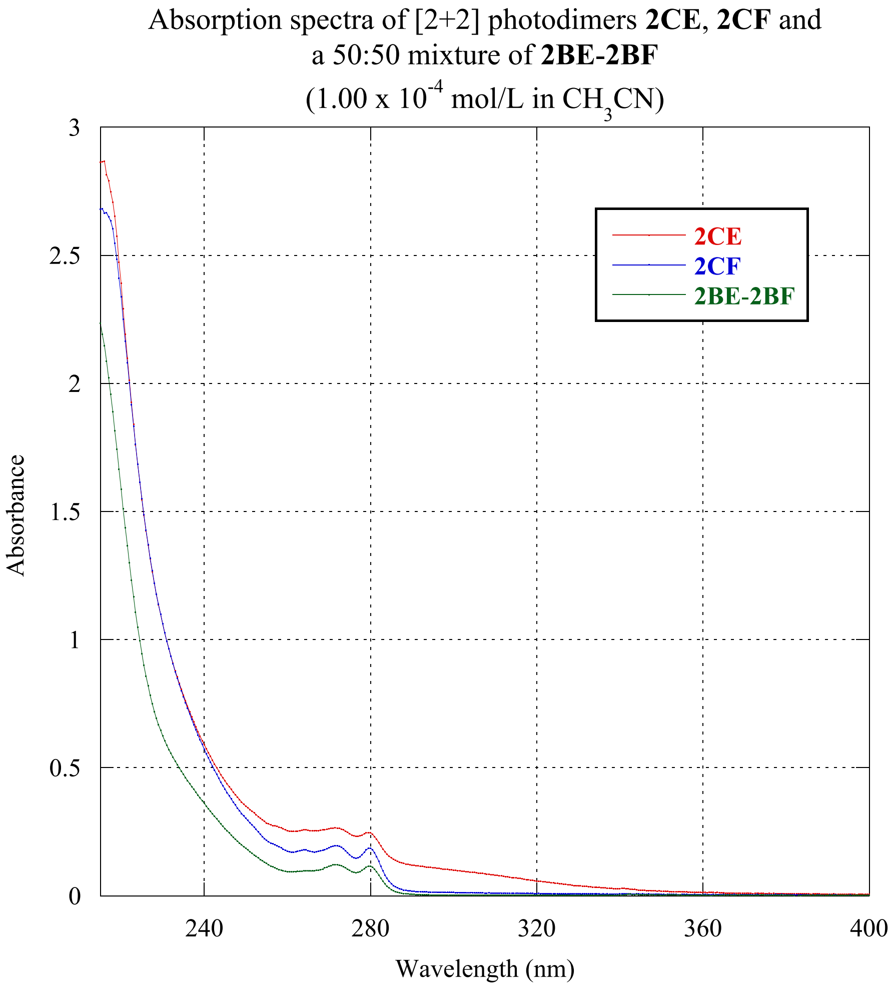

Crystal data

1C

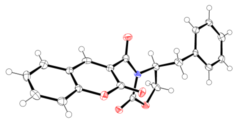

Crystal data

$C_{20}H_{15}NO_5$	V = 1584.3 (5) Å^3
M_r = 349.33	Z = 4
a = 6.1686 (11) Å	F(000) = 728
b = 15.664 (3) Å	D_x = 1.465 Mg m^{-3}
c = 16.396 (3) Å	Mo *K*a radiation, l = 0.71073 Å
a = 90°	m = 0.11 mm^{-1}
b = 90°	T = 296 K
g = 90°	0.40 × 0.20 × 0.20 mm

Data collection

Radiation source: fine-focus sealed tube	R_{int} = 0.039
Graphite monochromator	q_{max} = 20.9°, q_{min} = 1.8°
5186 measured reflections	h = -5®6
1681 independent reflections	k = -11®15
1510 reflections with I > 2s(I)	l = -16®16

Refinement

Refinement on F^2	Secondary atom site location: difference Fourier map
Least-squares matrix: full	Hydrogen site location: inferred from neighbouring sites
$R[F^2 > 2s(F^2)]$ = 0.033	H atoms treated by a mixture of independent and constrained refinement
$wR(F^2)$ = 0.072	$w = 1/[s^2(F_o^2) + (0.0399P)^2]$ where $P = (F_o^2 + 2F_c^2)/3$
S = 1.02	$(D/s)_{max}$ < 0.001
1681 reflections	$D\rho_{max}$ = 0.14 e Å^{-3}
235 parameters	$D\rho_{min}$ = -0.16 e Å^{-3}
0 restraints	Absolute structure: Flack H D (1983), Acta Cryst. A39, 876-881
Primary atom site location: structure-invariant direct methods	Absolute structure parameter: -1.5 (16)

Special details

Geometry. All esds (except the esd in the dihedral angle between two l.s. planes) are estimated using the full covariance matrix. The cell esds are taken into account individually in the estimation of esds in distances, angles and torsion angles; correlations between esds in cell parameters are only used when they are defined by crystal symmetry. An approximate (isotropic) treatment of cell esds is used for estimating esds involving l.s. planes.
Refinement. Refinement of F^2 against ALL reflections. The weighted R-factor wR and goodness of fit S are based on F^2, conventional R-factors R are based on F, with F set to zero for negative F^2. The threshold expression of F^2 > 2sigma(F^2) is used only for calculating R-factors(gt) etc. and is not relevant to the choice of reflections for refinement. R-factors based on F^2 are statistically about twice as large as those based on F, and R-factors based on ALL data will be even larger.

Fractional atomic coordinates and isotropic or equivalent isotropic displacement parameters (Å^2) for (hk041_2_0m_specegroups)

	x	y	z	U_{iso}*/U_{eq}
O5	0.8961 (3)	0.14397 (12)	0.30397 (11)	0.0185 (5)
O1	0.7006 (4)	0.35352 (13)	0.34080 (11)	0.0195 (6)
O4	1.0368 (4)	0.05975 (13)	0.20746 (12)	0.0201 (6)
O3	0.4333 (4)	0.18725 (13)	0.12513 (12)	0.0242 (6)
O2	0.8909 (4)	0.31524 (12)	0.23251 (12)	0.0218 (6)
C4	0.1658 (6)	0.29297 (19)	0.41969 (17)	0.0205 (9)
H4	0.0491	0.2578	0.4071	0.025*
C9	0.5711 (6)	0.19044 (19)	0.17841 (18)	0.0177 (8)
C20	0.7892 (6)	0.10853 (18)	-0.10400 (17)	0.0185 (9)
H20	0.6575	0.1357	-0.0947	0.022*
C6	0.5179 (5)	0.34848 (19)	0.38868 (17)	0.0155 (8)
C19	0.8196 (6)	0.06235 (18)	-0.17544 (19)	0.0194 (8)
H19	0.7088	0.0589	-0.2137	0.023*
C16	1.1501 (6)	0.07503 (19)	-0.06217 (18)	0.0225 (9)
H16	1.2631	0.0801	-0.0249	0.027*
C1	0.5151 (6)	0.39802 (19)	0.45869 (19)	0.0213 (9)
H1	0.6320	0.4329	0.4718	0.026*
C15	0.9536 (6)	0.11452 (18)	-0.04633 (18)	0.0154 (8)
C8	0.5504 (6)	0.24599 (17)	0.25149 (17)	0.0161 (8)
C5	0.3460 (5)	0.29565 (18)	0.36809 (17)	0.0149 (8)
C7	0.3689 (6)	0.24457 (18)	0.29588 (18)	0.0187 (8)
H7	0.2548	0.2097	0.2797	0.022*
C13	0.8132 (6)	0.09662 (18)	0.09553 (17)	0.0181 (8)
H13	0.6858	0.0689	0.0716	0.022*
C11	0.8945 (5)	0.11822 (18)	0.2349 (2)	0.0165 (8)
C14	0.9135 (6)	0.15854 (19)	0.03440 (16)	0.0200 (8)
H14A	0.8167	0.2066	0.0263	0.024*
H14B	1.0494	0.1801	0.0558	0.024*
C2	0.3340 (6)	0.39449 (19)	0.50896 (19)	0.0228 (9)
H2	0.3292	0.4274	0.5562	0.027*
C3	0.1606 (6)	0.34226 (19)	0.48916 (18)	0.0219 (8)
H3	0.0398	0.3405	0.5231	0.026*
C10	0.7277 (6)	0.30460 (19)	0.27113 (19)	0.0181 (8)
C18	1.0156 (6)	0.02138 (19)	-0.18961 (19)	0.0232 (9)
H18	1.0357	-0.0103	-0.2370	0.028*
C17	1.1801 (6)	0.02786 (19)	-0.13330 (18)	0.0228 (9)
H17	1.3117	0.0007	-0.1428	0.027*
C12	0.9699 (6)	0.0298 (2)	0.12789 (17)	0.0266 (9)
H12A	0.8991	-0.0253	0.1322	0.032*
H12B	1.0941	0.0243	0.0920	0.032*
N1	0.7539 (4)	0.13946 (16)	0.17201 (14)	0.0159 (7)

Atomic displacement parameters (Å^2) for (hk041_2_0m_specegroups)

	U^{11}	U^{22}	U^{33}	U^{12}	U^{13}	U^{23}
O5	0.0218 (15)	0.0200 (11)	0.0138 (12)	-0.0015 (11)	-0.0023 (11)	-0.0014 (11)
O1	0.0189 (15)	0.0188 (12)	0.0207 (12)	-0.0019 (11)	0.0025 (11)	-0.0031 (11)
O4	0.0208 (15)	0.0207 (11)	0.0188 (13)	0.0055 (11)	-0.0019 (11)	0.0001 (10)
O3	0.0198 (15)	0.0288 (13)	0.0240 (12)	0.0017 (11)	-0.0050 (13)	-0.0028 (11)
O2	0.0177 (16)	0.0209 (12)	0.0267 (12)	-0.0030 (11)	0.0052 (13)	0.0029 (10)
C4	0.022 (2)	0.0161 (17)	0.0236 (19)	0.0012 (17)	0.0015 (18)	0.0034 (16)
C9	0.017 (2)	0.0166 (17)	0.0193 (19)	-0.0039 (17)	0.0001 (18)	0.0023 (16)
C20	0.018 (2)	0.0181 (18)	0.0197 (19)	0.0022 (16)	0.0001 (19)	0.0037 (16)
C6	0.017 (2)	0.0145 (17)	0.0150 (18)	0.0017 (18)	0.0009 (17)	0.0020 (16)

C19	0.023 (2)	0.0191 (17)	0.0160 (18)	-0.0047 (18)	-0.0040 (16)	0.0014 (16)
C16	0.025 (3)	0.0246 (19)	0.018 (2)	-0.0026 (19)	-0.0040 (17)	0.0048 (17)
C1	0.023 (3)	0.0191 (17)	0.022 (2)	-0.0006 (17)	-0.0038 (19)	0.0037 (18)
C15	0.016 (2)	0.0123 (16)	0.0174 (18)	-0.0001 (16)	0.0040 (18)	0.0033 (15)
C8	0.017 (2)	0.0153 (17)	0.0161 (18)	0.0030 (16)	-0.0017 (17)	-0.0026 (15)
C5	0.016 (2)	0.0118 (17)	0.0168 (17)	0.0030 (17)	0.0002 (17)	0.0027 (16)
C7	0.019 (2)	0.0146 (17)	0.0228 (18)	0.0022 (16)	-0.0089 (18)	0.0000 (16)
C13	0.022 (2)	0.0166 (17)	0.0163 (18)	0.0015 (17)	0.0003 (17)	-0.0027 (15)
C11	0.013 (2)	0.0116 (18)	0.025 (2)	-0.0028 (16)	0.004 (2)	0.0047 (16)
C14	0.026 (2)	0.0153 (17)	0.0191 (17)	0.0016 (17)	-0.0019 (17)	-0.0002 (16)
C2	0.039 (3)	0.0129 (17)	0.0168 (19)	0.008 (2)	-0.002 (2)	0.0012 (15)
C3	0.026 (2)	0.0195 (19)	0.0203 (18)	0.0067 (19)	0.0079 (17)	0.0038 (17)
C10	0.019 (2)	0.0171 (18)	0.0184 (19)	0.0018 (18)	-0.0016 (19)	0.0030 (17)
C18	0.035 (3)	0.0170 (17)	0.0177 (19)	-0.0022 (18)	0.0037 (19)	-0.0016 (16)
C17	0.019 (2)	0.027 (2)	0.022 (2)	0.0024 (16)	0.005 (2)	0.0017 (18)
C12	0.040 (3)	0.0258 (19)	0.0138 (17)	0.0016 (18)	-0.0031 (18)	-0.0033 (16)
N1	0.0185 (18)	0.0185 (14)	0.0108 (13)	0.0043 (14)	-0.0005 (13)	-0.0033 (13)

Geometric parameters (Å, º) for (hk041_2_0m_specegroups)

O5—C11	1.201 (3)	C1—C2	1.389 (5)
O1—C6	1.376 (4)	C1—H1	0.9300
O1—C10	1.386 (3)	C15—C14	1.513 (4)
O4—C11	1.346 (4)	C8—C7	1.336 (4)
O4—C12	1.447 (3)	C8—C10	1.464 (5)
O3—C9	1.220 (4)	C5—C7	1.436 (4)
O2—C10	1.201 (4)	C7—H7	0.9300
C4—C3	1.376 (4)	C13—N1	1.468 (3)
C4—C5	1.397 (4)	C13—C12	1.521 (4)
C4—H4	0.9300	C13—C14	1.526 (4)
C9—N1	1.386 (4)	C13—H13	0.9800
C9—C8	1.486 (4)	C11—N1	1.388 (4)
C20—C19	1.390 (4)	C14—H14A	0.9700
C20—C15	1.390 (4)	C14—H14B	0.9700
C20—H20	0.9300	C2—C3	1.385 (5)
C6—C1	1.386 (4)	C2—H2	0.9300
C6—C5	1.387 (4)	C3—H3	0.9300
C19—C18	1.389 (5)	C18—C17	1.375 (4)
C19—H19	0.9300	C18—H18	0.9300
C16—C15	1.386 (5)	C17—H17	0.9300
C16—C17	1.393 (4)	C12—H12A	0.9700
C16—H16	0.9300	C12—H12B	0.9700
C6—O1—C10	122.5 (2)	C12—C13—C14	114.2 (3)
C11—O4—C12	109.7 (3)	N1—C13—H13	110.1
C3—C4—C5	120.2 (3)	C12—C13—H13	110.1
C3—C4—H4	119.9	C14—C13—H13	110.1
C5—C4—H4	119.9	O5—C11—O4	122.6 (3)
O3—C9—N1	119.3 (3)	O5—C11—N1	128.7 (3)
O3—C9—C8	122.8 (3)	O4—C11—N1	108.8 (3)
N1—C9—C8	117.9 (3)	C15—C14—C13	110.6 (2)
C19—C20—C15	120.7 (3)	C15—C14—H14A	109.5
C19—C20—H20	119.7	C13—C14—H14A	109.5
C15—C20—H20	119.7	C15—C14—H14B	109.5
O1—C6—C1	116.8 (3)	C13—C14—H14B	109.5
O1—C6—C5	121.5 (3)	H14A—C14—H14B	108.1
C1—C6—C5	121.7 (3)	C3—C2—C1	120.3 (3)

C18—C19—C20	119.9 (3)	C3—C2—H2	119.8
C18—C19—H19	120.0	C1—C2—H2	119.8
C20—C19—H19	120.0	C4—C3—C2	120.5 (3)
C15—C16—C17	120.6 (3)	C4—C3—H3	119.8
C15—C16—H16	119.7	C2—C3—H3	119.8
C17—C16—H16	119.7	O2—C10—O1	117.3 (3)
C6—C1—C2	118.6 (3)	O2—C10—C8	126.7 (3)
C6—C1—H1	120.7	O1—C10—C8	116.0 (3)
C2—C1—H1	120.7	C17—C18—C19	119.7 (3)
C16—C15—C20	118.7 (3)	C17—C18—H18	120.1
C16—C15—C14	120.7 (3)	C19—C18—H18	120.1
C20—C15—C14	120.5 (3)	C18—C17—C16	120.2 (3)
C7—C8—C10	121.1 (3)	C18—C17—H17	119.9
C7—C8—C9	120.1 (3)	C16—C17—H17	119.9
C10—C8—C9	118.7 (3)	O4—C12—C13	105.8 (2)
C6—C5—C4	118.6 (3)	O4—C12—H12A	110.6
C6—C5—C7	117.2 (3)	C13—C12—H12A	110.6
C4—C5—C7	124.1 (3)	O4—C12—H12B	110.6
C8—C7—C5	121.5 (3)	C13—C12—H12B	110.6
C8—C7—H7	119.2	H12A—C12—H12B	108.7
C5—C7—H7	119.2	C9—N1—C11	126.2 (2)
N1—C13—C12	100.1 (2)	C9—N1—C13	122.0 (2)
N1—C13—C14	111.8 (2)	C11—N1—C13	111.7 (2)

Document origin: *publCIF* [Westrip, S. P. (2010). *J. Apply. Cryst.*, **43**, 920-925].

A 1:1 diastereomeric mixture of *syn*-HT **2BE** and **2BF**

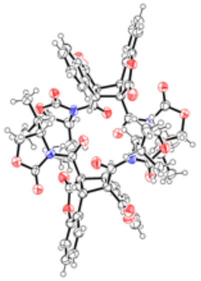

Crystal data

$C_{32}H_{30}N_2O_{10}$	D_x = 1.420 Mg m^{-3}
M_r = 602.60	Cu *K*a radiation, l = 1.54187 Å
Orthorhombic, $P2_12_12$	Cell parameters from 21682 reflections
a = 20.0542 (4) Å	q = 3.6–68.2°
b = 11.4435 (2) Å	m = 0.89 mm^{-1}
c = 12.2782 (2) Å	*T* = 93 K
V = 2817.72 (9) Å^3	Block, colorless
Z = 4	0.20 × 0.20 × 0.10 mm
F(000) = 1264.00	

Data collection

Rigaku R-AXIS RAPID diffractometer	3488 reflections with $F^2 > 2.0s(F^2)$
Detector resolution: 10.000 pixels mm^{-1}	R_{int} = 0.055
w scans	q_{max} = 68.2°, q_{min} = 3.6°
Absorption correction: multi-scan *ABSCOR* (Rigaku, 1995)	*h* = -23®24
T_{min} = 0.801, T_{max} = 0.914	*k* = -13®13
31475 measured reflections	*l* = -14®14
5177 independent reflections	

Refinement

Refinement on F^2	H-atom parameters constrained
$R[F^2 > 2s(F^2)]$ = 0.089	$w = 1/[s^2(F_o^2) + (0.1768P)^2]$ where $P = (F_o^2 + 2F_c^2)/3$
$wR(F^2)$ = 0.302	$(D/s)_{max}$ < 0.001
S = 1.20	$D\rho_{max}$ = 0.97 e Å^{-3}
5177 reflections	$D\rho_{min}$ = -0.46 e Å^{-3}
398 parameters	Extinction correction: *SHELXL*
0 restraints	Extinction coefficient: 0.0064 (14)
Primary atom site location: structure-invariant direct methods	Absolute structure: Flack x determined using 1073 quotients [(I+)-(I-)]/[(I+)+(I-)] (Parsons and Flack (2004), Acta Cryst. A60, s61).
Secondary atom site location: difference Fourier map	Absolute structure parameter: -0.12 (8)
Hydrogen site location: inferred from neighbouring sites	

Special details

Geometry. ENTER SPECIAL DETAILS OF THE MOLECULAR GEOMETRY
Refinement. Refinement was performed using all reflections. The weighted R-factor (wR) and goodness of fit (S) are based on F^2. R-factor (gt) are based on F. The threshold expression of F^2 > 2.0 sigma (F^2) is used only for calculating R-factor (gt).

Fractional atomic coordinates and isotropic or equivalent isotropic displacement parameters (Å^2) for (Ken20181211)

	x	*y*	*z*	U_{iso}*/U_{eq}
O1	0.5965 (3)	0.4448 (5)	0.5765 (4)	0.0430 (14)
O2	0.6693 (3)	1.0814 (5)	0.1043 (5)	0.0464 (15)
O3	0.6762 (3)	0.5385 (5)	0.4048 (5)	0.0431 (14)
O4	0.6039 (3)	0.9578 (5)	-0.0764 (4)	0.0428 (14)
O5	0.6103 (3)	0.2919 (5)	0.4689 (5)	0.0492 (15)
O6	0.7480 (3)	0.4624 (5)	0.2830 (4)	0.0429 (14)
O7	0.5367 (3)	0.3658 (5)	0.2133 (5)	0.0459 (15)
O8	0.5386 (3)	0.8706 (5)	0.2819 (5)	0.0471 (15)
O9	0.7224 (3)	1.0901 (6)	0.2652 (5)	0.0489 (16)
O35	0.6301 (3)	0.8210 (5)	0.0408 (5)	0.0507 (16)
N36	0.6317 (3)	0.9791 (6)	0.2570 (5)	0.0398 (16)
N37	0.6389 (3)	0.4296 (7)	0.2557 (5)	0.0443 (18)
C3	0.6736 (4)	1.0525 (8)	0.1980 (7)	0.0412 (19)
C4	0.5983 (4)	0.9087 (7)	0.0247 (7)	0.0394 (18)
C5	0.5470 (4)	0.4485 (7)	0.3925 (6)	0.0346 (17)
C6	0.5418 (4)	1.1344 (8)	-0.0212 (7)	0.0421 (19)
C7	0.5794 (4)	1.0713 (7)	-0.0973 (7)	0.0415 (19)
C8	0.5824 (4)	0.5625 (8)	0.5906 (7)	0.0410 (19)
C9	0.5489 (4)	0.9573 (7)	0.1036 (6)	0.0369 (18)
C10	0.5724 (4)	0.4123 (8)	0.2815 (7)	0.0424 (19)
C11	0.6858 (4)	0.4825 (8)	0.3224 (6)	0.0411 (19)
C12	0.5878 (4)	0.3908 (8)	0.4797 (7)	0.0412 (19)
C13	0.5244 (4)	1.2493 (7)	-0.0506 (7)	0.044 (2)
C14	0.6861 (4)	0.8301 (7)	0.3753 (7)	0.0407 (19)
C15	0.5986 (5)	1.1167 (8)	-0.1967 (7)	0.048 (2)
C16	0.5246 (4)	1.0875 (7)	0.0875 (7)	0.0378 (18)
C17	0.5519 (4)	0.6301 (7)	0.5125 (7)	0.0395 (19)
C18	0.6030 (4)	0.6080 (8)	0.6893 (7)	0.048 (2)
C19	0.5723 (4)	0.9319 (8)	0.2203 (7)	0.043 (2)
C20	0.5797 (5)	1.2292 (9)	-0.2237 (8)	0.057 (3)
C21	0.5934 (5)	0.7274 (9)	0.7125 (8)	0.051 (2)
C22	0.5418 (4)	0.7491 (7)	0.5370 (7)	0.0414 (19)
C23	0.7086 (4)	1.0532 (8)	0.3765 (7)	0.044 (2)
C24	0.6705 (4)	0.3824 (9)	0.1573 (7)	0.048 (2)
C25	0.7381 (4)	0.8039 (8)	0.2882 (8)	0.048 (2)
C26	0.5426 (4)	1.2974 (8)	-0.1498 (7)	0.047 (2)
C27	0.6586 (4)	0.9529 (8)	0.3657 (7)	0.0418 (19)
C28	0.5620 (4)	0.7955 (9)	0.6351 (7)	0.051 (2)
C29	0.7429 (4)	0.3770 (9)	0.1948 (7)	0.048 (2)
C30	0.7146 (5)	0.8114 (9)	0.4894 (7)	0.051 (2)
C31	0.6916 (5)	0.3955 (13)	-0.0406 (8)	0.077 (4)
C32	0.6600 (5)	0.4553 (12)	0.0569 (8)	0.066 (3)
C33	0.6822 (8)	0.5720 (13)	0.0670 (11)	0.103 (5)
C38	0.5313 (4)	0.5809 (7)	0.4058 (6)	0.0386 (18)
H13	0.49937	1.29529	-0.00083	0.0528*
H14	0.64831	0.77418	0.36534	0.0488*
H15	0.62437	1.07126	-0.24588	0.0570*
H16	0.53944	1.14007	0.14786	0.0454*
H18	0.6237	0.55876	0.74153	0.0571*
H20	0.59176	1.26071	-0.29251	0.0686*
H21	0.60806	0.76004	0.77949	0.0617*
H22	0.52081	0.79821	0.48492	0.0496*
H23A	0.68921	1.11821	0.41924	0.0522*
H23B	0.74984	1.02622	0.41289	0.0522*

H24	0.65379	0.30132	0.14392	0.0581*
H25A	0.75458	0.72388	0.29738	0.0578*
H25B	0.71785	0.8121	0.21596	0.0578*
H25C	0.77533	0.85889	0.29517	0.0578*
H26	0.53022	1.37519	-0.16776	0.0569*
H27	0.62243	0.96355	0.42085	0.0502*
H28	0.55418	0.87589	0.64986	0.0610*
H29A	0.75413	0.29775	0.2213	0.0571*
H29B	0.7734	0.39753	0.1344	0.0571*
H30A	0.7324	0.7319	0.49534	0.0608*
H30B	0.75049	0.86788	0.50259	0.0608*
H30C	0.67929	0.82255	0.54358	0.0608*
H31A	0.67535	0.31489	-0.04547	0.0921*
H31B	0.67977	0.43783	-0.10717	0.0921*
H31C	0.74022	0.39506	-0.03199	0.0921*
H32	0.61083	0.45814	0.04326	0.0798*
H33A	0.6737	0.61395	-0.00125	0.1232*
H33B	0.65815	0.61041	0.12654	0.1232*
H33C	0.73011	0.57269	0.08254	0.1232*
H38	0.54868	0.62811	0.34344	0.0463*

Atomic displacement parameters (Å^2) for (Ken20181211)

	U^{11}	U^{22}	U^{33}	U^{12}	U^{13}	U^{23}
O1	0.041 (3)	0.051 (4)	0.037 (3)	-0.003 (3)	-0.006 (3)	0.002 (3)
O2	0.038 (3)	0.054 (3)	0.047 (3)	-0.005 (3)	-0.004 (3)	0.010 (3)
O3	0.037 (3)	0.049 (3)	0.043 (3)	-0.001 (3)	0.001 (3)	-0.001 (3)
O4	0.044 (3)	0.044 (3)	0.041 (3)	0.005 (3)	0.001 (3)	0.001 (3)
O5	0.041 (3)	0.048 (3)	0.058 (4)	0.004 (3)	0.000 (3)	0.006 (3)
O6	0.034 (3)	0.052 (3)	0.043 (3)	-0.002 (3)	0.005 (2)	-0.007 (3)
O7	0.042 (3)	0.052 (4)	0.044 (3)	-0.005 (3)	0.002 (3)	-0.010 (3)
O8	0.038 (3)	0.056 (4)	0.048 (3)	-0.010 (3)	0.000 (3)	0.008 (3)
O9	0.041 (3)	0.057 (4)	0.049 (3)	-0.011 (3)	-0.004 (3)	0.010 (3)
O35	0.044 (3)	0.049 (4)	0.060 (4)	0.006 (3)	0.005 (3)	0.002 (3)
N36	0.037 (4)	0.044 (4)	0.038 (4)	-0.001 (3)	-0.010 (3)	0.008 (3)
N37	0.033 (4)	0.064 (5)	0.036 (4)	-0.005 (3)	0.002 (3)	-0.011 (4)
C3	0.029 (4)	0.047 (5)	0.047 (5)	-0.007 (4)	-0.011 (4)	0.003 (4)
C4	0.034 (4)	0.040 (4)	0.044 (4)	-0.003 (4)	0.000 (4)	0.000 (4)
C5	0.033 (4)	0.040 (4)	0.031 (4)	0.008 (3)	0.002 (3)	-0.001 (3)
C6	0.043 (4)	0.048 (5)	0.036 (4)	0.003 (4)	-0.005 (4)	-0.002 (4)
C7	0.044 (5)	0.040 (4)	0.041 (4)	-0.001 (4)	-0.001 (4)	0.003 (4)
C8	0.035 (4)	0.049 (5)	0.039 (4)	0.000 (4)	-0.001 (3)	0.004 (4)
C9	0.033 (4)	0.044 (4)	0.034 (4)	0.003 (4)	-0.005 (3)	-0.002 (3)
C10	0.037 (4)	0.045 (5)	0.044 (4)	-0.004 (4)	0.006 (4)	-0.001 (4)
C11	0.038 (4)	0.048 (5)	0.037 (4)	0.002 (4)	0.001 (4)	0.006 (4)
C12	0.037 (4)	0.044 (5)	0.042 (5)	-0.003 (4)	0.000 (4)	0.002 (4)
C13	0.041 (5)	0.039 (5)	0.051 (5)	-0.001 (4)	-0.009 (4)	0.006 (4)
C14	0.037 (4)	0.042 (5)	0.043 (5)	-0.002 (4)	-0.008 (4)	0.006 (4)
C15	0.048 (5)	0.057 (5)	0.038 (4)	-0.001 (4)	0.002 (4)	0.006 (4)
C16	0.032 (4)	0.037 (4)	0.044 (4)	0.002 (3)	-0.003 (3)	0.006 (4)
C17	0.037 (4)	0.040 (4)	0.042 (4)	-0.001 (4)	0.002 (4)	0.000 (4)
C18	0.042 (5)	0.067 (6)	0.034 (4)	-0.003 (4)	0.001 (4)	0.000 (4)
C19	0.036 (4)	0.056 (5)	0.038 (4)	-0.001 (4)	-0.004 (4)	0.003 (4)
C20	0.058 (6)	0.072 (7)	0.041 (5)	-0.015 (5)	-0.014 (4)	0.015 (5)

C21	0.041 (5)	0.070 (6)	0.044 (5)	-0.005 (4)	-0.006 (4)	-0.010 (5)
C22	0.038 (4)	0.046 (5)	0.040 (4)	-0.005 (4)	-0.003 (4)	-0.007 (4)
C23	0.041 (5)	0.045 (5)	0.045 (5)	0.001 (4)	-0.009 (4)	0.004 (4)
C24	0.045 (5)	0.059 (6)	0.042 (5)	-0.004 (4)	0.011 (4)	-0.013 (4)
C25	0.036 (4)	0.048 (5)	0.061 (6)	0.002 (4)	0.009 (4)	0.007 (4)
C26	0.044 (5)	0.048 (5)	0.050 (5)	-0.001 (4)	-0.009 (4)	0.012 (4)
C27	0.044 (5)	0.046 (5)	0.035 (4)	-0.001 (4)	-0.008 (4)	0.006 (4)
C28	0.045 (5)	0.059 (6)	0.048 (5)	-0.006 (4)	0.004 (4)	-0.013 (5)
C29	0.043 (5)	0.050 (5)	0.049 (5)	0.002 (4)	0.005 (4)	-0.011 (4)
C30	0.041 (5)	0.060 (6)	0.051 (5)	-0.001 (4)	-0.011 (4)	0.009 (5)
C31	0.055 (6)	0.137 (11)	0.039 (5)	0.009 (7)	0.003 (5)	-0.020 (6)
C32	0.058 (6)	0.096 (9)	0.045 (5)	0.022 (6)	0.006 (5)	-0.002 (6)
C33	0.145 (14)	0.084 (10)	0.079 (9)	0.017 (10)	0.027 (9)	0.008 (8)
C38	0.040 (4)	0.039 (4)	0.037 (4)	-0.004 (4)	0.003 (3)	-0.005 (4)

Geometric parameters (Å, º) for (Ken20181211)

O1—C8	1.387 (10)	C17—C38	1.483 (11)
O1—C12	1.351 (10)	C18—C21	1.408 (14)
O2—C3	1.201 (10)	C20—C26	1.409 (14)
O3—C11	1.214 (10)	C21—C28	1.381 (13)
O4—C4	1.368 (10)	C22—C28	1.378 (12)
O4—C7	1.412 (10)	C23—C27	1.529 (12)
O5—C12	1.225 (10)	C24—C29	1.523 (12)
O6—C11	1.357 (10)	C24—C32	1.504 (14)
O6—C29	1.462 (11)	C31—C32	1.518 (15)
O7—C10	1.223 (10)	C32—C33	1.41 (2)
O8—C19	1.233 (10)	C13—H13	0.950
O9—C3	1.351 (10)	C14—H14	1.000
O9—C23	1.457 (10)	C15—H15	0.950
O35—C4	1.205 (10)	C16—H16	1.000
N36—C3	1.390 (11)	C18—H18	0.950
N36—C19	1.384 (11)	C20—H20	0.950
N36—C27	1.470 (10)	C21—H21	0.950
N37—C10	1.385 (11)	C22—H22	0.950
N37—C11	1.386 (11)	C23—H23A	0.990
N37—C24	1.468 (11)	C23—H23B	0.990
C4—C9	1.493 (11)	C24—H24	1.000
C5—C10	1.513 (11)	C25—H25A	0.980
C5—C12	1.501 (11)	C25—H25B	0.980
C5—C38	1.557 (11)	C25—H25C	0.980
C5—C38^{i}	1.614 (11)	C26—H26	0.950
C6—C7	1.402 (12)	C27—H27	1.000
C6—C13	1.406 (12)	C28—H28	0.950
C6—C16	1.479 (12)	C29—H29A	0.990
C7—C15	1.382 (12)	C29—H29B	0.990
C8—C17	1.377 (12)	C30—H30A	0.980
C8—C18	1.381 (12)	C30—H30B	0.980
C9—C16	1.581 (11)	C30—H30C	0.980
C9—C16^{ii}	1.574 (11)	C31—H31A	0.980
C9—C19	1.536 (11)	C31—H31B	0.980
C13—C26	1.386 (12)	C31—H31C	0.980
C14—C25	1.523 (12)	C32—H32	1.000

C14—C27	1.515 (12)	C33—H33A	0.980
C14—C30	1.528 (12)	C33—H33B	0.980
C15—C20	1.382 (14)	C33—H33C	0.980
C17—C22	1.410 (11)	C38—H38	1.000
O1···O3	2.854 (8)	H31A···H33C	3.5185
O1···C8^{i}	3.594 (10)	H31B···H32	2.3188
O1···C17^{i}	3.194 (10)	H31B···H33A	2.4017
O1···C22^{i}	3.585 (10)	H31B···H33B	3.5103
O1···C38	2.921 (10)	H31B···H33C	2.9709
O1···C38^{i}	3.323 (10)	H31C···H32	2.8474
O2···O4	2.940 (8)	H31C···H33A	2.8629
O2···O35	3.179 (9)	H31C···H33B	3.5455
O2···C4	2.625 (10)	H31C···H33C	2.4800
O2···C6	3.048 (10)	H32···H33A	2.2511
O2···C7	3.065 (10)	H32···H33B	2.2321
O2···C9	2.801 (10)	H32···H33C	2.7700
O2···C16	2.911 (9)	H33B···H38	3.4573
O2···C19	2.957 (10)	H38···H38^{i}	3.5227
O2···C23	3.449 (10)	O1···H20^{iii}	2.6519
O2···C27	3.537 (10)	O1···H23B^{vi}	3.2220
O3···O5	3.214 (8)	O1···H25C^{vi}	3.1709
O3···C5	2.792 (9)	O1···H26^{iii}	3.5013
O3···C8	2.968 (10)	O1···H30B^{vi}	3.3367
O3···C10	2.951 (10)	O2···H24^{iv}	2.5824
O3···C12	2.617 (10)	O2···H29A^{iv}	3.3299
O3···C17	3.010 (10)	O2···H31A^{iv}	3.2457
O3···C24	3.527 (10)	O2···H31B^{v}	3.4435
O3···C29	3.443 (10)	O2···H31C^{v}	2.9366
O3···C38	2.946 (10)	O2···H33A^{v}	3.4129
O4···C6^{ii}	3.179 (10)	O2···H33C^{v}	3.0556
O4···C13^{ii}	3.511 (10)	O3···H14	2.7963
O4···C16	2.964 (10)	O3···H23A^{vi}	3.5762
O4···C16^{ii}	3.310 (9)	O3···H23B^{vi}	2.6894
O5···O7	3.569 (8)	O3···H25A	2.9518
O5···N37	3.109 (9)	O3···H30A	2.7212
O5···C8	3.483 (10)	O3···H30B^{vi}	2.6963
O5···C10	2.787 (11)	O4···H21^{xiii}	2.8733
O5···C11	3.207 (10)	O4···H29B^{v}	2.6528
O5···C17^{i}	3.414 (10)	O4···H31C^{v}	3.4729
O5···C22^{i}	3.198 (10)	O4···H33C^{v}	3.5801
O5···C38^{i}	3.282 (10)	O5···H20^{iii}	2.9748
O6···C10	3.568 (10)	O5···H23A^{vii}	2.6132
O6···C32	3.291 (11)	O5···H30A^{vi}	3.2590
O6···C33	3.217 (15)	O5···H30B^{vi}	2.9457
O7···O7^{i}	3.406 (8)	O6···H15^{xii}	2.8829
O7···C5^{i}	3.489 (9)	O6···H25A	2.9999
O7···C10^{i}	3.455 (10)	O6···H30B^{vi}	2.8462
O7···C11	3.537 (10)	O6···H30C^{vi}	3.0368
O7···C12	3.439 (10)	O7···H13^{vii}	2.8504
O7···C24	2.776 (10)	O7···H16^{vii}	2.7060
O7···C32	3.293 (12)	O8···H22	2.6511

O7···C38	3.415 (10)	O8···H38	2.8828
O7···C38^{i}	2.796 (10)	O9···H18^{viii}	3.1074
O8···O8^{ii}	3.342 (8)	O9···H24^{iv}	3.1551
O8···O35	3.528 (9)	O9···H29A^{iv}	2.5181
O8···C3	3.567 (10)	O9···H30A^{viii}	3.4783
O8···C4	3.405 (10)	O9···H31B^{v}	3.2629
O8···C9^{ii}	3.428 (10)	O35···H21^{xiii}	3.3132
O8···C14	3.207 (10)	O35···H29B^{v}	3.0235
O8···C16	3.456 (10)	O35···H31C^{v}	2.7377
O8···C16^{ii}	2.745 (10)	O35···H33A	2.5782
O8···C19^{ii}	3.260 (10)	O35···H33B	2.6895
O8···C27	2.782 (10)	O35···H33C	3.5160
O9···C14	3.349 (10)	C3···H24^{iv}	2.9504
O9···C19	3.556 (10)	C3···H29A^{iv}	3.2506
O9···C25	3.302 (11)	C3···H31B^{v}	3.4083
O35···N36	3.212 (9)	C3···H31C^{v}	3.2236
O35···C3	3.392 (11)	C4···H21^{xiii}	3.4633
O35···C6^{ii}	3.566 (10)	C4···H29B^{v}	3.2328
O35···C7	3.480 (10)	C4···H31C^{v}	3.2431
O35···C13^{ii}	3.391 (10)	C6···H24^{iv}	3.5786
O35···C16^{ii}	3.323 (10)	C6···H31A^{iv}	3.3956
O35···C19	2.794 (11)	C7···H29B^{v}	3.5885
N36···C4	3.039 (10)	C7···H31A^{iv}	3.4473
N36···C16	3.239 (10)	C8···H23B^{vi}	3.3893
N36···C25	2.953 (11)	C10···H16^{vii}	3.5825
N37···C12	2.969 (11)	C11···H14	3.4623
N37···C33	2.963 (16)	C11···H23B^{vi}	3.5328
N37···C38	3.324 (11)	C11···H25A	3.1030
C3···C4	3.085 (12)	C11···H30B^{vi}	2.8229
C3···C9	2.963 (11)	C12···H20^{iii}	3.1691
C3···C14	3.359 (12)	C12···H30B^{vi}	3.2616
C3···C16	3.306 (11)	C13···H21^{x}	3.3790
C3···C25	3.316 (13)	C13···H24^{iv}	3.5769
C4···C6	2.877 (12)	C13···H31A^{iv}	3.1200
C4···C6^{ii}	2.907 (12)	C13···H32^{iv}	3.1698
C4···C9^{ii}	3.465 (11)	C14···H22	3.5967
C4···C13^{ii}	3.190 (11)	C15···H25A^{v}	3.4215
C5···C8	2.850 (11)	C15···H28^{xiii}	3.4544
C5···C8^{i}	3.560 (11)	C15···H28^{x}	3.5967
C5···C10^{i}	3.182 (11)	C15···H31A^{iv}	3.3114
C5···C11	2.940 (11)	C17···H14	3.1183
C5···C12^{i}	3.440 (11)	C17···H30C	3.3953
C5···C22^{i}	3.381 (11)	C18···H23B^{vi}	3.3405
C6···C6^{ii}	3.503 (12)	C18···H26^{iii}	3.5090
C6···C7^{ii}	3.509 (12)	C18···H26^{xi}	3.2026
C6···C16^{ii}	3.163 (12)	C18···H30C	3.4007
C6···C20	2.817 (13)	C18···H31B^{xiv}	3.5231
C7···C7^{ii}	3.577 (12)	C20···H25A^{v}	3.4456
C7···C9	2.856 (11)	C20···H25C^{v}	3.3808
C7···C9^{ii}	3.579 (11)	C20···H28^{x}	3.3255
C7···C16^{ii}	3.577 (11)	C20···H31A^{iv}	3.0716
C7···C26	2.767 (12)	C20···H31B^{iv}	3.4321

C8···C28	2.753 (13)	C21···H26^{XI}	3.1118
C9···C13^{II}	3.366 (11)	C21···H29A^{VIII}	3.2650
C9···C19^{II}	3.094 (11)	C21···H30C	2.9080
C10···C10^{I}	3.529 (12)	C22···H14	3.0139
C10···C32	3.306 (13)	C22···H20^{XI}	3.4019
C11···C12	2.949 (12)	C22···H27	3.2661
C11···C32	3.315 (13)	C22···H30C	2.8829
C11···C33	3.300 (16)	C23···H29A^{IV}	3.5074
C11···C38	3.453 (11)	C23···H30A^{VIII}	2.8395
C12···C17	2.860 (12)	C25···H20^{XII}	3.4478
C12···C17^{I}	2.839 (11)	C25···H31A^{V}	3.4510
C12···C18	3.591 (12)	C25···H31B^{V}	3.1624
C12···C22^{I}	3.132 (12)	C25···H31C^{V}	3.3425
C13···C15	2.781 (12)	C25···H33B	3.3784
C14···C19	3.192 (12)	C26···H21^{X}	3.2109
C17···C21	2.823 (12)	C26···H31A^{IV}	2.9618
C17···C38^{I}	3.213 (11)	C26···H31B^{IV}	3.2292
C18···C22	2.758 (12)	C26···H32^{IV}	3.2983
C19···C19^{II}	3.293 (12)	C27···H22	3.5933
C23···C25	3.108 (13)	C28···H20^{XI}	3.2729
C23···C30	3.097 (13)	C28···H27	3.4768
C29···C31	3.075 (13)	C28···H30C	2.6253
C29···C33	2.987 (17)	C29···H15^{XII}	3.5244
O1···C20^{III}	3.495 (11)	C29···H21^{VI}	3.2908
O2···C24^{IV}	3.506 (12)	C30···H23A^{VI}	3.1409
O2···C31^{V}	3.593 (13)	C30···H23B^{VI}	3.5494
O3···C14	3.362 (10)	C31···H18^{XIII}	3.5364
O3···C23^{VI}	3.547 (10)	C31···H25B^{XII}	2.9737
O3···C25	3.580 (11)	C31···H25C^{XII}	3.2228
O3···C30	3.381 (12)	C33···H25B	3.3771
O4···C29^{V}	3.523 (10)	C38···H14	3.2627
O5···C23^{VII}	3.555 (11)	H13···O7^{IV}	2.8504
O5···C30^{VI}	3.556 (11)	H13···H21^{X}	3.5097
O6···C30^{VI}	3.369 (11)	H13···H24^{IV}	3.5712
O7···O8^{I}	3.211 (8)	H13···H31A^{IV}	3.5785
O7···C13^{VII}	3.513 (10)	H13···H32^{IV}	2.9601
O7···C16^{VII}	3.548 (10)	H14···O3	2.7963
O8···O7^{I}	3.211 (8)	H14···C11	3.4623
O8···C22	3.428 (10)	H14···C17	3.1183
O9···C18^{VIII}	3.551 (10)	H14···C22	3.0139
O9···C29^{IV}	3.420 (12)	H14···C38	3.2627
O35···C33	3.052 (16)	H14···H22	2.9613
C13···O7^{IV}	3.513 (10)	H14···H33B	3.4854
C14···O3	3.362 (10)	H14···H38	2.6189
C16···O7^{IV}	3.548 (10)	H15···O6^{V}	2.8829
C18···O9^{VI}	3.551 (10)	H15···C29^{V}	3.5244
C20···O1^{IX}	3.495 (11)	H15···H21^{XIII}	3.5900
C20···C21^{X}	3.592 (14)	H15···H25A^{V}	3.0567
C20···C28^{X}	3.340 (13)	H15···H28^{XIII}	2.9357
C21···C20^{XI}	3.592 (14)	H15···H29B^{V}	3.1669
C21···C26^{XI}	3.220 (13)	H15···H33C^{V}	3.5410
C22···O8	3.428 (10)	H16···O7^{IV}	2.7060

C22···C30	3.586 (12)	H16···C10^{iv}	3.5825
C23···O3^{viii}	3.547 (10)	H16···H24^{iv}	2.9438
C23···O5^{iv}	3.555 (11)	H18···O9^{vi}	3.1074
C24···O2^{vii}	3.506 (12)	H18···C31^{xiv}	3.5364
C25···O3	3.580 (11)	H18···H20^{iii}	3.4954
C25···C31^{v}	3.511 (14)	H18···H23B^{vi}	3.1883
C26···C21^{x}	3.220 (13)	H18···H25C^{vi}	3.0878
C26···C28^{x}	3.535 (13)	H18···H26^{iii}	3.0278
C26···C31^{iv}	3.463 (14)	H18···H26^{xi}	3.3675
C28···C20^{xi}	3.340 (13)	H18···H31B^{xiv}	2.5750
C28···C26^{xi}	3.535 (13)	H18···H33A^{xiv}	3.3732
C28···C30	3.550 (13)	H20···O1^{ix}	2.6519
C29···O4^{xii}	3.523 (10)	H20···O5^{ix}	2.9748
C29···O9^{vii}	3.420 (12)	H20···C12^{ix}	3.1691
C30···O3	3.381 (12)	H20···C22^{x}	3.4019
C30···O5^{viii}	3.556 (11)	H20···C25^{v}	3.4478
C30···O6^{viii}	3.369 (11)	H20···C28^{x}	3.2729
C30···C22	3.586 (12)	H20···H18^{ix}	3.4954
C30···C28	3.550 (13)	H20···H25A^{v}	3.1108
C31···O2^{xii}	3.593 (13)	H20···H25C^{v}	2.8927
C31···C25^{xii}	3.511 (14)	H20···H28^{x}	3.3926
C31···C26^{vii}	3.463 (14)	H20···H31A^{iv}	3.5206
C33···O35	3.052 (16)	H20···H31B^{iv}	3.5216
O1···H18	2.4705	H21···O4^{xiv}	2.8733
O2···H16	2.7428	H21···O35^{xiv}	3.3132
O2···H25B	3.5105	H21···C4^{xiv}	3.4633
O3···H33B	3.5328	H21···C13^{xi}	3.3790
O3···H38	2.8554	H21···C26^{xi}	3.2109
O4···H15	2.4867	H21···C29^{viii}	3.2908
O5···H22^{i}	2.8304	H21···H13^{xi}	3.5097
O6···H24	3.1441	H21···H15^{xiv}	3.5900
O6···H33B	3.1312	H21···H26^{xi}	3.2410
O6···H33C	2.7892	H21···H29A^{viii}	2.7972
O7···H24	2.6041	H21···H29B^{viii}	3.0405
O7···H32	2.7719	H21···H30C	3.3079
O7···H38	3.4088	H21···H33A^{xiv}	3.4315
O7···H38^{i}	2.3435	H22···O8	2.6511
O8···H14	2.6662	H22···C14	3.5967
O8···H16	3.4955	H22···C27	3.5933
O8···H16^{ii}	2.2740	H22···H14	2.9613
O8···H27	2.6212	H22···H27	2.8900
O9···H25B	3.2395	H22···H30C	3.2707
O9···H25C	2.8742	H23A···O3^{viii}	3.5762
O9···H27	3.1261	H23A···O5^{iv}	2.6132
O35···H13^{ii}	2.9618	H23A···C30^{viii}	3.1409
O35···H25B	2.7808	H23A···H29A^{iv}	3.4384
N36···H14	2.7169	H23A···H30A^{viii}	2.2943
N36···H16	2.9348	H23A···H30B^{viii}	3.2475
N36···H23A	2.7981	H23A···H30C^{viii}	3.5540
N36···H23B	3.0925	H23B···O1^{viii}	3.2220
N36···H25B	2.6248	H23B···O3^{viii}	2.6894
N36···H25C	3.2257	H23B···C8^{viii}	3.3893

N37···H29A	2.7925	H23B···C11^{viii}	3.5328
N37···H29B	3.1033	H23B···C18^{viii}	3.3405
N37···H32	2.6881	H23B···C30^{viii}	3.5494
N37···H33B	2.6350	H23B···H18^{viii}	3.1883
N37···H33C	3.2476	H23B···H30A^{viii}	2.6337
N37···H38	3.0970	H24···O2^{vii}	2.5824
C3···H16	2.9359	H24···O9^{vii}	3.1551
C3···H23A	2.8355	H24···C3^{vii}	2.9504
C3···H23B	3.0641	H24···C6^{vii}	3.5786
C3···H25B	2.8993	H24···C13^{vii}	3.5769
C3···H25C	3.2398	H24···H13^{vii}	3.5712
C3···H27	3.0941	H24···H16^{vii}	2.9438
C4···H13^{ii}	3.0631	H25A···O3	2.9518
C4···H16	3.2701	H25A···O6	2.9999
C4···H16^{ii}	3.1983	H25A···C11	3.1030
C4···H25B	3.5332	H25A···C15^{xii}	3.4215
C5···H22^{i}	3.3326	H25A···C20^{xii}	3.4456
C6···H15	3.2978	H25A···H15^{xii}	3.0567
C6···H26	3.2986	H25A···H20^{xii}	3.1108
C7···H13	3.2482	H25A···H31A^{v}	3.5533
C7···H16	3.2131	H25A···H33B	3.1346
C7···H20	3.2413	H25A···H33C	3.1926
C8···H21	3.2791	H25B···C31^{v}	2.9737
C8···H22	3.2386	H25B···C33	3.3771
C8···H38	3.1989	H25B···H31A^{v}	2.9950
C9···H13^{ii}	3.3072	H25B···H31B^{v}	2.8407
C10···H24	2.6704	H25B···H31C^{v}	2.5905
C10···H32	3.0699	H25B···H33B	2.8224
C10···H33B	3.4228	H25B···H33C	3.2015
C10···H38	2.6273	H25C···O1^{viii}	3.1709
C10···H38^{i}	2.5861	H25C···C20^{xii}	3.3808
C11···H24	3.0840	H25C···C31^{v}	3.2228
C11···H29A	2.8081	H25C···H18^{viii}	3.0878
C11···H29B	3.0590	H25C···H20^{xii}	2.8927
C11···H33B	2.8695	H25C···H31A^{v}	3.2606
C11···H33C	3.2447	H25C···H31B^{v}	2.6372
C11···H38	3.2261	H25C···H31C^{v}	3.2727
C12···H22^{i}	3.0694	H26···O1^{ix}	3.5013
C12···H38	3.2849	H26···C18^{ix}	3.5090
C12···H38^{i}	3.2145	H26···C18^{x}	3.2026
C13···H16	2.7550	H26···C21^{x}	3.1118
C13···H20	3.2658	H26···H18^{ix}	3.0278
C14···H23A	3.3417	H26···H18^{x}	3.3675
C14···H23B	2.6235	H26···H21^{x}	3.2410
C15···H26	3.2794	H26···H26^{xv}	3.1030
C16···H13	2.6614	H26···H31A^{iv}	3.3469
C16···H16^{ii}	2.9972	H26···H31B^{iv}	3.1721
C17···H18	3.2637	H26···H32^{iv}	3.1980
C17···H28	3.2805	H27···C22	3.2661
C18···H28	3.2537	H27···C28	3.4768
C19···H14	2.9580	H27···H22	2.8900
C19···H16	2.6272	H27···H28	3.2842

C19···H16^{ii}	2.5480	H28···C15^{xiv}	3.4544
C19···H25B	3.2248	H28···C15^{xi}	3.5967
C19···H27	2.6840	H28···C20^{xi}	3.3255
C20···H13	3.2641	H28···H15^{xiv}	2.9357
C21···H22	3.2532	H28···H20^{xi}	3.3926
C22···H21	3.2625	H28···H27	3.2842
C22···H38	2.7539	H28···H28^{ii}	3.5764
C23···H14	3.4161	H28···H30C	2.8932
C23···H25B	3.3955	H29A···O2^{vii}	3.3299
C23···H25C	2.7807	H29A···O9^{vii}	2.5181
C23···H30B	2.7567	H29A···C3^{vii}	3.2506
C23···H30C	3.3936	H29A···C21^{vi}	3.2650
C24···H31A	2.6089	H29A···C23^{vii}	3.5074
C24···H31B	3.3143	H29A···H21^{vi}	2.7972
C24···H31C	2.7163	H29A···H23A^{vii}	3.4384
C24···H33A	3.2892	H29A···H30A^{vi}	3.5700
C24···H33B	2.6485	H29A···H30B^{vi}	3.4851
C24···H33C	2.6485	H29A···H30C^{vi}	3.1933
C25···H23B	2.9786	H29B···O4^{xii}	2.6528
C25···H27	3.3723	H29B···O35^{xii}	3.0235
C25···H30A	2.6760	H29B···C4^{xii}	3.2328
C25···H30B	2.7436	H29B···C7^{xii}	3.5885
C25···H30C	3.3570	H29B···H15^{xii}	3.1669
C26···H15	3.2829	H29B···H21^{vi}	3.0405
C27···H25A	3.3579	H30A···O3	2.7212
C27···H25B	2.7177	H30A···O5^{viii}	3.2590
C27···H25C	2.7179	H30A···O9^{vi}	3.4783
C27···H30A	3.3348	H30A···C23^{vi}	2.8395
C27···H30B	2.6776	H30A···H23A^{vi}	2.2943
C27···H30C	2.6775	H30A···H23B^{vi}	2.6337
C28···H18	3.2526	H30A···H29A^{viii}	3.5700
C29···H31A	3.3231	H30B···O1^{viii}	3.3367
C29···H31C	2.7928	H30B···O3^{viii}	2.6963
C29···H32	3.3668	H30B···O5^{viii}	2.9457
C29···H33B	3.2743	H30B···O6^{viii}	2.8462
C29···H33C	2.6417	H30B···C11^{viii}	2.8229
C30···H23B	2.7246	H30B···C12^{viii}	3.2616
C30···H25A	2.6845	H30B···H23A^{vi}	3.2475
C30···H25B	3.3585	H30B···H29A^{viii}	3.4851
C30···H25C	2.7325	H30C···O6^{viii}	3.0368
C30···H27	2.6755	H30C···C17	3.3953
C31···H24	2.6206	H30C···C18	3.4007
C31···H29B	2.7028	H30C···C21	2.9080
C31···H33A	2.5717	H30C···C22	2.8829
C31···H33B	3.2728	H30C···C28	2.6253
C31···H33C	2.6445	H30C···H21	3.3079
C32···H29A	3.3000	H30C···H22	3.2707
C32···H29B	2.5529	H30C···H23A^{vi}	3.5540
C33···H24	3.2886	H30C···H28	2.8932
C33···H29B	2.8319	H30C···H29A^{viii}	3.1933
C33···H31A	3.2533	H31A···O2^{vii}	3.2457
C33···H31B	2.6329	H31A···C6^{vii}	3.3956

C33···H31C	2.6329	H31A···C7^{vii}	3.4473
C38···H22	2.6775	H31A···C13^{vii}	3.1200
C38···H38^{i}	2.9801	H31A···C15^{vii}	3.3114
H13···H16	2.6709	H31A···C20^{vii}	3.0716
H13···H26	2.3280	H31A···C25^{xii}	3.4510
H14···H23B	3.5784	H31A···C26^{vii}	2.9618
H14···H25A	2.3600	H31A···H13^{vii}	3.5785
H14···H25B	2.3446	H31A···H20^{vii}	3.5206
H14···H25C	2.8584	H31A···H25A^{xii}	3.5533
H14···H27	2.3302	H31A···H25B^{xii}	2.9950
H14···H30A	2.3718	H31A···H25C^{xii}	3.2606
H14···H30B	2.8616	H31A···H26^{vii}	3.3469
H14···H30C	2.3413	H31A···H33C^{xii}	3.3887
H15···H20	2.3357	H31B···O2^{xii}	3.4435
H16···H16^{ii}	3.5748	H31B···O9^{xii}	3.2629
H18···H21	2.3709	H31B···C3^{xii}	3.4083
H20···H26	2.3634	H31B···C18^{xiii}	3.5231
H21···H28	2.3363	H31B···C20^{vii}	3.4321
H22···H28	2.3107	H31B···C25^{xii}	3.1624
H22···H38	2.6681	H31B···C26^{vii}	3.2292
H23A···H27	2.2195	H31B···H18^{xiii}	2.5750
H23A···H30B	3.2808	H31B···H20^{vii}	3.5216
H23B···H25B	3.5017	H31B···H25B^{xii}	2.8407
H23B···H25C	2.4530	H31B···H25C^{xii}	2.6372
H23B···H27	2.6556	H31B···H26^{vii}	3.1721
H23B···H30A	3.5342	H31C···O2^{xii}	2.9366
H23B···H30B	2.1205	H31C···O4^{xii}	3.4729
H23B···H30C	3.1637	H31C···O35^{xii}	2.7377
H24···H29A	2.2256	H31C···C3^{xii}	3.2236
H24···H29B	2.6419	H31C···C4^{xii}	3.2431
H24···H31A	2.3703	H31C···C25^{xii}	3.3425
H24···H31B	3.4952	H31C···H25B^{xii}	2.5905
H24···H31C	2.9698	H31C···H25C^{xii}	3.2727
H24···H32	2.3431	H32···C13^{vii}	3.1698
H24···H33B	3.5446	H32···C26^{vii}	3.2983
H24···H33C	3.5432	H32···H13^{vii}	2.9601
H25A···H30A	2.4727	H32···H26^{vii}	3.1980
H25A···H30B	3.0117	H33A···O2^{xii}	3.4129
H25A···H30C	3.5627	H33A···O35	2.5782
H25B···H30A	3.5629	H33A···H18^{xiii}	3.3732
H25C···H30A	2.9822	H33A···H21^{xiii}	3.4315
H25C···H30B	2.5970	H33B···O35	2.6895
H27···H30A	3.5675	H33B···C25	3.3784
H27···H30B	2.9667	H33B···H14	3.4854
H27···H30C	2.4848	H33B···H25A	3.1346
H29A···H31C	3.3150	H33B···H25B	2.8224
H29B···H31A	3.1045	H33C···O2^{xii}	3.0556
H29B···H31B	3.5406	H33C···O4^{xii}	3.5801
H29B···H31C	2.1488	H33C···O35	3.5160
H29B···H32	3.5160	H33C···H15^{xii}	3.5410
H29B···H33A	3.5924	H33C···H25A	3.1926
H29B···H33B	3.3594	H33C···H25B	3.2015

H29B···H33C	2.2753	H33C···H31A^{v}	3.3887
H31A···H32	2.3555	H38···O8	2.8828
H31A···H33A	3.4652	H38···H14	2.6189
C8—O1—C12	121.9 (6)	C24—C32—C31	109.7 (10)
C4—O4—C7	120.9 (6)	C24—C32—C33	114.1 (10)
C11—O6—C29	108.2 (6)	C31—C32—C33	111.3 (11)
C3—O9—C23	110.0 (6)	C5—C38—C5^{i}	89.0 (6)
C3—N36—C19	125.9 (7)	C5—C38—C17	113.9 (6)
C3—N36—C27	112.0 (6)	C5^{i}—C38—C17	116.1 (6)
C19—N36—C27	122.1 (7)	C6—C13—H13	118.948
C10—N37—C11	125.5 (7)	C26—C13—H13	118.948
C10—N37—C24	123.5 (7)	C25—C14—H14	107.884
C11—N37—C24	110.7 (7)	C27—C14—H14	107.884
O2—C3—O9	123.3 (7)	C30—C14—H14	107.880
O2—C3—N36	128.5 (7)	C7—C15—H15	120.454
O9—C3—N36	108.2 (7)	C20—C15—H15	120.461
O4—C4—O35	116.6 (7)	C6—C16—H16	112.437
O4—C4—C9	119.4 (7)	C9—C16—H16	112.449
O35—C4—C9	123.7 (7)	C9^{ii}—C16—H16	112.438
C10—C5—C12	109.8 (6)	C8—C18—H18	119.907
C10—C5—C38	115.4 (6)	C21—C18—H18	119.903
C10—C5—C38^{i}	111.2 (6)	C15—C20—H20	119.791
C12—C5—C38	117.6 (6)	C26—C20—H20	119.797
C12—C5—C38^{i}	111.5 (6)	C18—C21—H21	120.957
C38—C5—C38^{i}	89.7 (6)	C28—C21—H21	120.966
C7—C6—C13	116.4 (8)	C17—C22—H22	119.407
C7—C6—C16	122.6 (8)	C28—C22—H22	119.400
C13—C6—C16	120.8 (7)	O9—C23—H23A	110.717
O4—C7—C6	122.7 (7)	O9—C23—H23B	110.710
O4—C7—C15	114.2 (7)	C27—C23—H23A	110.718
C6—C7—C15	123.0 (8)	C27—C23—H23B	110.714
O1—C8—C17	123.3 (7)	H23A—C23—H23B	108.815
O1—C8—C18	114.5 (7)	N37—C24—H24	109.405
C17—C8—C18	122.1 (8)	C29—C24—H24	109.412
C4—C9—C16	118.4 (6)	C32—C24—H24	109.410
C4—C9—C16^{ii}	114.7 (6)	C14—C25—H25A	109.475
C4—C9—C19	109.4 (6)	C14—C25—H25B	109.461
C16—C9—C16^{ii}	90.1 (6)	C14—C25—H25C	109.475
C16—C9—C19	113.0 (7)	H25A—C25—H25B	109.476
C16^{ii}—C9—C19	110.0 (6)	H25A—C25—H25C	109.464
O7—C10—N37	118.0 (8)	H25B—C25—H25C	109.476
O7—C10—C5	122.6 (7)	C13—C26—H26	120.500
N37—C10—C5	119.4 (7)	C20—C26—H26	120.499
O3—C11—O6	122.2 (7)	N36—C27—H27	108.936
O3—C11—N37	127.9 (7)	C14—C27—H27	108.943
O6—C11—N37	109.9 (7)	C23—C27—H27	108.938
O1—C12—O5	118.1 (7)	C21—C28—H28	119.415
O1—C12—C5	119.8 (7)	C22—C28—H28	119.417
O5—C12—C5	122.0 (8)	O6—C29—H29A	110.680
C6—C13—C26	122.1 (8)	O6—C29—H29B	110.683
C25—C14—C27	112.1 (7)	C24—C29—H29A	110.682

C25—C14—C30	111.1 (7)	C24—C29—H29B	110.687
C27—C14—C30	109.7 (7)	H29A—C29—H29B	108.795
C7—C15—C20	119.1 (8)	C14—C30—H30A	109.474
C6—C16—C9	112.5 (7)	C14—C30—H30B	109.476
C6—C16—C9^{ii}	116.8 (7)	C14—C30—H30C	109.475
C9—C16—C9^{ii}	88.0 (6)	H30A—C30—H30B	109.456
C8—C17—C22	117.2 (7)	H30A—C30—H30C	109.476
C8—C17—C38	121.8 (7)	H30B—C30—H30C	109.471
C22—C17—C38	121.0 (7)	C32—C31—H31A	109.474
C8—C18—C21	120.2 (8)	C32—C31—H31B	109.465
O8—C19—N36	119.7 (7)	C32—C31—H31C	109.465
O8—C19—C9	120.8 (7)	H31A—C31—H31B	109.477
N36—C19—C9	119.5 (7)	H31A—C31—H31C	109.477
C15—C20—C26	120.4 (9)	H31B—C31—H31C	109.469
C18—C21—C28	118.1 (8)	C24—C32—H32	107.129
C17—C22—C28	121.2 (8)	C31—C32—H32	107.125
O9—C23—C27	105.1 (6)	C33—C32—H32	107.134
N37—C24—C29	100.3 (7)	C32—C33—H33A	109.465
N37—C24—C32	114.1 (8)	C32—C33—H33B	109.469
C29—C24—C32	113.8 (8)	C32—C33—H33C	109.478
C13—C26—C20	119.0 (8)	H33A—C33—H33B	109.462
N36—C27—C14	113.2 (7)	H33A—C33—H33C	109.482
N36—C27—C23	99.5 (6)	H33B—C33—H33C	109.472
C14—C27—C23	116.8 (7)	C5—C38—H38	111.985
C21—C28—C22	121.2 (9)	C5^{i}—C38—H38	111.981
O6—C29—C24	105.3 (7)	C17—C38—H38	111.989
C8—O1—C12—O5	169.4 (6)	C38—C5—C38^{i}—C5^{i}	12.0 (5)
C8—O1—C12—C5	-15.1 (10)	C38—C5—C38^{i}—C17^{i}	-104.3 (6)
C12—O1—C8—C17	7.5 (11)	C38^{i}—C5—C38—C5^{i}	-11.6 (5)
C12—O1—C8—C18	-171.1 (6)	C38^{i}—C5—C38—C17	106.7 (6)
C4—O4—C7—C6	-7.4 (11)	C7—C6—C13—C26	0.8 (12)
C4—O4—C7—C15	169.4 (6)	C13—C6—C7—O4	176.0 (7)
C7—O4—C4—O35	-167.8 (6)	C13—C6—C7—C15	-0.5 (12)
C7—O4—C4—C9	18.7 (10)	C7—C6—C16—C9	-4.0 (11)
C11—O6—C29—C24	20.8 (8)	C7—C6—C16—C9^{ii}	-103.6 (9)
C29—O6—C11—O3	170.6 (7)	C16—C6—C7—O4	0.0 (12)
C29—O6—C11—N37	-8.9 (8)	C16—C6—C7—C15	-176.5 (7)
C3—O9—C23—C27	19.3 (8)	C13—C6—C16—C9	-179.8 (7)
C23—O9—C3—O2	172.0 (7)	C13—C6—C16—C9^{ii}	80.5 (9)
C23—O9—C3—N36	-7.5 (8)	C16—C6—C13—C26	176.9 (7)
C3—N36—C19—O8	-177.8 (7)	O4—C7—C15—C20	-177.4 (6)
C3—N36—C19—C9	1.7 (12)	C6—C7—C15—C20	-0.5 (13)
C19—N36—C3—O2	-6.0 (14)	O1—C8—C17—C22	-178.8 (6)
C19—N36—C3—O9	173.4 (7)	O1—C8—C17—C38	0.1 (12)
C3—N36—C27—C14	-105.9 (7)	O1—C8—C18—C21	178.2 (6)
C3—N36—C27—C23	18.8 (8)	C17—C8—C18—C21	-0.4 (12)
C27—N36—C3—O2	172.4 (8)	C18—C8—C17—C22	-0.2 (11)
C27—N36—C3—O9	-8.2 (9)	C18—C8—C17—C38	178.6 (7)
C19—N36—C27—C14	72.6 (9)	C4—C9—C16—C6	14.6 (9)
C19—N36—C27—C23	-162.7 (7)	C4—C9—C16—C9^{ii}	132.8 (6)
C27—N36—C19—O8	3.9 (12)	C4—C9—C16^{ii}—C6^{ii}	-21.5 (9)

C27—N36—C19—C9	-176.6 (6)	C4—C9—C16^{ii}—C9^{ii}	-135.9 (6)
C10—N37—C11—O3	-12.9 (14)	C4—C9—C19—O8	-117.9 (8)
C10—N37—C11—O6	166.7 (7)	C4—C9—C19—N36	62.6 (9)
C11—N37—C10—O7	178.9 (7)	C16—C9—C16^{ii}—C6^{ii}	100.1 (6)
C11—N37—C10—C5	-2.5 (12)	C16—C9—C16^{ii}—C9^{ii}	-14.3 (5)
C10—N37—C24—C29	-155.1 (7)	C16^{ii}—C9—C16—C6	-103.9 (6)
C10—N37—C24—C32	82.9 (10)	C16^{ii}—C9—C16—C9^{ii}	14.4 (5)
C24—N37—C10—O7	-7.9 (12)	C16—C9—C19—O8	108.0 (8)
C24—N37—C10—C5	170.7 (7)	C16—C9—C19—N36	-71.5 (9)
C11—N37—C24—C29	19.0 (9)	C19—C9—C16—C6	144.2 (6)
C11—N37—C24—C32	-103.1 (8)	C19—C9—C16—C9^{ii}	-97.5 (6)
C24—N37—C11—O3	173.2 (8)	C16^{ii}—C9—C19—O8	8.9 (10)
C24—N37—C11—O6	-7.2 (9)	C16^{ii}—C9—C19—N36	-170.6 (6)
O4—C4—C9—C16	-22.5 (10)	C19—C9—C16^{ii}—C6^{ii}	-145.4 (6)
O4—C4—C9—C16^{ii}	82.1 (8)	C19—C9—C16^{ii}—C9^{ii}	100.3 (6)
O4—C4—C9—C19	-153.7 (6)	C6—C13—C26—C20	0.0 (12)
O35—C4—C9—C16	164.5 (7)	C25—C14—C27—N36	54.4 (9)
O35—C4—C9—C16^{ii}	-91.0 (9)	C25—C14—C27—C23	-60.3 (9)
O35—C4—C9—C19	33.2 (10)	C30—C14—C27—N36	178.4 (6)
C10—C5—C12—O1	149.8 (7)	C30—C14—C27—C23	63.7 (8)
C10—C5—C12—O5	-34.9 (10)	C7—C15—C20—C26	1.3 (14)
C12—C5—C10—O7	123.1 (8)	C8—C17—C22—C28	0.1 (11)
C12—C5—C10—N37	-55.4 (9)	C8—C17—C38—C5	0.4 (10)
C10—C5—C38—C5^{i}	101.9 (6)	C8—C17—C38—C5^{i}	101.8 (8)
C10—C5—C38—C17	-139.9 (6)	C22—C17—C38—C5	179.3 (6)
C38—C5—C10—O7	-101.1 (9)	C22—C17—C38—C5^{i}	-79.4 (9)
C38—C5—C10—N37	80.4 (9)	C38—C17—C22—C28	-178.8 (6)
C10—C5—C38^{i}—C5^{i}	-105.3 (6)	C8—C18—C21—C28	1.2 (12)
C10—C5—C38^{i}—C17^{i}	138.4 (6)	C15—C20—C26—C13	-1.1 (14)
C38^{i}—C5—C10—O7	-0.7 (10)	C18—C21—C28—C22	-1.3 (13)
C38^{i}—C5—C10—N37	-179.3 (6)	C17—C22—C28—C21	0.7 (12)
C12—C5—C38—C5^{i}	-125.8 (6)	O9—C23—C27—N36	-21.8 (7)
C12—C5—C38—C17	-7.6 (9)	O9—C23—C27—C14	100.4 (7)
C38—C5—C12—O1	15.1 (10)	N37—C24—C29—O6	-23.2 (8)
C38—C5—C12—O5	-169.7 (7)	N37—C24—C32—C31	-177.1 (7)
C12—C5—C38^{i}—C5^{i}	131.8 (6)	N37—C24—C32—C33	57.2 (10)
C12—C5—C38^{i}—C17^{i}	15.5 (9)	C29—C24—C32—C31	68.6 (10)
C38^{i}—C5—C12—O1	-86.5 (8)	C29—C24—C32—C33	-57.1 (11)
C38^{i}—C5—C12—O5	88.8 (9)	C32—C24—C29—O6	99.1 (9)

Symmetry codes: (i) -*x*+1, -*y*+1, *z*; (ii) -*x*+1, -*y*+2, *z*; (iii) *x*, *y*-1, *z*+1; (iv) *x*, *y*+1, *z*; (v) -*x*+3/2, *y*+1/2, -*z*; (vi) -*x*+3/2, *y*-1/2, -*z*+1; (vii) *x*, *y*-1, *z*; (viii) -*x*+3/2, *y*+1/2, -*z*+1; (ix) *x*, *y*+1, *z*-1; (x) -*x*+1, -*y*+2, *z*-1; (xi) -*x*+1, -*y*+2, *z*+1; (xii) -*x*+3/2, *y*-1/2, -*z*; (xiii) *x*, *y*, *z*-1; (xiv) *x*, *y*, *z*+1; (xv) -*x*+1, -*y*+3, *z*.

Document origin: *publCIF* [Westrip, S. P. (2010). *J. Apply. Cryst.*, **43**, 920-925].

syn-HT **2CE**

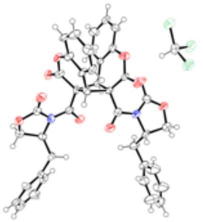

Crystal data

$C_{40}H_{29}Cl_3N_2O_{10}$	*Z* = 4
M_r = 804.00	*F*(000) = 1656
Orthorhombic, $P2_12_12_1$	D_x = 1.489 Mg m^{-3}
a = 10.620 (5) Å	Mo *K*a radiation, l = 0.71073 Å
b = 15.369 (6) Å	m = 0.32 mm^{-1}
c = 21.972 (9) Å	*T* = 296 K
V = 3586 (3) Å^3	

Data collection

17425 measured reflections	q_{max} = 25.1°, q_{min} = 1.6°
6314 independent reflections	*h* = -12®12
3951 reflections with *I* > 2s(*I*)	*k* = -16®18
R_{int} = 0.106	*l* = -26®24

Refinement

Refinement on F^2	H-atom parameters constrained
Least-squares matrix: full	$w = 1/[s^2(F_o^2) + (0.1674P)^2 + 5.378P]$ where $P = (F_o^2 + 2F_c^2)/3$
$R[F^2 > 2s(F^2)]$ = 0.096	$(D/s)_{max}$ < 0.001
$wR(F^2)$ = 0.301	$D\rho_{max}$ = 0.62 e Å^{-3}
S = 1.07	$D\rho_{min}$ = -0.55 e Å^{-3}
6314 reflections	Extinction correction: *SHELXL*, Fc*=kFc[1+0.001xFc^{2}l^3/sin(2q)]$^{-1/4}$
506 parameters	Extinction coefficient: 0.020 (4)
0 restraints	Absolute structure: Flack x determined using 1261 quotients [(I+)-(I-)]/[(I+)+(I-)] (Parsons and Flack (2004), Acta Cryst. A60, s61).
Hydrogen site location: inferred from neighbouring sites	Absolute structure parameter: -0.02 (8)

Special details

Geometry. All esds (except the esd in the dihedral angle between two l.s. planes) are estimated using the full covariance matrix. The cell esds are taken into account individually in the estimation of esds in distances, angles and torsion angles; correlations between esds in cell parameters are only used when they are defined by crystal symmetry. An approximate (isotropic) treatment of cell esds is used for estimating esds involving l.s. planes.

Fractional atomic coordinates and isotropic or equivalent isotropic displacement parameters (Å^2) for (p212121_a)

	x	*y*	*z*	U_{iso}*/U_{eq}
Cl01	0.6939 (4)	0.1918 (2)	0.8074 (2)	0.0529 (11)
Cl02	0.8089 (4)	0.3552 (2)	0.8394 (2)	0.0546 (11)
Cl03	0.9564 (4)	0.1990 (2)	0.8417 (2)	0.0538 (11)
O004	0.8981 (9)	0.5305 (5)	0.5295 (4)	0.034 (2)
O005	1.0319 (10)	0.4834 (6)	0.4628 (4)	0.037 (2)
O006	0.7458 (10)	0.4661 (6)	0.4271 (5)	0.041 (2)

O007	0.9756 (10)	0.2529 (6)	0.4485 (4)	0.039 (2)
O008	0.7880 (10)	0.4438 (6)	0.3288 (4)	0.039 (2)
O009	0.9667 (10)	0.0656 (5)	0.6654 (5)	0.039 (2)
O00A	0.9838 (10)	0.2106 (6)	0.6637 (5)	0.042 (2)
O00B	0.8439 (8)	0.3642 (6)	0.6806 (4)	0.036 (2)
O00C	0.7437 (9)	0.1835 (6)	0.5174 (5)	0.038 (2)
O00D	0.7024 (9)	0.2650 (6)	0.6662 (4)	0.039 (2)
N00E	0.8721 (11)	0.3527 (7)	0.3949 (5)	0.035 (3)
C1	0.7465 (14)	0.2108 (8)	0.2162 (6)	0.033 (3)
H1A	0.6705	0.2379	0.2254	0.040*
C00G	0.7958 (15)	0.4265 (9)	0.3873 (6)	0.035 (3)
N00H	0.8638 (11)	0.1352 (7)	0.5940 (5)	0.035 (3)
C00I	0.8338 (13)	0.1984 (8)	0.2613 (6)	0.031 (3)
C00J	0.9474 (14)	0.4666 (8)	0.4966 (7)	0.034 (3)
C00K	0.8328 (13)	0.2938 (8)	0.5822 (6)	0.028 (3)
C00L	1.1351 (13)	0.4244 (8)	0.6006 (7)	0.033 (3)
H00A	1.1804	0.4119	0.5655	0.040*
C00M	0.9207 (14)	0.3227 (8)	0.4480 (6)	0.033 (3)
C00N	1.1865 (15)	0.4805 (10)	0.6442 (7)	0.042 (4)
H00B	1.2631	0.5079	0.6367	0.051*
C00O	1.1245 (14)	0.4951 (8)	0.6974 (7)	0.036 (3)
H00C	1.1605	0.5304	0.7270	0.043*
C00P	1.0161 (12)	0.3867 (7)	0.6092 (6)	0.025 (3)
C00Q	0.9614 (13)	0.3277 (7)	0.5623 (6)	0.029 (3)
H00D	1.0191	0.2809	0.5506	0.035*
C00R	0.7901 (13)	0.3039 (8)	0.6450 (6)	0.031 (3)
C00S	0.7691 (15)	0.1840 (8)	0.1580 (7)	0.043 (4)
H00E	0.7109	0.1972	0.1278	0.052*
C00T	0.8986 (13)	0.3121 (8)	0.3364 (6)	0.032 (3)
H00F	0.9879	0.2962	0.3339	0.039*
C00U	0.5710 (14)	0.5163 (10)	0.6293 (6)	0.039 (3)
H00G	0.4971	0.5157	0.6520	0.047*
C00V	0.8460 (14)	0.0438 (8)	0.5774 (6)	0.031 (3)
H00H	0.7569	0.0320	0.5693	0.037*
C00W	0.6115 (14)	0.4403 (8)	0.5997 (6)	0.034 (3)
H00I	0.5639	0.3897	0.6025	0.041*
C00X	0.8093 (13)	0.2017 (8)	0.5606 (6)	0.030 (3)
C00Y	0.7210 (13)	0.4405 (8)	0.5665 (6)	0.031 (3)
C00Z	0.9002 (13)	0.3742 (8)	0.5059 (6)	0.028 (3)
C010	0.7884 (12)	0.5189 (8)	0.5630 (6)	0.032 (3)
C011	0.9414 (14)	0.1418 (9)	0.6431 (6)	0.036 (3)
C012	0.8718 (15)	0.3893 (9)	0.2955 (6)	0.037 (3)
H01A	0.9490	0.4202	0.2860	0.044*
H01B	0.8331	0.3704	0.2578	0.044*
C013	0.7702 (12)	0.3622 (8)	0.5360 (6)	0.029 (3)
H01C	0.7085	0.3352	0.5088	0.035*
C014	0.9085 (13)	-0.0757 (8)	0.5066 (7)	0.034 (3)
C015	0.8161 (14)	0.2321 (8)	0.3246 (6)	0.030 (3)
H01D	0.8373	0.1868	0.3535	0.037*
H01E	0.7283	0.2474	0.3306	0.037*
C016	0.6385 (15)	0.5892 (9)	0.6250 (7)	0.044 (4)
H01F	0.6107	0.6384	0.6456	0.053*
C017	0.9581 (13)	0.4038 (8)	0.6645 (6)	0.031 (3)
C018	1.0069 (14)	0.4570 (9)	0.7078 (6)	0.035 (3)
H01G	0.9630	0.4679	0.7436	0.042*
C019	0.7817 (18)	-0.1873 (12)	0.4651 (9)	0.064 (5)
H01H	0.7056	-0.2039	0.4477	0.077*

C01A	0.9466 (15)	0.1536 (9)	0.2455 (7)	0.041 (4)
H01I	1.0093	0.1451	0.2745	0.049*
C01B	0.7455 (14)	0.5946 (8)	0.5921 (6)	0.036 (3)
H01J	0.7893	0.6468	0.5887	0.044*
C01C	0.8860 (16)	0.0016 (9)	0.6366 (7)	0.043 (4)
H01K	0.8134	-0.0111	0.6620	0.052*
H01L	0.9315	-0.0520	0.6290	0.052*
C01D	0.876 (2)	-0.2477 (10)	0.4713 (7)	0.055 (5)
H01M	0.8630	-0.3052	0.4596	0.066*
C01E	0.9251 (14)	0.0185 (9)	0.5239 (7)	0.039 (4)
H01N	1.0130	0.0290	0.5333	0.047*
H01O	0.9028	0.0548	0.4894	0.047*
C01F	0.7971 (16)	-0.1029 (9)	0.4838 (6)	0.042 (4)
H01P	0.7302	-0.0640	0.4808	0.050*
C01G	0.8745 (16)	0.1384 (9)	0.1431 (8)	0.047 (4)
H01Q	0.8868	0.1180	0.1037	0.056*
C01H	0.986 (2)	-0.2223 (11)	0.4944 (10)	0.080 (7)
H01R	1.0518	-0.2619	0.4984	0.096*
C01I	0.8366 (18)	0.2526 (10)	0.8055 (8)	0.053 (4)
H01S	0.8607	0.2616	0.7629	0.064*
C01J	0.9619 (17)	0.1235 (10)	0.1877 (8)	0.049 (4)
H01T	1.0339	0.0918	0.1783	0.059*
C01K	1.002 (2)	-0.1372 (12)	0.5124 (9)	0.069 (6)
H01U	1.0794	-0.1206	0.5290	0.083*

Atomic displacement parameters (Å^2) for (p212121_a)

	U^{11}	U^{22}	U^{33}	U^{12}	U^{13}	U^{23}
Cl01	0.063 (3)	0.0325 (19)	0.063 (3)	0.0024 (19)	-0.014 (2)	0.0050 (18)
Cl02	0.071 (3)	0.0324 (18)	0.061 (3)	-0.007 (2)	-0.001 (2)	0.0001 (18)
Cl03	0.056 (2)	0.038 (2)	0.068 (3)	-0.0039 (18)	-0.001 (2)	0.0075 (19)
O004	0.043 (6)	0.015 (4)	0.044 (6)	0.001 (4)	0.001 (5)	0.004 (4)
O005	0.048 (6)	0.030 (5)	0.034 (5)	-0.006 (5)	0.004 (5)	0.007 (4)
O006	0.048 (6)	0.026 (5)	0.049 (6)	0.009 (5)	0.000 (5)	-0.003 (4)
O007	0.055 (6)	0.022 (5)	0.041 (6)	0.006 (5)	-0.001 (5)	-0.005 (4)
O008	0.058 (7)	0.027 (5)	0.031 (6)	0.001 (5)	0.004 (5)	0.000 (4)
O009	0.046 (6)	0.024 (5)	0.046 (6)	-0.004 (4)	-0.013 (5)	0.004 (4)
O00A	0.055 (7)	0.017 (4)	0.053 (6)	-0.003 (4)	-0.015 (5)	0.003 (4)
O00B	0.030 (5)	0.039 (5)	0.040 (6)	-0.007 (4)	-0.001 (4)	-0.007 (4)
O00C	0.039 (6)	0.028 (5)	0.047 (6)	0.001 (4)	-0.011 (5)	-0.003 (4)
O00D	0.039 (6)	0.035 (5)	0.042 (6)	-0.010 (5)	0.000 (5)	0.006 (4)
N00E	0.046 (7)	0.030 (6)	0.029 (6)	0.003 (5)	0.002 (5)	0.003 (5)
C1	0.043 (8)	0.018 (6)	0.038 (8)	0.005 (6)	-0.010 (7)	-0.008 (6)
C00G	0.046 (9)	0.027 (7)	0.033 (8)	-0.008 (7)	0.001 (7)	0.005 (6)
N00H	0.047 (7)	0.020 (6)	0.036 (7)	-0.005 (5)	0.003 (6)	-0.001 (5)
C00I	0.045 (8)	0.021 (6)	0.029 (7)	-0.010 (6)	-0.001 (6)	0.007 (5)
C00J	0.035 (8)	0.023 (7)	0.043 (9)	-0.003 (6)	-0.001 (7)	-0.005 (6)
C00K	0.041 (8)	0.013 (6)	0.029 (7)	0.003 (5)	-0.003 (6)	-0.002 (5)
C00L	0.032 (8)	0.021 (6)	0.046 (9)	0.000 (6)	0.006 (7)	-0.005 (6)
C00M	0.042 (8)	0.020 (7)	0.039 (8)	-0.005 (6)	0.003 (6)	-0.001 (6)
C00N	0.036 (8)	0.047 (9)	0.044 (9)	-0.014 (7)	0.000 (7)	-0.003 (7)
C00O	0.048 (9)	0.017 (6)	0.043 (9)	-0.005 (6)	-0.002 (7)	-0.001 (6)
C00P	0.030 (7)	0.016 (6)	0.030 (7)	-0.004 (5)	-0.004 (6)	0.004 (5)
C00Q	0.038 (8)	0.016 (6)	0.033 (7)	-0.003 (5)	-0.002 (6)	-0.006 (5)
C00R	0.043 (8)	0.015 (6)	0.036 (8)	-0.010 (6)	-0.005 (6)	0.002 (5)
C00S	0.057 (10)	0.017 (6)	0.057 (10)	-0.010 (7)	-0.010 (8)	-0.002 (7)
C00T	0.043 (8)	0.028 (7)	0.025 (7)	0.005 (6)	0.005 (6)	-0.006 (6)

C00U	0.029 (8)	0.051 (9)	0.037 (8)	-0.001 (7)	0.005 (6)	-0.006 (7)
C00V	0.039 (8)	0.013 (6)	0.040 (8)	-0.003 (5)	0.004 (6)	0.002 (5)
C00W	0.040 (9)	0.020 (7)	0.041 (8)	-0.002 (6)	0.000 (7)	0.007 (6)
C00X	0.034 (7)	0.025 (6)	0.032 (7)	0.000 (6)	0.000 (6)	0.004 (6)
C00Y	0.037 (8)	0.013 (6)	0.042 (8)	0.006 (5)	0.005 (6)	0.000 (5)
C00Z	0.037 (8)	0.022 (6)	0.025 (7)	-0.004 (6)	-0.004 (6)	0.009 (5)
C010	0.026 (7)	0.025 (7)	0.044 (8)	0.003 (6)	0.002 (6)	-0.002 (6)
C011	0.043 (9)	0.031 (8)	0.033 (8)	0.005 (7)	-0.008 (6)	-0.001 (6)
C012	0.047 (9)	0.033 (8)	0.032 (8)	-0.001 (6)	-0.001 (7)	-0.001 (6)
C013	0.030 (7)	0.032 (7)	0.027 (7)	-0.001 (6)	-0.008 (6)	0.003 (5)
C014	0.032 (8)	0.024 (6)	0.048 (9)	0.000 (6)	-0.001 (7)	0.000 (6)
C015	0.043 (8)	0.021 (6)	0.027 (7)	-0.001 (6)	0.002 (6)	0.005 (5)
C016	0.049 (10)	0.023 (7)	0.060 (10)	0.004 (7)	0.002 (8)	-0.009 (7)
C017	0.032 (7)	0.029 (7)	0.030 (7)	-0.006 (6)	-0.002 (6)	0.005 (6)
C018	0.047 (9)	0.033 (7)	0.025 (7)	0.005 (6)	-0.005 (6)	-0.005 (6)
C019	0.056 (11)	0.056 (11)	0.081 (13)	-0.017 (9)	0.021 (10)	-0.029 (9)
C01A	0.046 (9)	0.024 (7)	0.051 (9)	-0.002 (7)	0.001 (7)	0.008 (6)
C01B	0.048 (9)	0.018 (6)	0.043 (8)	-0.002 (6)	0.004 (7)	-0.008 (6)
C01C	0.061 (11)	0.026 (7)	0.043 (9)	-0.004 (7)	-0.003 (7)	0.000 (6)
C01D	0.101 (15)	0.018 (7)	0.046 (9)	0.004 (9)	0.007 (10)	0.004 (7)
C01E	0.043 (9)	0.027 (7)	0.047 (9)	-0.003 (6)	0.008 (7)	-0.009 (6)
C01F	0.050 (10)	0.035 (8)	0.041 (9)	0.013 (7)	0.005 (7)	-0.009 (6)
C01G	0.061 (11)	0.021 (7)	0.059 (10)	-0.005 (7)	0.007 (9)	0.000 (7)
C01H	0.099 (17)	0.043 (10)	0.099 (16)	0.036 (11)	-0.040 (13)	-0.029 (10)
C01I	0.079 (12)	0.043 (9)	0.038 (9)	-0.005 (9)	-0.005 (8)	0.011 (7)
C01J	0.055 (10)	0.036 (8)	0.055 (10)	-0.003 (8)	0.011 (9)	-0.009 (7)
C01K	0.072 (13)	0.055 (11)	0.081 (13)	0.027 (10)	-0.016 (10)	-0.025 (10)

Geometric parameters (Å, º) for (p212121_a)

Cl01—C01I	1.781 (19)	C00T—C012	1.516 (19)
Cl02—C01I	1.769 (17)	C00T—C015	1.532 (18)
Cl03—C01I	1.711 (18)	C00T—H00F	0.9800
O004—C00J	1.327 (16)	C00U—C016	1.33 (2)
O004—C010	1.390 (16)	C00U—C00W	1.41 (2)
O005—C00J	1.192 (16)	C00U—H00G	0.9300
O006—C00G	1.190 (17)	C00V—C01E	1.498 (19)
O007—C00M	1.222 (15)	C00V—C01C	1.51 (2)
O008—C00G	1.315 (16)	C00V—H00H	0.9800
O008—C012	1.424 (16)	C00W—C00Y	1.372 (19)
O009—C011	1.298 (16)	C00W—H00I	0.9300
O009—C01C	1.450 (17)	C00Y—C010	1.404 (18)
O00A—C011	1.235 (16)	C00Y—C013	1.473 (18)
O00B—C00R	1.342 (15)	C00Z—C013	1.543 (18)
O00B—C017	1.402 (16)	C010—C01B	1.404 (18)
O00C—C00X	1.211 (16)	C012—H01A	0.9700
O00D—C00R	1.201 (16)	C012—H01B	0.9700
N00E—C00M	1.358 (17)	C013—H01C	0.9800
N00E—C00G	1.404 (18)	C014—C01F	1.35 (2)
N00E—C00T	1.455 (17)	C014—C01K	1.38 (2)
C1—C00S	1.36 (2)	C014—C01E	1.508 (19)
C1—C00I	1.371 (18)	C015—H01D	0.9700
C1—H1A	0.9300	C015—H01E	0.9700
N00H—C011	1.360 (18)	C016—C01B	1.35 (2)
N00H—C00X	1.386 (17)	C016—H01F	0.9300
N00H—C00V	1.463 (16)	C017—C018	1.358 (18)
C00I—C01A	1.42 (2)	C018—H01G	0.9300

C00I—C015	1.495 (18)	C019—C01D	1.37 (3)
C00J—C00Z	1.520 (18)	C019—C01F	1.37 (2)
C00K—C00R	1.461 (18)	C019—H01H	0.9300
C00K—C00X	1.514 (17)	C01A—C01J	1.36 (2)
C00K—C00Q	1.525 (19)	C01A—H01I	0.9300
C00K—C013	1.604 (17)	C01B—H01J	0.9300
C00L—C00N	1.40 (2)	C01C—H01K	0.9700
C00L—C00P	1.402 (18)	C01C—H01L	0.9700
C00L—H00A	0.9300	C01D—C01H	1.34 (3)
C00M—C00Z	1.513 (19)	C01D—H01M	0.9300
C00N—C00O	1.36 (2)	C01E—H01N	0.9700
C00N—H00B	0.9300	C01E—H01O	0.9700
C00O—C018	1.40 (2)	C01F—H01P	0.9300
C00O—H00C	0.9300	C01G—C01J	1.37 (2)
C00P—C017	1.388 (18)	C01G—H01Q	0.9300
C00P—C00Q	1.490 (17)	C01H—C01K	1.38 (3)
C00Q—C00Z	1.571 (17)	C01H—H01R	0.9300
C00Q—H00D	0.9800	C01I—H01S	0.9800
C00S—C01G	1.36 (2)	C01J—H01T	0.9300
C00S—H00E	0.9300	C01K—H01U	0.9300
C00J—O004—C010	121.7 (10)	C00J—C00Z—C00Q	113.3 (10)
C00G—O008—C012	110.1 (11)	C013—C00Z—C00Q	88.7 (9)
C011—O009—C01C	109.0 (11)	O004—C010—C01B	114.0 (11)
C00R—O00B—C017	121.4 (11)	O004—C010—C00Y	124.5 (11)
C00M—N00E—C00G	126.5 (11)	C01B—C010—C00Y	121.5 (12)
C00M—N00E—C00T	122.8 (11)	O00A—C011—O009	124.0 (12)
C00G—N00E—C00T	110.7 (11)	O00A—C011—N00H	125.1 (12)
C00S—C1—C00I	121.1 (14)	O009—C011—N00H	110.9 (12)
C00S—C1—H1A	119.4	O008—C012—C00T	105.9 (11)
C00I—C1—H1A	119.4	O008—C012—H01A	110.6
O006—C00G—O008	125.8 (13)	C00T—C012—H01A	110.6
O006—C00G—N00E	125.8 (13)	O008—C012—H01B	110.6
O008—C00G—N00E	108.4 (12)	C00T—C012—H01B	110.6
C011—N00H—C00X	128.2 (11)	H01A—C012—H01B	108.7
C011—N00H—C00V	110.4 (11)	C00Y—C013—C00Z	114.5 (11)
C00X—N00H—C00V	121.4 (11)	C00Y—C013—C00K	113.3 (10)
C1—C00I—C01A	117.4 (13)	C00Z—C013—C00K	88.8 (9)
C1—C00I—C015	122.6 (13)	C00Y—C013—H01C	112.7
C01A—C00I—C015	120.0 (13)	C00Z—C013—H01C	112.7
O005—C00J—O004	118.4 (12)	C00K—C013—H01C	112.7
O005—C00J—C00Z	122.3 (12)	C01F—C014—C01K	117.1 (14)
O004—C00J—C00Z	119.2 (12)	C01F—C014—C01E	119.5 (13)
C00R—C00K—C00X	110.1 (10)	C01K—C014—C01E	123.3 (14)
C00R—C00K—C00Q	120.8 (11)	C00I—C015—C00T	111.3 (10)
C00X—C00K—C00Q	112.2 (11)	C00I—C015—H01D	109.4
C00R—C00K—C013	113.5 (10)	C00T—C015—H01D	109.4
C00X—C00K—C013	110.2 (10)	C00I—C015—H01E	109.4
C00Q—C00K—C013	88.1 (9)	C00T—C015—H01E	109.4
C00N—C00L—C00P	120.9 (13)	H01D—C015—H01E	108.0
C00N—C00L—H00A	119.5	C00U—C016—C01B	122.8 (14)
C00P—C00L—H00A	119.5	C00U—C016—H01F	118.6
O007—C00M—N00E	119.1 (12)	C01B—C016—H01F	118.6
O007—C00M—C00Z	121.4 (12)	C018—C017—C00P	123.9 (12)
N00E—C00M—C00Z	119.4 (11)	C018—C017—O00B	114.5 (12)
C00O—C00N—C00L	120.0 (13)	C00P—C017—O00B	121.6 (11)
C00O—C00N—H00B	120.0	C017—C018—C00O	118.6 (13)

C00L—C00N—H00B	120.0	C017—C018—H01G	120.7
C00N—C00O—C018	120.3 (13)	C00O—C018—H01G	120.7
C00N—C00O—H00C	119.9	C01D—C019—C01F	121.7 (17)
C018—C00O—H00C	119.9	C01D—C019—H01H	119.2
C017—C00P—C00L	116.1 (11)	C01F—C019—H01H	119.2
C017—C00P—C00Q	123.1 (11)	C01J—C01A—C00I	119.5 (15)
C00L—C00P—C00Q	120.7 (12)	C01J—C01A—H01I	120.3
C00P—C00Q—C00K	111.1 (11)	C00I—C01A—H01I	120.3
C00P—C00Q—C00Z	115.4 (10)	C016—C01B—C010	117.8 (13)
C00K—C00Q—C00Z	90.6 (10)	C016—C01B—H01J	121.1
C00P—C00Q—H00D	112.6	C010—C01B—H01J	121.1
C00K—C00Q—H00D	112.6	O009—C01C—C00V	104.5 (11)
C00Z—C00Q—H00D	112.6	O009—C01C—H01K	110.9
O00D—C00R—O00B	116.7 (12)	C00V—C01C—H01K	110.9
O00D—C00R—C00K	123.7 (12)	O009—C01C—H01L	110.9
O00B—C00R—C00K	119.5 (11)	C00V—C01C—H01L	110.9
C01G—C00S—C1	121.8 (15)	H01K—C01C—H01L	108.9
C01G—C00S—H00E	119.1	C01H—C01D—C019	118.6 (15)
C1—C00S—H00E	119.1	C01H—C01D—H01M	120.7
N00E—C00T—C012	98.7 (10)	C019—C01D—H01M	120.7
N00E—C00T—C015	112.5 (11)	C00V—C01E—C014	112.4 (12)
C012—C00T—C015	114.8 (11)	C00V—C01E—H01N	109.1
N00E—C00T—H00F	110.1	C014—C01E—H01N	109.1
C012—C00T—H00F	110.1	C00V—C01E—H01O	109.1
C015—C00T—H00F	110.1	C014—C01E—H01O	109.1
C016—C00U—C00W	120.1 (13)	H01N—C01E—H01O	107.8
C016—C00U—H00G	120.0	C014—C01F—C019	120.5 (15)
C00W—C00U—H00G	120.0	C014—C01F—H01P	119.7
N00H—C00V—C01E	111.9 (11)	C019—C01F—H01P	119.7
N00H—C00V—C01C	99.3 (10)	C00S—C01G—C01J	118.1 (15)
C01E—C00V—C01C	114.0 (12)	C00S—C01G—H01Q	120.9
N00H—C00V—H00H	110.4	C01J—C01G—H01Q	120.9
C01E—C00V—H00H	110.4	C01D—C01H—C01K	119.8 (17)
C01C—C00V—H00H	110.4	C01D—C01H—H01R	120.1
C00Y—C00W—C00U	120.3 (12)	C01K—C01H—H01R	120.1
C00Y—C00W—H00I	119.9	Cl03—C01I—Cl02	110.9 (9)
C00U—C00W—H00I	119.9	Cl03—C01I—Cl01	111.7 (8)
O00C—C00X—N00H	119.1 (12)	Cl02—C01I—Cl01	108.4 (10)
O00C—C00X—C00K	123.9 (11)	Cl03—C01I—H01S	108.6
N00H—C00X—C00K	117.1 (11)	Cl02—C01I—H01S	108.6
C00W—C00Y—C010	117.6 (11)	Cl01—C01I—H01S	108.6
C00W—C00Y—C013	122.7 (11)	C01A—C01J—C01G	122.0 (16)
C010—C00Y—C013	119.7 (12)	C01A—C01J—H01T	119.0
C00M—C00Z—C00J	109.1 (11)	C01G—C01J—H01T	119.0
C00M—C00Z—C013	115.3 (11)	C01H—C01K—C014	122.2 (18)
C00J—C00Z—C013	117.7 (11)	C01H—C01K—H01U	118.9
C00M—C00Z—C00Q	111.5 (10)	C014—C01K—H01U	118.9

Document origin: *publCIF* [Westrip, S. P. (2010). *J. Apply. Cryst.*, **43**, 920-925].

syn-HT **2CF**

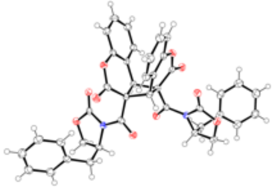

Crystal data

$C_{40}H_{30}N_2O_{10}$	D_x = 1.457 Mg m^{-3}
M_r = 698.67	Mo *K*a radiation, l = 0.71073 Å
Orthorhombic, $P2_12_12_1$	Cell parameters from 2436 reflections
a = 8.2036 (10) Å	q = 2.2–19.4°
b = 18.123 (2) Å	m = 0.11 mm^{-1}
c = 21.423 (3) Å	*T* = 296 K
V = 3185.0 (7) Å^3	, clear colourless
Z = 4	0.40 × 0.20 × 0.20 mm
F(000) = 1456	

Data collection

Bruker D8 goniometer diffractometer	2755 independent reflections
Radiation source: sealed tube	2538 reflections with $I > 2s(I)$
Graphite monochromator	R_{int} = 0.036
Detector resolution: 8.3333 pixels mm^{-1}	q_{max} = 19.5°, q_{min} = 1.5°
w scans	*h* = -7®7
Absorption correction: multi-scan *SADABS* V2008/1 (Bruker AXS)	*k* = -16®16
T_{min} = 0.89, T_{max} = 0.98	*l* = -20®16
8883 measured reflections	

Refinement

Refinement on F^2	Secondary atom site location: difference Fourier map
Least-squares matrix: full	Hydrogen site location: inferred from neighbouring sites
$R[F^2 > 2s(F^2)]$ = 0.055	H atoms treated by a mixture of independent and constrained refinement
$wR(F^2)$ = 0.147	$w = 1/[s^2(F_o^2) + (0.074P)^2 + 9.3449P]$ where $P = (F_o^2 + 2F_c^2)/3$
S = 1.07	$(D/s)_{max}$ = 0.050
2755 reflections	$D\rho_{max}$ = 0.40 e Å^{-3}
469 parameters	$D\rho_{min}$ = -0.22 e Å^{-3}
0 restraints	Absolute structure: Flack H D (1983), Acta Cryst. A39, 876-881
Primary atom site location: structure-invariant direct methods	Absolute structure parameter: 0 (2)

Special details

Geometry. All esds (except the esd in the dihedral angle between two l.s. planes) are estimated using the full covariance matrix. The cell esds are taken into account individually in the estimation of esds in distances, angles and torsion angles; correlations between esds in cell parameters are only used when they are defined by crystal symmetry. An approximate (isotropic) treatment of cell esds is used for estimating esds involving l.s. planes.

Refinement. Refinement of F^2 against ALL reflections. The weighted R-factor wR and goodness of fit S are based on F^2, conventional R-factors R are based on F, with F set to zero for negative F^2. The threshold expression of F^2 > 2sigma(F^2) is used only for calculating R-factors(gt) etc. and is not relevant to the choice of reflections for refinement. R-factors based on F^2 are statistically about twice as large as those based on F, and R-factors based on ALL data will be even larger.

Fractional atomic coordinates and isotropic or equivalent isotropic displacement parameters (Å²) for (I)

	x	*y*	*z*	U_{iso}*/U_{eq}
O2	0.2890 (6)	0.4432 (3)	0.2289 (2)	0.0250 (13)
N4	0.2939 (7)	0.5231 (3)	0.3088 (3)	0.0190 (15)
C3	0.2707 (9)	0.5066 (5)	0.2470 (3)	0.0208 (19)
C4	0.2695 (9)	0.5901 (5)	0.3386 (4)	0.025 (2)
O1	0.2888 (6)	0.5826 (3)	0.3994 (2)	0.0246 (13)
O3	0.2344 (6)	0.6483 (3)	0.3133 (2)	0.0266 (13)
C5	0.3075 (10)	0.5041 (4)	0.4161 (3)	0.025 (2)
C6	0.3404 (10)	0.4656 (4)	0.3537 (3)	0.027 (2)
C12	0.8425 (9)	0.5244 (5)	0.4503 (4)	0.033 (2)
C7	0.5194 (9)	0.4372 (4)	0.3452 (4)	0.026 (2)
C13	0.7241 (11)	0.4756 (5)	0.4263 (4)	0.035 (2)
C9	0.6918 (10)	0.5543 (4)	0.3364 (4)	0.031 (2)
C11	0.8840 (9)	0.5887 (5)	0.4163 (4)	0.034 (2)
C10	0.8085 (9)	0.6035 (4)	0.3593 (4)	0.030 (2)
C8	0.6482 (9)	0.4903 (4)	0.3693 (4)	0.026 (2)
O4	0.4910 (7)	0.6141 (3)	0.2098 (2)	0.0266 (13)
C14	0.3493 (11)	0.6275 (4)	0.2014 (3)	0.0185 (18)
O5	0.3073 (6)	0.6967 (3)	0.1854 (2)	0.0235 (13)
C18	0.1438 (9)	0.7199 (5)	0.1854 (3)	0.022 (2)
C16	0.0382 (8)	0.5919 (4)	0.2110 (3)	0.0205 (19)
C17	0.0168 (10)	0.6731 (4)	0.1985 (3)	0.0227 (19)
C15	0.2197 (9)	0.5694 (4)	0.2024 (3)	0.0228 (19)
C22	-0.1409 (10)	0.7038 (4)	0.2014 (3)	0.031 (2)
C20	-0.0274 (10)	0.8246 (4)	0.1764 (3)	0.026 (2)
C21	-0.1634 (10)	0.7798 (4)	0.1905 (3)	0.030 (2)
C19	0.1230 (11)	0.7950 (4)	0.1731 (3)	0.026 (2)
O6	-0.1004 (6)	0.4578 (3)	0.2365 (3)	0.0258 (13)
O7	-0.2736 (7)	0.5564 (3)	0.1132 (2)	0.0290 (13)
O8	-0.0762 (6)	0.6156 (3)	0.0646 (2)	0.0230 (13)
C30	-0.0088 (9)	0.5394 (4)	0.1553 (3)	0.0181 (19)
C25	0.1201 (10)	0.6709 (4)	-0.0001 (3)	0.028 (2)
C28	0.0864 (9)	0.6233 (4)	0.0501 (3)	0.022 (2)
C26	0.3709 (10)	0.5976 (4)	0.0609 (3)	0.026 (2)
C31	0.1756 (8)	0.5350 (4)	0.1361 (3)	0.0198 (19)
C27	0.2095 (9)	0.5861 (4)	0.0822 (3)	0.0197 (19)
C23	0.4051 (11)	0.6442 (4)	0.0112 (4)	0.028 (2)
C29	-0.1287 (11)	0.5682 (4)	0.1095 (3)	0.0202 (19)
C24	0.2801 (10)	0.6819 (4)	-0.0196 (3)	0.027 (2)
C32	-0.0704 (9)	0.4655 (4)	0.1817 (4)	0.022 (2)
N5	-0.0924 (7)	0.4071 (3)	0.1412 (3)	0.0192 (15)
O9	-0.0206 (6)	0.4584 (3)	0.0453 (2)	0.0253 (13)
O10	-0.0807 (6)	0.3383 (3)	0.0556 (2)	0.0245 (13)
C37	-0.4933 (9)	0.3964 (4)	0.1017 (3)	0.0232 (19)
C33	-0.0595 (9)	0.4057 (5)	0.0768 (4)	0.024 (2)
C36	-0.3239 (9)	0.3309 (4)	0.1842 (3)	0.0230 (19)
C35	-0.0975 (11)	0.2848 (4)	0.1069 (3)	0.031 (2)
C41	-0.5195 (10)	0.2639 (5)	0.1125 (4)	0.031 (2)
C39	-0.6843 (10)	0.3275 (5)	0.0367 (4)	0.033 (2)

C38	-0.6136 (10)	0.3949 (5)	0.0543 (4)	0.031 (2)
C34	-0.1416 (9)	0.3335 (4)	0.1645 (3)	0.026 (2)
C40	-0.6373 (10)	0.2628 (5)	0.0649 (4)	0.037 (2)
C42	-0.4493 (8)	0.3294 (4)	0.1308 (3)	0.024 (2)

Atomic displacement parameters (Å^2) for (I)

	U^{11}	U^{22}	U^{33}	U^{12}	U^{13}	U^{23}
O2	0.031 (3)	0.024 (3)	0.020 (3)	-0.001 (3)	-0.005 (2)	-0.005 (3)
N4	0.025 (4)	0.013 (4)	0.019 (4)	-0.001 (3)	0.001 (3)	-0.009 (3)
C3	0.014 (4)	0.030 (6)	0.018 (5)	-0.002 (4)	0.002 (4)	-0.001 (4)
C4	0.013 (5)	0.033 (6)	0.028 (6)	-0.006 (4)	0.000 (4)	0.003 (5)
O1	0.030 (3)	0.030 (3)	0.014 (3)	-0.002 (3)	-0.002 (3)	-0.002 (2)
O3	0.033 (3)	0.018 (3)	0.029 (3)	-0.004 (3)	0.000 (3)	-0.001 (3)
C5	0.043 (5)	0.012 (4)	0.019 (5)	0.003 (4)	0.005 (4)	0.006 (3)
C6	0.042 (6)	0.026 (5)	0.014 (4)	0.000 (4)	-0.001 (4)	0.002 (4)
C12	0.014 (5)	0.046 (6)	0.039 (5)	-0.005 (5)	0.001 (4)	0.005 (5)
C7	0.013 (4)	0.032 (5)	0.033 (5)	0.000 (4)	-0.006 (4)	-0.003 (4)
C13	0.035 (6)	0.043 (5)	0.027 (5)	0.016 (5)	0.007 (5)	0.004 (4)
C9	0.021 (5)	0.038 (5)	0.034 (5)	0.014 (5)	0.005 (4)	0.000 (5)
C11	0.016 (5)	0.055 (6)	0.030 (5)	0.011 (5)	-0.005 (4)	0.002 (5)
C10	0.018 (5)	0.036 (5)	0.038 (5)	0.004 (4)	0.007 (5)	-0.007 (4)
C8	0.024 (5)	0.033 (5)	0.021 (5)	0.010 (4)	-0.004 (4)	0.003 (4)
O4	0.022 (4)	0.034 (3)	0.024 (3)	-0.007 (3)	-0.002 (3)	0.002 (3)
C14	0.016 (6)	0.029 (6)	0.010 (4)	-0.004 (4)	-0.002 (4)	0.001 (4)
O5	0.026 (4)	0.017 (3)	0.027 (3)	-0.006 (3)	-0.001 (3)	0.003 (2)
C18	0.003 (5)	0.045 (6)	0.017 (4)	0.003 (5)	0.002 (3)	-0.002 (4)
C16	0.013 (5)	0.019 (5)	0.029 (5)	0.007 (3)	-0.006 (4)	-0.003 (4)
C17	0.020 (5)	0.027 (5)	0.021 (4)	0.009 (5)	-0.003 (4)	-0.001 (4)
C15	0.025 (5)	0.026 (5)	0.017 (4)	0.000 (4)	-0.001 (4)	0.000 (4)
C22	0.037 (6)	0.033 (6)	0.024 (5)	-0.002 (4)	0.000 (4)	-0.013 (4)
C20	0.026 (5)	0.025 (5)	0.026 (5)	0.005 (5)	-0.012 (4)	-0.001 (4)
C21	0.038 (6)	0.023 (5)	0.030 (5)	-0.002 (5)	-0.006 (4)	0.004 (4)
C19	0.043 (6)	0.012 (5)	0.022 (4)	-0.001 (4)	-0.017 (4)	0.000 (4)
O6	0.028 (3)	0.026 (3)	0.023 (4)	-0.002 (3)	0.004 (3)	-0.003 (3)
O7	0.024 (4)	0.029 (3)	0.035 (3)	-0.005 (3)	-0.003 (3)	-0.001 (2)
O8	0.017 (4)	0.025 (3)	0.026 (3)	-0.005 (3)	-0.005 (3)	0.004 (3)
C30	0.016 (5)	0.022 (5)	0.017 (4)	0.000 (4)	-0.004 (4)	0.001 (4)
C25	0.051 (7)	0.020 (5)	0.013 (4)	-0.008 (5)	-0.004 (4)	0.003 (4)
C28	0.018 (6)	0.027 (5)	0.021 (5)	-0.002 (4)	-0.007 (4)	-0.008 (4)
C26	0.040 (6)	0.022 (5)	0.017 (5)	-0.012 (4)	0.003 (4)	-0.004 (4)
C31	0.012 (5)	0.038 (5)	0.010 (4)	-0.001 (4)	0.000 (3)	0.005 (4)
C27	0.017 (5)	0.023 (4)	0.018 (4)	-0.006 (4)	0.007 (4)	0.000 (4)
C23	0.034 (5)	0.021 (5)	0.030 (5)	-0.002 (5)	-0.003 (5)	-0.010 (4)
C29	0.026 (6)	0.015 (5)	0.020 (5)	-0.005 (4)	-0.004 (5)	-0.003 (4)
C24	0.019 (5)	0.029 (5)	0.033 (5)	0.001 (5)	0.002 (4)	-0.010 (4)
C32	0.015 (5)	0.029 (5)	0.022 (6)	0.001 (4)	0.000 (4)	-0.003 (5)
N5	0.020 (4)	0.028 (4)	0.010 (4)	0.003 (3)	0.006 (3)	-0.006 (3)
O9	0.033 (3)	0.025 (3)	0.018 (3)	-0.005 (3)	-0.003 (3)	0.003 (3)
O10	0.031 (3)	0.018 (3)	0.025 (3)	-0.003 (3)	-0.003 (3)	-0.003 (3)
C37	0.018 (4)	0.025 (5)	0.027 (5)	0.003 (4)	0.002 (4)	0.003 (4)
C33	0.016 (5)	0.032 (7)	0.022 (6)	0.004 (4)	-0.006 (4)	-0.005 (5)
C36	0.016 (5)	0.026 (4)	0.028 (4)	-0.001 (4)	-0.004 (4)	0.003 (4)
C35	0.044 (5)	0.028 (5)	0.020 (5)	-0.005 (4)	0.014 (4)	0.002 (4)
C41	0.017 (5)	0.038 (6)	0.038 (5)	-0.008 (4)	0.007 (5)	-0.011 (4)
C39	0.030 (5)	0.042 (6)	0.029 (5)	0.004 (5)	0.005 (4)	0.004 (5)
C38	0.019 (5)	0.045 (6)	0.028 (5)	-0.003 (4)	0.014 (5)	0.002 (4)

C34	0.040 (6)	0.005 (4)	0.032 (5)	-0.006 (4)	0.013 (4)	-0.003 (4)
C40	0.033 (5)	0.049 (6)	0.029 (5)	-0.009 (5)	0.003 (5)	-0.004 (5)
C42	0.016 (5)	0.029 (5)	0.027 (5)	-0.002 (4)	0.007 (4)	-0.003 (4)

Geometric parameters (Å, º) for (I)

O2—C3	1.222 (8)	C20—C21	1.412 (11)
N4—C3	1.370 (9)	O6—C32	1.207 (8)
N4—C4	1.387 (10)	O7—C29	1.211 (9)
N4—C6	1.470 (9)	O8—C29	1.360 (9)
C3—C15	1.544 (11)	O8—C28	1.377 (9)
C4—O3	1.219 (9)	C30—C29	1.484 (10)
C4—O1	1.319 (9)	C30—C32	1.539 (10)
O1—C5	1.476 (8)	C30—C31	1.570 (11)
C5—C6	1.531 (10)	C25—C24	1.392 (11)
C6—C7	1.567 (11)	C25—C28	1.405 (10)
C12—C13	1.411 (11)	C28—C27	1.396 (10)
C12—C11	1.417 (11)	C26—C23	1.387 (10)
C7—C8	1.520 (11)	C26—C27	1.415 (10)
C13—C8	1.396 (11)	C31—C27	1.506 (10)
C9—C8	1.404 (10)	C23—C24	1.398 (11)
C9—C10	1.397 (11)	C32—N5	1.380 (9)
C11—C10	1.394 (11)	N5—C33	1.404 (10)
O4—C14	1.201 (8)	N5—C34	1.480 (9)
C14—O5	1.347 (8)	O9—C33	1.213 (9)
C14—C15	1.496 (10)	O10—C33	1.315 (9)
O5—C18	1.405 (9)	O10—C35	1.472 (9)
C18—C17	1.372 (10)	C37—C38	1.415 (11)
C18—C19	1.397 (10)	C37—C42	1.413 (10)
C16—C17	1.506 (10)	C36—C42	1.537 (10)
C16—C15	1.555 (10)	C36—C34	1.555 (10)
C16—C30	1.574 (10)	C35—C34	1.560 (11)
C17—C22	1.410 (11)	C41—C42	1.376 (11)
C15—C31	1.592 (10)	C41—C40	1.404 (11)
C22—C21	1.408 (11)	C39—C40	1.374 (11)
C20—C19	1.348 (11)	C39—C38	1.403 (11)
C3—N4—C4	128.1 (6)	C29—O8—C28	122.0 (6)
C3—N4—C6	120.9 (6)	C29—C30—C32	109.3 (6)
C4—N4—C6	110.8 (6)	C29—C30—C31	118.9 (6)
O2—C3—N4	119.6 (7)	C32—C30—C31	111.6 (6)
O2—C3—C15	122.0 (7)	C29—C30—C16	116.8 (6)
N4—C3—C15	118.3 (7)	C32—C30—C16	109.2 (6)
O3—C4—O1	123.9 (7)	C31—C30—C16	89.6 (5)
O3—C4—N4	125.8 (7)	C24—C25—C28	120.2 (7)
O1—C4—N4	110.4 (7)	O8—C28—C27	122.7 (7)
C4—O1—C5	110.6 (6)	O8—C28—C25	115.2 (7)
O1—C5—C6	104.2 (5)	C27—C28—C25	122.1 (7)
N4—C6—C5	101.8 (5)	C23—C26—C27	121.7 (7)
N4—C6—C7	113.6 (6)	C27—C31—C30	110.3 (6)
C5—C6—C7	114.6 (6)	C27—C31—C15	113.6 (6)
C13—C12—C11	119.6 (7)	C30—C31—C15	88.1 (5)
C8—C7—C6	113.8 (6)	C28—C27—C26	116.5 (6)

C8—C13—C12	120.4 (7)	C28—C27—C31	122.8 (6)
C8—C9—C10	121.6 (7)	C26—C27—C31	120.7 (6)
C10—C11—C12	120.1 (8)	C26—C23—C24	120.8 (7)
C11—C10—C9	119.4 (7)	O7—C29—O8	118.0 (7)
C9—C8—C13	119.0 (7)	O7—C29—C30	123.0 (7)
C9—C8—C7	122.0 (7)	O8—C29—C30	118.7 (7)
C13—C8—C7	119.0 (7)	C25—C24—C23	118.7 (7)
O4—C14—O5	118.3 (7)	O6—C32—N5	119.8 (7)
O4—C14—C15	122.9 (7)	O6—C32—C30	121.6 (7)
O5—C14—C15	118.5 (7)	N5—C32—C30	118.6 (7)
C14—O5—C18	121.5 (6)	C32—N5—C33	127.3 (7)
C17—C18—C19	123.2 (7)	C32—N5—C34	120.9 (6)
C17—C18—O5	122.7 (7)	C33—N5—C34	111.6 (6)
C19—C18—O5	114.0 (7)	C33—O10—C35	111.5 (6)
C17—C16—C15	110.3 (6)	C38—C37—C42	118.6 (7)
C17—C16—C30	115.3 (6)	O9—C33—O10	125.1 (7)
C15—C16—C30	89.2 (5)	O9—C33—N5	125.6 (7)
C18—C17—C22	117.5 (7)	O10—C33—N5	109.3 (7)
C18—C17—C16	123.5 (7)	C42—C36—C34	116.3 (6)
C22—C17—C16	119.0 (7)	O10—C35—C34	103.9 (6)
C14—C15—C3	109.6 (6)	C42—C41—C40	120.5 (8)
C14—C15—C16	119.8 (6)	C40—C39—C38	120.6 (7)
C3—C15—C16	112.3 (6)	C39—C38—C37	119.8 (7)
C14—C15—C31	115.1 (6)	N5—C34—C36	112.4 (6)
C3—C15—C31	109.0 (6)	N5—C34—C35	100.3 (6)
C16—C15—C31	89.4 (5)	C36—C34—C35	114.8 (6)
C21—C22—C17	119.9 (7)	C39—C40—C41	120.0 (8)
C19—C20—C21	120.3 (7)	C41—C42—C37	120.5 (7)
C22—C21—C20	119.6 (7)	C41—C42—C36	120.5 (7)
C20—C19—C18	119.4 (7)	C37—C42—C36	119.0 (7)

Document origin: *publCIF* [Westrip, S. P. (2010). *J. Apply. Cryst.*, **43**, 920-925].

References

1. T. Hintermann, D. Seebach, *Helv. Chim. Acta* **1998**, *81*, 2093.
2. H.-J. Yoon, M. Dakanali, D. Lichlyter, W. M. Chang, K. A. Nguyen, M. E. Nipper, M. A. Haidekker, and E. A. Theodorakis, *Org. Biomol. Chem.*, **2011**, *9*, 3530.
3. K. Itoh, R. Kato, D. Kinugawa, H. Kamiya, R. Kudo, M. Hasegawa, H. Fujii, and H. Suga, *Org. Biomol. Chem.*, **2015**, *13*, 8919.
